# Cell-type-specific expression quantitative trait loci associated with Alzheimer disease in blood and brain tissue

**DOI:** 10.1038/s41398-021-01373-z

**Published:** 2021-04-27

**Authors:** Devanshi Patel, Xiaoling Zhang, John J. Farrell, Jaeyoon Chung, Thor D. Stein, Kathryn L. Lunetta, Lindsay A. Farrer

**Affiliations:** 1 Bioinformatics Graduate Program, Boston University, Boston, MA USA; 2 Department of Medicine (Biomedical Genetics), Boston University School of Medicine, Boston, MA USA; 3 Department of Biostatistics, Boston University School of Public Health, Boston, MA USA; 4 Department of Pathology & Laboratory Medicine, Boston University School of Medicine, Boston, MA USA; 5 VA Boston Healthcare System, Boston, MA USA; 6 Department of Veterans Affairs Medical Center, Bedford, MA USA; 7 Departments of Neurology and Ophthalmology, Boston University School of Medicine, Boston, MA USA; 8 Department of Epidemiology, Boston University School of Public Health, Boston, MA USA

**Keywords:** Psychiatric disorders, Genomics

## Abstract

Because regulation of gene expression is heritable and context-dependent, we investigated AD-related gene expression patterns in cell types in blood and brain. Cis-expression quantitative trait locus (eQTL) mapping was performed genome-wide in blood from 5257 Framingham Heart Study (FHS) participants and in brain donated by 475 Religious Orders Study/Memory & Aging Project (ROSMAP) participants. The association of gene expression with genotypes for all cis SNPs within 1 Mb of genes was evaluated using linear regression models for unrelated subjects and linear-mixed models for related subjects. Cell-type-specific eQTL (ct-eQTL) models included an interaction term for the expression of “proxy” genes that discriminate particular cell type. Ct-eQTL analysis identified 11,649 and 2533 additional significant gene-SNP eQTL pairs in brain and blood, respectively, that were not detected in generic eQTL analysis. Of note, 386 unique target eGenes of significant eQTLs shared between blood and brain were enriched in apoptosis and Wnt signaling pathways. Five of these shared genes are established AD loci. The potential importance and relevance to AD of significant results in myeloid cell types is supported by the observation that a large portion of GWS ct-eQTLs map within 1 Mb of established AD loci and 58% (23/40) of the most significant eGenes in these eQTLs have previously been implicated in AD. This study identified cell-type-specific expression patterns for established and potentially novel AD genes, found additional evidence for the role of myeloid cells in AD risk, and discovered potential novel blood and brain AD biomarkers that highlight the importance of cell-type-specific analysis.

## Introduction

Recent expression quantitative trait locus (eQTL) analysis studies suggest that changes in gene expression have a role in the pathogenesis of AD^1,2^. However, regulation of gene expression, as well as many biological functions, has been shown to be context-specific (e.g., tissue and cell types, developmental time point, sex, disease status, and response to treatment or stimulus)^3–6^. One study of 500 healthy subjects found over-representation of T cell-specific eQTLs in susceptibility alleles for autoimmune disease and AD risk alleles polarized for monocyte-specific eQTL effects^7^. In addition, disease and trait-associated cis-eQTLs were more cell-type-specific than average cis-eQTLs^7^. Another study classified 12% of more than 23,000 eQTLs in blood as cell-type-specific^4^. Large eQTL studies across multiple human tissues have been conducted by the GTEx consortium, with a study on genetic effects on gene expression levels across 44 human tissues collected from the same donors characterizing patterns of tissue specificity recently published^8^.

Microglia, monocytes, and macrophages share a similar developmental lineage and are all considered to be myeloid cells^9^. It is believed that a large proportion of AD genetic risk can be explained by genes expressed in myeloid cells and not other cell types^10^. Several established AD genes are highly expressed in microglia^9,11^, and a variant in the AD-associated gene *CELF1* has been associated with lower expression of *SPI1* in monocytes and macrophages^10^. AD risk alleles have been shown to be enriched in myeloid-specific epigenomic annotations and in active enhancers of monocytes, macrophages, and microglia^12^, and to be polarized for cis-eQTL effects in monocytes^7^. These findings suggest that a cell-type-specific analysis in blood and brain tissue may identify novel and more precise AD associations that may help elucidate regulatory networks. In this study, we performed a genome-wide *cis* ct-eQTL analysis in blood and brain, respectively, then compared eQTLs and cell-type-specific eQTLs (ct-eQTLs) between brain and blood with a focus on genes, loci, and cell types previously implicated in AD risk by genetic approaches.

## Materials, subjects and methods

### Study cohorts

#### Framingham Heart Study (FHS)

The FHS is a multigenerational study of health and disease in a prospectively followed community-based and primarily non-Hispanic white sample. Procedures for assessing dementia and determining AD status in this cohort are described elsewhere^13^. Clinical, demographic, and pedigree information, as well as 1000 Genomes Project Phase 1 imputed SNP genotypes and Affymetrix Human Exon 1.0 ST array gene expression data from whole blood, were obtained from dbGaP (https://www.ncbi.nlm.nih.gov/projects/gap/cgi-bin/study.cgi?study_id=phs000007.v31.p12). Requisite information for this study was available for 5257 participants. Characteristics of these subjects are provided in Supplementary Table S1.

#### Religious Orders Study (ROS)/Memory and Aging Project (MAP)

ROS enrolled older nuns and priests from across the US, without known dementia for longitudinal clinical analysis and brain donation and MAP enrolled older subjects without dementia from retirement homes who agreed to brain donation at the time of death^14^ (http://www.eurekaselect.com/99959/article). RNA-sequencing brain gene expression and whole-genome sequencing (WGS) genotype data were obtained from the AMP-AD knowledge portal (https://www.synapse.org/#!Synapse:syn3219045) (https://www.synapse.org/).

### Data processing

Generation, initial quality control (QC), and pre-processing procedures of the FHS GWAS and expression data are described elsewhere^13^. Briefly, the Robust Multichip Average (RMA) method^15,16^ was used for background adjustment and normalization of gene expression levels and further adjusted for the first principal component of ancestry. ROSMAP gene expression data were log-normalized and adjusted for known and hidden variables detected by surrogate variable analysis (SVA)^17^ in order to determine which of these variables should be included as covariates in analysis models for eQTL discovery. Additional filtering steps of FHS and ROSMAP GWAS and gene expression data included eliminating subjects with missing data, restricting gene expression data to protein-coding genes, and retaining common variants (MAF ≥ 0.05) with good imputation quality (*R*^2^ ≥ 0.3).

### Cis-eQTL mapping

Cis-eQTL mapping was performed using a genome-wide design (Supplementary Fig. S1). The association of gene expression with SNP genotypes for all cis SNPs within 1 Mb of protein-coding genes was evaluated using linear-mixed models adjusting for family structure in FHS and linear regression models for unrelated individuals in ROSMAP. In FHS, lmekin function in the R kinship package (version 1.1.3)^18^ was applied assuming an additive genetic model with covariates for age and sex, and family structure modeled as a random-effects term for kinship—a matrix of kinship coefficients calculated from pedigree structures. The linear model for analysis of FHS can data be expressed as follows:

$$Y_i = I + \beta _{\it{1}}G_j + \beta _{\it{2}}A_{ij} + \beta _{\it{3}}S_{ij} + U_{ij} + \varepsilon _{ij}$$

where *Y*_*i*_ is the expression value for gene *i*, *G*_*j*_ is the genotype dosage for cis SNP j, *Aij* and *S*_*ij*_ are the covariates for age and sex respectively, *U*_*ij*_ is the random effect for family structure, and *β*_*1*_, *β*_*2*_, and *β*_*3*_ are regression coefficients.

ROSMAP data were analyzed using the lm function in the base stats package in R (http://www.R-project.org/). The regression model, which included covariates for age, sex, postmortem interval (PMI), study (ROS or MAP), and a term for a surrogate variable (SV1) derived from analysis of high dimensional data, can be expressed as:

$$Y_i = I + \beta _{\it{1}}G_j + \beta _{\it{2}}A_{ij} + \beta _{\it{3}}S_{ij} + \beta _{\it{3}}S_{ij} + \beta _{\it{4}}PM_{ij} + \beta _{{\it{5}}ij}{\it{S2}} + \beta _{{\it{6i}}j}{\it{SV1}} + \varepsilon _{ij}$$

where *Y*_*i*_ is the expression value for gene *i*, *G*_*j*_ is the genotype dosage for cis SNP j, *Aij, S*_*ij*_, *PM*_*ij*_, *S2*_*ij*_, and *SV1*_*ij*_ are the covariates for age, sex, PMI, study, and SV1, respectively, ɛ_ij_ is the residual error, and the *β*s are regression coefficients.

### Cis ct-eQTL mapping

Models testing associations with cell-type-specific eQTLs (ct-eQTLs) included an interaction term for expression levels of “proxy” genes that represent cell types. Proxy genes representing ten cell types in whole blood^4^ and five cell types in brain^19–21^ were incorporated in cell-type-specific models (Supplementary Table S2). These proxy genes for cell types in blood were established previously using BLUEPRINT expression data to validate cell-type-specific expression in each cell-type module^4^ and the proxy genes for brain cell types have been incorporated in several studies^19–21^. Cell-type-specific expression analyses in blood of FHS participants were conducted using the following model:

$$Y_i = I + \beta _{\it{1}}G_j + \beta _{\it{2}}P + {\boldsymbol{\beta}} _{\bf{\it{3}}}{\boldsymbol{(P \ast Gj)}} + \beta _{\it{4}}A_{ij} + \beta _{\it{5}}S_{ij} + U_{ij} + \varepsilon _{ij}$$

where in each eQTL_ij_ pair, *Y*_*i*_ is the eQTL expression value for gene *i*, *G*_*j*_ is the genotype dosage for cis SNP j, P is the proxy gene, ***P*** * ***G***_***j***_ is the interaction term representing the effect of genotype in a particular cell type, *Aij* and *S*_*ij*_ are covariates for age and sex, respectively, *U*_*ij*_ is the random effect for family structure, and *β*s are regression coefficients. Models with significant interaction terms indicate cell-type-specific eQTLs.

The following model was used to evaluate cell-type-specific expression in the brain in ROSMAP:

$$Y_i = I + \beta _{\it{1}}G_j + \beta _{\it{2}}P + {\boldsymbol{\beta}} _{\bf{\it{3}}}{\boldsymbol{(P \ast Gj)}} + \beta _{\it{4}}A_{ij} + \beta _{\it{5}}S_{ij} + \beta _{\it{6}}PM_{ij} + \beta _{{\it{7}}ij}{\it{S2}} + \beta _{{\it{8}}ij}{\it{SV1}} + \varepsilon _{ij}$$

where in each eQTL_ij_ pair, variables *Y*_*i*_, *G*_*j*_, P, *Aij, S*_*ij*_, *P*_*ij*_, *ɛ*_*ij*_, and *β*s are as described above, and *PM*_*ij*_, *S2*_*ij*_, and *SV1*_*ij*_ are covariates for PMI, study, and SV1, respectively.

A Bonferroni correction was applied to determine the significance threshold for each analysis (Supplementary Table S3).

We assessed the relevance of the significant findings more directly to AD in two ways. In one approach, AD status was included as a covariate in the eQTL and ct-eQTL analysis models. In addition, the significant eQTLs and ct-eQTLs were evaluated separately in AD cases and controls separately in the ROSMAP brain expression dataset, but not in the FHS blood expression dataset due to the paucity of AD cases (2%) in that sample.

### Selection of eQTLs in AD loci and gene-set pathway enrichment analysis

AD loci were determined based on the review of published GWAS and linkage studies of AD and AD-related traits, and this list was augmented with genes that are well recognized as functionally related to AD by experimental approaches (Supplementary Table S4). AD genes identified by GWAS met genome or study-wide significance thresholds and some of these were annotated as the closest gene to an intergenic association signal. eGenes (genes whose expression levels are associated with variation at a particular eSNP) included 88 genes and 80 eSNPs (no SNPs that significantly influence gene expression) which include genome-wide significant “peak” SNPs (i.e., top-ranked SNP within an association signal) for AD. Gene-set enrichment analysis was performed using the PANTHER (Protein ANalysis THrough Evolutionary Relationships) software tool^22^ to determine if the unique genes in the significant eQTL/ct-eQTL pairs shared by both brain and blood datasets are associated with a specific biological process or molecular function. The significance of the pathways was determined by the Fisher’s Exact test with false discovery rate (FDR) multiple test correction.

### Colocalization analyses

Assessment of causal variants shared by adjacent GWAS and eQTL signals was performed using a Bayesian colocalization approach implemented in the R package *coloc*^23^. This analysis incorporated SNP summary statistics from a recent large AD GWAS^24^ and eQTL analyses described above. For the purpose of this study, a peak SNP refers to the most significantly associated AD-SNPs under a particular GWAS signal and a lead eQTL variant is defined as the eSNP showing the strongest association with gene expression. Following recommended guidelines, the variants were deemed to be colocalized by a high posterior probability that a single shared variant is responsible for both signals (PP4 > 0.8)^23,25^. A lower threshold for statistical significance with a false discovery rate (FDR) < 0.05 for eQTL significant results was applied to maximize detection of colocalized pairs. Regional plots were constructed with LocusZoom^26^.

### Differential expression analysis of potential AD biomarker genes

The 386 distinct eGenes in shared eQTL pairs in significant blood and brain results were further examined for differentially expressed genes (DEG) between AD cases and controls in the AD enriched ROSMAP RNA-Seq dataset. After filtering, 308 of the total 386 genes were tested in the DEG analysis. The differences in expression among the groups were computed using the log2 transformation of the fold-change (log2FC). The differential analysis was performed using a linear model to identify DE genes between AD cases and controls implemented in R package limma (Linear Model for Microarray Data) version 3.32.7 (http://www.R-project.org/). The *P* values were adjusted for multiple testing to control the False Discovery Rate (FDR), with the gene considered DE when the adjusted *P* value was ≤0.05.

This study was approved by the Boston University Institutional Review Board.

## Results

**Fig. 1:**
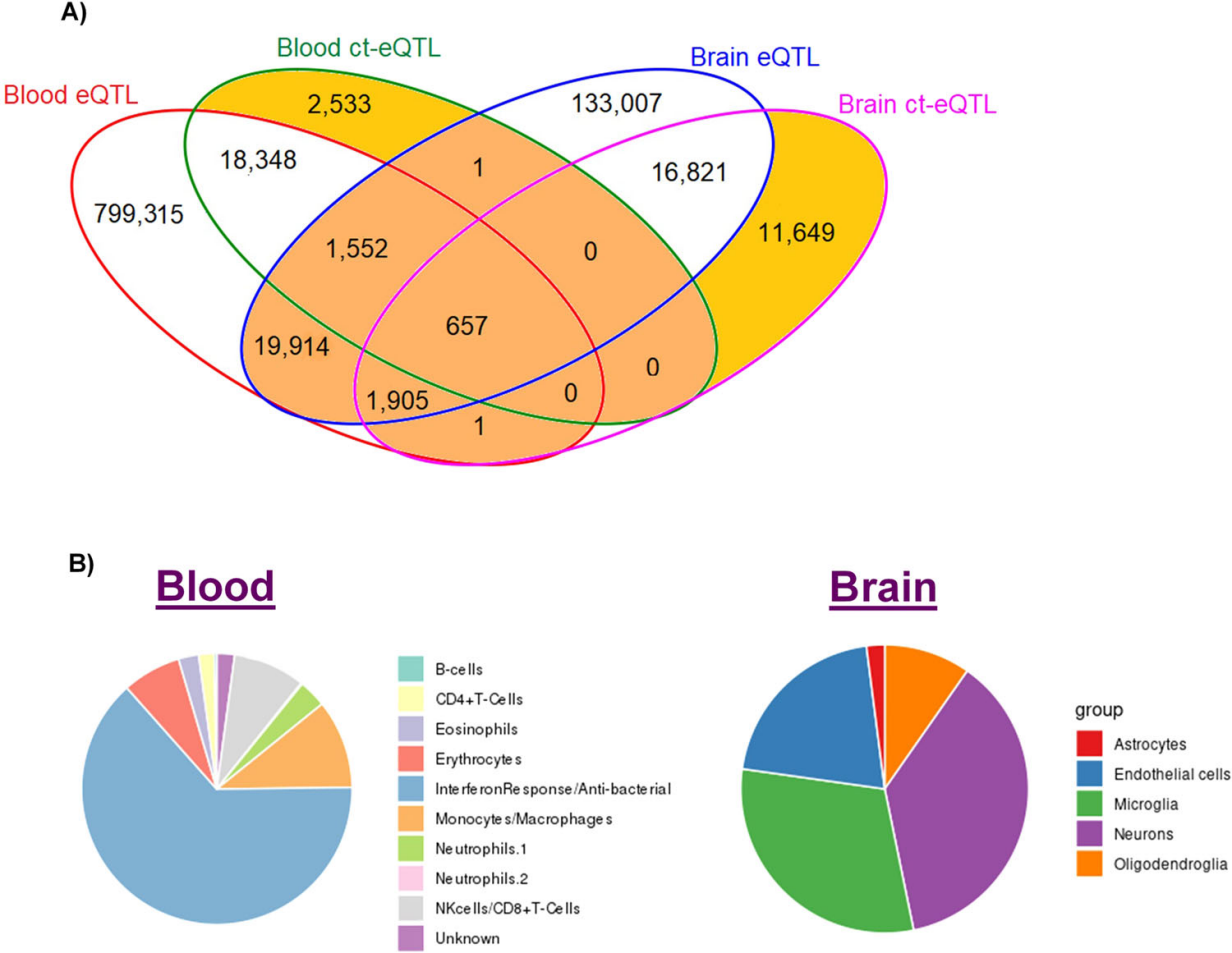
Significant gene-SNP eQTLs and ct-eQTLs in blood and brain tissue genome-wide. **A** Venn diagram shows the number of overlapping eQTLs and ct-eQTLs in blood and brain. Gold color indicates significant eQTLs that are cell-type-specific. Orange color indicates significant eQTLs that are shared between blood and brain. **B** Cell-type distributions of significant genome-wide ct-eQTL results in blood and brain.

A total of 173,857 eQTLs and 51,098 ct-eQTLs in the brain, and 847,429 eQTLs and 30,405 ct-eQTLs in blood were significant after Bonferroni correction (Supplementary Table S3 and Supplemental Resources). Additional significant gene-SNP eQTLs pairs in the brain (*n* = 11,649) and blood (*n* = 2533) were observed in ct-eQTL analysis that were not detected in eQTL analysis (Fig. 1A).

### eQTLs and ct-eQTLs common to blood and brain

Of note, 24,028 significant gene-SNP eQTL pairs were shared between blood and brain. The 386 distinct eGenes among these shared eQTL pairs (Supplementary Table S5) are most enriched in the apoptosis signaling (*P* = 0.023) and Wnt signaling (*P* = 0.036) pathways (Supplementary Table S6). Five of these eGenes (*HLA-DRB5*, *HLA-DRB1*, *ECHDC3*, *CR1*, and *WWOX*) were previously associated with AD^24,27^. Three eSNPs in eQTLs involving *HLA-DRB1*/*HLA-DRB5* (rs9271058) and *ARL17A*/*LRRC37A2* (rs2732703 and rs113986870, which are near *KANSL1* and *MAPT*) were previously associated with AD risk at the genome-wide significance level^24,28^ (Table 1).

**Table 1:** eQTLs and ct-eQTLs in established AD loci appearing in both blood and brain.

(A) eQTLs and ct-eQTLS in established AD genes in both blood and brain.										
eGene	Tissue	Cell type	Lead eSNP	Position	MAF	Beta	Std error	*P* value	Number of total significant eSNPs in gene/cell-type	AD GWAS peaks
CR1	Blood	NA	rs7533408	1:207673631	0.25	0.059	0.006	3.60E-22	169	NA
HLA-DRB5	Blood	NA	rs9269008	6:32436217	0.17	−2.580	0.057	<1.0E-314	72	NA
HLA-DRB1	Blood	NA	rs9271058	6:32575406	0.14	−2.950	0.028	<1.0E-314	630	Lead eSNP
ECHDC3	Blood	NA	rs11257290	10:11780324	0.28	0.041	0.005	2.91E-19	115	NA
WWOX	Blood	NA	rs7202722	16:78282458	0.40	0.023	0.003	2.60E-14	45	NA
**HLA-DRB5**	**Blood**	**Interferon response(+)/antibacterial (−)**	**rs9269047**	**6:32438783**	**0.12**	**−7.120**	**0.335**	**3.04E-100**	**9 [all (−)]**	**NA**
**HLA-DRB5**	**Blood**	**Monocytes/macrophages**	**rs9269047**	**6:32438783**	**0.12**	**−11.600**	**1.030**	**2.02E-29**	**1**	**NA**
**HLA-DRB5**	**Blood**	**NK cells/CD8** **+** **T cells**	**rs9269047**	**6:32438783**	**0.12**	**−7.660**	**0.994**	**1.30E-14**	**1**	**NA**
**HLA-DRB1**	**Blood**	**NK cells/CD8** **+** **T cells**	**rs9270928**	**6:32572461**	**0.15**	**−4.070**	**0.377**	**3.60E-27**	**287**	**rs9271058**
**HLA-DRB1**	**Blood**	**Eosinophils**	**rs9270994**	**6:32574250**	**0.14**	**−2.700**	**0.415**	**7.72E-11**	**42**	**NA**
**HLA-DRB1**	**Blood**	**Interferon response (+)/antibacterial (−)**	**rs9271147**	**6:32577385**	**0.14**	**−5.510**	**0.250**	**1.19E-107**	**346 [260 (−)/86 (+)]**	**rs9271058**
**HLA-DRB1**	**Blood**	**Monocytes/macrophages**	**rs9271148**	**6:32577442**	**0.13**	**−6.110**	**0.709**	**6.83E-18**	**222**	**rs9271058**
CR1	Brain	NA	rs12037841	1:207684192	0.17	−0.096	0.007	9.25E-44	64	rs6656401
HLA-DRB5	Brain	NA	rs3117116	6:32367017	0.12	−2.780	0.070	<1.0E-314	10537	rs9271058, rs9271192
HLA-DRB1	Brain	NA	rs73399473	6:32538959	0.26	−2.050	0.058	8.78E-272	10792	rs9271058, rs9271192
ECHDC3	Brain	NA	rs866770710	10:11784320	0.0002	−0.252	0.018	4.61E-44	45	NA
WWOX	Brain	NA	rs12933282	16:78124987	0.45	−0.133	0.017	1.13E-15	75	NA
**HLA-DRB5**	**Brain**	**Microglia**	**rs67987819**	**6:32497655**	**0.14**	**−1.900**	**0.137**	**9.82E-44**	**754**	**NA**
**HLA-DRB5**	**Brain**	**Endothelial cells**	**rs67987819**	**6:32497655**	**0.14**	**−2.410**	**0.220**	**6.32E-28**	**343**	**NA**
**HLA-DRB1**	**Brain**	**Microglia**	**rs72847627**	**6:32538512**	**0.28**	**−2.130**	**0.125**	**4.15E-65**	**2305**	**rs9271058, rs9271192**
**HLA-DRB1**	**Brain**	**Neurons**	**rs115480576**	**6:32538570**	**0.26**	**−2.210**	**0.153**	**2.72E-47**	**3263**	**rs9271058, rs9271192**
**HLA-DRB1**	**Brain**	**Endothelial cells**	**rs9269492**	**6:32542924**	**0.30**	**−2.250**	**0.243**	**2.06E-20**	**351**	**rs9271192**
**HLA-DRB5**	**Brain**	**Neurons**	**rs9270035**	**6:32553446**	**0.14**	**−2.520**	**0.137**	**1.46E-75**	**2540**	**rs9271058, rs9271192**
**ECHDC3**	**Brain**	**Neurons**	**rs866770710**	**10:11784320**	**0.0002**	**0.328**	**0.045**	**3.13E-13**	**2**	**NA**

(B) eQTLs and ct-eQTLs involving AD GWAS association peak SNPs in both brain and blood.								
eGene	Tissue	Cell type	eSNP + GWAS SNP	Position^a^	MAF	Beta	Std error	*P* value
HLA-DRB1	Blood	NA	rs9271058	6:32575406	0.27	−2.950	0.028	<1.0E-314
ARL17A	Blood	NA	rs2732703	17:44353222	0.21	0.147	0.023	5.95E-11
ARL17A	Blood	NA	rs113986870	17:44355683	0.09	0.166	0.025	2.30E-11
**HLA-DRB1**	**Blood**	**Interferon response (+)/antibacterial (−)**	**rs9271058**	**6:32575406**	**0.27**	**−3.010**	**0.159**	**6.36E-80**
**HLA-DRB1**	**Blood**	**NK cells/CD8** **+** **T cells**	**rs9271058**	**6:32575406**	**0.27**	**−4.090**	**0.464**	**1.20E-18**
**HLA-DRB1**	**Blood**	**Monocytes/macrophages**	**rs9271058**	**6:32575406**	**0.27**	**−3.540**	**0.497**	**1.06E-12**
HLA-DRB1	Brain	NA	rs9271058	6:32575406	0.27	−1.690	0.054	1.94E-213
HLA-DRB5	Brain	NA	rs9271058	6:32575406	0.27	−1.770	0.081	2.28E-106
LRRC37A2	Brain	NA	rs2732703	17:44353222	0.21	1.370	0.053	4.13E-150
LRRC37A2	Brain	NA	rs113986870	17:44355683	0.09	1.260	0.068	1.98E-76
ARL17A	Brain	NA	rs113986870	17:44355683	0.09	−0.326	0.047	4.96E-12
**HLA-DRB1**	**Brain**	**Microglia**	**rs9271058**	**6:32575406**	**0.27**	**−1.400**	**0.111**	**1.80E-36**
**HLA-DRB1**	**Brain**	**Neurons**	**rs9271058**	**6:32575406**	**0.27**	**−1.650**	**0.135**	**2.37E-34**
**HLA-DRB5**	**Brain**	**Neurons**	**rs9271058**	**6:32575406**	**0.27**	**−1.550**	**0.201**	**1.24E-14**
**LRRC37A2**	**Brain**	**Neurons**	**rs2732703**	**17:44353222**	**0.21**	**1.520**	**0.140**	**1.84E-27**
**LRRC37A2**	**Brain**	**Microglia**	**rs2732703**	**17:44353222**	**0.21**	**1.480**	**0.147**	**7.65E-24**
**LRRC37A2**	**Brain**	**Endothelial cells**	**rs2732703**	**17:44353222**	**0.21**	**1.750**	**0.233**	**5.88E-14**
**LRRC37A2**	**Brain**	**Microglia**	**rs113986870**	**17:44355683**	**0.09**	**1.530**	**0.195**	**4.29E-15**
**LRRC37A2**	**Brain**	**Neurons**	**rs113986870**	**17:44355683**	**0.09**	**1.400**	**0.184**	**2.77E-14**

^a^Position according to GRCh37 assembly.

*MAF* = minor allele frequency of variant in 1000 Genomes Combined European Population; cell-type-specific result rows are in bold.

eQTLs involving *CR1, ECHDC3,* and *WWOX* were much more significant in the brain than blood, whereas *HLA-DRB5* and *HLA-DRB1* were more significant in blood when comparing the effect sizes. *ECHDC3* was a significant eGene in blood and brain eQTLs (specifically in neurons). *HLA-DRB5* and *HLA-DRB1* were the only eGenes ascribed to significant ct-eQTLs in both blood and brain noting that of the ten distinct lead eSNPs, five are unique to each tissue (Table 1). Although the eQTLs involving these genes with the largest effect were observed in blood across multiple cell types, the total number of significant eSNP-eGene combinations was far greater in brain (particularly in microglia and neurons). The only instance in which the lead eSNP is also associated with AD risk at the GWS level was observed in the blood eQTL pair of *HLA-DRB1* with eSNP rs9271058 (Table 1). Among the AD-associated SNPs at the GWS level, rs9271058 is a significant eSNP for *HLA-DRB1* in both blood and brain cell types (the most significant association by *P* value was observed in antibacterial cells and microglia) and rs9271192 is a significant ct-eQTL for the gene in multiple brain cell types (Table 1). Both of these SNPs are also eSNPs for *HLA-DB5* in the brain in neurons only.

There were 657 gene–SNP eQTL pairs comprising 16 unique eGenes that were significant in blood and brain overall as well as in specific cell types in both blood and brain (Supplementary Table S7). None of these eGenes were observed in significant pathways enriched for AD genes, however, they included AD-associated genes *HLA-DRB1* and *HLA-DRB5*.

### eQTLs and ct-eQTLs among previously established AD loci

Slightly more than half (42/80 = 52.5%) of the established AD associations (Supplementary Table S3) are eGene targets for significant eQTLs in blood (Supplementary Table S8). By comparison, only seven established AD loci were eGene targets for significant eQTLs in the brain, among which *OARD1* was significant in endothelial cells only (Supplementary Table S8). Many GWS SNPs for AD risk are eSNPs affecting the expression of the nearest gene, which is usually recognized as the causative gene, but several GWS SNPs target other genes (Supplementary Table S9). For example, AD-associated eSNPs rs113986870 and rs2732703 in the *MAPT/KANSL1* region target *ARL17A* in blood, but are paired in seven of eight eQTLs and ct-eQTLs with *LRRC37A2* in the brain (Supplementary Table S9). *HLA-DRB1* is the only AD gene with a significant ct-eQTL in blood, whereas many AD genes have significant blood eQTLs. In the brain, only four AD loci (*CR1, HLA-DRB1/DRB5, IQCK*, and *MAPT/KANSL1*) have significant brain eQTLs of which *HLA-DRB1/DRB5* and *MAPT/KANSL1* are the only brain ct-eQTLs, noting that all are significant in microglia, neurons, and endothelial cells.

Next, we evaluated whether the most significant eSNPs and SNPs genome-wide significantly associated with AD status (i.e., AD-SNPs) co-localize and thus to identify a single shared variant responsible for both signals (posterior probability of shared signals (PP4) > 0.8). This analysis revealed eight eQTL/ct-eQTL signals that colocalized with seven AD GWAS signals and half of the colocalized signals involved a ct-eQTL (Table 2 and Supplementary Fig. S2). Two different eSNPs for *CD2AP*, rs4711880 (eQTL *P* = 1.4 × 10^−104^) and rs13201473 (NK/CD8 + T cell ct-eQTL *P* = 1.47 × 10^−9^), flank *CD2AP* GWAS SNP rs10948363 which is also the second most significant eQTL (*P* = 2.32 × 10^−104^) and the second most significant ct-eQTL in NK cells/CD8 + T cells (*P* = 2.66 × 10^−9^). These three SNPs span a 9.0-kb region in intron 2 and are in complete linkage disequilibrium (LD, *r*^2^ = 1.0), indicating that any one or more of them could affect the function of target gene CD2AP. Rs6557994 is the most significant eSNP for and located in *PTK2B* (blood interferon ct-eQTL *P* = 2.58 × 10^−9^) and is moderately correlated with the *PTK2B* GWAS SNP (rs28834970, *r*^2^ = 0.78, *P* = 1.58 × 10^−9^). Thus, it is not surprising that rs6557994 is also significantly associated with AD risk (*P* = 8.19 × 10^−7^). Rs6557994 is also correlated with a GWAS SNP in *CLU*, located approximately 150 kb from PTK2B, that is not significantly associated with the expression of any gene. Because *PTK2B* and *CLU* are independent AD risk loci^27^, it is possible that this eSNP has an effect on AD pathogenesis through independent pathways (Supplementary Fig. S2). The most significant eSNP in *MADD* (rs35233100, *P* = 2.88 × 10^−10^) was predicted to have functional consequences because it is a stop-gained mutation. This brain eQTL is colocalized (PP4 = 0.95) and weakly correlated with a GWAS SNP (*P* = 1.91 × 10^−5^) in *CELF1* rs10838725 (*r*^2^ = 0.12).

**Table 2:** Colocalized AD GWAS/lead eQTL SNP pairs.

Region^a^	AD GWAS Variant							Lead eQTL variant						eQTL type	PP4	*r*^2^
	rsID	Nearest gene	MAF	*P* value	eQTL *P* value	eGene	Cell type	rsID	MAF	eGene	eQTL *P* value	Cell type	GWAS *P* value			
6:46487762–48487762	rs10948363	CD2AP	0.72	1.77E-07	2.32E-104	CD2AP	NA	rs4711880	0.23	CD2AP	1.36E-104	NA	2.57E-07	Blood eQTL	0.909	1.00
6:46487762–48487762	rs10948363	CD2AP	0.72	1.77E-07	2.66E-09	CD2AP	NK cells/CD8 + T cells	rs13201473	0.27	CD2AP	1.47E-09	NK cells/CD8 + T cells	2.74E-07	Blood ct-eQTL	0.917	1.00
8:26195121–28195121	rs28834970	PTK2B	0.63	1.58E-09	9.15E-09	PTK2B	Interferon response/antibacterial cells	rs6557994	0.41	PTK2B	2.58E-09	Interferon response/antibacterial cells	8.19E-07	Blood ct-eQTL	0.990	0.78
8:26467686–28467686	rs9331896	CLU	0.61	3.62E-16	Not an eSNP			rs6557994	0.45	PTK2B	2.58E-09	Interferon response/antibacterial cells	8.19E-07	Blood ct-eQTL	0.990	0.00
1:206692049–208692049	rs6656401	CR1	0.19	2.17E-15	1.05E-43	CR1	NA	rs12037841	0.19	CR1	9.25E-44	NA	1.77E-15	Brain eQTL	0.993	1.00
11:46557871–48557871	rs10838725	CELF1	0.68	1.91E-05	Not an eSNP			rs35233100	0.068	MADD	2.88E-10	NA	1.25E-03	Brain eQTL	0.954	0.12
11:58923508–60923508	rs983392	MS4A6A	0.59	4.76E-15	Not an eSNP			rs11230563	0.35	CD6	2.31E-113	NA	0.48	Brain eQTL	0.854	0.00
19:44411941–46411941	rs429358	APOE	0.78	< 1.0E-300	Not an eSNP			rs74253343	0.47	RELB	1.9E-14	Oligodendroglia	0.23	Brain ct-eQTL	0.971	0.00

^a^Map position within 1 Mb of AD GWAS SNP according to GRCh37 assembly.

*MAF* minor allele frequency, *NA* not available, *PP4* posterior probability of colocalization, *r*^2^ correlation of AD and eQTL variants.

### ct-eQTLs genome-wide

Examination of the distribution of the significant ct-eQTL results genome-wide showed that nearly two-thirds of the ct-eQTLs in blood occurred in interferon response/antibacterial cells which are defined as type I interferon viral response cells in upregulated genes and type II interferon antibacterial inflammatory response cells in downregulated genes^4^, whereas brain ct-eQTLs are highly represented in endothelial cells, neurons, and microglia (Fig. 1B and Supplementary Table S10). Further examination of significant results within myeloid cell lineages (i.e., microglia and monocytes/macrophages) which account for a large proportion of the genetic risk for late-onset AD^10^ revealed that 3234 or 10.6% of all significant ct-eQTLs in blood were in monocytes/macrophages. This subset includes 128 unique eGenes which are significantly enriched in the AD amyloid secretase pathway (FDR *P* = 0.013, Supplementary Table S11). A total of 974 or 30.1% of ct-eQTLs including 4 of the 20 most significant eGenes in monocytes/macrophages are located within 1 Mb of established AD loci. One of the eGenes in this top-ranked group (*HLA-DRB5*) is an established AD gene, and three others that are near established AD loci (*DLG2* near *PICALM*^29^, *C4BPA* near *CR1*^30^, and *MYO1E* near *ADAM10* ^31^) are reasonable AD gene candidates based on evidence using non-genetic approaches (Table 3). Microglia accounted for 15,560 (30.5%) of significant ct-eQTLs in the brain (Supplementary Table S10) which involved 304 unique eGenes. Approximately 52% of significant ct-eQTLs in microglia are located in AD regions including five of the 20 most significant ct-eQTLs in this group (Table 3). One of these five eGenes is an established AD gene (*HLA-DRB1*) and two others (*ALCC*^32^ and *WNT3*^33^) have been linked to AD in previous studies.

**Table 3:** Top-ranked ct-eQTLs in myeloid cell types.

(A) Monocytes/macrophages							
eGene	Lead eSNP	Position^a^	MAF	Beta	Std error	*P* value	Number of significant eSNPs in gene/cell type
SLC12A1	rs8037626	15:48606346	0.17	−3.340	0.219	1.62E-52	126
DLG2	rs75798025	11:84018349	0.01	5.350	0.364	6.66E-49	597
ABCA9	rs4147976	17:66925923	0.44	0.872	0.068	1.97E-37	48
PTPRG	rs116497321	3:62245373	0.01	2.650	0.221	3.96E-33	10
CLNK	rs5028371	4:10452986	0.50	1.060	0.092	7.66E-31	272
NFXL1	rs10938499	4:47848377	0.33	−1.270	0.112	8.38E-30	73
FCRL5	rs12760587	1:157526021	0.23	2.140	0.19	1.99E-29	93
HLA-DRB5	rs9269047	6:32438783	0.12	−11.600	1.03	2.02E-29	1
FMOD	NA	1:203263699	NA	2.110	0.2	5.08E-26	42
ABCA6	rs144031521	17:67162715	0.01	8.620	0.833	4.27E-25	9
INPP5F	rs181735165	10:121555618	0.02	7.150	0.701	1.99E-24	11
RBMS3	rs192885607	3:29612955	0.00	2.570	0.257	1.52E-23	34
ARHGAP44	NA	17:12750576	NA	1.760	0.177	2.69E-23	54
C4BPA	rs74148971	1:207275799	0.07	−2.300	0.234	8.44E-23	24
DCLK2	rs114930380	4:150954757	0.03	1.630	0.169	5.16E-22	39
PAM	NA	5:102153433	NA	−0.691	0.073	2.00E-21	47
MYO1E	rs146483144	15:59422810	0.03	4.300	0.453	2.26E-21	17
DSP	rs4960328	6:7495948	0.42	0.554	0.061	9.30E-20	6
ROR1	rs1557596882	1:64453767	0.01	3.570	0.393	1.05E-19	31
CACNB2	rs117299889	10:18404550	0.06	1.510	0.168	2.52E-19	61

(B) Microglia							
eGene	Lead eSNP	Position^a^	MAF	Beta	Std error	*P* value	Number of significant eSNPs in gene/cell type
AC142381.1	rs199931530	16:33047273	0.45	−0.401	0.012	3.89E-233	43
MLANA	rs201480524	9:68457329	0.50	−0.176	0.007	4.82E-124	18
AC015688.3	rs62058902	17:25303954	0.50	−0.213	0.009	1.94E-113	11
HNRNPCL1	rs75627772	1:13182567	0.00	0.186	0.008	4.31E-113	8
AL050302.1	rs3875276	21:14472722	0.50	−0.890	0.040	2.62E-111	142
ALLC	rs9808287	2:3624799	0.11	0.833	0.044	1.41E-79	12
FAM21B	NA	10:47917284	NA	−2.210	0.118	2.88E-78	22
WNT3	rs9904865	17:44908263	0.37	−1.570	0.084	3.90E-78	1
RPL9	rs1458255	4:39446549	0.28	−2.370	0.137	4.76E-67	37
HLA-DRB1	rs72847627	6:32538512	0.32	−2.130	0.125	4.15E-65	2305
XRCC2	rs80034602	7:152104360	0.50	2.120	0.128	1.30E-61	5
WI2-3308P17.2	rs4067785	1:120576209	0.50	−0.184	0.011	1.33E-58	9
DEFB121	rs117541536	20:29422202	0.49	−7.420	0.460	1.56E-58	1
GINS1	rs75374582	20:26109209	0.50	−33.200	2.060	1.95E-58	5
EXOSC10	rs2580511	1:121113600	0.50	−4.390	0.276	5.78E-57	5
TRIM49B	rs202086299	11:48363026	0.50	0.205	0.013	2.16E-54	5
TMPRSS9	rs7248384	19:23936403	0.48	−1.410	0.093	1.83E-52	1
LDHC	NA	11:18432033	NA	0.428	0.030	1.07E-47	73
HLA-DOB	rs201194354	6:32796857	NA	1.190	0.084	4.39E-46	70
DEFB119	rs78099404	20:29617870	0.50	−0.377	3.51E-15	<1.0E-314	142

^a^Map position according to GRCh37 assembly.

*MAF* minor allele frequency, *NA* not available.

### Overlap of eQTLs and ct-eQTLs among myeloid cell types

**Fig. 2:**
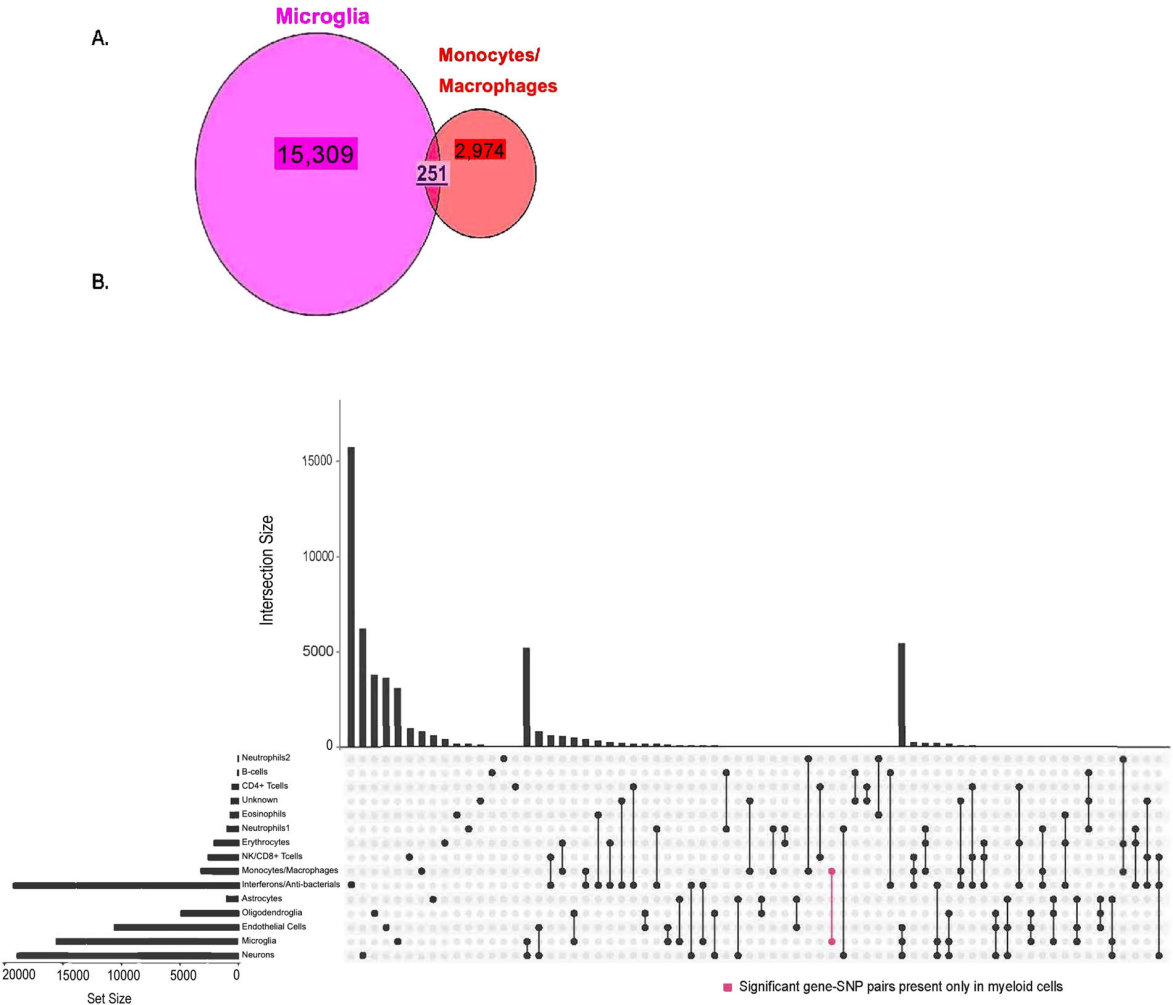
Intersection of significant gene-SNP eQTL pairs between cell types in blood and brain tissue. **A** Venn diagram showing overlap of ct-eQTL pairs in myeloid cell types (microglia and monocytes/macrophages). **B** Number of significant eQTLs unique to and that overlap cell types in blood and brain. The bar chart on the left side indicates the number of significant eQTLs involving each cell type and the bar chart above the matrix indicates the number of significant eQTLs that are unique to each cell type and set of cell types. Pink colored bar indicates the number of eQTLs pairs that are unique to microglia and monocytes/macrophages.

Considering significant eGene–eSNP pairs in myeloid cell types, 251 pairs including five distinct eGenes (*BTNL3*, *FAM118A*, *HLA-DOB*, *HLA-DRB1*, and *HLA-*DRB5) are shared between microglia and monocytes/macrophages (Table 4A and Fig. 2A). Three of these pairs involving eSNPs rs3763355, rs3763354, and rs1183595100 have the same target gene *HLA-DOB* and occur only in microglia and monocytes/macrophages (Table 4B). Among the significant ct-eQTLs in the brain, the cell types with the largest proportion that were also significant in monocytes/macrophages were microglia (1.6%) and neurons (1.3%) (Table 4). Conversely, among the significant ct-eQTLs in blood, the cell types with the largest proportion that were also significant in microglia were NK/CD + T cells (12.9%) and monocytes/macrophages (7.8%). Among ct-eQTLs which are significant only for one cell-type each in blood and one in the brain, monocytes/macrophages shared three ct-eQTLs with microglia but with no other brain cell types (Fig. 2B and Table 4C). By comparison, microglia shared 63 ct-eQTLs with interferons/antibacterial cells, but with no other blood cell types. The proportions of overlap of ct-eQTLs between blood and brain across ten paired cell types are significantly different (Fisher’s Exact test $\chi _9^2$ = 789.8, *P* = 2.2 × 10^−16^). The much larger number of ct-eQTLs in microglia that were common with interferons/bacterial cells than monocytes/macrophages may reflect the substantially greater proportion of significant eQTLs in blood involving interferons/antibacterial cells (64%) than monocytes/macrophages (10.6%) (Supplementary Table S10). The only other ct-eQTLs that were unique to a pair of cell types in brain and blood cell type involved neurons paired with neutrophils (*n* = 3) and with interferons/antibacterial cells (*n* = 65) (Fig. 2B).

**Table 4:** Overlap of ct-eQTLs in myeloid cell types in brain and blood.

(A) Unique eGenes shared in significantly associated ct-eQTLs in monocytes/macrophages and microglia. Number below each gene represents significant eGene-eSNP eQTL pairs in each gene.				
BTNL3	FAM118A	HLA-DOB	HLA-DRB1	HLA-DRB5
1	43	6	200	1

(B) eSNP-eGene pairs among ct-eQTLs significant in both monocytes/macrophages and microglia.								
eGene	eSNP	Position	MAF	Monocytes/macrophages		Microglia		AD GWAS *P* value^23^
				Beta	*P* value	Beta	*P* value	
HLA-DOB	rs3763355	6:32786882	0.06	−2.02	9.98E-15	0.938	3.89E-14	0.001
HLA-DOB	rs3763354	6:32786917	0.15	−1.11	1.40E-10	−0.642	2.80E-13	0.652
HLA-DOB	rs1183595100	6:32768232	NA	−1.13	8.34E-11	−0.605	1.98E-11	NA

(C) Overlap of significant eQTLs in brain and blood with ct-eQTLs in myeloid cell types.				
Cell types	Monocytes/macrophages		Microglia	
Blood	# ct-eQTLs common to cell-type pair	# ct-eQTLs unique to cell-type pair	# ct-eQTLs common to cell-type pair	# ct-eQTLs unique to cell-type pair
Neutrophils1			3 (0.3%)^a^	0
CD4 + T cells			3 (0.5%)	0
NK/CD8 + T cells			337 (12.9%)	0
Erythrocytes			119 (5.6%)	0
Monocytes/macrophages			251 (7.8%)	3
Unknown			0	0
Interferon/antibacterial			628 (3.3%)	63
Neutrophils2			0	0
B cells			0	0
Eosinophils			38 (5.2%)	0
*Brain*				
Endothelial cells	55 (0.5%)	0		
Neurons	250 (1.3%)	0		
Microglia	251 (1.6%)	3		
Astrocytes	0	0		
Oligodendroglia	0	0		

^a^Number in parentheses represent the proportion of ct-eQTLs for each cell type on the left that were also observed in either microglia or monocytes/macrophages.

### Effect of AD status on significant eQTLs and ct-eQTLs

None of the significant eQTLs and ct-eQTLs observed in the brain (Table 1) were influenced by the inclusion of AD status in the analysis models. Stratified analyses revealed that the top findings involving eSNPs that were previously associated with AD at the genome-wide significant level were evident in both AD cases and controls (Supplementary Table 12A). Although most of the findings were more significant in AD cases than controls (noting that the ROSMAP brain sample of AD cases was 44% larger than the control sample), the effect size for most eSNP–eGene pairs was similar. However, patterns among AD cases and controls differed when focusing on the most significant eQTLs and ct-eQTLs in established AD genes. For example, eQTLs observed in undifferentiated brain cells involving *CR1* paired with rs6656401 (*P* = 7.85 × 10^−22^), in endothelial cells involving *HLA-DRB1* paired with rs73399473 (*P* = 2.5 × 10^−10^) and *HLA-DRB5* paired with rs1064697 (P = 2.18 × 10^−14^), in microglia involving *HLA-DRB1* paired with rs72847627 (*P* = 4.43 × 10^−51^), and in neurons involving *ECHDC3* paired with rs866770710 (*P* = 5.79 × 10^−13^) were significant only in AD cases (Supplementary Table 12B). Other eQTLs observed in multiple cell types involving these same genes (*HLA-DRB1*: rs111976080, *P* = 1.68 × 10^−25^; *HLA-DRB5*: rs2395517, *P* = 8.64 × 10^−12^, rs9271184, *P* = 5.42 × 10^−41^, and rs80141235, *P* = 3.94 × 10^−9^) were significant only in controls. Several other eQTLs and ct-eQTLs in *CR1, HLA-DRB1*, and *HLA-DRB5* were highly significant in one group but showing a much less significant effect in the opposite direction in the other group.

Among the 386 eGenes that were significant in both blood and brain (Supplementary Table S5), 87 were differentially expressed between AD cases and controls (Supplementary Table S13). This includes *WWOX* (*P*_adj_ = 1.02 × 10^−4^) and *LRRC2* (*P*_adj_ = 2.38 × 10^−3^) which have been associated with AD risk by GWAS^24,34^.

## Discussion

We identified several novel AD-related eQTLs that highlight the importance of cell-type-dependent context. It is noteworthy that there were more significant ct-eQTLs in the brain (*n* = 51,098) than blood (*n* = 30,405) even though the dataset containing expression data from blood (FHS) is several times larger than the brain expression dataset (ROSMAP). This could be due to greater cell-type heterogeneity in the brain, the enrichment of AD cases in the ROSMAP dataset who may show different patterns of gene expression compared to persons without AD, or highly variable gene expression across cell types in the nervous system^35^. Because expression studies in the brain are often constrained by the small number of specimens compared to studies in other tissues, postmortem changes that may affect gene expression in the brain^36^, and the growing recognition that AD is a systemic disease^37–39^, incorporating expression data from multiple tissues can enhance discovery of additional genetic influences on AD risk and pathogenesis.

Although most significant findings were tissue-specific, the 386 distinct eGenes among more than 24,000 significant gene-SNP eQTL pairs that were shared between blood and brain were enriched in the apoptosis signaling pathway which has been suggested to contribute to the underlying pathology associated with AD^40,41^. Six established AD genes (*CR1*, *ECHDC3*, *HLA-DRB1*, *HLA-DRB5*, *LRRC2*, and *WWOX*^24,27,34^) were shared eGenes in the brain and blood. They were also involved in eQTLs and ct-eQTLs that showed different patterns of association in cases versus controls (i.e., *CR1, HLA-DRB1, HLA-DRB5*, and *ECHD3*) or differentially expressed in AD cases versus controls (i.e., *WWOX* and *LRRC2*).

The complement receptor 1 (*CR1*) gene encodes a transmembrane glycoprotein functioning in the innate immune system by promoting phagocytosis of immune complexes, cellular debris, and Aβ^42^. *CR1* is an eGene for several eSNPs, including AD GWAS peak SNP rs6656401 located within the gene, in brain and blood eQTLs and the effects on *CR1* expression are opposite in blood and brain. There are multiple possible explanations for the effect direction differences across tissues. The effect of eSNP rs6656401 on *CR1* expression may be developmental, noting that the average age of the FHS subjects (a group with expression data in blood) is more than 30 years younger than the ROSMAP subjects (group with expression data in the brain). The difference between brain and blood may also reflect postmortem changes in the brain that are not indicative of expression in vivo. Alternatively, these effects may be related to AD because few FHS subjects were AD cases at the time of blood draw, whereas 60% of subjects in the ROSMAP sample are AD cases. This idea is supported by the observation of a larger and positive effect of rs6656401 on *CR1* expression in AD (*β* = 0.020) compared to control brains (*β* = −0.0086). Opposite effect directions of expression in brain and blood from AD patients compared to controls have been observed for several ribosomal genes^43^. GWS variants located in the region spanning *ECHDC3* and *USP6NL* have previously been associated with AD^44^. Altered *ECHDC3* expression in AD brains^45^ supports the idea that this gene has a role in AD. Knockout of *WWOX* in mice leads to aggregation of amyloid-β (Aβ) and Tau, and subsequent cell death^46,47^. *LRRC2* is located in a region including GWS variants that modify the inverse relationship between education attainment and AD^34^. A recent study showed that the expression of a *LRRC2* long noncoding RNA (*LCCR2-AS1*) is significantly increased in children with autism spectrum disorder compared to children with normal development^48^.

The human leukocyte antigen (HLA) region is the key susceptibility gene in many immunological diseases and many associations have been reported between neurodegenerative diseases and HLA haplotypes^49^. In addition, the most widely used marker to examine activated microglia in normal and diseased human brains is *HLA-DR* and microglia activation increases with the progression of AD^50,51^. *HLA-DRB5* and *HLA-DRB1* have been implicated in numerous GWAS studies as significantly associated with AD risk^24,27^ and appeared frequently among significant results in blood and brain in this study. Rs9271058, which is located approximately 17.8 kb upstream of *HLA-DRB1*, is significantly associated with AD risk (*P* = 5.1 × 10^−8^)^24^. and when paired with *HLA-DRB1* is a significant eQTL and ct-eQTL in multiple cell types in blood and brain including myeloid lineage cells (i.e., monocytes/macrophages and microglia). This eSNP is also a significant eQTL in the brain and specifically in neurons when paired with *HLA-DRB5*. Rs9271192, which is adjacent to rs9271058 and also significantly associated with AD risk (*P* = 2.9 × 10^−12^)^27^, is a significant eQTL and ct-QTL with multiple cell types in brain but not blood when paired with *HLA-DRB5 and HLA-DRB1*.

Significant associations for AD have been reported with variants spanning a large portion of the major histocompatibility (MHC) region in HLA class I, II, and III loci^49,52,53^. While the strongest statistical evidence for association in this region is with variants in *HLA-DRB1*^24^, fine mapping in this region suggests that a class I haplotype (spanning the *HLA-A* and *HLA-B* loci) and a class II haplotype (including variants in *HLA-DRB1, HLA-DQA1*, and *HLA-DQB1*) are more precise markers of AD risk. Given the complexity of the MHC region and extensive LD, further work is needed to confirm whether this is a true eQTL or a signal generated from a specific HLA allele or haplotype. Although functional studies may be required to discern which HLA variants have AD relevant consequences and HLA polymorphisms methods would be required to detect differential gene expression between the HLA alleles, our findings support a role for the immune system in AD^37,54^ and the hypothesis that a large proportion of AD risk can be explained by genes expressed in myeloid cells^10^.

The potential importance and relevance to AD of eQTLs and ct-eQTLs in myeloid cell types are supported by the observation that a large portion of GWS ct-eQTLs we identified map within 1 Mb of established AD loci, and 58% (12/20 in monocytes/macrophages and 11/20 in microglia) of the most significant eGenes have been previously implicated in AD. *DLG2* encodes a synaptic protein whose expression was previously reported as downregulated in an AD proteome and transcriptome network^55^ and inversely associated with AD Braak stage^29^. Genome-wide significant associations of AD risk with *PTPRG* was observed in a family-based GWAS^56^ and with *CLNK* in a recent large GWAS for which the evidence was derived almost entirely with a proxy AD phenotype in the UK Biobank^57^. *NFXL1* is a novel putative substrate for *BACE1*, an important AD therapeutic target^58^. *FCRL5* may interact with the APOE*E2 allele and also modifies AD age of onset^59^. *C4BPA* was shown to be consistently downregulated in MCI and AD patients, and the protein encoded by this gene accumulates in Aβ plaques in AD brains^30,60^. Lower levels of the *PAM* have been observed in the brains and CSF of AD patients compared to healthy controls^61^ and *MYO1E* is expressed by anti-inflammatory disease-associated microglia^31^. As a calcium channel protein, *CACNB2* may affect AD risk by altering calcium levels which could cause mitochondrial damage and then induce apoptosis^62,63^.

Likewise, several eGenes of top-ranked ct-eQTLs in microglia that are not established AD loci may have a role in the disease. It was shown that copy number variants (CNVs) near *HNRNPCL1* overlapped the coding portion of the gene in AD cases but not controls^64^. A region of epigenetic variation in *ALLC* was associated with AD neuropathology^32^. *FAM21B*, a retromer gene in the endosome-to-Golgi retrieval pathway, was associated with AD in a candidate gene study^65^. Vacuolar sorting proteins genes in this pathway including *SORL1* have been functionally linked to AD through the trafficking of Aβ^66^. One study demonstrated that *WNT3* expression in the hippocampus was increased by exercise and alleviated AD-associated memory loss by increasing neurogenesis^33^. Expression of *RPL9* is downregulated in severe AD^67^ and significantly differs by sex among persons with the *APOE* ɛ4 allele^68^. Significant evidence of association with a *TRIM49B* SNP was found in a genome-wide pleiotropy GWAS of AD and major depressive disorder (MDD)^69^.

*HLA-DOB*, which is one of the five distinct eGenes (*BTNL3*, *FAM118A*, *HLA-DOB*, *HLA-DRB1*, and *HLA-DRB5)* for significant ct-eQTLs shared between microglia and monocytes/macrophages, and is the target gene for three eSNPs (rs3763355, rs3763354, and rs1183595100) that were evident only in these myeloid cell types. These eSNPs have similar eQTL p-values in both cell types, but have slightly larger effect sizes in monocytes (Fig. 2). The effect of rs3763355 on expression is in opposite directions in monocytes and microglia which suggests *HLA-DOB* may be acting in different immune capacitates in AD in blood and brain. Though the functions of the genes *BTNL3* and *FAM118A* are unknown, a *BTNL8-BTNL3* deletion has been correlated with TNF and ERK1/AKT pathways, which have an important role in immune regulation inducing inflammation, apoptosis, and proliferation, suggesting the deletion could be correlated to inflammatory disease^70^. This suggests that the majority of the shared myeloid cell-type genes—the *HLA* genes and possibly *BTNL3*—are all immune-related. Ct-eQTLs involving microglia and monocytes/macrophages had a larger proportion of total intersection, an isolated set interaction and a statistically significant overlap (*P* < 1.0 × 10^−314^), demonstrating a stronger connection than other brain/blood cell types in this study and thus providing further evidence for the importance of the immune system in AD.

The proportions of significant ct-eQTLs in NK cells/CD8 + T cells, monocytes/macrophages, and eosinophils are comparable to those observed in reference blood tissue (https://www.miltenyibiotec.com/US-en/resources/macs-handbook/human-cells-and-organs/human-cell-sources/blood-human.html#gref), (https://www.stemcell.com/media/files/wallchart/WA10006-Frequencies_Cell_Types_Human_Peripheral_Blood.pdf). Similarly, significant eQTL distributions in endothelial cells, neurons, and glia are consistent with reference brain tissue^71^. The majority of significant blood eQTLs were type I interferon response cells which cross-regulate with pro-inflammatory cytokines that drive the pathogenesis of autoimmune diseases including AD and certain heart diseases^72–74^ and the enrichment of interferon ct-eQTLs in this study could possibly be due to the high proportion of subjects these diseases in the FHS dataset. In contrast, the proportion of significant ct-eQTLs among glial cells is much lower in astrocytes and oligodendrocytes and three-fold higher in microglia than in reference brain tissue (https://www.stemcell.com/media/files/wallchart/WA10006-Frequencies_Cell_Types_Human_Peripheral_Blood.pdf). Because many AD risk genes are expressed in myeloid cells including microglia^10^, the large number of microglia ct-eQTLs is consistent with the high proportion of AD subjects in the ROSMAP dataset.

Several SNPs previously reported to be associated with AD at the GWS level were associated with eGenes that differ from genes ascribed to AD loci and thus may have a role in AD. Karch et al. observed that the expression of *PILRB*, which is involved in immune response and is the activator receptor to its inhibitory counterpart *PILRA*, an established AD gene^75,76^, was highest in microglia^11^. *CNN2*, the eGene for eSNP rs4147929 located near the end of *ABCA7*, significantly alters extracellular Aβ levels in human induced pluripotent stem cell-derived neurons and astrocytes^77^. Rs4147929 also targeted *HMHA1* which plays several roles in the immune system in an HLA-dependent manner^78^. The eSNP/GWAS SNP rs3740688 located in *SPI1* also affects expression of *MYBPC3* and *MADD*. *MYBPC3* was recently identified as a target gene for eSNPs located in *CELF1* and *MS64A6A* in a study of eQTLs in blood for GWS AD loci^79^. *MADD* is expressed in neurons^11^, is involved in neuronal cell death in the hippocampus^80^, and was shown to be a tau toxicity modulator^81^. Although eSNP rs113986870 in *KANSL1* when paired with the nearby eGene *LRRC37A2* was a significant brain eQTL and ct-eQTL, *LRRC37A2* encodes a leucine-rich repeat protein that is expressed primarily in testis and has no apparent connection to AD. However, rs113986870 also significantly influenced the expression of another gene in this region, *ARL17A*, that was previously linked to progressive supranuclear palsy by analysis of gene expression and methylation^82^.

The aim of this study was to identify context-dependent (i.e., cell-type-specific) eQTLs in blood and brain among older individuals including AD cases using a genome-wide approach. Previous studies have evaluated ct-eQTLs using purified cells, but they focused on only one or two cell types^7,10^. Other studies examined multiple cell types but using expression data generated from individuals who were on average much younger than the FHS and ROSMAP participants^4,83,84^. With the exception of a recent report by Patrick et al. who applied a deconvolution approach to estimate cell-type proportions from in cortical tissue obtained from ROSMAP participants but did not examine ct-eQTLs^85^, our study is one of the first to study eQTLs and ct-eQTLs in a sample enriched for AD cases and link these findings to established AD genes and AD risk.

Our study has several noteworthy limitations. The proxy genes individually or collectively may not accurately depict cell-type-specific context. In addition, the comparisons of gene expression in blood and brain may yield false results because they are based on independent groups ascertained from a community-based longitudinal study of health (FHS—blood) and multiple sources for studies of AD (ROSMAP—brain) which were not matched for age, sex, ethnicity and other factors which may affect gene expression. Moreover, the FHS cohort contains many elderly but relatively few AD cases, whereas ~60% of the ROSMAP participants in this autopsy sample are AD cases. Although the dataset for eQTL analysis in blood was much larger than the dataset derived from the brain, the effect sizes associated with many of the eQTLs common to both tissues were similar. Also, findings in the brain may reflect postmortem changes unrelated to disease or cell-type different expression^36^. Another limitation of our findings is that some cell types are vastly underrepresented compared to others. Because myeloid cell types are represented in only a small proportion of the total cell populations in the brain and blood (generally ~10%), it is difficult to identify myeloid-specific signals^12^. Despite this limitation, many of the most significant and noteworthy results of this study involved monocytes/macrophages and microglia.

## Conclusion

Our observation of cell-type-specific expression patterns for established and potentially novel AD genes, finding of additional evidence for the role of myeloid cells in AD risk, and discovery of potential novel blood and brain AD biomarkers highlight the importance of cell-type-specific analysis. Future studies that use more robust computational approaches such as deconvolution to reliably estimate cell type proportions^83–85^, compare cell-type-specific differential gene expression among AD cases and controls using single-cell RNA-sequencing or cell count data and conduct functional experiments are needed to validate and extend our findings.

## Supplementary information

Supplementary Tables and Figures

Supplemental Resources
