# Supplementary material for: Cell-type-specific expression quantitative trait loci associated with Alzheimer disease in blood and brain tissue
Source: Transl Psychiatry. 2021 Apr 27;11:250. doi: 10.1038/s41398-021-01373-z (PMC8079392; doi:10.1038/s41398-021-01373-z)
Supplement: Supplementary file 2 — Supplemental Resources [file 41398_2021_1373_MOESM2_ESM.pdf]

Supplemental Resources. Top-ranked eSNPs per eGene for significant eQTLs and ct-eQTLs in blood and brain.

FHS (blood) eQTLs					FHS (blood) ct-eQTLs		
Gene	Top eSNP Position	Beta	Std Error	P-value	Gene	Top eSNP Position	Cell-type
A2M	12:9172332	0.33	0.0088	1.95E-272	AAK1	2:69672414	Erythrocytes
A4GALT	22:43115776	-0.0747	0.00741	1.14E-23	AAK1	2:69672414	Monocytes / Macrophages
AACS	12:125593318	-0.0227	0.00289	5.15E-15	ABCA6	17:67162715	Erythrocytes
AAGAB	15:67490486	0.0322	0.00471	9.22E-12	ABCA6	17:67162715	Monocytes / Macrophages
AAMDC	11:77580548	0.161	0.00517	1.65E-196	ABCA6	17:67162715	Neutrophils 1
AASDH	4:57276739	0.114	0.00679	5.10E-62	ABCA9	17:67062977	CD4+ T-Cells
AASS	7:121784441	0.165	0.00693	1.63E-118	ABCA9	17:66925923	Erythrocytes
ABCA10	17:67289504	-0.0364	0.00434	6.29E-17	ABCA9	17:66925923	Monocytes / Macrophages
ABCA11P	4:458667	0.0753	0.00746	9.30E-24	ABCA9	17:66925923	Neutrophils 1
ABCA2	9:139927062	0.0418	0.00373	6.54E-29	ABLIM1	10:116342377	Monocytes / Macrophages
ABCA5	17:67289504	-0.0655	0.00533	2.82E-34	ACCS	11:44087989	Interferon response/Anti-bacterial
ABCA7	19:1100976	0.0173	0.00236	2.88E-13	ACCS	11:44087989	NK cells / CD8+ T-Cells
ABCB4	7:87078547	0.0609	0.00697	3.15E-18	ACTA2	10:90754607	Interferon response/Anti-bacterial
ABCB5	7:20735885	-0.036	0.00557	1.11E-10	ADAMTS6	5:64796549	Monocytes / Macrophages
ABCC1	16:16043174	0.0595	0.00534	1.45E-28	ADAMTS6	5:64796549	Neutrophils 1
ABCC13	21:15653302	0.0621	0.0069	3.29E-19	ADK	10:76248814	Interferon response/Anti-bacterial
ABCC3	17:48712087	-0.0752	0.00326	2.87E-112	ADK	10:76248814	Monocytes / Macrophages
ABCC4	13:95986038	0.207	0.0129	1.99E-56	ADK	10:75928933	Unknown
ABCC5	3:183743647	-0.134	0.00369	4.53E-258	ADPRM	17:10596713	Interferon response/Anti-bacterial
ABCD2	12:39905180	0.0494	0.00672	2.25E-13	AFAP1	4:7939008	Interferon response/Anti-bacterial
ABCD3	1:94874521	-0.0828	0.00386	8.89E-98	AFF3	2:100573658	Erythrocytes
ABCF2	7:150915471	0.0471	0.00295	4.36E-56	AGA	4:178361621	Interferon response/Anti-bacterial
ABCF3	3:183930979	-0.0259	0.00399	9.91E-11	AGFG1	2:228445810	CD4+ T-Cells
ABCG1	21:43641657	-0.0299	0.00446	2.01E-11	AGFG1	2:228445810	Erythrocytes
ABCG2	4:89090354	0.0602	0.00781	1.53E-14	AGFG1	2:228445810	Unknown
ABHD10	3:111697867	0.0884	0.00473	1.34E-75	AGPAT5	8:6556685	Erythrocytes
ABHD11	7:73149302	-0.0474	0.00318	2.90E-49	AHI1	6:135797808	Interferon response/Anti-bacterial
ABHD12	20:25333189	0.0582	0.00326	4.52E-69	AIF1	6:31548451	Erythrocytes
ABHD12B	14:51372103	0.217	0.00735	1.63E-176	AKAP10	17:19795946	Interferon response/Anti-bacterial
ABHD15	17:27917771	-0.0308	0.00359	1.39E-17	ALDH5A1	6:24464026	NK cells / CD8+ T-Cells
ABHD2	15:89612330	0.0832	0.0061	1.26E-41	ALOX15	17:4588856	Interferon response/Anti-bacterial
ABHD3	18:19235006	-0.0492	0.00545	2.61E-19	ALOX15	17:4559393	Neutrophils 10
ABHD4	14:23075116	0.0246	0.00318	1.13E-14	ANK2	4:113995834	Erythrocytes
ABI1	10:27033193	-0.029	0.0027	1.01E-26	ANKDD1A	15:65198309	Interferon response/Anti-bacterial
ABI3BP	3:100468407	-0.0195	0.00197	6.56E-23	ANPEP	15:90389045	Interferon response/Anti-bacterial

ABL2	1:179103834	-0.0401	0.00584	7.22E-12	AP3B1	5:77499698	Monocytes / Macrophages
ABLIM3	5:148471603	0.0702	0.0113	5.03E-10	AP3S2	15:90403035	Interferon response/Anti-bacterial
ABO	9:136139907	0.0265	0.00409	1.03E-10	APIP	11:34919083	Interferon response/Anti-bacterial
ABRACL	6:139350182	0.145	0.00683	1.49E-95	APOBR	16:28508447	Interferon response/Anti-bacterial
ABTB1	3:127390525	-0.0576	0.00583	8.54E-23	APPL1	3:57305253	Erythrocytes
ABTB2	11:34423774	-0.0221	0.00248	7.61E-19	ARFIP1	4:153695779	Interferon response/Anti-bacterial
ACAA2	18:47296298	0.0484	0.00586	1.84E-16	ARHGAP44	17:12931969	Erythrocytes
ACACA	17:35782182	0.0212	0.0032	3.79E-11	ARHGAP44	17:12750576	Monocytes / Macrophages
ACACB	12:109592558	0.0539	0.00289	1.96E-75	ARHGAP44	17:12706535	Neutrophils 1
ACAD8	11:134139780	0.0487	0.00351	5.66E-43	ARHGAP44	17:12932753	Neutrophils 2
ACAD9	3:128619505	-0.236	0.0148	6.95E-56	ARL15	5:53645362	Interferon response/Anti-bacterial
ACADM	1:76174280	0.199	0.0117	1.47E-63	ARNTL	11:13305689	Interferon response/Anti-bacterial
ACADSB	10:124790152	-0.0294	0.00483	1.21E-09	AS3MT	10:104623053	Interferon response/Anti-bacterial
ACADVL	17:7164563	0.0483	0.00434	1.59E-28	ASAH1	8:17928023	Interferon response/Anti-bacterial
ACAP1	17:7234112	0.124	0.00735	4.24E-62	ASNSD1	2:190583569	Interferon response/Anti-bacterial
ACAT1	11:108047982	-0.0763	0.00514	6.72E-49	ASPN	9:95172242	Interferon response/Anti-bacterial
ACAT2	6:160211554	-0.0325	0.00472	6.46E-12	ATXN7L3B	12:74960460	Interferon response/Anti-bacterial
ACBD3	1:226372266	0.0408	0.00377	4.22E-27	BACE2	21:42660973	Erythrocytes
ACCS	11:44078247	-0.224	0.00473	<1.0E-314	BACE2	21:42517239	Monocytes / Macrophages
ACER3	11:76654777	-0.128	0.00527	1.24E-122	BCAR3	1:94127935	Erythrocytes
ACIN1	14:23553110	-0.0646	0.00403	2.24E-56	BCL11A	2:60785864	Erythrocytes
ACN9	7:96754216	0.243	0.0317	1.98E-14	BCL11A	2:60775429	Monocytes / Macrophages
ACO1	9:32455320	-0.0704	0.00471	1.34E-49	BCL11A	2:60775429	Neutrophils 1
ACO2	22:41854446	-0.0246	0.00371	3.71E-11	BLK	8:11443007	Erythrocytes
ACOT4	14:74042189	-0.0815	0.0078	2.41E-25	BMF	15:40397191	Erythrocytes
ACOT8	20:44478114	-0.021	0.00308	1.11E-11	BMPR1A	10:88511532	Erythrocytes
ACOX1	17:73959857	0.19	0.00443	<1.0E-314	BMPR2	2:203395906	Erythrocytes
ACP1	2:290003	-0.132	0.0104	3.48E-36	BTN2A1	6:26465768	Interferon response/Anti-bacterial
ACP2	11:47257340	0.0623	0.00466	4.71E-40	BTN3A2	6:26356853.1	Interferon response/Anti-bacterial
ACP6	1:147135406	0.0649	0.00405	1.63E-56	BTN3A2	6:26377385.1	Monocytes / Macrophages
ACPL2	3:140938546	-0.134	0.00453	4.45E-178	BTN3A2	6:26356038	Neutrophils 10
ACPP	3:131989090	0.172	0.00805	2.36E-97	BTN3A2	6:26356038.1	NK cells / CD8+ T-Cells
ACSL3	2:223724984	0.0263	0.00429	8.74E-10	BTNL3	5:180366055	Erythrocytes
ACSL5	10:114149860	0.0716	0.00321	3.00E-105	BTNL3	5:180375306	Interferon response/Anti-bacterial
ACSL6	5:131322241	-0.0714	0.00449	1.41E-55	BTNL3	5:180430797	Monocytes / Macrophages
ACSM1	16:20674116	-0.0487	0.00455	1.94E-26	BTNL3	5:180365855	NK cells / CD8+ T-Cells
ACSM3	16:20733134	0.0509	0.00644	3.34E-15	C11orf80	11:66583502	Erythrocytes
ACSS1	20:25039046	0.0187	0.00279	2.19E-11	C11orf80	11:66583502	Monocytes / Macrophages
ACSS2	20:33468793	-0.0212	0.00287	1.92E-13	C11orf80	11:66583502	Neutrophils 1

ACSS3	12:81423842	-0.102	0.00744	2.37E-42	C11orf80	11:66583502	Neutrophils 2
ACTA2	10:90741615	-0.219	0.0052	<1.0E-314	C14orf159	14:91572577	Interferon response/Anti-bacterial
ACTB	7:5534598	1.1	0.0237	<1.0E-314	C15orf57	15:40869650	Interferon response/Anti-bacterial
ACTL6A	3:179336625	0.0261	0.00322	6.38E-16	C1orf85	1:156272271	Interferon response/Anti-bacterial
ACTN1	14:69466699	0.0268	0.0039	7.21E-12	C1orf85	1:156220056	Monocytes / Macrophages
ACTR10	14:58696823	0.16	0.0118	4.18E-41	C22orf32	22:42492063	Interferon response/Anti-bacterial
ACTR1A	10:104223628	-0.0223	0.00356	3.83E-10	C22orf32	22:42493036	NK cells / CD8+ T-Cells
ACTR1B	2:98271946	0.0555	0.00395	3.49E-44	C22orf34	22:50009300	Interferon response/Anti-bacterial
ACTR2	2:65504780	0.0772	0.00416	1.33E-74	C4BPA	1:207275799	CD4+ T-Cells
ACTR3B	7:152461934	-0.24	0.0388	6.67E-10	C4BPA	1:207275294	Erythrocytes
ACTR5	20:37406869	0.0576	0.00379	4.72E-51	C4BPA	1:207272272	Interferon response/Anti-bacterial
ACTR8	3:53899580	0.0445	0.00395	3.93E-29	C4BPA	1:207275799	Monocytes / Macrophages
ACVR2A	2:148710361	-0.0473	0.00499	3.92E-21	C4BPA	1:207280739	Neutrophils 10
ACVR2B	3:38507570	-0.0345	0.00314	7.89E-28	C4BPA	1:207279481	NK cells / CD8+ T-Cells
ACYP2	2:54368706	0.0354	0.00463	2.53E-14	C4BPA	1:207269858	Unknown
ADA	20:43271392	-0.0522	0.00453	2.29E-30	C9orf78	9:132554195	Interferon response/Anti-bacterial
ADAL	15:43618863	0.149	0.00918	4.52E-58	CABLES1	18:20768822	Erythrocytes
ADAM12	10:127660351	0.0298	0.00316	5.67E-21	CACNB2	10:18382714	Erythrocytes
ADAM15	1:155033918	0.0312	0.00363	1.09E-17	CACNB2	10:18404550	Monocytes / Macrophages
ADAM17	2:9647609	-0.0677	0.00411	1.42E-59	CACNB2	10:18398683	Neutrophils 1
ADAM19	5:156928008	-0.0611	0.00452	6.73E-41	CAT	11:34456108	Neutrophils 10
ADAM20	14:71048562	0.107	0.00858	2.29E-35	CCDC125	5:68598839	Interferon response/Anti-bacterial
ADAM22	7:87539249	0.0378	0.00459	2.08E-16	CCDC15	11:124933582	Interferon response/Anti-bacterial
ADAM23	2:207414601	-0.0505	0.00623	6.31E-16	CCDC171	9:15574495	Erythrocytes
ADAM28	8:24219671	0.0925	0.00711	3.96E-38	CCDC171	9:15504000	Interferon response/Anti-bacterial
ADAM32	8:38965271	-0.0659	0.0033	1.86E-85	CCDC23	1:43267130	Interferon response/Anti-bacterial
ADAM9	8:38842839	0.0636	0.00678	8.53E-21	CCDC23	1:43249620	NK cells / CD8+ T-Cells
ADAMDEC1	8:24220787	0.0248	0.00303	3.34E-16	CCDC88A	2:55480212	Erythrocytes
ADAMTS1	21:28215827	0.0723	0.00559	9.18E-38	CCDC88A	2:55480212	Monocytes / Macrophages
ADAMTS6	5:64823492	-0.134	0.0044	3.86E-188	CCL2	17:32538280	B-cells
ADAMTSL4	1:150539971	0.103	0.00986	1.65E-25	CCL2	17:32532532	Neutrophils 1
ADAR	1:154557685	0.0478	0.00435	7.53E-28	CCR3	3:46289113	Interferon response/Anti-bacterial
ADARB1	21:46564154	0.0446	0.00439	5.40E-24	CCR3	3:46276878	Neutrophils 10
ADARB2	10:1282530	-0.0302	0.00229	2.95E-39	CD28	2:204589944	Monocytes / Macrophages
ADAT1	16:75647536	0.104	0.00434	2.01E-120	CD3D	11:118167495	Monocytes / Macrophages
ADAT2	6:143789040	-0.0397	0.00502	3.49E-15	CD55	1:207489959	Interferon response/Anti-bacterial
ADCK1	14:78222798	-0.144	0.00677	1.65E-96	CD83	6:14159371	Monocytes / Macrophages
ADCK2	7:140359732	0.0685	0.00711	9.06E-22	CD93	20:23102752	Interferon response/Anti-bacterial
ADCK3	1:227175245	-0.0333	0.00265	9.20E-36	CD96	3:111304090	Monocytes / Macrophages

ADCY10	1:167737311	0.0309	0.00448	6.40E-12	CDC14A	1:100880328	Interferon response/Anti-bacterial
ADCY7	16:50369888	-0.0428	0.0029	2.12E-48	CDC16	13:115058408	Erythrocytes
ADCY9	16:4161793	-0.0647	0.0035	7.36E-74	CDC16	13:115047305	Interferon response/Anti-bacterial
ADD1	4:2854613	-0.0329	0.00449	2.72E-13	CDC42BPB	14:103505057	Erythrocytes
ADD3	10:111840725	-0.0571	0.0042	1.63E-41	CDC7	1:92021611	NK cells / CD8+ T-Cells
ADH6	4:100145592	-0.0725	0.00756	1.18E-21	CDS2	20:5141763	Interferon response/Anti-bacterial
ADHFE1	8:67377417	-0.0969	0.00471	1.18E-90	CELSR1	22:46970868	Erythrocytes
ADI1	2:3519283	0.127	0.00783	8.45E-58	CELSR1	22:46857853	Monocytes / Macrophages
ADIPOR2	12:1831355	-0.0323	0.00327	8.61E-23	CENPC1	4:68297763	Interferon response/Anti-bacterial
ADK	10:75928933	-0.364	0.00836	<1.0E-314	CENPK	5:64902091	Interferon response/Anti-bacterial
ADM	11:10294293	-0.0286	0.0033	6.01E-18	CENPK	5:64858687	Monocytes / Macrophages
ADO	10:64566572	0.0386	0.00578	2.74E-11	CENPK	5:64859027	NK cells / CD8+ T-Cells
ADORA1	1:203106293	0.0186	0.00261	1.11E-12	CENPK	5:64787704	Unknown
ADORA2B	17:15879303	0.09	0.00374	1.56E-121	CEP192	18:13093774	Interferon response/Anti-bacterial
ADORA3	1:112042149	0.0578	0.00549	1.07E-25	CFD	19:886257	Interferon response/Anti-bacterial
ADPGK	15:73018764	0.0426	0.00357	2.83E-32	CFL2	14:35176730	Interferon response/Anti-bacterial
ADPRH	3:119313876	0.169	0.0117	1.26E-46	CHI3L1	1:203155882	Interferon response/Anti-bacterial
ADPRM	17:10617803	0.197	0.00517	1.42E-281	CHPT1	12:102086805	Interferon response/Anti-bacterial
ADRB2	5:148207447	-0.0761	0.00496	5.24E-52	CISD1	10:60004851	Interferon response/Anti-bacterial
ADSS	1:244524272	0.0959	0.0148	9.53E-11	CLEC12A	12:10118747	Erythrocytes
ADTRP	6:11714197	-0.0451	0.0046	1.69E-22	CLEC12A	12:10118428	Interferon response/Anti-bacterial
AEBP2	12:19687105	-0.0354	0.00477	1.37E-13	CLEC12A	12:10126249	Monocytes / Macrophages
AFAP1	4:7871623	-0.122	0.00302	<1.0E-314	CLEC12A	12:10118262	Neutrophils 1
AFF1	4:87990864	-0.0248	0.00373	3.13E-11	CLEC12A	12:10118747	Neutrophils 10
AFF3	2:100741766	0.0478	0.00498	1.07E-21	CLEC12A	12:10117369	NK cells / CD8+ T-Cells
AFF4	5:132202411	-0.0314	0.00329	1.81E-21	CLEC12B	12:10118747	Erythrocytes
AFG3L1P	16:90063600	0.0581	0.00987	4.14E-09	CLEC12B	12:10118135	Interferon response/Anti-bacterial
AFG3L2	18:12327752	0.0443	0.0045	1.12E-22	CLEC12B	12:10118262	Neutrophils 1
AGA	4:178360550	0.178	0.00437	<1.0E-314	CLEC12B	12:10117369	NK cells / CD8+ T-Cells
AGAP1	2:236619055	-0.0458	0.00573	1.67E-15	CLEC17A	19:14763856	Erythrocytes
AGAP3	7:150787450	0.0141	0.00195	5.27E-13	CLEC4D	12:8682619	Interferon response/Anti-bacterial
AGER	6:32154285	-0.033	0.0038	5.91E-18	CLEC4F	2:71049129	Interferon response/Anti-bacterial
AGFG1	2:228295696	0.0322	0.00518	5.34E-10	CLEC4F	2:71052580	Monocytes / Macrophages
AGL	1:100310736	0.0266	0.00341	8.32E-15	CLEC9A	12:10190537	Interferon response/Anti-bacterial
AGMO	7:15287676	-0.0293	0.00298	1.42E-22	CLHC1	2:55459373	Interferon response/Anti-bacterial
AGPAT1	6:32136547	-0.0327	0.00336	2.75E-22	CLHC1	2:55459373	Monocytes / Macrophages
AGPAT4	6:161635526	-0.0339	0.00567	2.28E-09	CLN6	15:68540531	Interferon response/Anti-bacterial
AGPAT5	8:6587922	-0.0499	0.00459	3.74E-27	CLNK	4:10571872	CD4+ T-Cells
AGPAT9	4:84504821	0.0971	0.00614	4.13E-55	CLNK	4:10510869	Erythrocytes

AGPS	2:178216029	-0.0424	0.00445	2.65E-21	CLNK	4:10452986	Monocytes / Macrophages
AHCTF1	1:247115645	0.0641	0.00814	4.04E-15	CLNK	4:10466181	Neutrophils 1
AHCYL2	7:128861075	-0.0274	0.00395	4.77E-12	CLNK	4:10562143	Neutrophils 2
AHI1	6:135624811	0.187	0.0045	<1.0E-314	CLNK	4:10649812	NK cells / CD8+ T-Cells
AHR	7:17319630	-0.204	0.0216	5.25E-21	CLUHP3	16:31754654	Interferon response/Anti-bacterial
AHRR	5:299148	-0.075	0.00534	5.42E-44	CMBL	5:10286742	Interferon response/Anti-bacterial
AHSA1	14:77929941	-0.0607	0.00343	4.59E-68	CMBL	5:10281549	NK cells / CD8+ T-Cells
AHSA2	2:61404931	0.0842	0.00467	1.32E-70	CNR1	6:88856431	Erythrocytes
AHSP	16:31521334	0.201	0.0276	3.81E-13	CNR1	6:88856431	Monocytes / Macrophages
AIF1	6:31580539	0.0472	0.00526	3.88E-19	CNTNAP1	17:40810228	Erythrocytes
AIFM2	10:71910630	0.0176	0.00267	4.32E-11	COCH	14:31308704	Erythrocytes
AIG1	6:143376126	-0.0842	0.00794	5.59E-26	COCH	14:31308704	Monocytes / Macrophages
AIMP2	7:6061915	-0.0678	0.00422	7.01E-57	COPS3	17:17183479	NK cells / CD8+ T-Cells
AK2	1:33459484	0.0869	0.00565	2.98E-52	CORIN	4:47845299	Interferon response/Anti-bacterial
AK3	9:4758191	0.102	0.0165	6.34E-10	COX6C	8:100869351	Interferon response/Anti-bacterial
AK5	1:77897688	-0.243	0.00957	2.23E-134	CRISP2	6:49669359	Neutrophils 10
AKAP1	17:55176058	-0.0227	0.0035	9.41E-11	CRISP2	6:49669359	Neutrophils 2
AKAP10	17:19806050	-0.17	0.0051	2.18E-220	CRYZ	1:75217752	Interferon response/Anti-bacterial
AKAP11	13:42906121	-0.0317	0.00427	1.37E-13	CSGALNACT	8:19564149	Interferon response/Anti-bacterial
AKAP13	15:86251500	-0.0515	0.00709	4.42E-13	CSGALNACT	8:19564149	Unknown
AKAP7	6:131462299	0.116	0.0108	1.74E-26	CST7	20:24903185	Erythrocytes
AKD1	6:109815955	0.125	0.00628	1.66E-84	CYBRD1	2:172369069	Interferon response/Anti-bacterial
AKIP1	11:8909816	0.0611	0.0047	4.67E-38	CYP27A1	2:219630965	Erythrocytes
AKIRIN1	1:39453785	-0.0275	0.00398	5.06E-12	CYP27A1	2:219633071	Interferon response/Anti-bacterial
AKIRIN2	6:88402926	0.0466	0.0076	9.65E-10	DAD1	14:23062016	Interferon response/Anti-bacterial
AKNA	9:117180919	0.0166	0.00209	2.88E-15	DAPK1	9:90178594	Interferon response/Anti-bacterial
AKR1C2	10:4985193	-0.276	0.011	2.17E-130	DCAF10	9:37836508	Interferon response/Anti-bacterial
AKR1C3	10:5131180	-0.0851	0.0113	4.78E-14	DCAF10	9:37753512	NK cells / CD8+ T-Cells
AKR1E2	10:4874916	0.387	0.0172	2.13E-107	DCAF4	14:73393391	Interferon response/Anti-bacterial
AKT1	14:105258892	-0.0308	0.00369	7.51E-17	DCBLD1	6:117801639	Interferon response/Anti-bacterial
AKTIP	16:53522749	-0.161	0.00759	2.48E-95	DCLK2	4:150954757	Erythrocytes
ALAD	9:116167156	0.0356	0.00363	1.76E-22	DCLK2	4:150954757	Monocytes / Macrophages
ALCAM	3:105223763	-0.134	0.00463	9.12E-172	DCLK2	4:150950510	Neutrophils 1
ALDH1A1	9:75597138	-0.0728	0.00972	7.92E-14	DCTN5	16:23704005	Interferon response/Anti-bacterial
ALDH1B1	9:38397355	-0.0984	0.00515	7.94E-79	DGKH	13:42675906	Monocytes / Macrophages
ALDH2	12:112211833	0.063	0.00442	3.14E-45	DIP2A	21:47960493	CD4+ T-Cells
ALDH3A2	17:19559623	0.0435	0.00347	1.52E-35	DIP2A	21:47959698	Erythrocytes
ALDH5A1	6:24493922	0.204	0.00794	1.35E-137	DIP2A	21:47960493	Interferon response/Anti-bacterial
ALDH8A1	6:135271946	0.0483	0.00289	3.58E-61	DLG2	11:84086900	CD4+ T-Cells

ALDH9A1	1:165667781	0.0528	0.00435	2.01E-33	DLG2	11:84018349	Erythrocytes
ALDOB	9:104177498	0.0399	0.00312	6.43E-37	DLG2	11:84018349	Monocytes / Macrophages
ALDOC	17:26947652	0.0431	0.00513	5.76E-17	DLG2	11:83935158	Neutrophils 1
ALG11	13:52576498	0.0375	0.00379	8.47E-23	DLG2	11:83823752	Neutrophils 10
ALG6	1:63856651	-0.0502	0.00477	1.00E-25	DLG2	11:84202384	Neutrophils 2
ALG8	11:77777384	-0.0508	0.00467	2.67E-27	DNAJC15	13:43612736	Interferon response/Anti-bacterial
ALKBH1	14:78134243	-0.0376	0.00528	1.11E-12	DNMBP	10:101656449	Monocytes / Macrophages
ALKBH3	11:43947597	-0.0598	0.00389	2.59E-52	DOCK5	8:25248714	Erythrocytes
ALKBH4	7:102065322	0.108	0.00667	1.28E-57	DOCK5	8:25248714	Monocytes / Macrophages
ALKBH5	17:18127914	0.0201	0.00245	2.62E-16	DOCK7	1:63070537	Interferon response/Anti-bacterial
ALKBH8	11:107400136	-0.0662	0.00488	2.61E-41	DR1	1:93820575	Interferon response/Anti-bacterial
ALMS1	2:73613341	-0.0915	0.00355	3.30E-138	DSP	6:7495948	Interferon response/Anti-bacterial
ALMS1P	2:73837751	-0.0283	0.00359	3.79E-15	DSP	6:7495948	Monocytes / Macrophages
ALOX15	17:4568742	0.59	0.0189	5.41E-197	DZIP3	3:108264948	Interferon response/Anti-bacterial
ALOX5	10:45915941	0.0309	0.00432	1.00E-12	DZIP3	3:108350397	NK cells / CD8+ T-Cells
ALOX5AP	13:31312178	-0.158	0.0057	1.76E-158	ECM2	9:95207806	Interferon response/Anti-bacterial
ALPK1	4:113205543	0.0657	0.00572	3.73E-30	EFCAB13	17:45364390	Interferon response/Anti-bacterial
ALPL	1:21896925	0.0489	0.00766	1.76E-10	EFCAB13	17:45386412	Monocytes / Macrophages
ALS2	2:202639198	0.13	0.00393	1.35E-218	EFCAB2	1:245211973	Interferon response/Anti-bacterial
ALS2CR8	2:203733989	0.0639	0.00508	8.68E-36	EGR2	10:64533848	Erythrocytes
AMACR	5:33996456	-0.11	0.00434	1.38E-133	ENC1	5:73937854	Interferon response/Anti-bacterial
AMDHD1	12:96344495	0.0704	0.00302	2.33E-114	ENC1	5:73946488	Monocytes / Macrophages
AMFR	16:56428427	-0.0334	0.00472	1.68E-12	ENC1	5:73943431	Unknown
AMICA1	11:118072373	0.0871	0.00418	1.04E-92	ENPP2	8:120656832	Monocytes / Macrophages
AMIGO2	12:47473342	0.059	0.00492	1.07E-32	EPB41L2	6:131394493	Monocytes / Macrophages
AMPD3	11:10531025	-0.0319	0.00451	1.74E-12	ERAP2	5:96273033	Erythrocytes
AMPH	7:38388163	0.0242	0.00268	2.17E-19	ERAP2	5:96252589	Interferon response/Anti-bacterial
AMT	3:49453834	-0.0362	0.00293	1.49E-34	ERAP2	5:96252432	NK cells / CD8+ T-Cells
AMY2B	1:104128773	0.157	0.0115	8.96E-42	ERO1L	14:53160317	Interferon response/Anti-bacterial
AMZ2	17:66253696	0.036	0.00398	2.15E-19	ETV7	6:36315002	B-cells
AMZ2P1	17:62985398	-0.197	0.00775	7.09E-135	ETV7	6:36315002	Neutrophils 1
ANAPC13	3:134191880	0.0817	0.0061	3.19E-40	ETV7	6:36315002	Unknown
ANAPC15	11:71812592	0.042	0.00579	4.77E-13	EYA3	1:28416157	Interferon response/Anti-bacterial
ANAPC4	4:25397036	0.0545	0.00544	2.06E-23	F2RL1	5:76135511	Interferon response/Anti-bacterial
ANG	14:21153616	0.0698	0.00369	3.85E-77	FADS2	11:61556039	Interferon response/Anti-bacterial
ANGEL1	14:77284416	0.0194	0.00295	4.90E-11	FADS2	11:61547068	NK cells / CD8+ T-Cells
ANGEL2	1:213192458	0.0457	0.0034	1.24E-40	FAM117B	2:203505015	Monocytes / Macrophages
ANGPT1	8:108554458	0.101	0.0069	5.94E-48	FAM118A	22:45714489	CD4+ T-Cells
ANGPT2	8:6340423	-0.111	0.0135	1.97E-16	FAM118A	22:45731050	Erythrocytes

ANGPTL2	9:129814065	0.0459	0.00343	3.41E-40	FAM118A	22:45731731	Interferon response/Anti-bacterial
ANGPTL3	1:63018987	0.0318	0.00472	1.73E-11	FAM118A	22:45726345	Monocytes / Macrophages
ANK1	8:41506152	0.044	0.00721	1.15E-09	FAM118A	22:45745439	Neutrophils 1
ANK3	10:62488381	0.162	0.00506	3.38E-206	FAM118A	22:45771974	NK cells / CD8+ T-Cells
ANKDD1A	15:65163664	-0.113	0.00275	<1.0E-314	FAM134A	2:220035581	NK cells / CD8+ T-Cells
ANKH	5:14872689	0.169	0.00635	3.72E-146	FAM3B	21:42689637	Erythrocytes
ANKMY1	2:241481778	-0.0553	0.00312	4.95E-68	FAM3B	21:42690424	Interferon response/Anti-bacterial
ANKRA2	5:72894812	0.0596	0.00439	2.53E-41	FAM3B	21:42690424	Monocytes / Macrophages
ANKRD10	13:111547103	0.0741	0.00325	8.05E-110	FAM3B	21:42697714	NK cells / CD8+ T-Cells
ANKRD12	18:9109484	-0.0276	0.00399	4.65E-12	FAM3B	21:42690424	Unknown
ANKRD13D	11:67021532	-0.0915	0.0152	1.86E-09	FAN1	15:31157666	Interferon response/Anti-bacterial
ANKRD18DP	3:197814108	0.0376	0.00551	1.02E-11	FARP1	13:98945248	Erythrocytes
ANKRD26	10:27436566	-0.152	0.0098	4.46E-53	FBN2	5:127851383	Interferon response/Anti-bacterial
ANKRD27	19:33143605	-0.177	0.0127	2.73E-43	FBN2	5:127812816	Monocytes / Macrophages
ANKRD28	3:15694630	-0.188	0.00742	3.32E-133	FBXW8	12:117309440	Unknown
ANKRD32	5:94043608	0.181	0.0109	3.09E-60	FCN1	9:137798445	Interferon response/Anti-bacterial
ANKRD34B	5:79905411	0.257	0.00705	1.98E-259	FCRL2	1:157767519	Neutrophils 1
ANKRD36B	2:98086584	0.399	0.0336	4.84E-32	FCRL3	1:157603186	Erythrocytes
ANKRD36BP1	1:168234645	0.0477	0.0059	8.10E-16	FCRL5	1:157554177	Erythrocytes
ANKRD40	17:48767431	0.0283	0.00478	3.22E-09	FCRL5	1:157536682	Interferon response/Anti-bacterial
ANKRD44	2:198175179	0.0183	0.00311	4.14E-09	FCRL5	1:157526021	Monocytes / Macrophages
ANKRD5	20:10015729	0.147	0.0055	2.49E-148	FDFT1	8:11650918	Interferon response/Anti-bacterial
ANKRD50	4:125576279	0.114	0.0106	1.22E-26	FDFT1	8:11660614	NK cells / CD8+ T-Cells
ANKRD55	5:55444683	0.189	0.0113	2.96E-61	FGFR2	10:123210812	Interferon response/Anti-bacterial
ANKS1A	6:34856345	-0.0292	0.00381	2.44E-14	FGFR2	10:123211880	Neutrophils 10
ANO5	11:22231413	-0.0972	0.00446	5.22E-101	FHDC1	4:153893002	CD4+ T-Cells
ANP32B	9:100763890	0.0437	0.00735	2.94E-09	FLCN	17:17189404	NK cells / CD8+ T-Cells
ANP32E	1:150165849	0.11	0.00642	1.75E-64	FLNB	3:57948942	Neutrophils 10
ANPEP	15:90363995	0.271	0.00743	1.01E-259	FLVCR1-AS1	1:213039536	Interferon response/Anti-bacterial
ANTXR2	4:80821935	0.064	0.00466	4.29E-42	FMO3	1:171132137	Erythrocytes
ANXA1	9:75765478	-0.128	0.00814	9.41E-55	FMO3	1:171132137	Monocytes / Macrophages
ANXA11	10:81920010	-0.041	0.00394	4.02E-25	FMO3	1:171132137	Neutrophils 1
ANXA2	15:60689877	-0.0494	0.00556	7.90E-19	FMOD	1:203263699	Erythrocytes
ANXA2R	5:43039793	-0.078	0.00597	1.93E-38	FMOD	1:203263699	Monocytes / Macrophages
ANXA3	4:79473394	-0.147	0.0107	2.47E-42	FNBP1L	1:93898666	Interferon response/Anti-bacterial
ANXA4	2:69989801	0.0803	0.00505	1.21E-55	FNDC3B	3:171967454	Erythrocytes
ANXA5	4:122582127	0.144	0.00527	9.16E-154	FOLR3	11:71850156	Interferon response/Anti-bacterial
ANXA6	5:150534544	0.0828	0.0043	1.06E-79	FOLR3	11:71864967	Monocytes / Macrophages
AOAH	7:36758237	-0.158	0.00486	5.27E-212	FOLR3	11:71870070	Neutrophils 1

AP1B1	22:29785783	0.0302	0.00357	3.19E-17	FOLR3	11:71895857	NK cells / CD8+ T-Cells
AP1G1	16:71856611	-0.0201	0.00321	4.74E-10	FOXN3-AS2	14:90046899	Interferon response/Anti-bacterial
AP1M1	19:16321911	0.0319	0.00347	5.79E-20	FOXP4	6:41543584	Erythrocytes
AP1M2	19:10726288	0.0391	0.00494	2.72E-15	FPGT	1:74666344	Interferon response/Anti-bacterial
AP1S3	2:224689805	-0.077	0.00558	1.51E-42	FPR2	19:52269634	Interferon response/Anti-bacterial
AP2A2	11:1023045	-0.0805	0.0102	3.27E-15	FRA10AC1	10:95454768	Interferon response/Anti-bacterial
AP2B1	17:33892087	-0.0514	0.00439	2.41E-31	FRK	6:116364901	Erythrocytes
AP2M1	3:183899832	0.0776	0.00688	3.62E-29	FRK	6:116364901	Monocytes / Macrophages
AP3B1	5:77572367	-0.0397	0.00314	3.01E-36	FRMD6	14:52238848	Interferon response/Anti-bacterial
AP3B2	15:83378248	-0.0431	0.00349	1.62E-34	FUT10	8:33348000	Interferon response/Anti-bacterial
AP3M1	10:75845885	-0.06	0.00478	1.17E-35	FYB	5:39075286	Erythrocytes
AP3M2	8:42046212	0.0402	0.00432	1.95E-20	FYN	6:112192795	Erythrocytes
AP3S2	15:90418413	-0.162	0.00499	2.60E-210	FYN	6:112190113	Monocytes / Macrophages
AP4B1	1:114425426	0.0438	0.00406	6.00E-27	FYN	6:112190113	Neutrophils 2
AP4E1	15:51286576	-0.0903	0.0039	3.40E-113	GAB1	4:144362114	Erythrocytes
AP4M1	7:99685218	-0.0242	0.00266	1.19E-19	GAB1	4:144362114	Monocytes / Macrophages
AP5M1	14:57758539	0.0455	0.0076	2.33E-09	GAB1	4:144362114	Neutrophils 1
APAF1	12:99032580	-0.0742	0.00452	2.89E-59	GABRB2	5:160694519	Erythrocytes
APBA2	15:29214522	-0.0678	0.00858	3.30E-15	GABRB2	5:160961303	Monocytes / Macrophages
APBB1IP	10:26733355	-0.079	0.00405	5.66E-82	GABRB2	5:160974394	Neutrophils 1
APBB2	4:41216190	-0.091	0.00376	5.21E-123	GALNT2	1:230199116	CD4+ T-Cells
APC	5:112089480	-0.0357	0.0035	3.20E-24	GATAD2A	19:19577215	Interferon response/Anti-bacterial
APCDD1	18:10430913	0.0279	0.00415	1.97E-11	GATAD2A	19:19577215	NK cells / CD8+ T-Cells
APH1B	15:63595878	-0.0974	0.0115	3.29E-17	GBAP1	1:155201993	Interferon response/Anti-bacterial
API5	11:43338968	0.0384	0.00549	2.86E-12	GBP4	1:89622030	Neutrophils 1
APIP	11:34862466	-0.69	0.0134	<1.0E-314	GCC2	2:109048620	Interferon response/Anti-bacterial
APMAP	20:24909945	0.129	0.00695	6.90E-75	GCOM1	15:57855760	Interferon response/Anti-bacterial
APOA2	1:161178684	-0.0641	0.00496	1.22E-37	GDA	9:74815519	Erythrocytes
APOBEC3A	22:39358037	0.38	0.0238	3.15E-56	GDA	9:74815519	Interferon response/Anti-bacterial
APOBEC3B	22:39435855	0.123	0.00805	2.61E-51	GGTA1P	9:124273207	Interferon response/Anti-bacterial
APOBEC3D	22:39475964	0.0263	0.00384	8.62E-12	GLT25D2	1:183935353	Erythrocytes
APOBEC3G	22:39484178	0.138	0.00879	5.61E-54	GLT25D2	1:183939926	Interferon response/Anti-bacterial
APOBEC3H	22:39496902	0.137	0.0105	1.40E-38	GLYATL2	11:58626373	Erythrocytes
APOBEC4	1:183581965	-0.0434	0.00565	1.72E-14	GLYATL2	11:58638918	Interferon response/Anti-bacterial
APOBR	16:28473465	-2.69	0.0394	<1.0E-314	GM2A	5:150618993	Interferon response/Anti-bacterial
APOC4	19:45444646	-0.047	0.00661	1.33E-12	GNAO1	16:56406635	Erythrocytes
APOL2	22:36623148	-0.0531	0.00436	9.96E-34	GNRHR	4:68606934	Interferon response/Anti-bacterial
APOL3	22:36540434	0.0485	0.00415	3.45E-31	GPM6A	4:176856505	Erythrocytes
APP	21:27503527	-0.0439	0.00448	1.52E-22	GPM6A	4:176856505	Monocytes / Macrophages

APPL2	12:105659782	-0.0454	0.00306	7.55E-49	GPM6A	4:176856505	Neutrophils 1
APRT	16:88883288	0.145	0.0232	4.33E-10	GPR128	3:100344099	Erythrocytes
APTX	9:33025113	-0.0335	0.00352	2.99E-21	GPR128	3:100344099	Interferon response/Anti-bacterial
AQP10	1:154293923	-0.0672	0.0114	3.90E-09	GPR180	13:95266244	Interferon response/Anti-bacterial
AQP11	11:77369051	0.0432	0.00681	2.47E-10	GPX4	19:1118774	NK cells / CD8+ T-Cells
AQP3	9:33442986	-0.05	0.00444	4.56E-29	GRAMD1B	11:123514504	B-cells
AQR	15:35119748	0.0195	0.00285	9.49E-12	GRAMD1B	11:123514504	Interferon response/Anti-bacterial
ARAP1	11:72405450	0.0365	0.00416	2.66E-18	GRAMD1B	11:123514504	Neutrophils 1
ARAP3	5:141059158	0.0918	0.00547	1.46E-61	GSDMB	17:38026169	Interferon response/Anti-bacterial
ARF3	12:49351572	0.0723	0.00603	1.04E-32	GSTM3	1:110299052	Interferon response/Anti-bacterial
ARFGAP2	11:47188411	-0.0287	0.00334	1.17E-17	GSTM4	1:110198727	Interferon response/Anti-bacterial
ARFGAP3	22:43197897	0.0531	0.00448	4.64E-32	GSTM4	1:110175028	Neutrophils 10
ARFIP1	4:153681139	-0.222	0.00545	<1.0E-314	GSTP1	11:67352689	Interferon response/Anti-bacterial
ARG1	6:131898208	0.303	0.0116	2.77E-142	GSTT1	22:24366728	Interferon response/Anti-bacterial
ARHGAP11A	15:32907712	0.164	0.0146	4.61E-29	GUSBP5	4:144444995	Interferon response/Anti-bacterial
ARHGAP12	10:32247294	0.0831	0.00463	7.92E-70	HAUS4	14:23401441	Interferon response/Anti-bacterial
ARHGAP15	2:143884297	0.125	0.00705	1.01E-68	HBZ	16:207611	Interferon response/Anti-bacterial
ARHGAP19	10:99014575	-0.406	0.0136	7.76E-181	HBZ	16:209950	NK cells / CD8+ T-Cells
ARHGAP22	10:49822957	0.0185	0.00294	3.81E-10	HEATR6	17:58160568	Interferon response/Anti-bacterial
ARHGAP24	4:86590848	0.142	0.00728	2.28E-81	HERC2	15:28377772	Erythrocytes
ARHGAP25	2:69039254	-0.0269	0.00383	2.28E-12	HERC2	15:28530359	Interferon response/Anti-bacterial
ARHGAP26	5:142420072	-0.0792	0.00762	4.47E-25	HHAT	1:210820727	Erythrocytes
ARHGAP27	17:43551151	-0.03	0.00501	2.40E-09	HHAT	1:210842361	Monocytes / Macrophages
ARHGAP30	1:161026490	-0.0221	0.00296	8.88E-14	HIATL1	9:97201550	Interferon response/Anti-bacterial
ARHGAP32	11:129108747	0.0265	0.00404	5.91E-11	HIST1H2BF	6:26204506	Interferon response/Anti-bacterial
ARHGAP5	14:32592464	-0.0426	0.00726	4.72E-09	HIST1H3E	6:26208342	Interferon response/Anti-bacterial
ARHGAP9	12:57907769	0.147	0.00819	1.35E-69	HIST1H3E	6:26208342	Monocytes / Macrophages
ARHGEF10	8:1807449	-0.0649	0.00353	4.14E-73	HIST1H3E	6:26208641	Unknown
ARHGEF11	1:157005996	-0.0335	0.0041	3.69E-16	HIST1H4L	6:27810699	B-cells
ARHGEF19	1:16533830	-0.0155	0.00255	1.32E-09	HLA-B	6:31325235	Interferon response/Anti-bacterial
ARHGEF26	3:153962525	-0.0395	0.00374	6.90E-26	HLA-DOB	6:32796857	Interferon response/Anti-bacterial
ARHGEF3	3:56758448	-0.058	0.00553	1.74E-25	HLA-DOB	6:32743835	Monocytes / Macrophages
ARHGEF35	7:143858397	0.0576	0.00792	4.32E-13	HLA-DPA1	6:33035436	Interferon response/Anti-bacterial
ARHGEF40	14:21550499	-0.0691	0.00567	9.03E-34	HLA-DPB1	6:33052382	Interferon response/Anti-bacterial
ARHGEF7	13:111805525	0.0302	0.003	1.24E-23	HLA-DPB1	6:33048686	Unknown
ARID1B	6:157161912	0.0273	0.004	1.03E-11	HLA-DQA2	6:32668100	Interferon response/Anti-bacterial
ARID3B	15:74851416	0.0411	0.00567	4.84E-13	HLA-DRB1	6:32577385	Interferon response/Anti-bacterial
ARID4B	1:235491823	-0.0348	0.0054	1.20E-10	HLA-DRB1	6:32577442	Monocytes / Macrophages
ARID5A	2:97201682	-0.0136	0.00216	3.09E-10	HLA-DRB1	6:32574250	Neutrophils 10

ARL11	13:50209351	0.045	0.00547	2.21E-16	HLA-DRB1	6:32572461	NK cells / CD8+ T-Cells
ARL13B	3:93672516	-0.0343	0.00406	3.98E-17	HLA-DRB5	6:32438783	Interferon response/Anti-bacterial
ARL15	5:53606295	-0.305	0.00773	2.47E-298	HLA-DRB5	6:32438783	Monocytes / Macrophages
ARL17A	17:44603035	-0.377	0.0434	4.11E-18	HLA-DRB5	6:32438783	NK cells / CD8+ T-Cells
ARL4A	7:12730362	0.0608	0.00712	1.73E-17	HP	16:72105084	Interferon response/Anti-bacterial
ARL4C	2:235402656	0.0471	0.00473	4.17E-23	HPS1	10:100217808	NK cells / CD8+ T-Cells
ARL5B	10:18964097	-0.0666	0.00888	7.54E-14	HSD17B13	4:88231392	Interferon response/Anti-bacterial
ARL6IP1	16:18784310	0.0932	0.0104	4.08E-19	HSD17B13	4:88231865	Unknown
ARL6IP5	3:69153854	0.0281	0.00347	6.42E-16	HSD17B7P2	10:38681389	Interferon response/Anti-bacterial
ARL6IP6	2:153586899	0.0449	0.00523	1.25E-17	IGFBP4	17:38639764	Erythrocytes
ARMC1	8:66555467	0.0934	0.00459	9.17E-89	IGFBP4	17:38639764	Monocytes / Macrophages
ARMC3	10:23181000	0.0609	0.00444	4.10E-42	IGFBP4	17:38641599	Neutrophils 1
ARNT	1:150772613	-0.0719	0.00512	6.35E-44	IL18RAP	2:102991213	Erythrocytes
ARNTL	11:13294268	0.105	0.00425	1.98E-128	IL18RAP	2:103032180	Interferon response/Anti-bacterial
ARPC1B	7:98978755	-0.0301	0.0048	3.75E-10	IL18RAP	2:102991191	NK cells / CD8+ T-Cells
ARPC2	2:219105450	-0.0339	0.00257	3.89E-39	IL1RN	2:113863271	Neutrophils 1
ARPC5	1:183593512	-0.0494	0.0025	9.29E-84	IL4R	16:27285997	Erythrocytes
ARPP19	15:52854832	0.0751	0.00563	6.12E-40	IL4R	16:27395898	Monocytes / Macrophages
ARRB1	11:75075038	0.0679	0.0065	2.79E-25	IL7	8:79743403	Monocytes / Macrophages
ARRB2	17:4600510	-0.168	0.007	1.49E-120	IL7R	5:35856630	Interferon response/Anti-bacterial
ARRDC1	9:140533378	-0.0385	0.00495	9.16E-15	IL7R	5:35906488	Monocytes / Macrophages
ARSB	5:78281977	0.0961	0.0047	2.16E-89	INPP4A	2:99122921	Erythrocytes
ARSG	17:66413539	0.0336	0.00361	1.81E-20	INPP4A	2:99122921	Monocytes / Macrophages
ARV1	1:231101943	0.057	0.00525	3.19E-27	INPP4B	4:143290182	Monocytes / Macrophages
AS3MT	10:104611764	0.24	0.00581	<1.0E-314	INPP5F	10:121556259	Erythrocytes
ASAH1	8:17923339	-0.112	0.00373	4.75E-182	INPP5F	10:121555618	Monocytes / Macrophages
ASAP1	8:131269886	0.0855	0.00513	9.08E-61	INPP5F	10:121545752	Neutrophils 1
ASAP1-IT1	8:131270076	-0.0739	0.00517	1.79E-45	IRF4	6:403544	Erythrocytes
ASB13	10:5688795	0.0505	0.00412	3.82E-34	ISCA1	9:88947254	Interferon response/Anti-bacterial
ASB14	3:57321433	-0.0716	0.00691	5.95E-25	ITGA4	2:182425330	Erythrocytes
ASB16-AS1	17:42239349	-0.0717	0.00619	1.04E-30	ITGA4	2:182425330	Monocytes / Macrophages
ASB3	2:53955513	0.0378	0.00383	7.72E-23	ITGA6	2:173417021	Monocytes / Macrophages
ASB7	15:101176989	0.0448	0.00605	1.62E-13	ITGAX	16:31377113	Interferon response/Anti-bacterial
ASB8	12:48578809	0.0381	0.00559	1.00E-11	ITLN1	1:160827838	NK cells / CD8+ T-Cells
ASCC1	10:74025656	-0.207	0.0183	2.72E-29	ITSN1	21:35029572	Interferon response/Anti-bacterial
ASCC2	22:30220190	0.0675	0.00818	1.87E-16	JUP	17:39946867	Interferon response/Anti-bacterial
ASCC3	6:101328762	-0.0528	0.00632	7.64E-17	JUP	17:39947206	NK cells / CD8+ T-Cells
ASF1A	6:119178035	-0.0509	0.00696	2.93E-13	KCNJ15	21:39594637	Interferon response/Anti-bacterial
ASGR2	17:7015711	-0.11	0.00581	8.91E-78	KCNMA1	10:79246201	Monocytes / Macrophages

ASL	7:65595217	0.025	0.00246	4.59E-24	KCNMA1	10:79322102	Unknown
ASNS	7:97515313	-0.237	0.018	4.25E-39	KCNQ5	6:73957218	Erythrocytes
ASNSD1	2:190505697	0.184	0.00442	<1.0E-314	KHDRBS2	6:62851443	Erythrocytes
ASPDH	19:50973259	-0.0461	0.0039	5.85E-32	KIAA0040	1:175132938	Interferon response/Anti-bacterial
ASPH	8:62644645	-0.209	0.00839	3.91E-129	KIAA0226L	13:46936789	Erythrocytes
ASPN	9:95172242	-0.203	0.00534	4.99E-280	KIAA0226L	13:46905723	Neutrophils 1
ASPRV1	2:70207897	0.065	0.00715	1.42E-19	KIAA0391	14:35561440	Interferon response/Anti-bacterial
ASRGL1	11:62087343	-0.18	0.00722	1.36E-129	KIAA0391	14:35602567	Monocytes / Macrophages
ASUN	12:27054552	-0.0237	0.00392	1.60E-09	KIAA0391	14:35602087	Unknown
ATAD5	17:29177841	-0.107	0.00808	4.47E-39	KIAA1324	1:109706393	Erythrocytes
ATF1	12:51202554	-0.064	0.00832	1.79E-14	KIAA1324	1:109706880	Interferon response/Anti-bacterial
ATF6	1:161835754	-0.0751	0.0071	7.10E-26	KIAA1324	1:109708064	Monocytes / Macrophages
ATF7	12:54057912	0.0268	0.00406	4.89E-11	KIAA1324	1:109706393	NK cells / CD8+ T-Cells
ATF7IP2	16:10455828	0.193	0.00831	1.60E-113	KIAA1598	10:118712455	Interferon response/Anti-bacterial
ATG10	5:81299921	0.273	0.0105	1.77E-141	KIF16B	20:16552181	Interferon response/Anti-bacterial
ATG16L1	2:234147930	-0.0277	0.0036	1.61E-14	KIF16B	20:16545368	Monocytes / Macrophages
ATG5	6:106631860	0.0641	0.00573	1.02E-28	KIF1B	1:10271688	Interferon response/Anti-bacterial
ATG7	3:11294310	0.063	0.00379	1.35E-60	KRT23	17:39092063	Interferon response/Anti-bacterial
ATIC	2:216181279	-0.0644	0.00397	1.14E-57	L3MBTL3	6:130374461	Interferon response/Anti-bacterial
ATL1	14:51072713	-0.0292	0.00415	2.49E-12	LAMC1	1:182963380	Erythrocytes
ATL2	2:38505813	0.0295	0.00504	4.95E-09	LAMP3	3:182869163	B-cells
ATL3	11:63463937	0.0661	0.00951	4.05E-12	LAMP3	3:182861353	Neutrophils 1
ATMIN	16:81069317	-0.08	0.0102	4.92E-15	LBH	2:30509158	NK cells / CD8+ T-Cells
ATOX1	5:151128214	0.0296	0.00474	4.47E-10	LCA5L	21:40766048	Interferon response/Anti-bacterial
ATP10A	15:26075917	-0.0444	0.00322	1.41E-42	LCLAT1	2:30676422	Interferon response/Anti-bacterial
ATP10D	4:47442361	0.0576	0.00399	2.58E-46	LGALS2	22:37970808	Interferon response/Anti-bacterial
ATP11B	3:182575631	0.0857	0.00508	3.00E-62	LGALS2	22:37970866	Monocytes / Macrophages
ATP13A3	3:194237025	0.113	0.00932	1.73E-33	LINC00339	1:22355289	Interferon response/Anti-bacterial
ATP13A4	3:193300315	0.0835	0.00283	2.91E-177	LIPA	10:91011681	Interferon response/Anti-bacterial
ATP1A4	1:160136208	-0.0456	0.00357	9.52E-37	LIX1	5:96425293	Monocytes / Macrophages
ATP1B1	1:169079268	-0.128	0.00816	2.18E-54	LOC654433	2:113981137	Interferon response/Anti-bacterial
ATP1B3	3:141645747	0.0372	0.00569	6.58E-11	LOXHD1	18:44165341	Interferon response/Anti-bacterial
ATP2A3	17:3862165	0.0533	0.00874	1.18E-09	LOXHD1	18:44165341	Unknown
ATP2C2	16:84414164	-0.0271	0.00229	5.06E-32	LPL	8:19843930	Erythrocytes
ATP5A1	18:43714920	-0.131	0.017	1.68E-14	LPL	8:19860197	Monocytes / Macrophages
ATP5C1	10:7830064	-0.103	0.0047	3.73E-102	LRRC16A	6:25398426	Interferon response/Anti-bacterial
ATP5F1	1:111968710	-0.102	0.0101	1.55E-23	LRRC6	8:133718548	Interferon response/Anti-bacterial
ATP5G2	12:54087705	-0.032	0.00523	1.05E-09	LRRC6	8:133715804	NK cells / CD8+ T-Cells
ATP5G3	2:176042819	-0.0413	0.005	1.78E-16	LRRC8B	1:90031987	Interferon response/Anti-bacterial

ATP5L2	22:43046707	-0.0689	0.00966	1.13E-12	LRRC8B	1:90025981	Unknown
ATP5O	21:35284683	0.24	0.0112	1.69E-97	LRRN1	3:3831122	Monocytes / Macrophages
ATP5S	14:50789102	-0.0562	0.00332	1.09E-62	LYSMD3	5:89814604	Interferon response/Anti-bacterial
ATP5SL	19:41906270	-0.0504	0.00397	2.21E-36	LYZ	12:69732105	Interferon response/Anti-bacterial
ATP6V0D1	16:67549746	0.157	0.0084	2.37E-75	MACF1	1:39952956	Neutrophils 1
ATP6V1C1	8:104084584	0.119	0.00633	6.76E-76	MANEA	6:96024424	Interferon response/Anti-bacterial
ATP6V1D	14:67820457	-0.121	0.0167	4.33E-13	MAP2	2:210648810	Monocytes / Macrophages
ATP6V1E1	22:18077640	0.194	0.00623	3.07E-195	MAP2	2:210404154	Neutrophils 1
ATP6V1E2	2:46738846	0.0414	0.00467	1.02E-18	MAP2K5	15:67865045	Interferon response/Anti-bacterial
ATP7B	13:52626337	0.0226	0.00309	2.68E-13	MASTL	10:27474078	Interferon response/Anti-bacterial
ATP8A1	4:42676817	-0.14	0.01	1.60E-43	MASTL	10:27475444	Neutrophils 1
ATP8B1	18:55304929	0.0281	0.00452	5.20E-10	MBD4	3:129099921	Monocytes / Macrophages
ATP8B2	1:154313232	-0.0292	0.00422	5.16E-12	MBD4	3:129099921	Neutrophils 1
ATP8B4	15:50157261	0.14	0.00668	2.05E-94	MDGA1	6:37674339	Interferon response/Anti-bacterial
ATP9B	18:76812450	0.0653	0.00336	1.41E-81	MDM2	12:69184350	Interferon response/Anti-bacterial
ATPIF1	1:28560580	0.116	0.00373	7.79E-194	MDN1	6:90489072	Neutrophils 1
ATR	3:142340807	0.0173	0.00284	1.29E-09	MFN2	1:12031581	CD4+ T-Cells
ATRAID	2:27439751	-0.054	0.00677	1.85E-15	MFN2	1:12046880	Interferon response/Anti-bacterial
ATRIP	3:48484016	-0.0136	0.0023	3.83E-09	MFN2	1:12046063	NK cells / CD8+ T-Cells
ATRN	20:3594089	-0.0204	0.00289	1.96E-12	MFSD9	2:103347094	Interferon response/Anti-bacterial
ATXN1	6:16755768	0.0579	0.00386	1.15E-49	MFSD9	2:103347094	Monocytes / Macrophages
ATXN10	22:46052777	-0.106	0.00449	1.21E-116	MFSD9	2:103347857	Unknown
ATXN3	14:92550658	0.129	0.0046	1.52E-160	MGAT4A	2:99201070	Monocytes / Macrophages
ATXN7L3B	12:74946815	-0.14	0.00413	2.14E-227	MGAT4A	2:99201070	Neutrophils 1
AUH	9:94045131	0.0456	0.00466	1.68E-22	MGST3	1:165600267	Interferon response/Anti-bacterial
AUP1	2:74729481	-0.0525	0.00586	4.51E-19	MLLT3	9:20642438	Monocytes / Macrophages
AUTS2	7:70144841	-0.052	0.00457	1.28E-29	MOV10	1:113236087	Neutrophils 1
AVIL	12:58170335	-0.072	0.00469	3.68E-52	MPZL2	11:118126576	Interferon response/Anti-bacterial
AVL9	7:32618281	-0.0334	0.00453	2.17E-13	MRE11A	11:94232883	Interferon response/Anti-bacterial
AVPR1A	12:63541714	0.0291	0.0046	2.84E-10	MRPL18	6:160188891	Interferon response/Anti-bacterial
AXDND1	1:179322810	-0.179	0.0151	2.90E-32	MRPL21	11:68646845	Interferon response/Anti-bacterial
AXIN1	16:407723	0.0287	0.00273	1.11E-25	MRPS18C	4:84423038	Interferon response/Anti-bacterial
AXIN2	17:63556290	-0.0636	0.0055	1.38E-30	MSH2	2:47661644	Interferon response/Anti-bacterial
AZI2	3:28332087	0.0269	0.00409	5.05E-11	MSH3	5:79920073	Interferon response/Anti-bacterial
AZIN1	8:103817865	0.0316	0.00403	5.32E-15	MSH3	5:79913275	NK cells / CD8+ T-Cells
B3GALNT1	3:160855599	0.0265	0.00347	2.80E-14	MTPAP	10:30653561	Erythrocytes
B3GALNT2	1:235628908	0.2	0.00409	<1.0E-314	MTPAP	10:30647695	Interferon response/Anti-bacterial
B3GAT1	11:134268411	0.0313	0.00482	9.48E-11	MYH11	16:15820863	Interferon response/Anti-bacterial
B3GAT2	6:71531118	-0.0342	0.00563	1.26E-09	MYH11	16:15821246	NK cells / CD8+ T-Cells

B3GAT3	11:62424096	-0.0554	0.00931	2.80E-09	MYL6	12:56509535	B-cells
B3GNT2	2:62491643	0.106	0.00549	1.04E-79	MYL6	12:56509535	Erythrocytes
B3GNT5	3:182925233	-0.258	0.0164	2.10E-54	MYL6	12:56509535	Interferon response/Anti-bacterial
B3GNTL1	17:81006286	-0.0391	0.00277	1.54E-44	MYL6	12:56509535	Monocytes / Macrophages
B4GALNT3	12:662838	-0.069	0.0024	3.32E-169	MYL6	12:56509535	Neutrophils 1
B4GALT4	3:118957370	-0.174	0.00491	4.95E-247	MYL6	12:56509535	Neutrophils 2
B4GALT6	18:29255261	-0.0432	0.00461	9.46E-21	MYL6	12:56509535	Unknown
BABAM1	19:17391787	-0.0774	0.00948	4.18E-16	MYO10	5:16875276	Erythrocytes
BACE1	11:117189216	0.0199	0.00241	1.67E-16	MYO10	5:16875276	Monocytes / Macrophages
BACE2	21:42521213	0.0646	0.00535	3.87E-33	MYO10	5:16872627	Neutrophils 2
BACH1	21:30706470	0.0282	0.00398	1.64E-12	MYO1E	15:59422810	Erythrocytes
BAG3	10:121437012	0.032	0.00356	2.99E-19	MYO1E	15:59422810	Monocytes / Macrophages
BAG5	14:104022944	0.0376	0.00503	9.04E-14	MYO1E	15:59422810	Neutrophils 1
BAG6	6:31594181	-0.0732	0.008	7.66E-20	MYOM2	8:2121846	Interferon response/Anti-bacterial
BAHCC1	17:79339668	-0.272	0.0382	1.22E-12	NAAA	4:76816143	Interferon response/Anti-bacterial
BAI3	6:69454400	0.0277	0.00249	2.83E-28	NAGK	2:71294360	Interferon response/Anti-bacterial
BAIAP2	17:79015989	0.0184	0.00307	2.39E-09	NAPG	18:10536906	Interferon response/Anti-bacterial
BAIAP2L1	7:97942436	0.0516	0.00368	6.18E-44	NCF2	1:183553870	Erythrocytes
BAIAP3	16:1394741	0.0374	0.00306	5.06E-34	NDST1	5:149862325	Interferon response/Anti-bacterial
BAK1	6:33546837	-0.0755	0.00467	2.32E-57	NEBL	10:21503412	Interferon response/Anti-bacterial
BARD1	2:215673440	0.106	0.00529	4.37E-86	NECAP2	1:16791384	Interferon response/Anti-bacterial
BASP1	5:17234578	-0.0456	0.00464	1.48E-22	NETO1	18:70477566	Erythrocytes
BATF3	1:212871081	0.0398	0.00478	1.04E-16	NFXL1	4:47910943	CD4+ T-Cells
BAZ2B	2:160427646	-0.145	0.00527	1.49E-156	NFXL1	4:47888664	Erythrocytes
BBS2	16:56479298	0.219	0.00421	<1.0E-314	NFXL1	4:47858518	Interferon response/Anti-bacterial
BBS4	15:72978531	-0.149	0.0127	2.02E-31	NFXL1	4:47848377	Monocytes / Macrophages
BBS9	7:33344075	0.0705	0.00581	2.02E-33	NFXL1	4:47862792	NK cells / CD8+ T-Cells
BBX	3:107312676	0.0404	0.0054	8.00E-14	NFXL1	4:47906461	Unknown
BCAS4	20:49431913	-0.0402	0.00554	4.75E-13	NINL	20:25492584	Erythrocytes
BCAT1	12:25051265	-0.307	0.00623	<1.0E-314	NINL	20:25492584	Monocytes / Macrophages
BCKDK	16:31162303	0.0292	0.00409	1.11E-12	NIPAL2	8:99305539	Interferon response/Anti-bacterial
BCL2A1	15:80263345	0.162	0.00749	7.56E-100	NME4	16:450759	NK cells / CD8+ T-Cells
BCL2L11	2:111896243	0.0411	0.00686	2.27E-09	NMRK1	9:77707695	Interferon response/Anti-bacterial
BCL9	1:147020818	-0.0816	0.0107	3.01E-14	NOD2	16:50714979	CD4+ T-Cells
BCO2	11:112049856	-0.0269	0.00353	3.09E-14	NOD2	16:50724614	Interferon response/Anti-bacterial
BCR	22:23522810	-0.0205	0.0027	3.50E-14	NQO2	6:3028028	Interferon response/Anti-bacterial
BDH1	3:197273882	-0.0269	0.00428	3.73E-10	NRG1	8:32430888	Interferon response/Anti-bacterial
BDH2	4:104000973	-0.152	0.00747	2.00E-88	NSD1	5:176517058	Neutrophils 1
BDP1	5:70730687	-0.111	0.0149	1.01E-13	NSG1	4:4350655	Interferon response/Anti-bacterial

BEGAIN	14:101005215	-0.0319	0.00385	1.44E-16	NT5C3L	17:39991988	Interferon response/Anti-bacterial
BEND7	10:13560871	0.052	0.00499	3.18E-25	NT5C3L	17:39979267	Monocytes / Macrophages
BET1	7:93624179	0.0533	0.0056	2.90E-21	NUDT12	5:102855443	Interferon response/Anti-bacterial
BET1L	11:198986	-0.0925	0.00469	2.36E-83	OGN	9:95096811	Interferon response/Anti-bacterial
BFAR	16:14777006	0.031	0.00461	2.00E-11	OSBPL10	3:31742717	Erythrocytes
BFSP1	20:17473671	-0.0274	0.00392	3.38E-12	OSBPL10	3:31854210	Monocytes / Macrophages
BHLHE40	3:5025654	0.0625	0.00873	8.96E-13	OSBPL10	3:31854210	Neutrophils 1
BHMT2	5:78434291	-0.0223	0.00376	3.17E-09	OSBPL10	3:31854210	NK cells / CD8+ T-Cells
BICD2	9:95508176	-0.0344	0.00356	6.37E-22	P2RX1	17:3821967	Interferon response/Anti-bacterial
BID	22:18180911	0.0174	0.00225	1.23E-14	PACS1	11:66003843	Neutrophils 2
BIN1	2:127826533	-0.0596	0.00276	2.48E-99	PADI2	1:17425473	Interferon response/Anti-bacterial
BIN2	12:51685831	-0.112	0.0066	4.87E-63	PADI2	1:17414305	Monocytes / Macrophages
BIRC2	11:102227409	-0.112	0.0112	2.28E-23	PADI2	1:17414305	NK cells / CD8+ T-Cells
BIRC7	20:61875007	-0.0346	0.005	5.02E-12	PAM	5:102165082	CD4+ T-Cells
BIVM	13:103430929	-0.058	0.0056	6.77E-25	PAM	5:102151674	Erythrocytes
BLK	8:11339882	0.0517	0.00456	2.22E-29	PAM	5:102153434	Interferon response/Anti-bacterial
BLM	15:91351930	0.0724	0.00413	7.75E-67	PAM	5:102153433	Monocytes / Macrophages
BLMH	17:28637173	0.0738	0.00436	1.03E-62	PAM	5:102164118	Neutrophils 1
BLOC1S2	10:102011702	-0.198	0.0101	4.90E-83	PAM	5:102151674	Unknown
BLVRA	7:43845185	0.152	0.00648	1.58E-115	PAPSS1	4:108609628	Erythrocytes
BLVRB	19:40941154	0.207	0.0182	1.47E-29	PAPSS1	4:108615679	Interferon response/Anti-bacterial
BMF	15:40447265	0.0291	0.00311	1.08E-20	PAPSS1	4:108610005	Neutrophils 10
BMP6	6:7862631	0.0452	0.00515	2.55E-18	PAQR6	1:156219450	Interferon response/Anti-bacterial
BMPR1A	10:88635779	0.0516	0.0073	1.72E-12	PCTP	17:53840694	NK cells / CD8+ T-Cells
BMPR2	2:203270092	-0.0616	0.00499	1.69E-34	PEX6	6:42940673	Interferon response/Anti-bacterial
BMS1P1	10:47619049	-0.9	0.0984	8.66E-20	PF4V1	4:74718845	Interferon response/Anti-bacterial
BNC2	9:16876283	-0.0584	0.0048	1.44E-33	PFKP	10:3190499	Erythrocytes
BNIP1	5:172591337	0.0422	0.00604	3.22E-12	PGGT1B	5:114548071	Interferon response/Anti-bacterial
BNIP2	15:59966305	0.0979	0.00428	1.36E-110	PGM5	9:71175565	Interferon response/Anti-bacterial
BNIPL	1:151033979	-0.0248	0.00353	2.12E-12	PI4K2B	4:25290490	B-cells
BOD1	5:173046555	-0.0491	0.00776	2.86E-10	PIAS2	18:44380602	Unknown
BOD1L1	4:13580532	-0.0677	0.0101	2.29E-11	PIEZO2	18:10829069	Erythrocytes
BOLL	2:198568076	-0.0385	0.0036	2.41E-26	PISD	22:31998612	Interferon response/Anti-bacterial
BORA	13:73296812	0.257	0.0119	2.65E-99	PLB1	2:28677086	Interferon response/Anti-bacterial
BPI	20:36925819	-0.428	0.0345	6.53E-35	PLD6	17:17158343	NK cells / CD8+ T-Cells
BRCA2	13:32887616	0.0249	0.00328	3.40E-14	PLEKHA1	10:124156944	Monocytes / Macrophages
BRD1	22:50250464	-0.0291	0.00493	3.73E-09	PLIN2	9:19127489	Interferon response/Anti-bacterial
BRD2	6:32919524	-0.017	0.0029	5.09E-09	PLK1S1	20:21179145	Interferon response/Anti-bacterial
BRD3	9:136925451	-0.0213	0.00238	4.88E-19	PLOD1	1:12048595	Interferon response/Anti-bacterial

BRI3BP	12:125471505	-0.0388	0.00351	4.80E-28	PLXNC1	12:94606526	Erythrocytes
BRIP1	17:59967940	-0.0466	0.00619	5.45E-14	PLXNC1	12:94632933	Monocytes / Macrophages
BRK1	3:10165778	0.119	0.00686	7.92E-66	POLI	18:51781019	Interferon response/Anti-bacterial
BRWD1	21:40661881	-0.0845	0.00656	1.98E-37	PPFIBP2	11:7561609	Unknown
BSCL2	11:62451457	-0.219	0.0141	2.83E-53	PPIE	1:40213670	Interferon response/Anti-bacterial
BST1	4:15737348	0.0779	0.00509	7.91E-52	PPIP5K2	5:102450576	Interferon response/Anti-bacterial
BTAF1	10:93702042	-0.0221	0.00321	6.28E-12	PPIP5K2	5:102436268	Monocytes / Macrophages
BTBD10	11:13467982	0.0826	0.00553	2.26E-49	PPP1R16B	20:37506112	Erythrocytes
BTBD11	12:107718282	-0.108	0.00604	8.38E-70	PPP2R3C	14:35559545	Interferon response/Anti-bacterial
BTBD6	14:105722726	0.0908	0.0068	4.40E-40	PPP2R5A	1:212516456	Interferon response/Anti-bacterial
BTBD7	14:93751018	-0.0204	0.00309	4.10E-11	PPT1	1:40561471	Interferon response/Anti-bacterial
BTF3	5:72797720	-0.0626	0.0104	2.14E-09	PRDM5	4:121693006	Interferon response/Anti-bacterial
BTG2	1:203255493	0.0619	0.00395	4.65E-54	PRDX5	11:64076244	Interferon response/Anti-bacterial
BTN2A1	6:26465768	-0.178	0.00477	1.88E-270	PRDX6	1:173485336	Interferon response/Anti-bacterial
BTN2A2	6:26404374	-0.0697	0.00475	7.13E-48	PRKCA	17:64536982	Monocytes / Macrophages
BTN3A1	6:26404694	-0.0759	0.00491	1.04E-52	PRKCE	2:45925708	CD4+ T-Cells
BTN3A2	6:26316295	0.635	0.0133	<1.0E-314	PRKCE	2:45862997	Erythrocytes
BTN3A3	6:26391391	-0.0667	0.00999	2.74E-11	PRKCE	2:45862997	Monocytes / Macrophages
BTNL3	5:180365855	-0.477	0.00685	<1.0E-314	PRKCE	2:45925708	Neutrophils 1
BTNL9	5:180470133	-0.0221	0.00346	1.80E-10	PRKCQ	10:6542361	Monocytes / Macrophages
BUB3	10:124910214	-0.0634	0.00515	2.16E-34	PRKD3	2:37565276	Interferon response/Anti-bacterial
BUD13	11:116640768	0.0245	0.00314	6.32E-15	PRMT5	14:23430714	Interferon response/Anti-bacterial
BUD31	7:99015791	0.0632	0.00813	9.69E-15	PRUNE2	9:79318471	Erythrocytes
C10orf111	10:15134114	0.0593	0.00858	5.28E-12	PRUNE2	9:79326505	Interferon response/Anti-bacterial
C10orf137	10:127441439	0.0281	0.00468	2.17E-09	PSAP	10:73578151	Monocytes / Macrophages
C10orf32	10:104623053	0.0717	0.00669	1.49E-26	PSMA8	18:23766127	Erythrocytes
C10orf35	10:71384325	0.0282	0.00438	1.37E-10	PSMA8	18:23782005	Monocytes / Macrophages
C10orf54	10:73526468	-0.0305	0.00499	1.03E-09	PSMD13	11:240868	Interferon response/Anti-bacterial
C10orf68	10:33087660	-0.118	0.00519	8.31E-109	PTPLA	10:17598276	Monocytes / Macrophages
C10orf76	10:103657957	0.0329	0.00439	8.65E-14	PTPRG	3:62245373	Erythrocytes
C10orf88	10:124704695	-0.0359	0.00502	9.60E-13	PTPRG	3:62245373	Monocytes / Macrophages
C11orf21	11:2316720	-0.121	0.0102	7.61E-32	PTPRG	3:62245373	Neutrophils 1
C11orf30	11:76125330	0.0206	0.003	7.10E-12	PVRL2	19:45376317	Interferon response/Anti-bacterial
C11orf31	11:57493622	-0.0409	0.00437	1.04E-20	PVRL2	19:45372329	NK cells / CD8+ T-Cells
C11orf42	11:6228594	-0.0462	0.00618	8.37E-14	QKI	6:163871318	Interferon response/Anti-bacterial
C11orf48	11:62426194	-0.16	0.0119	1.86E-40	RABGAP1L	1:174995816	Erythrocytes
C11orf54	11:93500659	-0.0621	0.00411	1.62E-50	RABGAP1L	1:174995816	Monocytes / Macrophages
C11orf58	11:16748468	-0.0592	0.00687	8.44E-18	RAC1	7:6414762	Interferon response/Anti-bacterial
C11orf63	11:122748288	-0.0954	0.00514	1.61E-74	RAD51C	17:56816892	Interferon response/Anti-bacterial

C11orf71	11:114269266	0.0334	0.00401	1.03E-16	RAP1GAP	1:22023238	NK cells / CD8+ T-Cells
C11orf75	11:93231389	-0.169	0.00703	1.94E-121	RAPGEF2	4:160237160	B-cells
C11orf92	11:111197776	-0.0382	0.00477	1.28E-15	RASGRF1	15:79304104	Erythrocytes
C12orf23	12:107321748	-0.053	0.00518	2.39E-24	RASGRF1	15:79304104	Monocytes / Macrophages
C12orf29	12:88444592	-0.0393	0.00641	9.69E-10	RASGRF1	15:79268295	Neutrophils 1
C12orf39	12:21714448	-0.07	0.00632	2.91E-28	RASGRF2	5:80232479	Monocytes / Macrophages
C12orf4	12:4676899	0.0545	0.00863	2.96E-10	RBL2	16:53490676	Interferon response/Anti-bacterial
C12orf43	12:121438382	0.0252	0.00344	2.47E-13	RBMS3	3:29612955	Erythrocytes
C12orf44	12:52460424	0.0258	0.00417	6.52E-10	RBMS3	3:29612955	Monocytes / Macrophages
C12orf5	12:4446116	0.0827	0.00662	2.41E-35	RBPMS2	15:65098593	Interferon response/Anti-bacterial
C12orf60	12:14955702	-0.0442	0.00669	4.45E-11	RCBTB1	13:50159305	Interferon response/Anti-bacterial
C12orf65	12:123747783	-0.027	0.00413	6.26E-11	RIN2	20:19962931	Neutrophils 1
C12orf75	12:105754678	0.329	0.0248	1.62E-39	RNASE3	14:21349608	Neutrophils 10
C14orf101	14:57107856	-0.0599	0.00753	2.21E-15	RNASET2	6:167376466	Interferon response/Anti-bacterial
C14orf132	14:96468108	0.0485	0.00609	1.95E-15	RNASET2	6:167369992	Neutrophils 10
C14orf159	14:91581340	0.298	0.00907	3.02E-216	RNF182	6:13918461	Interferon response/Anti-bacterial
C14orf166	14:52471263	0.11	0.00931	5.09E-32	RNPEP	1:201954728	Interferon response/Anti-bacterial
C14orf2	14:104381687	0.119	0.00896	2.42E-39	ROR1	1:64498282	Erythrocytes
C15orf26	15:81397782	-0.0381	0.0047	6.03E-16	ROR1	1:64453767	Monocytes / Macrophages
C15orf37	15:80212491	-0.122	0.00503	3.56E-123	ROR1	1:64556996	Neutrophils 1
C15orf38	15:90455379	0.162	0.00631	2.43E-137	RORA	15:61451437	Monocytes / Macrophages
C15orf40	15:83646331	0.0273	0.00413	3.88E-11	RPH3A	12:113324731	Interferon response/Anti-bacterial
C15orf41	15:36870972	0.0328	0.00553	3.12E-09	RPL31	2:101629413	Interferon response/Anti-bacterial
C15orf54	15:39546830	0.298	0.0256	7.13E-31	RPS15A	16:18838751	Interferon response/Anti-bacterial
C15orf57	15:40848351	0.133	0.00451	2.33E-177	RPS18	6:33196122	B-cells
C16orf13	16:675067	-0.0815	0.0126	9.60E-11	RPS18	6:33196122	Unknown
C16orf54	16:29748924	-0.0593	0.00465	1.08E-36	RPS25	11:118892787	Neutrophils 1
C16orf55	16:89716222	0.0653	0.00754	6.71E-18	RRP12	10:99143878	Interferon response/Anti-bacterial
C16orf62	16:19634841	0.0243	0.0037	5.59E-11	RSU1	10:16872797	Interferon response/Anti-bacterial
C16orf80	16:58205340	0.104	0.0126	1.63E-16	RYR3	15:33967794	Unknown
C17orf49	17:6902179	0.185	0.0106	3.00E-66	S100A12	1:153376880	CD4+ T-Cells
C17orf62	17:80402045	0.045	0.00408	4.61E-28	S100A12	1:153362146	Interferon response/Anti-bacterial
C17orf75	17:30659006	0.0466	0.00546	1.81E-17	S100P	4:6697554	Interferon response/Anti-bacterial
C17orf77	17:72563686	-0.0406	0.00637	2.14E-10	S100PBP	1:33304596	Unknown
C17orf80	17:71217074	-0.0294	0.00383	1.95E-14	SAMD12	8:119666300	Interferon response/Anti-bacterial
C17orf97	17:259304	-0.158	0.00754	5.00E-94	SAMD3	6:130455734	Erythrocytes
C18orf25	18:43802778	0.0327	0.00514	2.05E-10	SAMD3	6:130419776	Neutrophils 2
C18orf32	18:47047929	-0.119	0.0193	7.22E-10	SCHIP1	3:159388148	Erythrocytes
C19orf10	19:4658254	0.0736	0.00991	1.24E-13	SCHIP1	3:159628463	Monocytes / Macrophages

C19orf12	19:30204855	-0.0748	0.00811	4.16E-20	SCHIP1	3:159663351	Neutrophils 1
C19orf33	19:38821697	0.0715	0.00401	4.82E-69	SCP2	1:53402552	Interferon response/Anti-bacterial
C19orf40	19:33468410	0.0744	0.00596	3.14E-35	SCP2	1:53392908	Unknown
C19orf47	19:40855872	-0.0189	0.00324	5.07E-09	SENP6	6:76304062	Interferon response/Anti-bacterial
C19orf54	19:41255500	-0.0317	0.00293	4.75E-27	SEPT10	2:110412315	Monocytes / Macrophages
C19orf60	19:18702497	-0.0542	0.00391	5.87E-43	SEPT2	2:242294913	Interferon response/Anti-bacterial
C19orf66	19:10237185	-0.0536	0.00777	6.09E-12	SEPT4	17:56549663	B-cells
C1GALT1	7:7244181	0.163	0.00619	8.43E-144	SEPT4	17:56549663	Neutrophils 1
C1orf101	1:244628341	-0.0178	0.003	2.98E-09	SEPT4	17:56549663	Unknown
C1orf131	1:231376806	0.0499	0.00648	1.50E-14	SERPINA1	14:94843455	Erythrocytes
C1orf174	1:3788499	-0.21	0.00854	2.27E-126	SETD9	5:56226824	Interferon response/Anti-bacterial
C1orf177	1:55274052	-0.0275	0.00401	8.29E-12	SFXN4	10:120923339	Interferon response/Anti-bacterial
C1orf198	1:231012288	-0.0581	0.00545	2.64E-26	SGPP2	2:223429784	Erythrocytes
C1orf220	1:178532010	-0.0815	0.00614	1.75E-39	SHKBP1	19:41091821	Interferon response/Anti-bacterial
C1orf27	1:186330315	-0.047	0.00367	5.44E-37	SIDT2	11:117068504	Interferon response/Anti-bacterial
C1orf54	1:150245082	0.231	0.0282	3.07E-16	SIRPB1	20:1599142	Interferon response/Anti-bacterial
C1orf85	1:156215899	0.156	0.00354	<1.0E-314	SIRT5	6:13574491	Interferon response/Anti-bacterial
C1orf87	1:60570185	-0.114	0.0135	4.96E-17	SLC12A1	15:48596713	Erythrocytes
C1QA	1:22968425	0.0488	0.00751	8.87E-11	SLC12A1	15:48596713	Interferon response/Anti-bacterial
C1QBP	17:5293080	-0.0299	0.00305	2.16E-22	SLC12A1	15:48606346	Monocytes / Macrophages
C1QTNF6	22:37573712	-0.0235	0.0031	3.69E-14	SLC12A1	15:48597514	Neutrophils 1
C1QTNF7	4:15494124	-0.118	0.00756	4.92E-54	SLC12A1	15:48596713	Neutrophils 10
C1RL-AS1	12:7263699	-0.0489	0.00605	8.18E-16	SLC12A1	15:48596713	NK cells / CD8+ T-Cells
C2	6:31851234	-0.0221	0.00363	1.08E-09	SLC12A1	15:48596713	Unknown
C20orf111	20:42832789	-0.143	0.00553	1.13E-139	SLC1A3	5:36641858	CD4+ T-Cells
C20orf112	20:31042169	-0.0265	0.00415	1.93E-10	SLC1A3	5:36641858	Unknown
C20orf196	20:5755860	0.0384	0.00515	1.09E-13	SLC23A2	20:4950311	Erythrocytes
C20orf197	20:58644272	0.0494	0.00359	2.51E-42	SLC23A2	20:4896214	Monocytes / Macrophages
C20orf24	20:35208051	0.0498	0.00843	3.53E-09	SLC25A24	1:108742123	Monocytes / Macrophages
C20orf72	20:17954706	0.136	0.00602	4.69E-108	SLC44A4	6:31864304	Interferon response/Anti-bacterial
C20orf96	20:262570	0.0658	0.00391	5.30E-62	SLC44A4	6:31847196	NK cells / CD8+ T-Cells
C21orf128	21:43529185	-0.03	0.0046	7.20E-11	SLC44A5	1:75933611	Interferon response/Anti-bacterial
C21orf2	21:45759355	-0.052	0.00756	6.76E-12	SLC4A7	3:27445270	Monocytes / Macrophages
C21orf33	21:45515366	-0.0254	0.00344	1.57E-13	SLFN5	17:33520263	Neutrophils 1
C21orf62	21:34146179	0.14	0.00976	4.54E-46	SLFN5	17:33565304	Neutrophils 2
C21orf67	21:46352040	-0.039	0.00389	1.74E-23	SMG5	1:156275281	Interferon response/Anti-bacterial
C21orf7	21:30530692	-0.0401	0.0057	2.19E-12	SMG5	1:156220056	Unknown
C21orf91	21:19169155	-0.0679	0.00467	4.91E-47	SMG7	1:183572649	B-cells
C22orf32	22:42429049	0.394	0.00553	<1.0E-314	SNRPN	15:25235326	Interferon response/Anti-bacterial

C22orf34	22:50018651	-0.156	0.00428	5.57E-259	SNX16	8:82748437	Interferon response/Anti-bacterial
C22orf43	22:23988257	0.145	0.00414	1.95E-240	SNX25	4:186199652	Erythrocytes
C2CD2	21:43326524	-0.0216	0.00342	2.84E-10	SNX25	4:186124959	Monocytes / Macrophages
C2CD3	11:73802736	0.0531	0.00393	7.51E-41	SNX6	14:35061728	Interferon response/Anti-bacterial
C2CD5	12:22697423	-0.183	0.00687	6.14E-147	SP140	2:231207684	Neutrophils 1
C2orf15	2:99764260	-0.0679	0.00596	9.39E-30	SPARCL1	4:88476673	B-cells
C2orf29	2:101917365	0.0224	0.0028	1.38E-15	SPARCL1	4:88476673	Neutrophils 2
C2orf42	2:70414485	0.0644	0.00833	1.31E-14	SPARCL1	4:88476673	Unknown
C2orf43	2:20888265	0.0481	0.00513	9.50E-21	SPATA20	17:48624523	Interferon response/Anti-bacterial
C2orf44	2:24319352	-0.0441	0.00626	2.13E-12	SPATA7	14:88907471	Interferon response/Anti-bacterial
C2orf49	2:105937633	-0.0419	0.00643	7.52E-11	SPATS2L	2:201135905	B-cells
C2orf61	2:47381097	-0.0828	0.01	1.62E-16	SPATS2L	2:201135905	Neutrophils 1
C2orf88	2:191091275	-0.0835	0.0104	9.04E-16	SPTLC2	14:78031773	Neutrophils 1
C3AR1	12:8206167	0.414	0.00741	<1.0E-314	STK32B	4:5399876	Erythrocytes
C3orf14	3:62305073	0.0841	0.00776	3.85E-27	STK32B	4:5399876	Monocytes / Macrophages
C3orf17	3:112753327	-0.15	0.0102	2.44E-48	STRBP	9:125916612	Monocytes / Macrophages
C3orf20	3:14712473	0.0319	0.00217	5.98E-48	STX3	11:59563481	Interferon response/Anti-bacterial
C3orf35	3:37441773	0.0207	0.00341	1.37E-09	SUPT20H	13:37609725	Interferon response/Anti-bacterial
C3orf49	3:63815326	-0.0963	0.0153	3.44E-10	SYCP2L	6:10886949	Interferon response/Anti-bacterial
C3orf58	3:143705727	-0.0472	0.00444	4.42E-26	SYCP2L	6:10887276	Monocytes / Macrophages
C3orf62	3:49305140	0.0401	0.00626	1.56E-10	SYCP2L	6:10887253	Neutrophils 10
C4B	6:31973863	0.0411	0.00216	2.30E-78	SYCP2L	6:10887276	NK cells / CD8+ T-Cells
C4BPA	1:207269205	-1.39	0.0321	<1.0E-314	SYCP2L	6:10893120	Unknown
C4BPB	1:207261196	-0.0459	0.00359	6.92E-37	SYT17	16:19290797	Erythrocytes
C4orf19	4:37640962	-0.0358	0.00466	1.94E-14	SYT17	16:19290797	Monocytes / Macrophages
C4orf21	4:113463265	-0.0768	0.00354	4.91E-100	TACSTD2	1:59048003	Interferon response/Anti-bacterial
C4orf27	4:170664777	-0.0792	0.01	2.80E-15	TANK	2:162007430	Interferon response/Anti-bacterial
C4orf3	4:120219238	-0.0847	0.00664	9.26E-37	TBC1D4	13:75842705	Monocytes / Macrophages
C4orf32	4:113119806	-0.0316	0.00305	5.48E-25	TBC1D7	6:13319402	Interferon response/Anti-bacterial
C4orf33	4:130037838	0.0651	0.00476	7.55E-42	TC2N	14:92290983	Erythrocytes
C4orf45	4:159972514	-0.0947	0.0076	3.60E-35	TC2N	14:92290983	Monocytes / Macrophages
C5	9:123769200	-0.0861	0.00352	3.56E-125	TCF4	18:53011777	Erythrocytes
C5AR1	19:47787587	-0.122	0.0172	1.31E-12	TCF4	18:53011777	Monocytes / Macrophages
C5orf22	5:31524477	0.0696	0.00571	1.08E-33	TCF4	18:53289421	Neutrophils 1
C5orf34	5:43542511	0.0231	0.00321	7.40E-13	TDRD9	14:104534574	Erythrocytes
C5orf4	5:154200612	-0.192	0.00866	1.39E-103	TFG	3:100428460	Interferon response/Anti-bacterial
C5orf44	5:64893443	0.0384	0.00432	7.79E-19	THEMIS2	1:28207887	Interferon response/Anti-bacterial
C5orf45	5:179276913	-0.0249	0.00379	5.53E-11	TIAM1	21:32856175	Monocytes / Macrophages
C5orf58	5:169666462	0.0394	0.00488	8.92E-16	TIMM10	11:57311754	B-cells

C6orf1	6:34234953	-0.0476	0.00665	9.41E-13	TIMM10	11:57339950	Interferon response/Anti-bacterial
C6orf10	6:32331002	0.125	0.00772	8.60E-58	TIMM10	11:57347525	Neutrophils 1
C6orf106	6:34603691	-0.0456	0.0052	2.52E-18	TM6SF1	15:83819409	Erythrocytes
C6orf170	6:121655392	0.0712	0.00643	3.06E-28	TMEM19	12:72140391	Erythrocytes
C6orf201	6:4121333	0.0305	0.0037	1.81E-16	TMEM258	11:61560081	Interferon response/Anti-bacterial
C6orf211	6:151829847	-0.109	0.0103	3.29E-26	TNIK	3:171065575	Monocytes / Macrophages
C6orf25	6:31672242	0.1	0.0136	2.19E-13	TNIK	3:170981811	Neutrophils 1
C6orf48	6:31841010	-0.292	0.0254	2.17E-30	TOR1B	9:132551788	Interferon response/Anti-bacterial
C6orf57	6:71289189	0.0404	0.00549	2.07E-13	TPM2	9:35680337	Interferon response/Anti-bacterial
C6orf62	6:24713723	-0.0467	0.0054	7.47E-18	TPR	1:186369973	B-cells
C7orf10	7:40328119	-0.0313	0.00517	1.47E-09	TRAK1	3:42199095	Erythrocytes
C7orf13	7:156445785	0.0725	0.00622	5.44E-31	TRAM2	6:52333130	Erythrocytes
C7orf25	7:42942126	-0.194	0.0102	7.78E-78	TRAT1	3:108512303	Monocytes / Macrophages
C7orf26	7:6672079	0.103	0.0146	1.43E-12	TREML4	6:41195147	Interferon response/Anti-bacterial
C7orf31	7:25220533	-0.022	0.00363	1.31E-09	TRIB2	2:12863770	Monocytes / Macrophages
C7orf34	7:142624333	0.361	0.0224	5.10E-57	TRIM37	17:57129882	Interferon response/Anti-bacterial
C7orf41	7:30230870	0.0771	0.00938	2.56E-16	TRPC6	11:101398450	Neutrophils 10
C7orf43	7:99744174	0.0362	0.00494	2.65E-13	TSGA10	2:99724512	Interferon response/Anti-bacterial
C7orf49	7:134831048	-0.106	0.00485	1.24E-100	TSHZ2	20:51636715	Monocytes / Macrophages
C7orf50	7:1029585	-0.0194	0.00252	1.79E-14	TSPAN5	4:99568001	NK cells / CD8+ T-Cells
C7orf53	7:112095622	-0.0761	0.00742	1.99E-24	TTC39C	18:21621280	Monocytes / Macrophages
C7orf55	7:139018957	0.0237	0.00397	2.61E-09	TUBB2A	6:3158460	Interferon response/Anti-bacterial
C7orf60	7:112579251	0.0813	0.0107	3.01E-14	TUBB2A	6:3147432	NK cells / CD8+ T-Cells
C7orf61	7:100099466	-0.0381	0.00617	6.68E-10	TXK	4:48061275	Erythrocytes
C8orf31	8:144161293	-0.0942	0.00611	2.11E-52	TXK	4:48061275	Monocytes / Macrophages
C8orf40	8:42418445	-0.156	0.00563	1.85E-158	TXK	4:48061275	Neutrophils 1
C8orf44	8:67583681	-0.0601	0.0102	4.65E-09	TXNL4A	18:77749211	Interferon response/Anti-bacterial
C8orf46	8:67378739	-0.032	0.00339	6.72E-21	UBASH3B	11:122644031	Monocytes / Macrophages
C8orf59	8:86130628	-0.145	0.00566	2.01E-136	UBASH3B	11:122644031	Neutrophils 1
C9orf123	9:7793622	-0.0704	0.0104	1.74E-11	UBE2D1	10:60144207	Interferon response/Anti-bacterial
C9orf131	9:34997569	-0.034	0.00341	3.93E-23	UGDH	4:39526467	Interferon response/Anti-bacterial
C9orf156	9:100663700	-0.08	0.0036	1.92E-104	UHRF1BP1	6:34799221	Interferon response/Anti-bacterial
C9orf3	9:97638926	-0.0982	0.00467	2.12E-94	USP28	11:113716123	Erythrocytes
C9orf41	9:77566246	0.0326	0.00432	4.76E-14	USP34	2:61404415	Monocytes / Macrophages
C9orf47	9:91566754	-0.0274	0.0044	5.08E-10	USP53	4:120131644	Interferon response/Anti-bacterial
C9orf64	9:86549939	0.0659	0.00521	4.52E-36	USP6	17:5016068	B-cells
C9orf66	9:215511	0.0735	0.00731	1.42E-23	UTS2	1:7962157	Interferon response/Anti-bacterial
C9orf72	9:27572255	0.127	0.0052	4.81E-124	UTS2	1:7961294	Monocytes / Macrophages
C9orf78	9:132549493	0.462	0.00969	<1.0E-314	UTS2	1:7960476	NK cells / CD8+ T-Cells

C9orf84	9:114435288	-0.127	0.00651	2.09E-81
C9orf85	9:74569379	0.0724	0.0105	5.23E-12
C9orf89	9:95864147	0.0526	0.00437	6.97E-33
C9orf91	9:117405645	0.0395	0.00483	3.55E-16
CA13	8:86135096	0.0544	0.00456	2.14E-32
CA2	8:86387530	-0.118	0.00878	1.43E-40
CA6	1:9000220	-0.126	0.0123	3.00E-24
CA8	8:61193588	-0.119	0.00553	4.11E-98
CAB39	2:231596072	0.0435	0.00415	1.79E-25
CAB39L	13:50019376	-0.0384	0.00491	6.17E-15
CABIN1	22:24580485	-0.0334	0.00374	4.71E-19
CABP5	19:48527902	-0.352	0.0346	4.58E-24
CACHD1	1:65016399	0.0438	0.00669	6.49E-11
CACNA1D	3:53730735	0.0195	0.00248	3.80E-15
CACNA1E	1:181467205	0.046	0.00473	3.45E-22
CACNB2	10:18868840	0.0273	0.00342	1.69E-15
CACNB3	12:49195780	-0.0305	0.00435	2.61E-12
CACNB4	2:152971003	-0.104	0.0115	2.37E-19
CACUL1	10:120485266	-0.0276	0.003	5.44E-20
CACYBP	1:175015808	0.0411	0.00509	7.71E-16
CALB1	8:91064455	0.0972	0.00524	1.79E-74
CALCOCO1	12:54112381	0.0186	0.00317	4.28E-09
CALCRL	2:188293636	0.084	0.00651	1.60E-37
CALHM2	10:105231314	0.0971	0.00779	3.58E-35
CALM3	19:47081668	-0.0463	0.00502	4.15E-20
CALML4	15:68537929	-0.0507	0.00447	2.06E-29
CALU	7:128424406	0.0487	0.00631	1.31E-14
CAMK1D	10:12343629	0.116	0.00932	3.07E-35
CAMK2D	4:114719328	0.0487	0.00434	7.36E-29
CAMK2N1	1:20834610	0.0741	0.0102	3.45E-13
CAMK4	5:110566360	0.179	0.0186	1.21E-21
CAMKK2	12:121681536	-0.0369	0.00355	4.56E-25
CAMKMT	2:44666570	0.0731	0.00732	2.96E-23
CAMSAP1	9:138684454	-0.035	0.00553	2.62E-10
CAMSAP2	1:200708025	0.0561	0.0052	7.10E-27
CAMTA1	1:7809383	-0.0737	0.0062	4.04E-32
CAMTA2	17:4897042	-0.0698	0.00364	1.64E-79
CANT1	17:76994843	0.0565	0.00352	1.06E-56
CANX	5:179177544	-0.0496	0.00698	1.39E-12

VAMP8	2:85808573	Interferon response/Anti-bacterial
VASH1	14:77182302	Interferon response/Anti-bacterial
VIM	10:17270912	B-cells
VNN1	6:133031252	Interferon response/Anti-bacterial
VPS53	17:446554	Interferon response/Anti-bacterial
WDFY3	4:85881832	Erythrocytes
WLS	1:68736352	Interferon response/Anti-bacterial
WRB	21:40770885	Interferon response/Anti-bacterial
XKR3	22:17245877	Erythrocytes
XKR3	22:17265194	Interferon response/Anti-bacterial
XKR3	22:17245877	Neutrophils 1
XRRA1	11:74574998	B-cells
XRRA1	11:74659789	Interferon response/Anti-bacterial
YEATS4	12:69776886	Interferon response/Anti-bacterial
ZADH2	18:72920065	Interferon response/Anti-bacterial
ZC2HC1A	8:79558153	Interferon response/Anti-bacterial
ZNF138	7:64291976	Interferon response/Anti-bacterial
ZNF155	19:44488352	Interferon response/Anti-bacterial
ZNF286A	17:15611343	Interferon response/Anti-bacterial
ZNF354A	5:178099833	Interferon response/Anti-bacterial
ZNF429	19:21770143	Interferon response/Anti-bacterial
ZNF718	4:182845	Interferon response/Anti-bacterial
XRRA1	11:74659789	Interferon response/Anti-bacterial
YEATS4	12:69776886	Interferon response/Anti-bacterial
ZADH2	18:72920065	Interferon response/Anti-bacterial
ZC2HC1A	8:79558153	Interferon response/Anti-bacterial
ZNF138	7:64291976	Interferon response/Anti-bacterial
ZNF155	19:44488352	Interferon response/Anti-bacterial
ZNF286A	17:15611343	Interferon response/Anti-bacterial
ZNF354A	5:178099833	Interferon response/Anti-bacterial
ZNF429	19:21770143	Interferon response/Anti-bacterial
ZNF718	4:182845	Interferon response/Anti-bacterial

CAP1	1:40545964	-0.0403	0.00426	4.59E-21
CAPG	2:85628983	0.119	0.00602	2.43E-83
CAPN1	11:64944965	-0.0558	0.00295	4.08E-77
CAPN3	15:42690901	-0.193	0.022	2.11E-18
CAPN7	3:15295364	-0.0454	0.00383	5.36E-32
CAPRIN2	12:30907594	0.0688	0.0075	6.56E-20
CAPS2	12:75771980	-0.0382	0.00425	2.95E-19
CAPZA1	1:113191184	-0.0607	0.00495	4.81E-34
CAPZA2	7:116569136	0.0884	0.00606	2.72E-47
CAPZB	1:19672376	0.034	0.00415	3.05E-16
CARD11	7:3131792	-0.0249	0.0035	1.21E-12
CARD8	19:48762257	-0.0637	0.00386	1.27E-59
CARD9	9:139301960	-0.0411	0.00517	2.18E-15
CARHSP1	16:8929919	0.144	0.00933	5.71E-53
CARKD	13:111268196	0.0883	0.00324	2.66E-153
CARS2	13:111297448	0.0686	0.00344	2.95E-85
CASC1	12:25258073	-0.138	0.00786	2.53E-67
CASC3	17:38282624	0.0563	0.00886	2.22E-10
CASD1	7:94136931	0.0525	0.00707	1.28E-13
CASP1	11:104949552	-0.132	0.0115	5.61E-30
CASP10	2:202036292	-0.0263	0.00415	2.62E-10
CASP2	7:142948773	-0.124	0.0187	3.72E-11
CASP3	4:185591804	0.108	0.00701	1.32E-52
CASP5	11:104931537	-0.296	0.0199	4.46E-49
CASP6	4:110592090	-0.0659	0.00569	1.02E-30
CASP7	10:115439640	-0.0716	0.00468	8.00E-52
CASP8	2:202164837	0.0279	0.00315	1.06E-18
CASP8AP2	6:90590112	0.0307	0.00375	3.15E-16
CASP9	1:15817090	-0.0255	0.00374	1.05E-11
CASQ1	1:160136208	-0.0852	0.0053	8.75E-57
CASS4	20:55045843	0.0398	0.00465	1.50E-17
CAST	5:95995733	-0.116	0.00579	1.88E-86
CAT	11:34442997	-0.256	0.00637	<1.0E-314
CATSPER2	15:43920964	0.142	0.0117	3.08E-33
CATSPERB	14:92232971	-0.36	0.0127	1.39E-164
CATSPERG	19:38834377	-0.0944	0.00555	3.69E-63
CBL	11:119152684	0.0842	0.00745	2.91E-29
CBLB	3:105388590	0.0347	0.00582	2.59E-09
CBLL1	7:107409810	0.0317	0.00458	4.79E-12

CBLN3	14:24898751	0.102	0.00391	9.96E-142
CBR1	21:37435576	0.366	0.0196	1.87E-75
CBR3	21:37489890	0.237	0.0076	1.27E-195
CBR4	4:169909593	-0.0651	0.00649	1.68E-23
CBS	21:44473062	-0.0497	0.00209	2.31E-118
CBX1	17:46141276	0.0593	0.00504	1.40E-31
CBX3	7:26191192	0.0943	0.0102	3.07E-20
CBX5	12:54655470	-0.0531	0.00564	6.49E-21
CC2D1B	1:52843747	-0.0458	0.0055	1.03E-16
CC2D2B	10:97812812	-0.227	0.0204	1.51E-28
CCAR1	10:70483838	-0.0381	0.0046	1.65E-16
CCBL2	1:89440977	0.0649	0.00338	2.73E-79
CCDC101	16:28575870	0.101	0.00553	2.67E-72
CCDC107	9:35657063	-0.02	0.00322	6.20E-10
CCDC109B	4:110590479	0.0379	0.00478	2.58E-15
CCDC111	4:185571938	-0.0577	0.00589	1.71E-22
CCDC117	22:29225500	-0.0454	0.00386	1.74E-31
CCDC12	3:46979013	-0.0363	0.00362	1.86E-23
CCDC122	13:44434367	-0.114	0.00466	4.36E-125
CCDC124	19:18054643	-0.0571	0.00713	1.46E-15
CCDC125	5:68624793	0.203	0.00529	7.21E-284
CCDC126	7:23634985	-0.122	0.00654	1.66E-75
CCDC127	5:256472	0.163	0.0116	1.99E-44
CCDC13	3:42801920	-0.0313	0.00277	2.38E-29
CCDC130	19:13860145	0.0199	0.00312	1.87E-10
CCDC132	7:92894727	0.067	0.00844	2.52E-15
CCDC134	22:42198093	0.0281	0.00377	1.05E-13
CCDC135	16:57722576	-0.0259	0.00295	1.89E-18
CCDC137	17:79636842	0.0415	0.00366	1.85E-29
CCDC141	2:179909458	-0.0626	0.00525	2.45E-32
CCDC144B	17:18573285	-0.682	0.0543	1.27E-35
CCDC146	7:76767073	0.667	0.0157	<1.0E-314
CCDC149	4:24901114	-0.0487	0.00553	1.73E-18
CCDC15	11:124847675	-0.196	0.00642	1.59E-188
CCDC169	13:36846472	0.0962	0.00572	5.90E-62
CCDC170	6:151816537	-0.124	0.0101	6.50E-34
CCDC171	9:15553128	-0.15	0.00635	6.79E-117
CCDC176	14:74577697	-0.112	0.0173	8.78E-11
CCDC18	1:93791098	-0.112	0.0149	6.76E-14

CCDC19	1:159892088	0.0426	0.00563	4.27E-14
CCDC23	1:43230112	-0.832	0.0162	<1.0E-314
CCDC25	8:27623295	-0.0506	0.00505	2.19E-23
CCDC28A	6:139097820	-0.0741	0.00525	2.00E-44
CCDC3	10:13084866	-0.0449	0.00523	1.20E-17
CCDC30	1:42951330	0.0591	0.00552	1.68E-26
CCDC41	12:94853731	0.0716	0.00721	4.86E-23
CCDC50	3:191045874	-0.05	0.00595	5.60E-17
CCDC53	12:102399354	0.106	0.00613	4.88E-65
CCDC57	17:80091441	-0.0344	0.0033	3.36E-25
CCDC6	10:61549818	-0.0283	0.00394	7.16E-13
CCDC62	12:123275609	0.0742	0.00914	6.13E-16
CCDC64	12:120482878	-0.0448	0.00608	2.02E-13
CCDC65	12:49353027	0.0253	0.0039	1.07E-10
CCDC68	18:52637450	0.0367	0.005	2.26E-13
CCDC69	5:150618993	0.0897	0.00549	1.19E-58
CCDC75	2:37325108	0.0437	0.00602	4.43E-13
CCDC77	12:518986	0.119	0.00676	8.65E-68
CCDC80	3:112295907	-0.0282	0.00456	6.50E-10
CCDC81	11:86067375	-0.0282	0.00385	2.62E-13
CCDC82	11:96115744	-0.243	0.00627	1.64E-289
CCDC88A	2:55644201	-0.0748	0.00827	2.12E-19
CCDC9	19:47715831	-0.0374	0.0038	1.20E-22
CCDC90A	6:13845712	0.0575	0.00499	2.30E-30
CCDC91	12:28611779	0.0544	0.00516	9.84E-26
CCDC92	12:124440743	0.0473	0.00559	3.48E-17
CCDC93	2:118670137	0.0575	0.00702	3.49E-16
CCDC94	19:4236388	0.0951	0.00393	1.98E-122
CCL20	2:228672579	0.0665	0.00637	3.22E-25
CCL4	17:34431403	-0.142	0.0132	1.35E-26
CCL5	17:34221702	0.105	0.0101	6.74E-25
CCM2	7:45067267	-0.0419	0.00302	4.56E-43
CCNA1	13:37025916	-0.0392	0.00495	2.95E-15
CCNB1	5:68488815	-0.0332	0.00466	1.14E-12
CCNB1IP1	14:20806659	0.0564	0.00497	2.06E-29
CCNE1	19:30322427	-0.0266	0.00368	5.41E-13
CCNH	5:86735368	0.0311	0.00437	1.34E-12
CCNJ	10:97850096	0.0303	0.00483	3.91E-10
CCNJL	5:159741390	0.0582	0.00861	1.55E-11

CCNL1	3:156896364	-0.0503	0.00318	3.92E-55
CCNT1	12:49036033	0.0267	0.00432	6.40E-10
CCNT2	2:135682094	-0.04	0.00319	1.16E-35
CCNY	10:35771615	0.0547	0.00344	1.49E-55
CCPG1	15:55697964	-0.0857	0.00827	6.68E-25
CCR1	3:46205686	-0.218	0.00959	1.36E-109
CCR2	3:46374273	-0.119	0.0103	2.17E-30
CCR3	3:46289113	-0.334	0.00811	<1.0E-314
CCR4	3:33029986	0.0742	0.00872	2.17E-17
CCR6	6:167533062	0.0503	0.00684	2.21E-13
CCS	11:66348503	-0.0538	0.00402	3.35E-40
CCSAP	1:229408995	-0.121	0.003	<1.0E-314
CCSER2	10:86257341	0.181	0.00498	3.75E-259
CCT2	12:69985452	0.0458	0.00423	5.37E-27
CCT3	1:156274991	0.0292	0.00374	6.43E-15
CCT4	2:62057548	-0.0409	0.00445	5.93E-20
CCT5	5:10261434	0.0311	0.00504	7.63E-10
CCT6A	7:56126360	-0.128	0.00422	5.22E-185
CCT6B	17:33330150	-0.0314	0.00402	6.81E-15
CCT7	2:73470615	0.0638	0.00702	1.46E-19
CCT8	21:30445948	-0.0844	0.00568	6.04E-49
CD101	1:117567048	0.132	0.00619	1.23E-96
CD109	6:74445387	0.0197	0.00226	3.96E-18
CD14	5:139978241	0.061	0.00741	2.27E-16
CD151	11:823809	0.121	0.00605	3.14E-86
CD160	1:145703115	-0.0631	0.0071	8.71E-19
CD163L1	12:7622509	0.0235	0.00263	6.84E-19
CD177	19:43852065	0.138	0.0145	2.36E-21
CD180	5:66492725	0.0864	0.0102	2.58E-17
CD1A	1:158226667	0.0564	0.00852	3.92E-11
CD1E	1:158363687	-0.156	0.026	2.40E-09
CD200R1	3:112654447	-0.133	0.00574	3.46E-113
CD200R1L	3:112521519	0.0548	0.00518	6.52E-26
CD209	19:7783150	0.109	0.016	1.43E-11
CD22	19:35837705	-0.204	0.0171	3.58E-32
CD226	18:67519174	-0.0477	0.00668	1.06E-12
CD244	1:160840185	-0.0354	0.00512	4.93E-12
CD247	1:167418468	-0.0535	0.00493	3.71E-27
CD274	9:5466085	-0.152	0.0146	4.47E-25

CD28	2:204592021	-0.0657	0.00695	4.38E-21
CD2AP	6:47480676	-0.146	0.00659	1.36E-104
CD300A	17:72469958	-0.105	0.00521	7.05E-88
CD300C	17:72532568	0.0623	0.00494	5.93E-36
CD300E	17:72653890	0.0688	0.00695	6.46E-23
CD300LB	17:72498108	-0.297	0.021	2.00E-44
CD300LD	17:72564119	-0.338	0.0101	1.76E-221
CD300LF	17:72715382	0.0422	0.00518	4.24E-16
CD33	19:51724326	0.0375	0.00468	1.42E-15
CD36	7:80318032	0.132	0.0174	4.77E-14
CD37	19:49845895	0.0678	0.00574	7.68E-32
CD38	4:15738483	-0.0452	0.00557	6.24E-16
CD3E	11:118176898	-0.0412	0.00692	2.84E-09
CD3G	11:118230739	0.0813	0.00888	7.92E-20
CD4	12:6894164	-0.048	0.00586	3.32E-16
CD40	20:44747086	-0.0505	0.00441	4.53E-30
CD44	11:35245287	0.0257	0.00317	7.32E-16
CD47	3:107807485	-0.0311	0.00306	5.11E-24
CD48	1:160654786	0.145	0.00516	1.60E-161
CD5	11:60858179	-0.0549	0.00762	6.65E-13
CD52	1:26666068	0.0609	0.00777	5.53E-15
CD55	1:207444928	0.175	0.00402	<1.0E-314
CD59	11:33755355	-0.107	0.00515	7.71E-93
CD6	11:60793701	0.0423	0.00607	3.93E-12
CD69	12:9909286	0.139	0.0165	3.39E-17
CD72	9:35602420	0.0466	0.00696	2.42E-11
CD79B	17:62010232	0.0393	0.00463	2.92E-17
CD83	6:14089043	0.0782	0.00845	3.04E-20
CD84	1:160548247	-0.125	0.00777	3.76E-57
CD8A	2:87009742	0.0352	0.00557	2.90E-10
CD9	12:6280584	-0.13	0.0114	4.36E-30
CD93	20:23101571	0.36	0.00906	7.89E-302
CD96	3:111249002	-0.0458	0.00492	1.65E-20
CDA	1:20958024	-0.263	0.00849	2.08E-193
CDADC1	13:49848833	0.032	0.00425	5.96E-14
CDC123	10:12309268	-0.0508	0.00521	2.47E-22
CDC14A	1:100853755	0.0969	0.00355	1.28E-153
CDC16	13:114963436	-0.206	0.00507	<1.0E-314
CDC25B	20:3788479	-0.036	0.00358	1.35E-23

CDC26	9:116031822	0.0613	0.00888	5.69E-12
CDC42	1:22345647	-0.042	0.0046	8.52E-20
CDC42BPA	1:227453739	0.0697	0.00958	3.93E-13
CDC42BPB	14:103438905	0.0283	0.00276	1.72E-24
CDC42EP1	22:37960825	0.0474	0.00486	2.72E-22
CDC42EP3	2:37877131	0.0888	0.0138	1.33E-10
CDC42SE1	1:151063940	-0.0306	0.00447	8.92E-12
CDC5L	6:44356303	0.0747	0.00885	4.38E-17
CDC7	1:91966406	0.0765	0.00715	1.85E-26
CDCA4	14:105498583	-0.04	0.00574	3.58E-12
CDCA7L	7:21966979	0.132	0.00798	5.00E-60
CDCP1	3:45187671	-0.0178	0.00283	3.23E-10
CDH1	16:68800849	-0.0644	0.0054	2.02E-32
CDH2	18:25726723	0.0968	0.00467	6.19E-92
CDH23	10:73387404	-0.0436	0.00173	4.39E-133
CDHR1	10:85946204	-0.0195	0.0031	3.79E-10
CDHR3	7:105593511	-0.0192	0.00272	1.84E-12
CDIPT	16:29869176	-0.0387	0.00619	4.54E-10
CDK10	16:89708267	0.156	0.0115	1.27E-41
CDK15	2:202687170	-0.107	0.0113	5.32E-21
CDK17	12:96688389	0.0537	0.00419	4.98E-37
CDK19	6:110955566	-0.0601	0.00836	7.43E-13
CDK2AP1	12:123736084	-0.265	0.00724	9.60E-261
CDK5R1	17:30790378	-0.0406	0.00337	7.79E-33
CDK5RAP3	17:46042494	0.161	0.0182	1.57E-18
CDK7	5:68574032	0.081	0.00508	6.06E-56
CDK8	13:26869939	-0.167	0.0118	3.50E-44
CDKL1	14:50811025	0.078	0.00297	2.64E-143
CDKN1B	12:12890155	-0.0385	0.00635	1.39E-09
CDKN2AIP	4:184363887	0.0556	0.00382	3.80E-47
CDKN2B	9:22034267	0.029	0.00416	3.61E-12
CDKN3	14:54864797	0.0784	0.00702	1.21E-28
CDON	11:125914929	-0.0346	0.00404	1.65E-17
CDS2	20:5118897	0.185	0.00414	<1.0E-314
CDV3	3:133316038	0.0353	0.00481	2.67E-13
CEACAM21	19:42092815	0.114	0.00556	1.48E-89
CEACAM4	19:42127907	0.0944	0.0082	2.69E-30
CEBPZ	2:37459670	0.0554	0.00531	3.07E-25
CECR1	22:17700284	0.0554	0.0057	3.97E-22

CECR6	22:17598158	0.0207	0.00327	3.01E-10
CELA1	12:51723598	-0.0944	0.00484	8.37E-82
CENPBD1	16:90049307	-0.0704	0.0069	3.67E-24
CENPC1	4:68400065	-0.133	0.00519	5.69E-137
CENPK	5:64768677	-0.603	0.0107	<1.0E-314
CENPP	9:95117419	-0.148	0.0057	5.34E-141
CENPQ	6:49436199	-0.0707	0.00499	7.68E-45
CENPV	17:16225506	-0.0813	0.00702	1.28E-30
CEP104	1:3756532	-0.0831	0.00321	2.15E-139
CEP112	17:64175223	-0.029	0.00425	9.94E-12
CEP120	5:122761313	-0.0602	0.00348	3.67E-65
CEP128	14:81413039	-0.101	0.00573	4.99E-67
CEP164	11:117193619	-0.0157	0.002	4.88E-15
CEP170	1:243439386	-0.0678	0.0066	1.62E-24
CEP19	3:196438428	0.193	0.00774	1.85E-129
CEP192	18:12945310	-0.164	0.00318	<1.0E-314
CEP350	1:179927135	0.0319	0.00492	9.80E-11
CEP44	4:175213931	0.0548	0.00379	1.57E-46
CEP57	11:95553079	-0.0559	0.00461	1.89E-33
CEP57L1	6:109442109	0.0416	0.00698	2.75E-09
CEP63	3:134198419	0.102	0.00466	3.10E-102
CEP70	3:138278329	-0.0981	0.0132	1.47E-13
CEP85	1:26559306	-0.0777	0.00425	1.67E-72
CEP85L	6:119080379	0.0551	0.00512	1.15E-26
CEP89	19:33454535	0.0592	0.00449	4.03E-39
CEP95	17:62502435	-0.087	0.00859	6.70E-24
CEP97	3:101441900	-0.0696	0.00426	9.77E-59
CEPT1	1:111694789	-0.0359	0.00541	3.50E-11
CERK	22:47135272	-0.055	0.00346	1.46E-55
CERKL	2:182399486	0.105	0.00617	6.29E-63
CERS3	15:101114805	-0.0635	0.00388	9.87E-59
CERS4	19:8324825	0.067	0.0095	1.99E-12
CERS5	12:50541726	-0.0418	0.00377	2.59E-28
CERS6	2:169280713	-0.04	0.00452	1.07E-18
CETN3	5:89728310	0.0405	0.00603	2.08E-11
CFD	19:871457	-1.25	0.0286	<1.0E-314
CFDP1	16:75420750	-0.0306	0.00462	3.66E-11
CFH	1:196725939	-0.0636	0.0102	4.99E-10
CFL1	11:65624562	-0.0428	0.00392	1.81E-27

CFL2	14:35184228	-0.27	0.00743	8.75E-258
CFLAR	2:201982693	0.0573	0.00535	1.58E-26
CGGBP1	3:88107179	-0.162	0.00723	1.08E-105
CHAF1A	19:4393294	0.0473	0.00347	1.32E-41
CHAF1B	21:37773867	-0.0848	0.0139	9.78E-10
CHCHD2	7:56132741	-0.146	0.00721	1.73E-87
CHCHD5	2:113332271	-0.0705	0.00887	2.34E-15
CHD1L	1:146694247	-0.0335	0.00342	1.79E-22
CHD9	16:53327418	-0.0359	0.00466	1.44E-14
CHI3L1	1:203155882	0.569	0.012	<1.0E-314
CHI3L2	1:111765671	-0.212	0.00895	3.93E-118
CHID1	11:918793	0.133	0.0161	2.21E-16
CHKA	11:67840155	0.0316	0.0049	1.25E-10
CHKB-CPT1B	22:51021599	-0.0762	0.00333	2.19E-110
CHL1	3:283808	0.0149	0.00234	2.08E-10
CHML	1:241787568	0.0365	0.00616	3.41E-09
CHMP1A	16:89738276	0.0283	0.00351	9.50E-16
CHMP2B	3:87321757	0.0314	0.00417	5.64E-14
CHMP3	2:86816501	-0.0266	0.004	3.33E-11
CHMP4A	14:24654489	-0.0353	0.00418	3.65E-17
CHMP4B	20:32409142	-0.0888	0.00485	1.19E-72
CHMP7	8:23118503	0.0488	0.00441	3.28E-28
CHN1	2:175707817	0.0917	0.00549	3.79E-61
CHN2	7:29186576	0.141	0.00465	7.22E-186
CHP1	15:41530359	0.0512	0.00495	6.94E-25
CHPF2	7:150926775	0.0423	0.00361	2.50E-31
CHPT1	12:102091379	-0.163	0.00698	1.63E-114
CHRNA3	15:78836723	-0.0198	0.00265	9.50E-14
CHRNA5	15:78843051	-0.108	0.00334	2.66E-208
CHRNB2	1:154575613	-0.0483	0.0044	1.15E-27
CHRNE	17:4811761	-0.0403	0.00533	5.08E-14
CHST11	12:104981594	-0.059	0.0036	8.10E-59
CHSY1	15:101716797	0.163	0.00578	2.49E-162
CHURC1	14:65370389	0.114	0.00539	1.15E-94
CIAO1	2:96936254	-0.021	0.00293	8.44E-13
CIB1	15:90776375	0.142	0.00449	8.47E-201
CIITA	16:10961882	-0.0334	0.00565	3.53E-09
CIR1	2:175261148	-0.0459	0.00768	2.39E-09
CIRH1A	16:69202908	-0.0863	0.0147	4.95E-09

CISD1	10:60054487	0.243	0.00761	2.06E-205
CISD2	4:103760250	0.0692	0.0116	2.27E-09
CKAP2	13:53040822	0.144	0.00482	1.51E-180
CKAP2L	2:113486941	0.0228	0.00375	1.24E-09
CKAP4	12:106618665	0.143	0.02	9.15E-13
CKMT2	5:80590921	0.121	0.00535	7.84E-109
CKS2	9:91963682	0.19	0.0182	2.42E-25
CLASP1	2:122225284	-0.0394	0.00591	2.87E-11
CLASP2	3:33723538	0.0401	0.00462	4.80E-18
CLCA3P	1:87091891	-0.0586	0.00457	3.86E-37
CLCC1	1:109483975	-0.0358	0.00495	5.46E-13
CLCN6	1:11865816	-0.111	0.00505	1.73E-102
CLCN7	16:1511758	0.0378	0.00314	5.20E-33
CLDN20	6:155551221	0.0982	0.00734	3.29E-40
CLDN23	8:8563198	-0.0286	0.0038	6.01E-14
CLDN5	22:19492200	-0.0215	0.00345	5.47E-10
CLDND1	3:98245164	-0.0489	0.00686	1.21E-12
CLEC10A	17:7018222	-0.0478	0.00507	6.79E-21
CLEC12A	12:10060125	-0.765	0.0186	<1.0E-314
CLEC12B	12:10113258	-0.635	0.00776	<1.0E-314
CLEC1A	12:10202685	-0.043	0.00475	2.11E-19
CLEC1B	12:10109584	-0.406	0.00971	<1.0E-314
CLEC2A	12:10096086	-0.247	0.0111	4.28E-105
CLEC2B	12:10007863	-0.0695	0.00574	2.79E-33
CLEC2D	12:9869271	0.0447	0.00469	2.59E-21
CLEC3B	3:45081629	0.0377	0.00367	1.66E-24
CLEC4A	12:8280755	-0.0815	0.00566	3.93E-46
CLEC4C	12:7878685	-0.45	0.00712	<1.0E-314
CLEC4D	12:8668281	-0.345	0.0106	1.21E-209
CLEC4E	12:8697920	-0.243	0.0107	2.21E-109
CLEC4F	2:71049117	-0.195	0.00364	<1.0E-314
CLEC4G	19:7778963	0.0243	0.00356	9.29E-12
CLEC5A	7:141577186	-0.114	0.0129	1.36E-18
CLEC6A	12:8605829	0.504	0.0132	2.98E-282
CLEC7A	12:10222786	-0.166	0.0078	4.76E-96
CLEC9A	12:10202685	-0.326	0.00941	9.73E-238
CLECL1	12:9881125	0.199	0.00736	2.70E-151
CLHC1	2:55358675	-0.403	0.00962	<1.0E-314
CLIC4	1:25054223	-0.0758	0.00678	1.15E-28

CLINT1	5:157210977	-0.0287	0.00478	2.13E-09
CLIP4	2:29356669	-0.0564	0.00378	2.01E-49
CLK4	5:178094484	0.146	0.00458	2.00E-203
CLMN	14:95789261	0.0687	0.00605	1.60E-29
CLN5	13:77542896	0.0323	0.00466	4.89E-12
CLN6	15:68526355	-0.113	0.00333	1.68E-229
CLN8	8:1709972	0.0575	0.00466	1.32E-34
CLNS1A	11:77327368	0.14	0.0196	9.64E-13
CLOCK	4:56317656	-0.0545	0.00331	3.14E-59
CLPB	11:72124325	0.0385	0.00633	1.31E-09
CLPTM1	19:45487178	0.0538	0.00304	5.58E-68
CLRN1	3:150609382	-0.0993	0.00615	3.01E-57
CLSTN1	1:9829664	0.127	0.0167	3.03E-14
CLSTN3	12:7283646	0.0604	0.00432	1.58E-43
CLTA	9:36194569	0.0433	0.00659	5.36E-11
CLTB	5:175788920	-0.075	0.00484	6.65E-53
CLTCL1	22:19154522	-0.0355	0.00289	3.18E-34
CLU	8:27454682	-0.0591	0.00995	3.15E-09
CLUAP1	16:3565123	-0.0364	0.0059	7.42E-10
CLUHP3	16:31697235	-0.124	0.00346	7.07E-251
CLYBL	13:100310215	0.0568	0.00795	1.03E-12
CMAHP	6:25098265	-0.13	0.00478	7.00E-153
CMAS	12:22243661	-0.0563	0.00644	3.01E-18
CMBL	5:10286742	-0.188	0.00549	3.78E-233
CMC2	16:81028511	-0.0716	0.00646	3.38E-28
CMIP	16:81735012	0.0493	0.00673	2.76E-13
CMKLR1	12:108721636	-0.115	0.00643	1.32E-69
CMPK1	1:47752600	0.0236	0.00394	2.43E-09
CMPK2	2:6996862	-0.0543	0.00598	1.57E-19
CMSS1	3:99906264	0.058	0.00481	4.86E-33
CMTM1	16:66557418	-0.08	0.0107	8.58E-14
CMTM3	16:66629177	-0.0664	0.00838	2.76E-15
CMTM6	3:32559556	0.0261	0.00397	5.61E-11
CMTM7	3:32427326	0.129	0.0136	3.07E-21
CMTM8	3:32286791	-0.132	0.00718	5.99E-73
CNDP2	18:72174023	0.105	0.00446	4.35E-117
CNEP1R1	16:50058869	-0.068	0.00946	7.48E-13
CNGA1	4:47915437	-0.352	0.0115	4.25E-188
CNIH	14:54890924	-0.0421	0.00562	7.37E-14

CNIH4	1:224560371	0.108	0.00805	3.05E-40
CNN2	19:1038871	-0.222	0.00835	1.46E-145
CNN3	1:95389337	0.0704	0.0111	2.46E-10
CNNM3	2:97489870	0.0402	0.00497	6.87E-16
CNOT3	19:54634143	0.0599	0.00782	2.16E-14
CNOT4	7:135198589	-0.0379	0.0046	2.10E-16
CNOT6	5:179996111	-0.108	0.0051	4.07E-95
CNOT6L	4:78738705	-0.0371	0.00602	7.55E-10
CNPPD1	2:220037666	-0.0592	0.0063	7.67E-21
CNPY3	6:42902508	-0.0207	0.00353	4.83E-09
CNPY4	7:99745585	0.0516	0.00793	8.47E-11
CNR2	1:24204008	-0.106	0.0107	7.97E-23
CNRIP1	2:68495002	0.0557	0.0033	3.95E-62
CNTD1	17:40960253	0.0992	0.00829	1.37E-32
CNTLN	9:17325279	-0.0466	0.00738	3.01E-10
CNTNAP2	7:148000098	0.0349	0.00259	1.05E-40
CNTRL	9:123870336	0.17	0.0278	9.57E-10
COA1	7:43668087	-0.102	0.00495	3.73E-91
COCH	14:31328045	-0.0386	0.00407	3.90E-21
COG1	17:71192663	0.0191	0.00314	1.26E-09
COG3	13:46093557	0.0523	0.00278	1.88E-76
COG4	16:70584390	-0.0623	0.00324	7.42E-80
COG5	7:106796537	-0.173	0.00362	<1.0E-314
COG6	13:40215836	-0.0818	0.004	1.53E-89
COG8	16:69365430	-0.031	0.00301	9.22E-25
COL10A1	6:116453231	0.0284	0.00434	6.85E-11
COL18A1	21:46908008	0.0358	0.00173	6.79E-92
COL5A2	2:189913123	-0.0557	0.00858	9.19E-11
COL5A3	19:10118375	-0.0178	0.00209	1.96E-17
COL9A3	20:61464760	0.0918	0.00506	1.54E-71
COLQ	3:15567137	-0.0265	0.0028	4.55E-21
COMMD5	8:146067054	0.0204	0.00234	4.82E-18
COMMD6	13:76089580	0.0946	0.00812	5.33E-31
COMT	22:19951271	-0.0257	0.00339	3.52E-14
COPB1	11:14489182	0.0771	0.00284	7.18E-152
COPB2	3:139060722	-0.0268	0.00379	1.61E-12
COPS3	17:17166056	0.124	0.00468	8.22E-146
COPS6	7:99726446	0.0816	0.00917	7.29E-19
COPS7A	12:6853891	0.0544	0.0056	4.51E-22

COPS8	2:238032491	0.0836	0.00935	5.43E-19
COPZ2	17:46163867	-0.0805	0.00725	2.70E-28
COQ10B	2:198361058	0.0277	0.00464	2.42E-09
COQ2	4:84242078	-0.0863	0.00784	6.73E-28
COQ6	14:74451315	0.0281	0.00409	7.21E-12
COQ7	16:19105138	-0.126	0.0105	2.96E-32
COQ9	16:57445376	-0.0265	0.00378	2.64E-12
CORIN	4:47833295	-0.124	0.00304	<1.0E-314
CORO1C	12:109158025	-0.0806	0.00526	6.18E-52
CORO2A	9:100958924	-0.0902	0.00868	4.43E-25
CORO2B	15:68923210	0.0211	0.00306	5.35E-12
COTL1	16:84599070	-0.0833	0.00531	3.55E-54
COX10	17:13972860	-0.0897	0.013	6.82E-12
COX11	17:53041890	0.0882	0.00626	2.79E-44
COX14	12:50528977	-0.0587	0.00776	4.53E-14
COX15	10:101474499	-0.0453	0.00354	6.87E-37
COX18	4:73922549	-0.035	0.00571	9.27E-10
COX4I1	16:85838916	-0.0445	0.00437	3.79E-24
COX6B1	19:36157167	-0.0535	0.00365	1.03E-47
COX6C	8:100843414	-0.385	0.00754	<1.0E-314
COX7A2	6:75910031	0.451	0.0446	8.22E-24
COX7A2L	2:42591387	0.0441	0.00427	1.11E-24
CPA3	3:148628004	0.174	0.0271	1.50E-10
CPA5	7:130006808	-0.0611	0.00345	2.61E-68
CPAMD8	19:17118433	0.014	0.00239	4.70E-09
CPB1	3:148546321	-0.0473	0.00473	2.89E-23
CPD	17:28667282	0.0816	0.00492	3.39E-60
CPEB2	4:15077693	0.0879	0.00406	1.73E-99
CPEB3	10:93879078	0.0344	0.00353	2.39E-22
CPEB4	5:173305432	-0.0676	0.00431	2.95E-54
CPED1	7:120648485	-0.248	0.00896	1.32E-157
CPM	12:69331714	-0.0425	0.00517	2.27E-16
CPNE1	20:34218673	0.118	0.0037	3.47E-206
CPNE3	8:87573853	-0.0381	0.00571	2.68E-11
CPNE8	12:39145545	-0.203	0.0303	2.29E-11
CPOX	3:98302932	-0.0649	0.00371	8.15E-67
CPPED1	16:12897395	-0.112	0.00636	1.74E-67
CPQ	8:97749564	0.0544	0.00528	1.06E-24
CPSF1	8:145627556	0.0954	0.00738	1.30E-37

CPSF2	14:92587277	-0.0639	0.00323	6.50E-84
CPSF3	2:9654601	-0.0361	0.00611	3.61E-09
CPSF6	12:69655167	-0.0336	0.00319	1.36E-25
CPVL	7:29187946	-0.129	0.00595	4.06E-100
CR1	1:207673631	0.0591	0.00608	3.60E-22
CR2	1:207655815	-0.0471	0.00677	4.04E-12
CRAT	9:131894934	0.0823	0.00329	5.33E-131
CRBN	3:3192524	0.0399	0.00331	4.60E-33
CRCP	7:65570310	-0.091	0.00669	2.04E-41
CREB1	2:208371637	-0.0402	0.00419	1.29E-21
CREB5	7:28724374	0.115	0.00703	1.08E-58
CREG1	1:167516862	-0.119	0.00835	2.65E-45
CRELD2	22:50316797	-0.11	0.00573	1.63E-79
CREM	10:35514002	0.0429	0.0045	2.59E-21
CRIM1	2:36789166	0.0788	0.00299	7.32E-144
CRIP3	6:43260008	0.0298	0.00305	1.99E-22
CRIPAK	4:1341553	-1.22	0.0509	4.41E-120
CRIPT	2:46849495	0.0887	0.00436	1.72E-88
CRISP2	6:49669359	-0.225	0.0195	2.43E-30
CRISP3	6:49674612	0.114	0.0124	6.59E-20
CRISPLD2	16:84845034	0.154	0.00697	1.08E-103
CRK	17:1323238	0.108	0.0143	4.94E-14
CRLF3	17:29078224	0.096	0.00527	5.76E-72
CRNKL1	20:20033755	-0.0363	0.00262	8.66E-43
CROCCP3	1:16786928	-0.0378	0.00606	4.65E-10
CROT	7:86928387	-0.0427	0.00478	5.68E-19
CRTAM	11:122714782	-0.072	0.00631	8.17E-30
CRY1	12:107448333	0.0281	0.00425	4.66E-11
CRY2	11:45846619	-0.0215	0.00269	1.60E-15
CRYM	16:21272591	0.0357	0.00356	1.56E-23
CRYZ	1:75193264	-0.373	0.00771	<1.0E-314
CSAD	12:53547987	0.0247	0.00402	7.89E-10
CSDA	12:10870900	0.127	0.0103	2.95E-34
CSF1R	5:149483427	0.0567	0.00666	2.40E-17
CSF2RB	22:37269541	-0.0613	0.00631	4.28E-22
CSF3R	1:36940615	0.143	0.0191	8.66E-14
CSGALNACT1	8:19564149	0.228	0.00562	<1.0E-314
CSGALNACT2	10:43647868	-0.0513	0.00481	3.19E-26
CSK	15:75057747	0.0369	0.0032	1.94E-30

CSMD1	8:2970477	-0.0154	0.00223	5.98E-12
CSNK1A1L	13:37679729	0.0402	0.00368	1.63E-27
CSNK1G1	15:64650273	-0.0358	0.00448	1.60E-15
CSNK1G3	5:122818269	-0.0353	0.00457	1.32E-14
CSNK2B	6:31637807	-0.084	0.00406	1.69E-91
CSRNP2	12:51503442	0.0278	0.00429	9.18E-11
CSRP2BP	20:18120761	-0.0423	0.00317	5.85E-40
CST7	20:24930085	0.197	0.00908	1.19E-99
CSTA	3:122055495	0.158	0.00817	7.83E-81
CSTB	21:45201832	-0.106	0.00322	5.75E-217
CSTF2T	10:53452245	0.0757	0.00635	2.38E-32
CSTF3	11:33118137	-0.054	0.00394	5.97E-42
CTAGE5	14:39850261	0.0315	0.00359	2.36E-18
CTBP1	4:1198957	-0.0219	0.00237	3.04E-20
CTBP1-AS1	4:1240636	0.109	0.00715	3.41E-51
CTBS	1:85064272	0.076	0.00698	2.65E-27
CTC1	17:8142129	0.0908	0.00346	1.59E-142
CTDP1	18:77492750	-0.0237	0.00254	1.47E-20
CTGF	6:132320848	-0.0194	0.00319	1.20E-09
CTH	1:70876758	0.0456	0.00374	9.61E-34
CTHRC1	8:104431198	-0.0509	0.00501	5.65E-24
CTNNA1	5:138085286	0.136	0.00397	2.44E-231
CTNNA3	10:69264209	-0.0664	0.00897	1.58E-13
CTNNAL1	9:111680359	-0.504	0.0336	1.09E-49
CTNNB1	3:41260402	0.0572	0.00275	1.12E-92
CTNND1	11:57537787	0.0268	0.00396	1.58E-11
CTNS	17:3564836	0.0439	0.00509	8.39E-18
CTPS1	1:41446231	0.0447	0.00412	3.87E-27
CTSA	20:44501934	-0.032	0.00423	5.18E-14
CTSB	8:11720502	0.227	0.026	3.28E-18
CTSC	11:88071498	0.149	0.00518	4.63E-170
CTSD	11:1769843	0.105	0.00859	4.94E-34
CTSF	11:66335832	-0.0181	0.00261	4.44E-12
CTSG	14:25045589	-0.201	0.016	1.05E-35
CTSH	15:79233792	0.322	0.014	1.10E-111
CTSK	1:150772613	-0.116	0.00749	2.25E-53
CTSL1	9:90342675	0.195	0.00788	2.27E-127
CTSS	1:150737565	0.0294	0.00415	1.73E-12
CTSW	11:65654665	0.285	0.00848	2.72E-224

CTSZ	20:57574841	-0.0383	0.00472	5.70E-16
CUBN	10:17175484	-0.168	0.00397	<1.0E-314
CUL2	10:35355402	0.0426	0.0032	6.39E-40
CUL7	6:43008876	0.0566	0.00461	3.14E-34
CUZD1	10:124638827	0.032	0.00493	9.27E-11
CWC25	17:36991976	-0.0355	0.00358	5.99E-23
CWF19L1	10:101953705	-0.0888	0.00336	1.88E-144
CWF19L2	11:107273938	0.0809	0.00559	1.64E-46
CXCL1	4:74735244	0.0501	0.00744	1.85E-11
CXCL16	17:4640869	0.115	0.00544	6.03E-96
CXCL5	4:74857970	-0.185	0.00702	9.88E-144
CXCR2P1	2:218970736	-0.097	0.00609	9.82E-56
CXCR7	2:237445326	0.0656	0.0108	1.13E-09
CYB5A	18:71948257	-0.0649	0.0101	1.54E-10
CYB5B	16:69497203	0.039	0.00574	1.28E-11
CYB5D2	17:4038014	-0.0268	0.00445	1.71E-09
CYB5R1	1:202967764	-0.0371	0.00583	2.20E-10
CYB5R2	11:7692051	-0.0591	0.00464	1.09E-36
CYB5R3	22:43022832	0.0617	0.00556	2.84E-28
CYBA	16:88710882	-0.089	0.00353	7.20E-133
CYBRD1	2:172369069	0.243	0.0065	9.05E-272
CYCS	7:25163744	-0.138	0.0109	2.24E-36
CYFIP2	5:156769649	-0.0369	0.00588	3.66E-10
CYLD	16:50727075	0.0383	0.00319	9.46E-33
CYP1B1	2:38351969	-0.135	0.0102	3.72E-39
CYP1B1-AS1	2:38416531	-0.0303	0.00434	3.32E-12
CYP26B1	2:72403408	-0.0851	0.00419	1.92E-88
CYP27A1	2:219597185	-0.294	0.00538	<1.0E-314
CYP2B7P1	19:41415087	-0.0872	0.00762	6.17E-30
CYP2R1	11:14861517	0.0521	0.00516	1.09E-23
CYP2U1	4:108877783	0.0487	0.0045	5.00E-27
CYP4F12	19:15776028	-0.0896	0.00595	3.40E-50
CYP4F22	19:15632989	-0.176	0.027	6.97E-11
CYP4F3	19:15749169	-0.109	0.00847	3.62E-37
CYP4V2	4:187099609	-0.141	0.00531	1.15E-146
CYP51A1	7:91749798	0.258	0.00771	1.72E-222
CYSTM1	5:139665761	0.0573	0.00788	3.92E-13
CYTH2	19:48986144	0.0941	0.0118	2.16E-15
CYTH4	22:37678378	-0.0419	0.00375	9.31E-29

CYYR1	21:27973726	-0.0338	0.00402	4.92E-17
DAAM2	6:39712340	-0.0451	0.00731	7.05E-10
DAD1	14:23058084	-0.231	0.00483	<1.0E-314
DAGLB	7:6456091	-0.0715	0.00396	6.93E-71
DALRD3	3:49030828	-0.0326	0.003	2.77E-27
DAP	5:10676209	0.0555	0.00413	1.22E-40
DAP3	1:155614815	0.0357	0.00455	5.14E-15
DAPK1	9:90167240	-0.218	0.00538	<1.0E-314
DAPK2	15:64279053	-0.0607	0.00459	2.60E-39
DAPP1	4:100760165	0.0668	0.00504	1.55E-39
DARC	1:159174920	-0.0543	0.0044	1.44E-34
DARS	2:136773803	-0.278	0.0251	3.23E-28
DAXX	6:33290402	0.0232	0.00358	1.02E-10
DAZAP2	12:51641035	-0.0336	0.00386	4.32E-18
DBF4B	17:42773081	0.0561	0.00298	1.11E-76
DBI	2:120120771	0.0479	0.00488	1.43E-22
DBIL5P2	2:63328774	-0.141	0.00922	1.69E-51
DBN1	5:176875956	0.0201	0.0025	1.25E-15
DBNL	7:44082764	0.0278	0.00449	6.97E-10
DCAF10	9:37854274	0.275	0.00749	8.89E-263
DCAF13	8:104410420	0.028	0.00361	1.17E-14
DCAF4	14:73393391	-0.0985	0.00497	2.61E-84
DCAKD	17:43093021	0.0506	0.00383	3.45E-39
DCBLD1	6:117791163	0.209	0.00531	5.95E-298
DCBLD2	3:98661631	-0.102	0.00622	2.17E-59
DCHS1	11:6679132	-0.0228	0.00377	1.53E-09
DCK	4:71860311	-0.213	0.0297	7.69E-13
DCLK2	4:151049132	-0.0544	0.004	2.29E-41
DCP1A	3:53271193	-0.203	0.0196	6.09E-25
DCP1B	12:2064575	0.0895	0.00806	2.30E-28
DCP2	5:112326410	0.0768	0.00322	7.81E-120
DCPS	11:126156979	0.0457	0.00394	1.10E-30
DCTD	4:183820557	-0.0536	0.00475	3.21E-29
DCTN5	16:23653343	-0.223	0.00484	<1.0E-314
DCTN6	8:30049102	-0.081	0.0079	1.98E-24
DCUN1D2	13:114171794	-0.0587	0.00568	7.32E-25
DCUN1D4	4:52788822	0.0434	0.00457	3.44E-21
DCXR	17:79993003	-0.138	0.00497	2.14E-157
DDA1	19:17426171	-0.054	0.00654	1.91E-16

DDAH1	1:85749715	-0.0369	0.00435	2.99E-17
DDAH2	6:31697957	-0.0329	0.00458	7.57E-13
DDB2	11:47263060	0.06	0.00483	6.56E-35
DDHD2	8:38107873	-0.0646	0.00517	2.43E-35
DDI2	1:15921899	0.0848	0.00667	1.72E-36
DDO	6:110738291	0.0307	0.00346	9.26E-19
DDOST	1:20983863	0.027	0.00356	4.01E-14
DDR1	6:30892999	-0.0208	0.00221	7.32E-21
DDRGK1	20:3155375	0.0454	0.00346	1.01E-38
DDT	22:24335228	0.306	0.0162	3.11E-77
DDX1	2:15729583	-0.0239	0.003	1.95E-15
DDX10	11:108591328	-0.0544	0.00664	3.09E-16
DDX17	22:38879688	-0.0261	0.00224	6.73E-31
DDX18	2:118596654	-0.0544	0.0043	3.06E-36
DDX20	1:112279555	0.0569	0.00706	9.30E-16
DDX23	12:49236156	0.0314	0.00391	1.04E-15
DDX27	20:47908060	-0.0237	0.00373	2.23E-10
DDX31	9:135488542	-0.0206	0.00265	8.88E-15
DDX39B	6:31509779	0.0338	0.00284	2.87E-32
DDX43	6:74127543	-0.146	0.0214	8.77E-12
DDX5	17:62502435	0.0981	0.00674	3.73E-47
DDX50	10:70670776	0.0406	0.00622	7.13E-11
DDX52	17:36002599	0.0438	0.00522	5.92E-17
DDX55	12:124069614	0.0857	0.00491	2.18E-66
DDX56	7:44612753	-0.0436	0.00322	5.56E-41
DDX59	1:200645101	0.091	0.0036	7.13E-133
DECR1	8:91018214	0.213	0.0142	1.10E-49
DEF6	6:35236915	0.0624	0.003	1.23E-92
DEFA4	8:6789291	-0.199	0.0322	7.15E-10
DEGS1	1:224424442	-0.136	0.00942	2.33E-46
DEK	6:18262906	-0.0412	0.0036	6.13E-30
DENND1A	9:126641091	0.0886	0.00512	2.98E-65
DENND2C	1:115212525	-0.053	0.00715	1.41E-13
DENND2D	1:111729325	0.043	0.00456	6.08E-21
DENND4A	15:65998702	0.0796	0.00575	9.08E-43
DENND4C	9:19227803	0.0347	0.0036	9.27E-22
DENND5A	11:9270700	-0.0357	0.00429	1.01E-16
DENND5B	12:31647153	0.0421	0.00446	5.41E-21
DENND6A	3:57692917	-0.0841	0.00405	3.71E-92

DEPDC5	22:32182708	-0.0289	0.00322	3.97E-19
DEPDC7	11:33057988	-0.0369	0.00315	3.19E-31
DEPTOR	8:120901797	0.0424	0.00397	2.51E-26
DERL1	8:124043301	0.0243	0.00403	1.79E-09
DFNB31	9:117261987	-0.0162	0.00195	9.08E-17
DGAT2	11:75561128	0.0513	0.00602	2.21E-17
DGCR2	22:19099813	0.0311	0.00333	1.75E-20
DGCR8	22:20090192	-0.042	0.00371	2.40E-29
DGKA	12:56313676	-0.0515	0.0077	2.53E-11
DGKE	17:54883437	-0.0518	0.00828	4.24E-10
DHCR24	1:55373374	-0.0364	0.00523	4.20E-12
DHDDS	1:26805002	0.0284	0.00451	3.10E-10
DHRS1	14:24751184	-0.0895	0.00956	1.12E-20
DHRS12	13:52423280	0.049	0.005	1.55E-22
DHRS3	1:12649332	0.046	0.00612	6.76E-14
DHRS4	14:24439932	0.0956	0.0139	6.14E-12
DHRS7	14:60604297	0.0567	0.00878	1.15E-10
DHRS9	2:169961897	0.194	0.00635	5.44E-188
DHTKD1	10:12111784	-0.0282	0.00393	8.26E-13
DHX32	10:127596596	0.13	0.00431	3.04E-183
DHX34	19:47899522	-0.0427	0.00626	1.04E-11
DHX35	20:37593743	0.063	0.00393	2.31E-56
DHX36	3:154021063	-0.0307	0.00377	4.07E-16
DHX57	2:39102671	0.0651	0.00372	8.90E-67
DHX58	17:40264661	0.0394	0.00386	3.18E-24
DICER1	14:95594687	0.0401	0.00361	2.58E-28
DICER1-AS1	14:95648424	0.0578	0.00548	9.42E-26
DIDO1	20:61533039	0.0801	0.00361	2.60E-104
DIEXF	1:210001586	0.105	0.00335	1.21E-196
DIMT1	5:61719506	-0.0396	0.00333	3.41E-32
DIP2A	21:47833789	-0.138	0.00341	<1.0E-314
DIP2B	12:51130207	0.0423	0.00365	9.73E-31
DIP2C	10:529144	-0.042	0.00373	3.94E-29
DIRC2	3:122480238	0.243	0.00853	8.90E-166
DIS3	13:73341704	0.0222	0.0034	6.28E-11
DIS3L	15:66572692	-0.145	0.00896	9.83E-58
DIS3L2	2:232827254	-0.0306	0.00489	4.56E-10
DISC1	1:231773397	-0.108	0.00332	3.13E-211
DIXDC1	11:111897050	-0.0354	0.00353	1.94E-23

DKFZP686I1521	6:2940666	0.025	0.00366	8.76E-12
DKK3	11:12017400	0.113	0.0102	6.52E-28
DLAT	11:111933088	0.0225	0.00335	1.99E-11
DLEU2	13:50610989	-0.135	0.00873	1.12E-52
DLEU7	13:51293037	0.0343	0.00434	3.53E-15
DLG5	10:79617980	0.0675	0.00254	3.37E-146
DLGAP5	14:55653306	0.0965	0.0104	3.13E-20
DLST	14:75317699	0.0361	0.00536	1.86E-11
DMXL2	15:51914033	-0.0892	0.00537	2.30E-60
DNAAF2	14:50112712	-0.117	0.00567	2.35E-91
DNAH6	2:84709215	0.114	0.00371	7.98E-192
DNAH7	2:196887188	-0.0365	0.00186	3.21E-83
DNAJA3	16:4479734	-0.0433	0.00299	1.12E-46
DNAJA4	15:78569930	0.11	0.00851	2.03E-37
DNAJB1	19:14618185	-0.0945	0.00748	3.93E-36
DNAJB13	11:73682867	-0.0426	0.00427	2.74E-23
DNAJB2	2:220140796	-0.0384	0.00557	5.70E-12
DNAJB5	9:35013174	-0.189	0.0112	3.37E-62
DNAJB6	7:157145474	-0.0887	0.00528	9.00E-62
DNAJB7	22:41256802	0.0381	0.00604	3.08E-10
DNAJB9	7:108211586	0.0343	0.00412	1.08E-16
DNAJC11	1:6771569	0.0273	0.00418	6.72E-11
DNAJC13	3:132183991	-0.094	0.0125	7.40E-14
DNAJC15	13:43608754	-0.242	0.00544	<1.0E-314
DNAJC18	5:138773874	0.0553	0.00512	5.99E-27
DNAJC19	3:180702240	0.0431	0.00679	2.41E-10
DNAJC21	5:34948532	0.0342	0.00462	1.48E-13
DNAJC8	1:28518264	0.0518	0.00863	2.05E-09
DNAJC9	10:75008561	0.258	0.023	1.10E-28
DNASE1L3	3:58199534	0.0747	0.00538	5.25E-43
DNASE2	19:12998205	0.0863	0.00481	7.65E-70
DNHD1	11:6530278	-0.046	0.00347	1.68E-39
DNM2	19:10939792	0.0222	0.00285	8.12E-15
DNMBP	10:101697047	0.135	0.0162	1.06E-16
DNPEP	2:220263811	-0.167	0.0163	2.01E-24
DNTTIP1	20:44404647	0.0363	0.00297	8.49E-34
DNTTIP2	1:94339851	-0.222	0.0346	1.50E-10
DOCK10	2:225755912	-0.0721	0.00626	2.30E-30
DOCK7	1:62993403	0.102	0.00365	1.07E-161

DOCK8	9:370199	-0.0831	0.0048	2.84E-65
DOCK9	13:99550351	-0.0737	0.00597	1.65E-34
DOK2	8:21762915	0.0366	0.00395	2.59E-20
DOK4	16:57511407	-0.0553	0.00751	2.05E-13
DOK6	18:67531642	0.0757	0.00315	5.57E-121
DOM3Z	6:31938412	0.102	0.00589	5.57E-66
DOPEY1	6:83843781	0.154	0.00441	1.13E-240
DOPEY2	21:37620240	-0.0497	0.0035	5.26E-45
DPAGT1	11:118973133	0.0299	0.00376	2.27E-15
DPEP2	16:68039309	-0.0502	0.00606	1.46E-16
DPEP3	16:68036939	0.0451	0.00635	1.47E-12
DPH1	17:1942861	0.0231	0.00276	7.12E-17
DPH3	3:16293425	0.0472	0.00662	1.13E-12
DPM2	9:130722212	-0.115	0.0131	1.45E-18
DPP4	2:162930725	-0.0564	0.00696	6.98E-16
DPP7	9:140024587	0.0735	0.00929	3.05E-15
DPPA4	3:109061221	-0.0545	0.00652	8.78E-17
DPY19L2P2	7:102787381	-0.389	0.0194	1.65E-86
DPY19L3	19:32925370	-0.11	0.00637	2.76E-65
DPY19L4	8:95763759	0.0756	0.0111	1.01E-11
DPYSL4	10:133969872	-0.0286	0.00266	1.13E-26
DR1	1:93816400	-0.122	0.00337	6.00E-255
DRAM2	1:111728610	-0.116	0.00474	1.03E-125
DSC1	18:28737770	0.102	0.00578	2.35E-67
DSC2	18:28724903	0.0788	0.0106	9.67E-14
DSCC1	8:120868138	-0.0874	0.00509	3.29E-64
DSE	6:116795040	0.128	0.0046	1.10E-159
DSN1	20:35404978	0.126	0.0202	4.90E-10
DSP	6:7495948	-0.139	0.00357	9.88E-292
DST	6:56290054	-0.0243	0.00375	9.28E-11
DSTN	20:17627727	-0.0501	0.00524	1.63E-21
DSTYK	1:205138321	-0.101	0.0108	1.16E-20
DTD1	20:18552359	0.0622	0.00469	1.86E-39
DTNB	2:25860320	0.0236	0.00329	9.14E-13
DTNBP1	6:15662891	-0.0837	0.0044	3.94E-78
DTWD1	15:49968752	-0.0613	0.00508	4.70E-33
DTWD2	5:118315744	0.0454	0.00523	5.04E-18
DTX2	7:76135340	0.107	0.0169	3.32E-10
DUOX1	15:45459564	0.0218	0.00268	4.54E-16

DUS2L	16:68127846	-0.158	0.00844	2.66E-75
DUS3L	19:5799806	0.0313	0.00314	2.92E-23
DUSP10	1:221917338	0.0411	0.00478	1.03E-17
DUSP12	1:161721707	0.0305	0.00406	6.56E-14
DUSP14	17:35817105	-0.0277	0.00394	2.14E-12
DUSP16	12:12617006	0.0831	0.00683	1.45E-33
DUSP18	22:31063804	0.176	0.00544	1.44E-209
DUSP3	17:41858810	-0.106	0.00709	7.67E-50
DUSP6	12:89747875	0.0646	0.00795	5.23E-16
DUT	15:48605208	-0.0942	0.0056	5.03E-62
DYM	18:46979529	0.0435	0.00552	4.30E-15
DYNC1LI1	3:32621214	-0.157	0.00486	4.14E-209
DYNC2LI1	2:44001185	0.134	0.0112	1.37E-32
DYNLL1	12:120950249	0.0896	0.0138	8.71E-11
DYNLL2	17:56165525	0.0258	0.0039	4.40E-11
DYNLT1	6:159047087	0.0473	0.00472	1.69E-23
DYRK1A	21:38892198	0.0263	0.00293	4.03E-19
DYX1C1	15:55772799	-0.0889	0.0069	2.11E-37
DZANK1	20:18434153	-0.0151	0.00237	1.88E-10
DZIP3	3:108264948	0.193	0.00465	<1.0E-314
E2F4	16:67178973	-0.0384	0.00635	1.58E-09
EAF1	3:15476011	0.03	0.00368	4.20E-16
EAF2	3:121558894	-0.109	0.00669	8.52E-58
EBLN2	3:73111973	0.0965	0.0043	1.10E-106
EBPL	13:50266544	0.0984	0.0049	2.21E-86
ECD	10:74910452	0.144	0.0072	1.49E-85
ECE1	1:21663030	-0.0957	0.00553	2.38E-65
ECH1	19:39324154	-0.039	0.00314	5.92E-35
ECHDC1	6:127696869	-0.105	0.00493	3.38E-97
ECHDC2	1:53340320	0.0429	0.00373	2.76E-30
ECHDC3	10:11780324	0.0406	0.00451	2.91E-19
ECI2	6:4126097	-0.0249	0.00391	2.16E-10
ECM2	9:95207806	-0.0861	0.00307	2.01E-161
ECSIT	19:11612283	0.0489	0.007	3.22E-12
EDAR	2:109514464	-0.029	0.00387	8.70E-14
EDEM2	20:33730387	0.0364	0.00364	2.85E-23
EDEM3	1:184710178	0.0637	0.00319	2.47E-85
EDIL3	5:83408439	-0.069	0.00366	1.09E-76
EEA1	12:93280699	-0.0471	0.00484	3.33E-22

EED	11:85964887	-0.0596	0.00289	4.11E-91
EEF1DP3	13:32444439	-0.0673	0.0041	5.99E-59
EEF2	19:3990291	-0.0292	0.00486	2.09E-09
EEPD1	7:36192787	0.0326	0.00376	5.86E-18
EFCAB13	17:45401335	0.431	0.0115	2.48E-272
EFCAB2	1:245199100	0.351	0.00932	1.67E-275
EFCAB5	17:28223982	0.0345	0.00317	2.60E-27
EFCAB6	22:44212838	0.0171	0.00244	3.03E-12
EFCAB7	1:64065817	-0.0398	0.00387	1.31E-24
EFEMP2	11:65595803	-0.0243	0.00385	2.95E-10
EFHA1	13:22155152	-0.0564	0.00548	1.33E-24
EFHA2	8:16924115	-0.0692	0.00548	4.65E-36
EFHB	3:19987539	-0.0884	0.00338	1.11E-141
EFHC1	6:52268008	0.0432	0.00516	7.18E-17
EFHD2	1:15738365	0.164	0.0172	2.56E-21
EFTUD1	15:82427605	-0.0352	0.0046	2.30E-14
EFTUD2	17:42986982	0.0297	0.00377	4.21E-15
EGF	4:110891673	0.0335	0.0054	5.59E-10
EGLN2	19:41305793	-0.0518	0.00354	1.57E-47
EHD3	2:31480967	-0.041	0.00532	1.50E-14
EHHADH	3:184886137	0.0429	0.00518	1.54E-16
EHMT1	9:140604118	-0.0366	0.00253	1.47E-46
EI24	11:125438802	-0.0383	0.00628	1.19E-09
EIF2A	3:150259942	0.0515	0.0046	8.56E-29
EIF2AK1	7:6060908	-0.0864	0.013	3.13E-11
EIF2AK2	2:37363968	0.141	0.0218	8.64E-11
EIF2AK4	15:40283250	0.0405	0.00481	4.61E-17
EIF2B2	14:75446778	0.056	0.00402	2.84E-43
EIF2C2	8:141604684	-0.0577	0.00424	2.04E-41
EIF3G	19:10227010	0.0642	0.00749	1.40E-17
EIF3K	19:39106022	-0.055	0.00928	3.36E-09
EIF4A3	17:78112333	-0.0346	0.00382	1.89E-19
EIF4B	12:53401481	0.0628	0.00861	3.29E-13
EIF4E2	2:233440924	-0.0309	0.00482	1.58E-10
EIF4E3	3:71769437	0.0993	0.0045	1.57E-103
EIF4EBP2	10:72202312	-0.0302	0.00499	1.58E-09
EIF4ENIF1	22:31861950	-0.0155	0.00253	9.69E-10
EIF4G3	1:21351885	0.0609	0.00672	1.80E-19
EIF5	14:103753469	0.0815	0.0118	5.46E-12

EIF6	20:33865852	-0.0768	0.00562	6.79E-42
ELAC2	17:12914024	0.0256	0.00353	4.68E-13
ELAVL1	19:8028544	0.0378	0.00344	8.93E-28
ELF2	4:140021554	0.0441	0.00477	3.43E-20
ELK3	12:96599780	-0.0243	0.00401	1.51E-09
ELL	19:18567039	0.0255	0.00327	6.89E-15
ELL2	5:95262044	0.0661	0.00538	2.89E-34
ELMO1	7:37455281	-0.108	0.00427	1.26E-133
ELMOD2	4:141430495	0.0375	0.00608	7.14E-10
ELOF1	19:11659930	-0.0898	0.0143	3.63E-10
ELOVL2	6:10945334	-0.0319	0.00328	3.93E-22
ELOVL4	6:80704185	-0.0494	0.00593	1.06E-16
ELOVL5	6:53153954	0.0352	0.00345	3.54E-24
ELP2	18:33759196	-0.072	0.00365	1.02E-83
ELP3	8:27950008	-0.083	0.00363	1.29E-110
ELP6	3:47531935	0.0227	0.0034	2.83E-11
EMB	5:49734505	-0.0848	0.00871	3.37E-22
EMC1	1:19558584	-0.0236	0.00335	1.95E-12
EMC2	8:109536300	0.0713	0.0055	5.94E-38
EML3	11:62378221	-0.0482	0.00401	7.52E-33
EML4	2:42526974	0.0342	0.00439	7.68E-15
EMR1	19:6900955	-0.134	0.00958	6.00E-44
EMR2	19:14887259	-0.266	0.00867	4.20E-190
EMR3	19:14776900	-0.0773	0.0066	2.90E-31
ENC1	5:73884146	-0.473	0.00811	<1.0E-314
ENDOD1	11:94863186	-0.0768	0.00457	8.22E-62
ENDOG	9:131614082	0.0632	0.00494	6.25E-37
ENDOU	12:48053875	-0.0899	0.00618	4.71E-47
ENG	9:130628665	0.0495	0.00502	1.06E-22
ENGASE	17:77035607	-0.0315	0.00296	3.47E-26
ENKUR	10:25317626	-0.069	0.0104	3.62E-11
ENO1	1:8913653	0.0384	0.0049	4.87E-15
ENO3	17:4856580	-0.0403	0.00417	7.19E-22
ENOSF1	18:712319	-0.139	0.00464	1.07E-182
ENPP1	6:132223944	-0.0277	0.00452	9.84E-10
ENPP2	8:120648375	-0.0732	0.00568	1.87E-37
ENPP3	6:132018000	-0.0629	0.00807	7.49E-15
ENPP4	6:46116102	0.0866	0.00674	2.98E-37
ENTPD1	10:97559214	-0.294	0.0102	1.54E-169

ENTPD3	3:40483027	-0.0456	0.00307	9.22E-49
ENTPD4	8:23273758	-0.0404	0.00298	2.82E-41
ENTPD5	14:74480136	0.0606	0.0072	5.45E-17
ENTPD6	20:25186137	0.0242	0.00248	2.18E-22
EOGT	3:69055411	-0.0283	0.00344	2.28E-16
EOMES	3:27759338	0.0433	0.00618	2.80E-12
EP400NL	12:132568660	0.127	0.00552	2.27E-111
EPAS1	2:46623611	-0.0186	0.00257	6.09E-13
EPB41L2	6:131383686	-0.0912	0.00556	5.04E-59
EPB41L3	18:5452310	0.0697	0.00619	4.66E-29
EPB41L4A	5:111669756	-0.101	0.00983	1.43E-24
EPB41L5	2:120903167	0.0343	0.00484	1.59E-12
EPC1	10:32650071	-0.0223	0.00332	1.94E-11
EPDR1	7:37958467	0.0267	0.00378	1.90E-12
EPG5	18:43563022	-0.0734	0.00511	5.93E-46
EPHA1	7:143104331	0.0389	0.00318	5.29E-34
EPHA4	2:222393635	0.108	0.0045	1.55E-120
EPHB2	1:23054719	0.0318	0.00463	7.32E-12
EPHB4	7:100378274	-0.0533	0.00232	1.15E-111
EPHX2	8:27389702	-0.0512	0.00795	1.26E-10
EPHX4	1:92496655	-0.0676	0.0085	2.26E-15
EPM2A	6:146003401	-0.076	0.00373	8.84E-89
EPS15	1:51811930	-0.143	0.0124	2.74E-30
EPS8	12:15843771	-0.0254	0.00429	3.33E-09
EPSTI1	13:43566373	-0.26	0.014	1.43E-74
EPT1	2:26583506	0.0391	0.00401	3.24E-22
ERAP1	5:96121715	-0.111	0.00327	3.87E-228
ERAP2	5:96200770	-0.464	0.00661	<1.0E-314
ERBB2IP	5:65314414	0.0286	0.00464	7.58E-10
ERC1	12:1160615	0.0417	0.00508	2.67E-16
ERCC3	2:127992907	-0.0532	0.00447	2.78E-32
ERCC5	13:103501256	-0.0266	0.00369	7.22E-13
ERCC6	10:50772362	-0.0314	0.00298	9.81E-26
ERCC6L2	9:98650046	0.0563	0.0044	6.18E-37
EREG	4:75190202	0.0677	0.00661	2.26E-24
ERGIC1	5:172365059	0.0303	0.00399	3.68E-14
ERGIC2	12:29560218	-0.0869	0.0097	4.45E-19
ERI1	8:8882571	0.0655	0.00428	1.12E-51
ERICH1	8:638867	0.177	0.00459	1.79E-286

ERLEC1	2:54002064	-0.0261	0.00432	1.60E-09
ERLIN1	10:101903906	0.0864	0.00628	2.44E-42
ERMAP	1:43311563	-0.0651	0.00456	2.52E-45
ERN1	17:62145672	0.0866	0.00403	3.30E-98
ERO1L	14:53160317	0.343	0.00762	<1.0E-314
ERO1LB	1:236394926	0.0911	0.0039	5.70E-115
ERP27	12:15085950	0.0353	0.00591	2.62E-09
ERV3-1	7:64446245	0.321	0.0226	6.93E-45
ERVFRD-1	6:11095317	0.033	0.00458	6.74E-13
ESD	13:47371659	0.0367	0.0043	1.86E-17
ESF1	20:13704953	0.0371	0.00599	6.79E-10
ESPN	1:6487031	-0.138	0.00816	3.06E-62
ESYT2	7:158521732	-0.0853	0.00431	4.11E-84
ETAA1	2:67587192	0.065	0.00684	2.96E-21
ETFA	15:76603679	-0.135	0.00691	1.43E-82
ETFB	19:51850290	-0.0381	0.00319	1.66E-32
ETFDH	4:159601676	0.0732	0.0054	3.86E-41
ETS1	11:128330520	0.0317	0.0044	6.57E-13
ETS2	21:40206859	0.034	0.00386	1.73E-18
ETV5	3:185822353	-0.045	0.00551	4.33E-16
ETV7	6:36385935	-0.0674	0.00401	8.66E-62
EVA1C	21:33787602	-0.0362	0.00517	2.97E-12
EVI2A	17:29671327	-0.0923	0.00598	9.92E-53
EVI2B	17:29590121	-0.0913	0.0135	1.37E-11
EVI5	1:93266838	-0.0904	0.00713	2.26E-36
EXO5	1:41005412	-0.15	0.0105	8.67E-45
EXOC1	4:56770708	-0.0431	0.00526	2.89E-16
EXOC2	6:671354	-0.063	0.00335	1.77E-76
EXOC3	5:457261	0.117	0.00382	2.02E-191
EXOC4	7:132980371	0.0704	0.00695	7.02E-24
EXOC7	17:74083905	-0.0163	0.00271	1.69E-09
EXOC8	1:231461261	-0.0328	0.00382	1.17E-17
EXOSC1	10:99205731	0.104	0.00685	1.15E-50
EXOSC10	1:11128654	-0.0337	0.00344	1.51E-22
EXOSC3	9:37781891	0.122	0.00712	4.07E-64
EXOSC6	16:70315911	-0.0327	0.00307	2.90E-26
EXOSC7	3:45051827	-0.0626	0.00449	2.38E-43
EXOSC8	13:37583831	0.134	0.00495	1.55E-150
EXOSC9	4:122729251	-0.0505	0.00423	1.94E-32

EXPH5	11:108464209	0.0519	0.00314	5.94E-60
EXT1	8:118870151	-0.0581	0.00513	2.01E-29
EXT2	11:44087989	-0.104	0.00439	2.85E-118
EXTL2	1:101363666	0.0792	0.0116	1.12E-11
EYA3	1:28416157	-0.364	0.00949	5.95E-284
EZH2	7:148525904	0.0536	0.00695	1.43E-14
F11R	1:161000113	-0.0407	0.00465	2.74E-18
F13A1	6:6333084	-0.0998	0.011	1.81E-19
F2R	5:76037551	0.0744	0.00703	7.31E-26
F2RL1	5:76114963	-0.174	0.00522	1.46E-221
F5	1:169540080	0.154	0.00688	1.46E-106
FAAH	1:46898063	-0.016	0.0027	3.68E-09
FADD	11:70049523	0.0406	0.00313	5.15E-38
FADS1	11:61556039	-0.115	0.00538	2.65E-97
FADS2	11:61546592	-0.273	0.00604	<1.0E-314
FAH	15:80473662	0.0811	0.00641	3.15E-36
FAHD1	16:1886022	0.236	0.0119	1.43E-84
FAIM3	1:207080565	-0.0685	0.00457	1.17E-49
FAM102A	9:130699319	-0.0743	0.00931	1.77E-15
FAM104A	17:71188992	0.0543	0.00715	3.79E-14
FAM105A	5:14572712	-0.103	0.0163	3.20E-10
FAM105B	5:14654464	-0.0501	0.00565	1.04E-18
FAM107B	10:14562793	0.0563	0.00366	2.54E-52
FAM110A	20:826492	0.0453	0.00331	6.84E-42
FAM114A1	4:38878081	-0.045	0.00659	9.60E-12
FAM114A2	5:153390416	0.052	0.00313	1.47E-60
FAM117B	2:203491699	0.193	0.00487	2.29E-300
FAM118A	22:45690275	-2.14	0.0317	<1.0E-314
FAM118B	11:126060666	-0.0894	0.00502	7.16E-69
FAM120B	6:170635900	0.053	0.00371	1.58E-45
FAM126A	7:23036691	-0.167	0.0136	3.53E-34
FAM126B	2:201858087	-0.127	0.0203	4.61E-10
FAM134A	2:220041928	0.0255	0.00426	2.38E-09
FAM135A	6:71192813	0.0993	0.0058	6.84E-64
FAM136A	2:70506809	0.0467	0.00728	1.51E-10
FAM13A	4:89848583	-0.105	0.00609	2.58E-64
FAM151B	5:79782137	0.0903	0.00657	3.35E-42
FAM153B	5:175556054	-0.265	0.0438	1.60E-09
FAM159A	1:53100880	-0.0335	0.00506	3.59E-11

FAM160A2	11:6228594	-0.0492	0.00306	9.93E-57
FAM160B1	10:116604344	0.0423	0.00342	8.65E-35
FAM168B	2:131810267	-0.0322	0.00462	3.34E-12
FAM173B	5:10270030	-0.119	0.0153	1.25E-14
FAM174A	5:99970339	0.243	0.013	8.21E-76
FAM175A	4:84397133	-0.0644	0.00381	2.22E-62
FAM177A1	14:35499482	0.0721	0.00452	6.37E-56
FAM177B	1:222940752	0.117	0.00764	4.24E-52
FAM178A	10:102674531	-0.0323	0.00411	4.82E-15
FAM184A	6:119247734	-0.0288	0.00302	1.96E-21
FAM188A	10:15843462	0.0425	0.00606	2.38E-12
FAM194A	3:150419164	-0.0246	0.00339	5.04E-13
FAM198B	4:159018833	-0.34	0.0123	3.87E-156
FAM19A1	3:68169693	0.192	0.00761	2.53E-133
FAM19A2	12:62631026	-0.14	0.00719	3.17E-81
FAM208B	10:5850664	0.0366	0.00598	9.37E-10
FAM210B	20:54935242	-0.0723	0.00724	2.80E-23
FAM212B	1:112238216	-0.0917	0.00955	1.10E-21
FAM213A	10:82203293	0.0921	0.00593	3.06E-53
FAM216A	12:110897202	0.0749	0.00561	5.60E-40
FAM220A	7:6371100	-0.0472	0.00334	1.61E-44
FAM227B	15:49592511	-0.0287	0.00354	5.94E-16
FAM228A	2:24371691	-0.0242	0.0028	8.38E-18
FAM3B	21:42659676	-0.663	0.0141	<1.0E-314
FAM43A	3:194403491	-0.0575	0.0036	3.81E-56
FAM46C	1:118154575	0.0937	0.00866	5.25E-27
FAM47E	4:77227485	0.0896	0.00692	8.07E-38
FAM53A	4:1650891	0.0346	0.00584	3.18E-09
FAM53C	5:137673167	-0.0197	0.00319	6.82E-10
FAM63B	15:59063196	0.104	0.00564	8.78E-74
FAM65A	16:67607848	0.0394	0.00535	2.03E-13
FAM65B	6:24819447	0.02	0.00326	9.42E-10
FAM76B	11:95533557	0.081	0.00336	4.73E-122
FAM81B	5:94810950	-0.0408	0.00503	6.08E-16
FAM82A1	2:38153486	-0.0984	0.00487	1.17E-87
FAM82B	8:87491951	0.0431	0.00435	7.23E-23
FAM83A	8:124187620	-0.0339	0.00533	2.28E-10
FAM84B	8:127516678	0.0888	0.00649	7.46E-42
FAM91A1	8:124785708	0.115	0.0145	2.44E-15

FAM92A1	8:94771530	-0.0346	0.00527	5.47E-11
FAM96A	15:64416621	0.0366	0.00563	8.82E-11
FAM98C	19:38899548	-0.0469	0.00574	4.08E-16
FAN1	15:31157666	-0.141	0.00373	6.27E-278
FANCA	16:89825464	0.058	0.00238	1.25E-124
FANCD2	3:10067651	0.0651	0.00424	3.92E-52
FANCF	11:22665932	-0.0427	0.00566	5.57E-14
FANCI	15:89869329	0.0523	0.0065	1.05E-15
FANCL	2:58468569	-0.462	0.0228	7.91E-88
FANK1	10:127680550	-0.0718	0.00543	2.49E-39
FAR2	12:29491528	-0.06	0.00499	6.52E-33
FARP1	13:99102279	-0.0261	0.00208	1.60E-35
FARP2	2:242395674	0.0552	0.0026	5.70E-96
FARS2	6:5258730	-0.221	0.0253	3.18E-18
FARSA	19:13073068	0.0379	0.00621	1.08E-09
FARSB	2:223528484	-0.135	0.012	7.86E-29
FAS	10:90751516	-0.104	0.00486	1.81E-97
FASTK	7:150750980	0.0231	0.00291	2.36E-15
FASTKD1	2:170429975	-0.0816	0.00572	2.38E-45
FASTKD3	5:7860404	-0.0708	0.0045	1.63E-54
FAT2	5:150838293	0.0125	0.00198	2.79E-10
FBLN5	14:92339850	0.0397	0.00264	4.66E-50
FBN1	15:48934858	0.04	0.00222	1.68E-70
FBN2	5:127752157	-0.28	0.00697	<1.0E-314
FBXL13	7:102618968	-0.75	0.0325	2.82E-112
FBXL17	5:107574367	0.0263	0.00282	1.80E-20
FBXL18	7:5511470	-0.0454	0.00717	2.66E-10
FBXL4	6:99433539	0.038	0.0036	9.95E-26
FBXL5	4:15665083	0.0568	0.00473	8.11E-33
FBXO10	9:37598317	0.118	0.0072	7.21E-59
FBXO15	18:71815063	0.0488	0.0071	6.63E-12
FBXO18	10:5945672	-0.0253	0.00276	8.84E-20
FBXO28	1:224315198	-0.0316	0.00465	1.22E-11
FBXO3	11:33792977	-0.065	0.00381	1.22E-63
FBXO30	6:146177815	0.0548	0.00735	9.98E-14
FBXO32	8:124525377	0.0541	0.00881	8.54E-10
FBXO33	14:39884876	-0.0245	0.00348	2.16E-12
FBXO34	14:55811327	0.0329	0.00354	2.37E-20
FBXO42	1:16590230	0.0602	0.00371	1.16E-57

FBXO7	22:32900216	0.0675	0.00727	2.27E-20
FBXW2	9:123517704	-0.0333	0.00334	3.91E-23
FBXW8	12:117330844	-0.175	0.00472	2.10E-267
FCAR	19:55388245	-0.15	0.00965	1.52E-53
FCER1G	1:161187665	-0.204	0.00655	4.03E-196
FCGBP	19:40406203	0.12	0.00611	3.75E-83
FCGR1A	1:149788657	-0.355	0.0322	5.07E-28
FCGR2A	1:161479745	0.0472	0.00425	2.47E-28
FCGR2B	1:161623934	0.234	0.018	4.27E-38
FCGRT	19:50042867	0.105	0.0131	1.80E-15
FCHO1	19:17856803	0.0406	0.00371	1.49E-27
FCHO2	5:72399896	0.12	0.00741	2.10E-57
FCN1	9:137808961	-0.192	0.00522	6.37E-263
FCN2	9:137809861	-0.0188	0.00304	7.11E-10
FCRL1	1:157787253	-0.116	0.0092	4.76E-36
FCRL3	1:157669278	0.165	0.00585	6.27E-164
FCRL5	1:157528999	-0.276	0.00703	3.04E-295
FCRLB	1:161703992	0.0443	0.00706	3.95E-10
FDFT1	8:11666451	0.128	0.00385	7.66E-220
FDX1	11:110275001	-0.0449	0.0051	1.93E-18
FECH	18:55238820	0.265	0.0253	2.27E-25
FEM1A	19:4775602	0.0408	0.00479	2.23E-17
FEM1C	5:114896050	0.0663	0.00315	1.53E-94
FERMT3	11:63963947	0.0312	0.00361	6.72E-18
FEZ2	2:36781708	-0.0738	0.00354	9.63E-93
FGD2	6:36974607	0.0449	0.00616	3.45E-13
FGD3	9:95787579	-0.0936	0.00573	1.51E-58
FGD4	12:32606149	-0.0397	0.00642	6.63E-10
FGD6	12:95473185	0.0995	0.00821	2.17E-33
FGF2	4:123724239	0.0465	0.006	1.13E-14
FGFBP2	4:15961555	0.166	0.00819	8.50E-88
FGFR1	8:38340154	0.0431	0.00235	5.68E-73
FGFR1OP	6:167497487	-0.245	0.0141	1.12E-65
FGFR2	10:123197653	0.17	0.00389	<1.0E-314
FGGY	1:59813880	-0.0691	0.00603	4.64E-30
FGL2	7:76787832	0.0986	0.0147	2.04E-11
FHIT	3:59996668	0.263	0.0244	7.18E-27
FHL2	2:105997385	-0.0323	0.00503	1.61E-10
FHL3	1:38465315	-0.322	0.0178	6.68E-71

FIG4	6:110051141	0.0436	0.0035	5.31E-35
FIGNL1	7:50515627	-0.0862	0.00472	3.27E-72
FIS1	7:100892196	0.175	0.0291	2.05E-09
FKBP1A	20:1323773	-0.0417	0.00593	2.14E-12
FKTN	9:108347398	0.0591	0.00415	3.67E-45
FLCN	17:17172160	0.0476	0.00312	1.83E-51
FLJ13197	4:38641936	-0.202	0.00638	2.72E-202
FLJ37201	10:91401407	-0.0581	0.00695	8.36E-17
FLJ40852	7:141459327	-0.0514	0.00375	4.78E-42
FLNB	3:58080960	0.0815	0.00963	3.27E-17
FLOT1	6:30756882	0.0927	0.00454	3.09E-89
FLOT2	17:27208589	-0.0455	0.00473	1.04E-21
FLT1	13:29039871	0.0477	0.00441	6.06E-27
FLT3	13:28697891	-0.467	0.053	1.63E-18
FLVCR1	1:213035671	-0.139	0.00515	5.09E-150
FLVCR1-AS1	1:213010589	-0.253	0.00579	<1.0E-314
FLVCR2	14:76085273	-0.045	0.00385	3.49E-31
FLYWCH1	16:2986323	0.123	0.00489	2.97E-132
FLYWCH2	16:2955700	0.0452	0.00607	1.17E-13
FMNL2	2:153416838	-0.0298	0.0046	1.10E-10
FMNL3	12:50063768	0.0352	0.00491	8.41E-13
FMO5	1:146678399	-0.0679	0.00969	2.81E-12
FN1	2:216214791	0.0258	0.00239	5.13E-27
FN3K	17:80696692	0.224	0.00709	3.14E-200
FN3KRP	17:80667812	0.134	0.00922	7.29E-47
FNBP1	9:132657208	0.0245	0.00375	7.62E-11
FNBP1L	1:93911011	-0.482	0.0141	6.59E-231
FNDC3A	13:49593015	-0.0969	0.0122	2.31E-15
FNDC5	1:33319277	0.0308	0.00514	2.23E-09
FNTB	14:65500149	-0.0535	0.00629	2.36E-17
FOCAD	9:20652462	-0.0635	0.00931	1.04E-11
FOLR3	11:71844701	-1.92	0.0425	<1.0E-314
FOPNL	16:15975829	0.0371	0.00495	7.43E-14
FOXJ2	12:8210637	0.0271	0.0032	3.51E-17
FOXK2	17:80551319	-0.14	0.019	1.66E-13
FOXN2	2:48554955	-0.103	0.00619	8.45E-61
FOXN3	14:89805934	-0.0256	0.0039	6.13E-11
FOXN3-AS2	14:90043381	-0.157	0.00991	5.73E-55
FOXQ1	6:1286450	-0.0274	0.00345	2.69E-15

FOXRED1	11:126148432	0.0363	0.00343	6.81E-26
FOXRED2	22:36901622	-0.0437	0.00587	1.22E-13
FPGT	1:74667686	0.153	0.00416	4.25E-262
FPR1	19:52276665	0.0809	0.00509	1.07E-55
FPR2	19:52276665	0.237	0.00679	1.22E-240
FPR3	19:52270939	-0.118	0.0139	1.90E-17
FRA10AC1	10:95422045	0.227	0.00562	<1.0E-314
FRAT1	10:99100432	-0.0848	0.0055	1.61E-52
FRAT2	10:99115683	-0.0487	0.0037	6.01E-39
FRMD3	9:86079180	0.0716	0.00772	2.35E-20
FRMD4A	10:13747195	0.0234	0.0032	3.40E-13
FRS2	12:69908429	-0.0871	0.0105	1.84E-16
FRY	13:32683295	-0.107	0.0116	4.04E-20
FSD1L	9:108210212	0.0718	0.00643	1.16E-28
FST	5:52778262	-0.389	0.0204	9.59E-79
FTH1	11:61731727	0.245	0.0191	3.01E-37
FTO	16:53873977	0.0429	0.00693	6.28E-10
FTSJ3	17:61913214	0.0335	0.00383	2.82E-18
FTSJD1	16:71292172	-0.0394	0.00327	6.94E-33
FUBP1	1:78453699	-0.0492	0.00666	1.72E-13
FUCA1	1:24201919	0.244	0.00989	4.05E-127
FUCA2	6:143825104	-0.0309	0.00493	4.03E-10
FUT10	8:33276407	-0.265	0.00657	<1.0E-314
FUT2	19:49201217	0.0405	0.00552	2.70E-13
FUT7	9:139933589	-0.228	0.0176	1.40E-37
FUT8	14:65860092	0.0399	0.00445	4.03E-19
FXN	9:71690963	-0.0625	0.0048	4.11E-38
FXYD5	19:35677210	-0.119	0.00448	6.61E-147
FXYD6	11:117708625	0.0664	0.00715	2.47E-20
FYB	5:39116820	-0.0571	0.00448	8.94E-37
FYCO1	3:45963497	-0.0256	0.00291	2.00E-18
FYN	6:112099797	-0.034	0.00417	4.36E-16
FYTTD1	3:197498635	-0.0876	0.00563	2.55E-53
FZD3	8:28436650	0.0818	0.00687	2.56E-32
FZD5	2:208629381	0.0858	0.00602	3.42E-45
FZD6	8:104345778	0.12	0.00428	1.44E-160
G0S2	1:209824899	0.098	0.0114	8.99E-18
G3BP2	4:76564611	0.0254	0.0026	2.62E-22
GAA	17:78064064	-0.0521	0.00261	1.48E-85

GAB2	11:77948095	-0.0909	0.00602	1.58E-50
GABARAPL1	12:10381873	0.11	0.0127	7.43E-18
GABBR1	6:29626154	0.11	0.00901	1.13E-33
GABPB1	15:50585337	0.0254	0.0037	7.08E-12
GABPB2	1:151028929	-0.0553	0.0058	2.50E-21
GABRB1	4:47413121	0.022	0.00324	1.26E-11
GABRG1	4:46099034	-0.2	0.0221	1.73E-19
GABRR2	6:90032082	-0.051	0.00412	9.18E-35
GAD1	2:171678269	-0.103	0.00915	3.17E-29
GAL	11:68402220	-0.0662	0.00781	3.14E-17
GALC	14:88459150	0.0707	0.00451	3.54E-54
GALK2	15:49614081	0.0462	0.00361	6.54E-37
GALNS	16:88913946	-0.0257	0.00262	1.38E-22
GALNT1	18:33188845	0.045	0.00405	2.09E-28
GALNT10	5:153552873	0.0433	0.00468	3.21E-20
GALNT14	2:31235581	0.0397	0.00311	1.11E-36
GALNT4	12:89907166	0.0429	0.00559	1.90E-14
GALNT5	2:158117683	0.227	0.0301	4.71E-14
GALNT6	12:51785401	-0.057	0.00289	2.43E-83
GALNT7	4:174092791	-0.0344	0.00339	5.07E-24
GALNT8	12:4781448	-0.0493	0.00793	5.56E-10
GART	21:34898740	0.0218	0.00359	1.48E-09
GAS2	11:22682639	-0.0655	0.00616	3.85E-26
GAS7	17:9815187	-0.115	0.00557	1.99E-91
GAS8	16:90149077	-0.0685	0.0111	8.61E-10
GATAD1	7:92097529	0.0558	0.00541	1.05E-24
GATAD2A	19:19605763	-0.0884	0.0026	1.75E-228
GATC	12:120902279	-0.0255	0.00429	2.85E-09
GATM	15:45633270	0.126	0.00579	1.47E-100
GATS	7:99809835	-0.0538	0.00442	1.08E-33
GBA	1:155214066	-0.0466	0.00467	3.39E-23
GBA2	9:35752252	-0.0506	0.00288	5.67E-67
GBAP1	1:155214473	0.172	0.00491	6.31E-242
GBAS	7:56033558	0.0364	0.00503	5.27E-13
GBE1	3:81769493	0.0527	0.00489	7.11E-27
GBP3	1:89456659	0.225	0.00552	<1.0E-314
GBP4	1:89704458	0.0651	0.00978	3.10E-11
GBP5	1:89742521	0.0882	0.0113	6.71E-15
GCAT	22:38205060	-0.104	0.00461	4.51E-108

GCC2	2:109038026	0.237	0.00435	<1.0E-314
GCFC2	2:75893853	-0.0473	0.00391	3.43E-33
GCH1	14:55359653	0.111	0.00763	4.44E-47
GCLM	1:94372009	0.0521	0.00609	1.69E-17
GCM1	6:53050717	0.0564	0.00537	1.53E-25
GCNT2	6:10534503	0.196	0.00501	7.72E-294
GCNT4	5:74349933	0.231	0.019	2.08E-33
GCOM1	15:57855677	-0.299	0.0057	<1.0E-314
GCSAM	3:111860222	0.0563	0.00598	7.35E-21
GDAP1	8:75272727	-0.0661	0.00584	2.36E-29
GDE1	16:19505990	0.0985	0.00804	5.31E-34
GDI2	10:5855403	0.103	0.00392	1.90E-143
GDPD3	16:30155290	0.113	0.00935	5.48E-33
GDPD5	11:75215421	-0.025	0.00226	4.39E-28
GEN1	2:17974041	0.0355	0.00571	5.49E-10
GET4	7:867405	0.0179	0.00298	1.89E-09
GFER	16:2037108	0.0683	0.00508	1.47E-40
GFM2	5:74081943	0.0493	0.00403	6.86E-34
GFPT1	2:69598243	-0.0773	0.00326	3.37E-118
GGACT	13:101241720	-0.215	0.0244	1.50E-18
GGCT	7:30577041	-0.0281	0.00458	9.13E-10
GGCX	2:85759588	-0.039	0.00285	6.62E-42
GGH	8:63884089	-0.0388	0.00557	3.88E-12
GGNBP2	17:34866915	0.0596	0.00282	3.23E-95
GGPS1	1:235451482	-0.186	0.00617	6.27E-185
GGTA1P	9:124264116	0.374	0.01	2.92E-269
GHITM	10:85898172	-0.102	0.00732	3.06E-43
GHRL	3:10320865	-0.0582	0.00526	4.12E-28
GHRLOS2	3:10314602	-0.196	0.00584	9.94E-224
GIF	11:59567968	-0.127	0.0128	5.32E-23
GIGYF2	2:233765133	0.0264	0.00449	4.56E-09
GIMAP1	7:150408032	0.0749	0.00647	1.25E-30
GIMAP2	7:150389677	0.0841	0.005	6.71E-62
GIMAP4	7:150254030	0.149	0.0104	1.16E-45
GIMAP5	7:150430711	-0.205	0.00468	<1.0E-314
GIMAP6	7:150335490	0.0509	0.00399	1.25E-36
GIMAP7	7:150215280	0.148	0.00901	5.21E-59
GIMAP8	7:150153813	-0.0448	0.00476	6.85E-21
GIN1	5:102391465	0.0843	0.00496	4.84E-63

GINM1	6:149897455	-0.102	0.00622	1.42E-58
GINS1	20:25387465	0.038	0.00515	1.94E-13
GIPC2	1:78478362	-0.0399	0.00591	1.53E-11
GIT1	17:27904711	0.0553	0.00297	5.43E-75
GIT2	12:110473247	-0.111	0.00777	3.15E-45
GJA9	1:39325063	0.0228	0.00371	9.31E-10
GK3P	4:166237058	-0.143	0.0148	4.84E-22
GK5	3:141932531	0.0398	0.00675	4.22E-09
GKAP1	9:86394466	0.0581	0.00708	2.82E-16
GLB1L	2:220113728	0.169	0.00743	2.13E-109
GLCCI1	7:8067512	0.0831	0.00754	6.03E-28
GLCE	15:69548338	-0.195	0.0158	1.84E-34
GLE1	9:131308732	0.0919	0.0135	1.31E-11
GLI1	12:57870155	-0.0302	0.00251	7.09E-33
GLIPR1	12:75871264	-0.116	0.00524	5.83E-104
GLIPR1L1	12:75799521	-0.0769	0.0051	2.67E-50
GLIPR1L2	12:75784773	0.152	0.00505	4.84E-183
GLO1	6:38652456	-0.0633	0.0044	3.98E-46
GLRX	5:95149966	-0.148	0.00816	5.26E-71
GLRX3	10:131934596	0.0899	0.00418	1.89E-98
GLRX5	14:96009307	0.0424	0.00595	1.18E-12
GLS	2:191836562	0.0316	0.00335	5.04E-21
GLT1D1	12:129360928	0.0816	0.00796	2.07E-24
GLT25D1	19:17654964	0.0374	0.0042	6.93E-19
GLT25D2	1:183937280	0.127	0.00367	3.24E-236
GLTP	12:110297809	-0.0443	0.00543	4.23E-16
GLTSCR1	19:48188809	0.0288	0.00239	4.59E-33
GLTSCR2	19:48258717	-0.0311	0.0041	4.22E-14
GLUL	1:182377078	0.0479	0.00481	4.31E-23
GLYATL2	11:58638918	-1.11	0.0293	5.99E-280
GLYR1	16:4883781	-0.025	0.00281	7.08E-19
GM2A	5:150597378	-0.411	0.00898	<1.0E-314
GMDS	6:2232808	-0.19	0.0148	3.78E-37
GMEB1	1:29003360	-0.0526	0.00694	4.04E-14
GNA12	7:2755237	-0.116	0.00537	6.17E-100
GNA15	19:3155902	-0.0822	0.00732	6.06E-29
GNAI1	7:79766896	-0.0615	0.00787	6.36E-15
GNAI3	1:110114611	0.038	0.00398	2.06E-21
GNAO1	16:56254646	0.113	0.0167	1.50E-11

GNAQ	9:80630123	0.0319	0.00507	3.41E-10
GNAS	20:57478807	-0.0402	0.00522	1.58E-14
GNAZ	22:23455366	0.0306	0.00455	1.90E-11
GNB4	3:179166083	0.0375	0.00433	6.82E-18
GNB5	15:52452261	-0.0931	0.00767	1.85E-33
GNE	9:36253826	-0.029	0.0044	5.19E-11
GNG11	7:93551428	0.214	0.0124	5.78E-65
GNG2	14:52421031	0.052	0.00511	4.24E-24
GNG5	1:84953765	-0.0717	0.0107	2.18E-11
GNG8	19:47145973	0.103	0.011	1.34E-20
GNL3	3:52687566	-0.0248	0.00356	3.53E-12
GNLY	2:85933003	-0.399	0.0119	5.68E-224
GNPAT	1:231408091	0.0387	0.00419	3.51E-20
GNPDA2	4:44706919	-0.0391	0.00484	8.73E-16
GNRH1	8:25280800	0.0796	0.00557	2.31E-45
GNRHR	4:68579924	-0.282	0.00736	1.03E-283
GNS	12:65153273	0.0544	0.00451	4.18E-33
GOLGA1	9:127603814	0.0223	0.00334	2.84E-11
GOLGA3	12:133407523	-0.0699	0.00294	9.55E-119
GOLGA5	14:93322590	-0.114	0.00944	4.77E-33
GOLGA6L9	15:82691423	-0.258	0.0328	4.38E-15
GOLGA7	8:41352161	-0.0854	0.00392	1.13E-100
GOLGA8B	15:34890377	0.721	0.041	2.61E-67
GOLIM4	3:167838679	-0.0488	0.00513	2.93E-21
GOLM1	9:88692049	0.0834	0.00495	4.78E-62
GOLPH3L	1:150709785	-0.0537	0.00517	4.31E-25
GOLT1B	12:21626944	-0.0453	0.00488	2.26E-20
GORAB	1:170507904	0.0992	0.00598	2.33E-60
GOSR1	17:28825756	-0.0448	0.00568	3.80E-15
GP6	19:55501866	-0.0725	0.00452	1.83E-56
GPAM	10:113908060	-0.0296	0.00338	2.62E-18
GPANK1	6:31632808	-0.122	0.0163	6.34E-14
GPATCH1	19:33604175	0.0376	0.00417	3.07E-19
GPATCH2L	14:76606406	-0.0458	0.00342	3.22E-40
GPBP1L1	1:46178112	0.0383	0.00334	4.69E-30
GPCPD1	20:5626343	-0.07	0.00485	2.43E-46
GPD1L	3:32204518	0.0808	0.00406	3.74E-85
GPD2	2:157305763	-0.136	0.00423	2.37E-207
GPHN	14:67009600	0.0816	0.0135	1.64E-09

GPI	19:34919940	-0.052	0.00759	8.47E-12
GPLD1	6:24491341	0.0378	0.00284	1.33E-39
GPM6A	4:176935634	0.105	0.00682	4.46E-52
GPN2	1:27182984	-0.0181	0.00301	2.06E-09
GPN3	12:110857694	0.184	0.00549	2.33E-222
GPNMB	7:23278283	0.0586	0.00336	3.91E-66
GPR108	19:6737063	-0.0375	0.00556	1.77E-11
GPR114	16:57578966	0.0187	0.00302	6.69E-10
GPR126	6:142753338	0.0239	0.00262	8.66E-20
GPR128	3:100321173	-1.05	0.0244	<1.0E-314
GPR133	12:131467623	0.141	0.0124	8.90E-30
GPR135	14:59980614	-0.0315	0.00272	1.09E-30
GPR137B	1:236312326	-0.119	0.0074	4.75E-57
GPR141	7:37732623	-0.124	0.00877	1.59E-44
GPR146	7:1063593	-0.382	0.0152	7.42E-132
GPR155	2:175354156	0.0938	0.00604	3.60E-53
GPR160	3:169765795	-0.195	0.00875	1.96E-105
GPR162	12:6901077	-0.0913	0.0043	8.19E-96
GPR180	13:95253920	0.153	0.0044	1.30E-239
GPR22	7:107127496	-0.118	0.00861	2.76E-42
GPR52	1:174423131	-0.0967	0.00897	7.72E-27
GPR63	6:97294970	-0.1	0.00905	4.55E-28
GPR65	14:88426154	0.0475	0.00523	1.50E-19
GPR83	11:94136161	-0.0312	0.00458	1.13E-11
GPR97	16:57713536	-0.103	0.00814	7.66E-36
GPRC5C	17:72425499	-0.113	0.00803	2.34E-44
GPRC5D	12:13091286	-0.0469	0.00765	9.48E-10
GPX2	14:65404442	0.0958	0.00623	2.63E-52
GPX3	5:150403098	0.0461	0.00516	5.53E-19
GPX4	19:1103465	-0.099	0.00459	9.32E-99
GPX7	1:53073556	-0.0396	0.00382	6.25E-25
GRAMD1A	19:35490861	0.0325	0.00233	1.98E-43
GRAMD1B	11:123355391	-0.0356	0.00603	3.76E-09
GRAMD1C	3:113557690	0.257	0.0111	6.25E-112
GRAMD4	22:47007378	-0.0337	0.00435	1.01E-14
GRAP	17:18924188	0.162	0.019	1.66E-17
GRB10	7:50817513	-0.0472	0.00703	2.15E-11
GRB14	2:165401801	0.13	0.00734	7.63E-68
GRB2	17:73271758	0.0361	0.00395	9.53E-20

GRHL1	2:10110019	0.027	0.00368	2.47E-13
GRHPR	9:37422656	-0.162	0.00456	4.93E-247
GRIK1	21:30943987	-0.0252	0.00414	1.27E-09
GRIK4	11:120779520	-0.0614	0.00343	1.04E-69
GRIN3A	9:104339000	-0.0249	0.00407	9.26E-10
GRINA	8:145070403	0.163	0.00728	3.43E-106
GRK4	4:2954717	-0.104	0.00728	3.79E-45
GRK5	10:121064720	-0.0289	0.00374	1.35E-14
GRK6	5:176857270	-0.0285	0.00362	4.49E-15
GRN	17:42398802	-0.0813	0.00823	8.57E-23
GSDMB	17:38020058	0.324	0.00655	<1.0E-314
GSK3B	3:119850315	-0.0468	0.00398	1.38E-31
GSKIP	14:96842676	-0.174	0.0065	1.00E-148
GSN	9:124034809	-0.0497	0.00213	1.25E-114
GSPT1	16:12021486	0.315	0.0445	1.84E-12
GSR	8:30560887	-0.0454	0.00607	8.93E-14
GSTA1	6:52658191	-0.135	0.00923	2.52E-47
GSTA3	6:52712691	-0.234	0.0209	1.02E-28
GSTA4	6:52849146	0.0375	0.00475	3.34E-15
GSTCD	4:106664211	-0.0386	0.00407	3.67E-21
GSTK1	7:142923787	0.113	0.00827	8.36E-42
GSTM1	1:110230073	0.982	0.0359	2.90E-154
GSTM2	1:110218486	-0.603	0.0318	1.43E-77
GSTM3	1:110247164	0.209	0.00516	<1.0E-314
GSTM4	1:110172362	0.463	0.00882	<1.0E-314
GSTM5	1:110254396	0.124	0.00694	6.67E-69
GSTP1	11:67352689	0.213	0.00639	2.43E-220
GSTT1	22:24366720	0.554	0.0129	<1.0E-314
GTF2A1	14:81701004	0.04	0.00379	9.22E-26
GTF2A2	15:59923729	0.0834	0.00621	1.68E-40
GTF2E2	8:30501232	-0.0383	0.00405	4.57E-21
GTF2F2	13:45694139	-0.208	0.00834	9.41E-130
GTF2H1	11:18357270	-0.0314	0.00405	1.09E-14
GTF2H4	6:30865386	-0.126	0.0131	8.09E-22
GTF2H5	6:158591478	0.31	0.0365	2.43E-17
GTF3A	13:27997305	0.0248	0.00381	8.76E-11
GTF3C3	2:197664437	0.122	0.017	7.59E-13
GTPBP10	7:89976025	0.223	0.0164	1.34E-41
GTPBP4	10:1075488	-0.0267	0.00389	7.91E-12

GTPBP5	20:60766599	-0.0207	0.0031	2.55E-11
GTSF1	12:54818211	-0.215	0.0218	6.64E-23
GUCD1	22:24938455	-0.0707	0.00902	5.60E-15
GUCY1B2	13:51631607	-0.0515	0.00328	3.21E-54
GUCY2C	12:14721568	-0.0868	0.00347	8.11E-131
GUF1	4:44727151	0.0849	0.00363	4.02E-115
GULP1	2:189365907	-0.0392	0.00543	6.30E-13
GUSB	7:65416536	-0.069	0.0052	1.29E-39
GUSBP5	4:144443295	-0.119	0.00417	9.42E-167
GXYLT1	12:42516376	0.0499	0.00594	6.03E-17
GYPA	4:145046566	-0.134	0.0185	3.97E-13
GYPB	4:144978172	-0.299	0.029	1.33E-24
GYPC	2:127404215	0.083	0.00913	1.32E-19
GYPE	4:144784844	0.417	0.0265	1.13E-54
GZMB	14:25100933	0.0893	0.0105	2.89E-17
GZMK	5:54317900	0.0981	0.0121	4.97E-16
H1F0	22:38199222	-0.266	0.00735	1.16E-256
H1FNT	12:48715162	-0.0243	0.00379	1.59E-10
H2AFJ	12:14958051	-0.0468	0.00421	2.00E-28
H2AFV	7:44910667	-0.077	0.00581	1.83E-39
H2AFZ	4:100836788	0.0469	0.0077	1.24E-09
H3F3B	17:73773000	-0.0756	0.00699	5.54E-27
HABP4	9:99212378	0.0599	0.00494	2.27E-33
HACE1	6:105142061	0.037	0.0043	9.26E-18
HADH	4:108955622	0.0404	0.00548	1.78E-13
HADHA	2:26424583	-0.078	0.00469	1.94E-60
HAGH	16:1906352	0.155	0.0134	8.86E-31
HAL	12:96388182	-0.119	0.0058	2.91E-89
HAT1	2:172813241	0.0604	0.005	3.70E-33
HAUS3	4:2234875	-0.0391	0.00651	1.90E-09
HAUS4	14:23375251	0.319	0.00643	<1.0E-314
HAUS8	19:17211566	-0.0432	0.00585	1.77E-13
HAVCR1	5:156515406	0.0727	0.00835	4.04E-18
HBE1	11:5239643	-0.0598	0.00978	1.08E-09
HBM	16:216420	-0.567	0.0536	6.06E-26
HBQ1	16:264642	-0.049	0.00506	5.62E-22
HBS1L	6:135323203	0.0626	0.00346	6.62E-71
HBZ	16:209950	-1.39	0.0414	2.04E-223
HCAR2	12:123199826	-0.0967	0.00737	9.59E-39

HCAR3	12:123200099	0.191	0.00737	4.83E-140
HCG27	6:31175667	0.0797	0.00554	4.38E-46
HCG4	6:29748308	0.0622	0.00487	8.14E-37
HCG9	6:29934109	0.1	0.00735	7.73E-42
HCK	20:30640593	0.0456	0.00561	5.20E-16
HCLS1	3:121411876	0.0459	0.00351	1.75E-38
HCP5	6:31431691	-0.132	0.00896	1.90E-48
HDAC4	2:240264555	-0.0113	0.00186	1.27E-09
HDDC2	6:125596957	-0.127	0.00441	8.62E-171
HDGF	1:156744737	0.107	0.0139	1.55E-14
HDGFRP3	15:83852620	-0.241	0.0102	4.77E-117
HDHD2	18:44709035	-0.145	0.0105	1.10E-42
HDHD3	9:116141399	0.189	0.0259	3.20E-13
HDLBP	2:242176429	0.0275	0.00426	1.24E-10
HEATR1	1:236714747	-0.0377	0.00357	6.68E-26
HEATR3	16:50089132	0.107	0.00338	2.10E-202
HEATR6	17:58167485	-0.164	0.0049	2.46E-222
HEBP1	12:13116794	-0.301	0.00748	<1.0E-314
HEBP2	6:138725857	0.169	0.00825	4.26E-90
HECTD2	10:93172388	-0.0791	0.00646	5.21E-34
HECTD3	1:45491504	-0.0237	0.00358	3.94E-11
HEG1	3:124821561	-0.129	0.0209	7.76E-10
HELB	12:66756215	-0.0496	0.00394	8.29E-36
HELLS	10:96382128	0.138	0.0045	1.72E-189
HELQ	4:84394682	-0.0261	0.00421	6.33E-10
HEMGN	9:100720053	-0.0845	0.00997	2.99E-17
HEMK1	3:50630221	0.0583	0.00464	9.92E-36
HERC1	15:64001876	-0.0179	0.00302	3.47E-09
HERC2	15:28419974	-0.255	0.00632	<1.0E-314
HERC3	4:89648845	0.0413	0.00489	4.11E-17
HERC4	10:69872209	0.0773	0.00999	1.21E-14
HERPUD1	16:56944947	0.037	0.0059	3.89E-10
HERPUD2	7:35756797	0.0202	0.00316	2.00E-10
HEXB	5:73937854	-0.0737	0.00621	5.41E-32
HEXIM1	17:43226477	0.0302	0.00324	1.96E-20
HEY1	8:80723932	-0.1	0.00735	1.24E-41
HGS	17:79642731	0.0325	0.00505	1.25E-10
HGSNAT	8:43091271	-0.0883	0.0148	2.35E-09
HHAT	1:210502184	-0.0366	0.00473	1.16E-14

HHEX	10:94432000	0.0423	0.00406	3.88E-25
HIAT1	1:100543962	0.0443	0.00637	3.79E-12
HIATL1	9:97094721	-0.956	0.0258	4.03E-267
HIBADH	7:27619594	-0.0515	0.00413	3.58E-35
HIBCH	2:191193094	0.0659	0.00592	1.96E-28
HIF1A	14:62139577	0.0739	0.0119	6.11E-10
HIF1AN	10:102298855	0.0757	0.00563	1.49E-40
HIGD1B	17:42927721	0.0507	0.00502	9.70E-24
HIGD2A	5:175810837	0.199	0.0117	8.35E-63
HILPDA	7:128054100	-0.0306	0.00471	8.41E-11
HINT1	5:130501557	0.323	0.0193	4.54E-61
HIP1	7:75245141	0.377	0.00623	<1.0E-314
HIST1H1D	6:26229684	-0.133	0.016	8.41E-17
HIST1H2AB	6:26033828	-0.236	0.0177	1.01E-39
HIST1H2AC	6:26124634	-0.083	0.00722	3.13E-30
HIST1H2AE	6:26221537	0.162	0.014	1.08E-30
HIST1H2BD	6:26171250	0.135	0.0071	2.93E-78
HIST1H2BF	6:26198845	-0.216	0.00593	7.37E-259
HIST1H2BG	6:26234476	-0.21	0.0173	2.61E-33
HIST1H2BJ	6:27080460	0.0368	0.00626	4.40E-09
HIST1H2BK	6:27137634	-0.16	0.0177	2.52E-19
HIST1H3B	6:26028819	0.176	0.0105	4.63E-61
HIST1H3D	6:26190609	0.0924	0.00947	2.66E-22
HIST1H3E	6:26175832	-0.422	0.0102	<1.0E-314
HIST1H3I	6:27839746	0.216	0.0145	2.11E-49
HIST1H4B	6:26030710	0.331	0.0119	2.52E-159
HIST1H4C	6:26118570	0.223	0.00971	9.10E-111
HIST1H4D	6:26204506	0.0876	0.0117	7.05E-14
HIST1H4E	6:26229684	0.134	0.0167	1.09E-15
HIST1H4F	6:26242777	-0.0885	0.00775	8.05E-30
HIST1H4H	6:26276650	-0.179	0.00656	4.48E-153
HIST1H4L	6:27840926	0.493	0.023	1.78E-97
HIST2H2BF	1:149772497	0.425	0.0424	2.02E-23
HIST4H4	12:14966165	-0.0426	0.00589	5.77E-13
HIVEP1	6:12127054	0.0498	0.00732	1.18E-11
HK1	10:71001110	-0.0365	0.00557	6.09E-11
HK2	2:75085163	-0.0421	0.00405	5.03E-25
HKDC1	10:70979924	0.0394	0.00273	1.81E-46
HKR1	19:37839871	0.0429	0.00368	5.63E-31

HLA-B	6:31325235	-0.263	0.00741	1.09E-246
HLA-DMA	6:32961104	0.0424	0.0071	2.55E-09
HLA-DMB	6:32904601	0.0647	0.0069	1.07E-20
HLA-DOA	6:32974401	-0.027	0.00367	2.07E-13
HLA-DOB	6:32796856	-0.601	0.017	8.89E-246
HLA-DPA1	6:33025107	0.387	0.00794	<1.0E-314
HLA-DPB1	6:33047031	0.401	0.00723	<1.0E-314
HLA-DPB2	6:33096708	0.0932	0.00546	1.03E-63
HLA-DQA2	6:32668100	-0.254	0.00776	4.12E-213
HLA-DQB2	6:32678206	-0.112	0.00848	1.79E-39
HLA-DRA	6:32411035	0.0571	0.00456	1.90E-35
HLA-DRB1	6:32569859	-5.43	0.0438	<1.0E-314
HLA-DRB5	6:32436217	-2.58	0.0569	<1.0E-314
HLA-E	6:30457732	0.048	0.00402	1.65E-32
HLA-F	6:29691090	0.127	0.00452	3.95E-161
HLA-L	6:30228879	0.0835	0.00516	1.57E-57
HLCS	21:38330036	-0.062	0.00329	6.66E-77
HLTF	3:148761815	0.0558	0.00384	4.94E-47
HM13	20:30164874	-0.086	0.00336	4.70E-136
HMBOX1	8:28908874	-0.0352	0.00389	1.92E-19
HMCN1	1:185998333	-0.0377	0.00586	1.28E-10
HMG20A	15:77723027	-0.0415	0.00399	3.46E-25
HMGN3	6:79954685	0.0644	0.00993	9.31E-11
HMGN4	6:26538543	0.0894	0.00625	1.81E-45
HMHA1	19:1062598	-0.0354	0.00407	4.20E-18
HMHB1	5:143182261	-0.147	0.0168	3.50E-18
HMMR	5:162912162	0.0584	0.00587	4.56E-23
HMOX1	22:35806238	0.0825	0.00696	5.45E-32
HMOX2	16:4576423	-0.0488	0.00406	6.77E-33
HNMT	2:138792443	-0.0588	0.00616	1.99E-21
HNRNPA1	12:54685880	-0.137	0.0142	8.55E-22
HNRPLL	2:38868395	0.0816	0.0124	5.82E-11
HOOK1	1:60261892	0.174	0.0152	4.69E-30
HORMAD1	1:150721175	-0.155	0.00627	3.82E-128
HOXA1	7:27141657	-0.0281	0.00443	2.34E-10
HOXA2	7:27093099	-0.251	0.00617	<1.0E-314
HOXA3	7:27102949	-0.0256	0.00271	4.94E-21
HOXA9	7:27200855	0.0536	0.00616	4.13E-18
HOXB2	17:46581184	0.103	0.00371	1.84E-157

HOXB3	17:46602432	-0.0569	0.00326	2.02E-66
HP	16:72118324	0.409	0.0129	7.75E-202
HP1BP3	1:21146422	-0.0441	0.00353	2.45E-35
HPCAL1	2:10552641	-0.0233	0.00271	1.12E-17
HPCAL4	1:40150156	-0.0955	0.00495	3.80E-80
HPGD	4:175361437	0.0796	0.00509	4.90E-54
HPGDS	4:95228540	-0.416	0.0406	1.92E-24
HPR	16:72106024	-0.141	0.00858	2.77E-59
HPS1	10:100218195	0.0431	0.00269	1.56E-56
HPS3	3:148806754	-0.0729	0.00764	2.25E-21
HPS4	22:26924995	0.0264	0.00276	1.54E-21
HPS5	11:18353640	-0.0337	0.00351	1.18E-21
HPSE	4:84237883	0.274	0.0204	2.63E-40
HRAS	11:545276	0.0492	0.00533	3.81E-20
HRSP12	8:99126359	-0.137	0.00668	5.71E-90
HS2ST1	1:87364781	0.0446	0.00602	1.54E-13
HSBP1	16:83800282	0.0511	0.00507	1.07E-23
HSD17B11	4:88286409	-0.0599	0.00505	4.99E-32
HSD17B12	11:43870871	-0.0905	0.00635	3.04E-45
HSD17B13	4:88176030	-0.277	0.00623	<1.0E-314
HSD17B3	9:99052366	-0.0329	0.00512	1.49E-10
HSD17B4	5:118771423	-0.141	0.0103	7.06E-42
HSD17B7P2	10:38595712	-0.698	0.0125	<1.0E-314
HSD17B8	6:33140009	-0.0439	0.00536	3.26E-16
HSDL1	16:84109997	-0.0599	0.00496	3.71E-33
HSDL2	9:115118961	0.0731	0.00598	6.66E-34
HSF2	6:122721510	0.0501	0.00438	5.79E-30
HSH2D	19:16268664	0.0659	0.00933	1.83E-12
HSP90AA1	14:102550569	0.163	0.00742	5.35E-102
HSPA1B	6:31785228	-0.05	0.00559	5.07E-19
HSPA1L	6:31809504	0.14	0.0144	4.73E-22
HSPA4	5:132384131	0.0638	0.00491	5.32E-38
HSPA6	1:161482520	-0.0642	0.00923	3.99E-12
HSPB11	1:54373313	0.0557	0.00882	2.97E-10
HSPBAP1	3:122473431	-0.144	0.00476	2.52E-186
HSPH1	13:31700923	0.061	0.00565	6.47E-27
HTATIP2	11:20384886	-0.106	0.00428	1.02E-128
HTRA4	8:38856336	-0.0179	0.00294	1.27E-09
HUS1	7:48015688	0.094	0.00316	1.69E-179

HYAL3	3:50329826	-0.0166	0.00234	1.75E-12
HYDIN	16:71311855	-0.0956	0.00681	5.55E-44
HYOU1	11:118921306	-0.0267	0.00428	5.30E-10
HYPK	15:44120559	0.0279	0.00394	1.51E-12
IARS	9:94973571	0.0818	0.00889	5.06E-20
ICA1	7:8301583	0.0286	0.00302	3.70E-21
ICA1L	2:203625866	0.0558	0.00322	2.32E-65
ICAM2	17:62106978	0.0284	0.00407	3.14E-12
ICAM3	19:10458547	-0.0824	0.00686	8.03E-33
ICAM4	19:10422972	-0.0782	0.013	2.08E-09
ICMT	1:6274304	0.0649	0.00729	7.31E-19
ICOSLG	21:45639368	-0.0219	0.00352	5.88E-10
IDE	10:94379609	-0.0765	0.00868	1.53E-18
IDH1	2:209108718	0.0799	0.0083	9.01E-22
IDI1	10:1093992	0.0946	0.00706	2.69E-40
IDI2	10:1078782	0.0493	0.00687	8.77E-13
IDNK	9:86234596	-0.0408	0.00489	9.19E-17
IDO1	8:39773599	-0.123	0.00807	1.64E-51
IER3	6:30756882	0.0676	0.0042	9.54E-57
IFFO1	12:6652572	0.0217	0.00311	3.51E-12
IFI16	1:159045424	-0.108	0.0137	5.24E-15
IFI27	14:94583864	-0.21	0.0156	2.11E-40
IFI27L1	14:94564542	0.0516	0.0041	8.81E-36
IFI27L2	14:94595989	0.0418	0.00546	2.26E-14
IFI30	19:18282895	0.171	0.00595	9.07E-168
IFI35	17:41166417	0.0714	0.0116	7.23E-10
IFIT2	10:91066460	-0.0573	0.00956	2.27E-09
IFIT5	10:91186842	0.0942	0.0107	1.63E-18
IFITM1	11:316931	0.326	0.0407	1.41E-15
IFITM2	11:280000	-0.124	0.0106	5.36E-31
IFITM4P	6:29696852	-0.153	0.00531	1.29E-169
IFNAR1	21:34670242	-0.0556	0.00455	6.58E-34
IFNAR2	21:34607358	0.065	0.00436	3.31E-49
IFNGR2	21:34796556	-0.0853	0.00567	3.71E-50
IFNK	9:27570348	-0.0931	0.00861	5.64E-27
IFRD1	7:112109331	-0.0965	0.00737	1.42E-38
IFT27	22:37171611	-0.0479	0.00436	8.50E-28
IFT46	11:118437279	0.0642	0.00643	2.91E-23
IFT52	20:42225923	0.0403	0.00638	2.87E-10

IFT57	3:107934470	-0.0613	0.00854	8.31E-13
IFT74	9:26956768	-0.0861	0.00648	1.09E-39
IFT80	3:160060748	-0.0296	0.00355	9.36E-17
IFT88	13:21248862	-0.164	0.00477	3.76E-235
IGF1R	15:99505423	-0.0417	0.00536	9.39E-15
IGF2BP3	7:23351080	0.0738	0.0113	7.33E-11
IGFBP3	7:45975649	-0.0405	0.00466	4.40E-18
IGFBP7	4:57899602	-0.0236	0.00335	1.99E-12
IGFLR1	19:36230174	0.104	0.00377	9.82E-158
IGSF11	3:118909523	-0.0512	0.00658	8.36E-15
IGSF6	16:21620547	-0.0392	0.00538	3.75E-13
IK	5:140030306	-0.0298	0.0039	2.63E-14
IKBIP	12:99022400	-0.156	0.00787	7.39E-85
IKBKAP	9:111631743	0.0802	0.011	3.51E-13
IKBKE	1:206643774	0.0593	0.00427	3.46E-43
IKZF1	7:50462418	-0.0954	0.00401	4.58E-119
IKZF3	17:37985801	-0.0505	0.00582	5.58E-18
IKZF5	10:124763922	-0.0325	0.00383	3.20E-17
IL10RB	21:34606972	-0.052	0.00481	6.91E-27
IL11RA	9:34649442	-0.0813	0.00598	2.56E-41
IL12RB1	19:18194529	0.0432	0.00313	1.57E-42
IL12RB2	1:67798445	-0.107	0.00506	5.31E-96
IL15	4:142672097	-0.0297	0.00369	9.93E-16
IL15RA	10:6017838	0.0239	0.00231	9.02E-25
IL17RA	22:17586583	0.0292	0.00314	1.77E-20
IL17RB	3:53892553	-0.0223	0.00288	1.02E-14
IL18	11:112033129	-0.108	0.00659	7.04E-59
IL18BP	11:71685714	0.0429	0.00619	4.43E-12
IL18R1	2:102941338	0.112	0.00657	3.51E-63
IL18RAP	2:102985424	0.514	0.0112	<1.0E-314
IL1R2	2:102598754	-0.0929	0.00694	2.91E-40
IL1RAP	3:190284838	-0.157	0.0127	1.06E-34
IL1RL1	2:102941338	0.225	0.0104	1.29E-100
IL23A	12:56728137	0.0932	0.00876	3.42E-26
IL23R	1:67605134	0.0578	0.00524	5.98E-28
IL31RA	5:55131582	-0.239	0.013	5.23E-73
IL32	16:3116186	0.199	0.0107	3.47E-74
IL4R	16:27324880	-0.0486	0.00607	1.35E-15
IL5	5:131835395	-0.0376	0.00524	7.66E-13

IL5RA	3:3144765	0.0752	0.0115	5.78E-11
IL6R	1:154423764	-0.0251	0.00315	1.68E-15
IL6ST	5:55310583	0.165	0.0239	5.16E-12
IL7	8:79647495	0.0483	0.00577	7.73E-17
IL7R	5:35854265	-0.245	0.00721	2.31E-228
IL8	4:74572039	0.298	0.0179	1.32E-60
ILF3-AS1	19:10803124	-0.264	0.0211	1.88E-35
ILK	11:6633608	0.044	0.00491	3.75E-19
ILKAP	2:239075812	0.0369	0.0035	1.21E-25
IMMP2L	7:111057345	0.047	0.00453	6.48E-25
IMMT	2:86400092	0.0301	0.00333	2.11E-19
IMP4	2:131089135	-0.0589	0.00516	8.39E-30
IMPA1	8:82528953	0.0376	0.00471	1.65E-15
IMPA2	18:11962002	0.0972	0.00626	3.52E-53
IMPACT	18:21988293	-0.089	0.00869	2.00E-24
IMPG2	3:101046933	-0.0387	0.00306	5.12E-36
INADL	1:62207254	-0.109	0.0116	1.11E-20
ING1	13:111380701	0.0306	0.00292	2.05E-25
ING5	2:242630987	-0.259	0.0132	1.21E-82
INIP	9:115514725	-0.0993	0.0138	7.54E-13
INO80B	2:74710491	-0.0915	0.00401	7.42E-110
INO80C	18:33077912	0.19	0.0213	4.53E-19
INO80E	16:30050494	0.0212	0.00337	3.70E-10
INPP1	2:191225866	0.0355	0.00498	1.13E-12
INPP4B	4:143625262	0.12	0.00586	4.15E-90
INPP5A	10:134531955	0.0562	0.00724	1.02E-14
INPP5B	1:38331912	0.199	0.00527	9.83E-278
INPP5D	2:234077240	0.0259	0.00319	5.79E-16
INPP5K	17:1446091	0.14	0.00892	1.80E-54
INSIG1	7:155091753	-0.0858	0.00398	6.79E-99
INSIG2	2:118873636	0.0391	0.0054	5.46E-13
INSL3	19:17942927	0.0659	0.00801	2.37E-16
INTS10	8:19702001	-0.0846	0.00469	1.04E-70
INTS12	4:106675946	0.0678	0.00793	1.63E-17
INTS4	11:77653405	0.0472	0.0034	4.28E-43
INTS5	11:62438207	-0.0556	0.00595	1.29E-20
INVS	9:103061366	0.0289	0.00316	9.22E-20
IP6K2	3:48731961	-0.0518	0.00706	2.47E-13
IPCEF1	6:154542491	0.038	0.00641	3.24E-09

IPMK	10:60015313	0.139	0.00547	1.10E-133
IPO11	5:61891622	0.0268	0.00303	1.21E-18
IPO8	12:30783184	0.0377	0.00283	5.29E-40
IQCB1	3:121558911	0.0811	0.00443	1.13E-72
IQCE	7:2568770	0.0305	0.0038	1.23E-15
IQCG	3:197664916	-0.0809	0.0107	4.50E-14
IQGAP1	15:91037534	0.0617	0.00418	2.69E-48
IQGAP2	5:75698761	-0.158	0.00437	2.99E-256
IRAK3	12:66533086	-0.0855	0.00632	5.37E-41
IRAK4	12:44166318	0.0493	0.00608	6.87E-16
IREB2	15:78793921	0.0555	0.00283	7.82E-83
IRF2BP2	1:234751563	0.0291	0.00306	2.61E-21
IRF3	19:50115057	-0.0475	0.00781	1.27E-09
IRF6	1:209980027	-0.0353	0.00354	3.12E-23
IRS1	2:227647098	-0.0333	0.00507	5.52E-11
ISCA1	9:88876385	-0.344	0.00933	4.44E-265
ISCA2	14:74962233	-0.0802	0.00931	8.79E-18
ISCU	12:108949557	-0.0534	0.00577	2.88E-20
ISG20	15:89202717	-0.161	0.00611	1.35E-143
ISL2	15:76674624	0.018	0.0026	5.28E-12
ISOC1	5:128389088	-0.0664	0.0044	2.41E-50
ITCH	20:32993845	-0.019	0.0029	6.38E-11
ITGA1	5:52056209	-0.16	0.00989	1.05E-57
ITGA2	5:52316172	-0.0765	0.00313	6.85E-125
ITGA4	2:182323766	-0.102	0.00395	4.53E-137
ITGA9	3:37444901	0.0265	0.00232	9.02E-30
ITGAM	16:31248870	-0.0355	0.00412	9.96E-18
ITGAV	2:187492742	-0.0336	0.00335	1.68E-23
ITGAX	16:31331160	0.175	0.00428	<1.0E-314
ITGB1	10:33266876	-0.0322	0.00525	8.86E-10
ITGB2	21:46328099	0.15	0.00558	1.48E-149
ITGB3	17:45324590	0.0814	0.0123	4.56E-11
ITGB5	3:124600652	0.0233	0.0037	2.95E-10
ITGB7	12:53587468	-0.0944	0.00661	1.81E-45
ITIH2	10:7838932	0.0505	0.00242	3.01E-93
ITIH4	3:52870618	-0.0568	0.00248	6.10E-111
ITK	5:156670047	-0.0557	0.0075	1.27E-13
ITLN1	1:160827772	0.377	0.0154	1.26E-124
ITM2B	13:48824231	-0.0239	0.00352	1.36E-11

ITM2C	2:231750269	0.0696	0.0064	2.94E-27
ITPA	20:3192385	0.0766	0.00716	1.95E-26
ITPK1	14:93583933	0.12	0.0173	4.70E-12
ITPKB	1:226923938	-0.0165	0.00279	3.61E-09
ITPR1	3:4519303	0.0879	0.00838	1.62E-25
ITPR2	12:26986098	0.15	0.0062	3.01E-123
ITPR3	6:33601132	0.033	0.0026	2.45E-36
ITSN1	21:35030075	0.129	0.00358	3.68E-255
ITSN2	2:24459736	0.0182	0.0029	3.91E-10
IVD	15:40654038	0.0526	0.00339	3.70E-53
IVNS1ABP	1:185294981	0.0264	0.00379	3.65E-12
IWS1	2:128299079	-0.0451	0.00466	5.48E-22
JAGN1	3:9936427	0.107	0.00495	1.29E-99
JAK1	1:65344284	0.0426	0.00486	2.49E-18
JAK2	9:4984530	-0.0446	0.00562	2.77E-15
JAKMIP1	4:6179711	0.062	0.0038	1.72E-58
JAKMIP2	5:147162475	-0.169	0.0055	2.30E-190
JARID2	6:15231515	0.0856	0.00482	1.33E-68
JAZF1	7:28224053	0.0611	0.00817	8.49E-14
JKAMP	14:59973281	0.0548	0.00447	4.77E-34
JMJD6	17:74712310	0.0899	0.00361	1.86E-129
JMJD7-PLA2G4E	15:42115747	-0.0244	0.00229	2.56E-26
JMY	5:78548010	-0.12	0.00441	3.72E-153
JOSD1	22:39054474	0.034	0.00523	8.23E-11
JUP	17:39940775	-0.453	0.00741	<1.0E-314
KALRN	3:124352236	0.0453	0.00313	1.41E-46
KANK1	9:518755	-0.0493	0.00405	1.08E-33
KANK2	19:11303469	0.0551	0.00478	2.07E-30
KANSL1	17:44323046	-0.0865	0.00623	4.75E-43
KANSL1L	2:211060050	0.0279	0.00315	1.22E-18
KANSL2	12:49024333	-0.114	0.00395	9.57E-171
KAT2B	3:20105673	-0.172	0.00752	5.15E-110
KAT6A	8:41799893	-0.042	0.00391	1.19E-26
KATNA1	6:149992258	0.0524	0.00412	1.31E-36
KATNAL1	13:30895193	0.0814	0.00646	5.87E-36
KAZN	1:15278473	0.0178	0.00227	5.07E-15
KBTBD11	8:1956660	-0.0469	0.00585	1.27E-15
KBTBD2	7:32911455	-0.0745	0.00409	4.65E-72
KBTBD3	11:105947285	0.0812	0.00563	2.42E-46

KBTBD7	13:41760947	-0.0921	0.00625	3.18E-48
KBTBD8	3:67048762	-0.0561	0.00864	9.31E-11
KCNAB1	3:156248263	0.0425	0.00418	4.96E-24
KCNAB2	1:6094800	-0.0418	0.00365	4.42E-30
KCNE1	21:35872145	-0.0638	0.00423	3.45E-50
KCNE3	11:74171965	-0.124	0.00849	4.24E-47
KCNJ1	11:128679211	0.0729	0.00437	5.80E-61
KCNJ15	21:39613236	0.28	0.00728	8.05E-286
KCNJ2	17:68159261	0.074	0.0112	4.12E-11
KCNK17	6:39282036	-0.0464	0.00376	1.68E-34
KCNMA1	10:79312114	-0.0465	0.0021	2.87E-104
KCNMB1	5:169806001	-0.0338	0.00458	1.77E-13
KCNMB4	12:70719934	-0.0463	0.00579	1.60E-15
KCNN4	19:44282529	-0.0562	0.00295	3.85E-78
KCNQ5	6:73287285	-0.0629	0.00501	1.08E-35
KCTD10	12:109904436	-0.118	0.0083	2.95E-45
KCTD18	2:201397724	0.0404	0.00684	3.82E-09
KCTD2	17:73067482	0.0412	0.00454	1.69E-19
KCTD4	13:45784496	-0.124	0.0148	5.38E-17
KCTD7	7:66145659	-0.04	0.00518	1.31E-14
KCTD9	8:25272364	-0.0449	0.00565	2.45E-15
KDELC2	11:108387409	0.0986	0.00612	4.31E-57
KDELR2	7:6502367	0.0985	0.00573	2.32E-64
KDELR3	22:38895520	0.0566	0.00464	8.78E-34
KDM3A	2:86645750	-0.0626	0.00362	3.07E-65
KDM4C	9:7051826	0.0325	0.00442	2.14E-13
KDM5A	12:428693	0.0657	0.00325	2.23E-87
KDM5B	1:202738322	-0.0345	0.00389	9.16E-19
KDSR	18:61034420	-0.063	0.00484	4.15E-38
KHNYN	14:24899364	-0.0484	0.00321	1.49E-50
KIAA0020	9:2839608	-0.0646	0.00435	8.43E-49
KIAA0040	1:175139295	0.148	0.00461	1.56E-206
KIAA0226L	13:46932953	-0.154	0.00869	1.34E-68
KIAA0247	14:70114367	-0.0448	0.00686	7.44E-11
KIAA0319	6:24638740	0.0799	0.0039	1.26E-89
KIAA0355	19:34878790	-0.102	0.0113	2.33E-19
KIAA0368	9:114220603	0.0264	0.00371	1.35E-12
KIAA0391	14:35542759	-0.284	0.00558	<1.0E-314
KIAA0430	16:15687514	0.0567	0.00849	2.76E-11

KIAA0513	16:85059628	-0.111	0.00544	1.18E-89
KIAA0586	14:58894577	0.0255	0.00366	4.15E-12
KIAA0753	17:6529728	0.0808	0.00352	3.71E-111
KIAA0825	5:93918392	-0.113	0.00653	9.52E-65
KIAA0922	4:154516739	0.0319	0.0029	8.22E-28
KIAA0930	22:45630223	0.0431	0.00326	2.34E-39
KIAA1109	4:123172373	0.154	0.00834	3.29E-74
KIAA1191	5:175762026	-0.0866	0.0077	5.20E-29
KIAA1324	1:109704979	-0.685	0.00865	<1.0E-314
KIAA1324L	7:86663578	-0.15	0.0125	7.15E-33
KIAA1328	18:34557858	-0.0627	0.00474	2.16E-39
KIAA1383	1:232941153	0.0291	0.0033	1.71E-18
KIAA1429	8:95571602	0.0237	0.00294	7.84E-16
KIAA1430	4:186131648	0.092	0.00706	3.30E-38
KIAA1432	9:5750243	0.0239	0.00338	1.88E-12
KIAA1468	18:59898173	0.0393	0.00317	8.27E-35
KIAA1524	3:108251509	-0.157	0.00527	2.30E-179
KIAA1551	12:32123820	0.0853	0.00802	3.35E-26
KIAA1586	6:56896601	0.0568	0.00894	2.27E-10
KIAA1598	10:118643670	-0.345	0.0077	<1.0E-314
KIAA1683	19:18388158	-0.0745	0.00253	3.75E-177
KIAA1715	2:176871243	0.127	0.00525	1.33E-122
KIAA1737	14:77586119	0.0514	0.00405	2.97E-36
KIAA1804	1:233468562	-0.0419	0.00453	2.82E-20
KIAA1919	6:111582885	0.138	0.00532	1.30E-140
KIAA1967	8:22492143	-0.0202	0.00234	1.02E-17
KIF11	10:94432000	0.036	0.00478	5.90E-14
KIF13A	6:17965147	-0.0566	0.00535	6.24E-26
KIF14	1:200564868	-0.0329	0.00509	1.16E-10
KIF16B	20:16536155	0.266	0.0064	<1.0E-314
KIF1B	1:10271688	-0.209	0.00657	5.93E-204
KIF1C	17:4897042	-0.0223	0.00305	2.95E-13
KIF24	9:34302153	-0.0815	0.00867	8.17E-21
KIF27	9:86505242	-0.409	0.036	1.46E-29
KIF3A	5:132072913	0.075	0.00912	2.47E-16
KIF5B	10:32332682	-0.0612	0.00576	4.29E-26
KIF5C	2:149797754	-0.0578	0.0068	2.53E-17
KIFAP3	1:169990587	0.0585	0.00627	1.68E-20
KIN	10:7869814	-0.0341	0.00535	2.01E-10

KIR3DL1	19:55376524	0.112	0.0115	2.58E-22
KIR3DX1	19:55040648	-0.0503	0.00541	2.07E-20
KLC1	14:104095320	-0.0699	0.00329	3.89E-96
KLC3	19:45847110	-0.0438	0.00584	7.44E-14
KLF10	8:103662852	-0.048	0.00466	1.28E-24
KLF11	2:10219746	0.0778	0.00642	2.40E-33
KLF12	13:74339104	0.0288	0.00426	1.61E-11
KLF3	4:38692835	-0.0221	0.0037	2.44E-09
KLF6	10:3820787	0.0221	0.00376	4.41E-09
KLF7	2:208019791	0.0441	0.00385	6.18E-30
KLHDC1	14:50162763	-0.0648	0.00923	2.54E-12
KLHDC10	7:129764961	0.0878	0.00634	7.07E-43
KLHDC4	16:87764267	-0.0647	0.00311	2.03E-92
KLHDC8B	3:49256529	0.188	0.0221	2.57E-17
KLHL11	17:40068613	-0.113	0.00655	8.96E-65
KLHL20	1:173674087	0.0259	0.00423	9.67E-10
KLHL3	5:137070445	-0.0293	0.0036	4.72E-16
KLHL36	16:84696385	-0.0285	0.00378	5.63E-14
KLHL5	4:39023992	0.0648	0.00517	1.45E-35
KLHL7	7:23113503	-0.056	0.00734	2.77E-14
KLHL8	4:88187880	-0.0347	0.00569	1.20E-09
KLKB1	4:187119285	-0.228	0.00704	1.68E-209
KLRB1	12:9735686	0.118	0.00876	1.15E-40
KLRC1	12:10596720	-0.398	0.0152	3.05E-142
KLRC3	12:10584152	-0.663	0.0198	4.85E-224
KLRD1	12:10463014	0.315	0.0114	1.64E-155
KLRG1	12:9181706	0.298	0.0109	7.39E-154
KLRK1	12:10563592	0.0874	0.00779	6.87E-29
KMO	1:241689251	0.191	0.0224	1.84E-17
KNTC1	12:123025873	-0.038	0.00437	4.79E-18
KPNA4	3:160294185	-0.0405	0.00303	3.84E-40
KRAS	12:25338274	-0.0317	0.00372	1.89E-17
KREMEN1	22:29514956	-0.157	0.00676	1.79E-113
KRI1	19:10672493	0.0565	0.00356	1.70E-55
KRR1	12:75910334	-0.0543	0.00428	2.79E-36
KRT1	12:53065916	-0.137	0.00378	2.00E-255
KRT10	17:39027692	-0.0338	0.00542	4.84E-10
KRT23	17:39087292	0.249	0.00805	3.44E-193
KRT5	12:52916022	0.0261	0.00349	9.41E-14

KRT72	12:53027940	0.202	0.00699	3.06E-170
KRT73	12:53026975	0.139	0.00562	2.31E-127
KRT77	12:53086337	-0.0799	0.00912	2.47E-18
KRTCAP3	2:27711157	0.0556	0.00689	9.32E-16
KY	3:134317008	0.0525	0.0037	9.59E-45
KYNU	2:143649203	-0.407	0.0146	1.29E-160
L1TD1	1:62660090	-0.214	0.0127	6.49E-62
L3HYPDH	14:59944725	0.0651	0.00387	9.32E-62
L3MBTL3	6:130374461	-0.16	0.00408	1.48E-296
L3MBTL4	18:6411957	0.0652	0.00433	3.47E-50
LACC1	13:44442238	-0.0405	0.00511	2.74E-15
LACTB	15:63408320	-0.0915	0.00466	8.68E-83
LACTB2	8:71564667	-0.0685	0.0043	8.74E-56
LAIR1	19:54852514	0.0415	0.00391	4.61E-26
LAIR2	19:55014172	0.776	0.0695	1.16E-28
LAMC1	1:182988052	0.0923	0.00327	1.36E-163
LAMP5	20:9492062	-0.0364	0.00602	1.64E-09
LAMTOR2	1:156025096	0.0451	0.00567	2.12E-15
LAMTOR5	1:110966889	0.0598	0.00598	2.33E-23
LANCL1	2:211247567	0.035	0.00408	1.31E-17
LAP3	4:17577329	-0.143	0.00655	8.58E-102
LAPTM4A	2:20226596	0.0764	0.00532	6.12E-46
LAPTM4B	8:98779797	0.107	0.00986	6.29E-27
LAPTM5	1:31206637	-0.116	0.00465	9.14E-131
LARP1	5:154196592	0.0272	0.00425	1.79E-10
LARP1B	4:129017293	0.0316	0.00404	6.10E-15
LARP4	12:50806930	-0.0913	0.0118	1.47E-14
LARP4B	10:846977	0.042	0.00485	6.16E-18
LARP7	4:113510368	-0.0511	0.00845	1.56E-09
LARS	5:145487296	-0.0745	0.0036	1.50E-91
LARS2	3:45407535	0.024	0.00323	1.23E-13
LASP1	17:37054253	0.0583	0.00384	6.59E-51
LAT	16:28957172	0.0938	0.0127	1.57E-13
LATS2	13:21590089	-0.0281	0.00475	3.71E-09
LBH	2:30509158	-0.163	0.00542	8.00E-183
LBR	1:225608435	0.052	0.0071	2.95E-13
LBX2-AS1	2:74740961	-0.102	0.00598	2.30E-63
LCA5L	21:40761545	-0.13	0.00376	7.85E-237
LCLAT1	2:30657532	-0.23	0.00426	<1.0E-314

LCMT1	16:25159145	0.0346	0.0043	9.64E-16
LCMT2	15:43609509	0.0588	0.00588	2.24E-23
LCN8	9:139684867	-0.0315	0.00535	4.22E-09
LCP1	13:46714885	-0.0166	0.00279	3.35E-09
LCP2	5:169730365	-0.0412	0.00647	2.01E-10
LDB1	10:103874822	0.139	0.0123	2.52E-29
LDHA	11:18415057	0.0271	0.0031	2.86E-18
LDHC	11:18400811	-0.254	0.00597	<1.0E-314
LDLRAP1	1:25890835	0.0835	0.00432	1.75E-80
LDOC1L	22:44852717	0.03	0.00281	1.97E-26
LEKR1	3:156546675	0.0274	0.00382	9.25E-13
LEMD3	12:65575683	-0.021	0.00306	8.36E-12
LEPR	1:65890236	0.132	0.00567	9.76E-114
LEPROTL1	8:29967903	0.0612	0.00754	5.87E-16
LETMD1	12:51441897	0.0357	0.0038	8.63E-21
LGALS1	22:38062112	0.0616	0.00656	8.44E-21
LGALS14	19:40227363	-0.0435	0.0037	1.62E-31
LGALS17A	19:40176917	0.0476	0.00409	8.02E-31
LGALS2	22:37960365	1.05	0.0184	<1.0E-314
LGALS3	14:55610940	0.0435	0.00623	3.20E-12
LGALS4	19:39304402	-0.057	0.00829	6.94E-12
LGALS8	1:236699354	0.184	0.0082	3.30E-106
LGALSL	2:64690627	-0.132	0.0203	8.37E-11
LGR4	11:27480827	-0.0169	0.00287	3.96E-09
LGSN	6:64078347	-0.0876	0.00753	7.14E-31
LHFPL2	5:77784738	-0.0344	0.0043	1.35E-15
LHPP	10:126160495	0.0337	0.00403	7.89E-17
LIAS	4:39473195	0.0924	0.00831	2.16E-28
LIG1	19:48689548	-0.0191	0.00316	1.43E-09
LIG3	17:33307586	0.0212	0.00296	8.86E-13
LILRA1	19:55093320	0.274	0.0109	4.97E-132
LILRA2	19:55093173	0.402	0.0148	3.53E-151
LILRA5	19:54827040	0.28	0.0248	2.96E-29
LILRB1	19:55118872	0.124	0.0106	1.94E-31
LILRB2	19:54800679	-0.352	0.0135	3.89E-141
LILRB3	19:54768528	0.357	0.0144	1.71E-128
LILRB4	19:55172539	-0.0927	0.0152	1.18E-09
LIM2	19:51883149	-0.031	0.00478	9.31E-11
LIMA1	12:50599219	-0.0352	0.00389	1.87E-19

LIMD1	3:45693416	-0.0737	0.00379	2.75E-81
LIMD2	17:61817048	0.0309	0.00364	2.75E-17
LIMK1	7:73549169	0.0444	0.00298	3.93E-49
LIN54	4:83919524	0.0343	0.00369	2.28E-20
LIN7A	12:81333553	0.387	0.0189	3.21E-90
LIN7C	11:27477864	-0.0584	0.0049	2.19E-32
LIN9	1:226449936	-0.145	0.024	1.40E-09
LINC00085	19:52178063	0.0432	0.00347	4.37E-35
LINC00161	21:29923340	0.0474	0.00637	1.08E-13
LINC00173	12:116975822	0.144	0.0165	3.39E-18
LINC00189	21:30582732	-0.396	0.0203	1.51E-81
LINC00282	13:52462939	-0.0854	0.0111	1.37E-14
LINC00324	17:8160602	-0.0296	0.00423	3.00E-12
LINC00339	1:22353986	-0.216	0.00536	<1.0E-314
LINC00467	1:211621441	-0.131	0.0221	3.32E-09
LINC00471	2:232378792	0.103	0.00687	2.49E-49
LINC00476	9:98635849	-0.0784	0.0056	9.46E-44
LINC00612	12:9173237	0.165	0.00599	2.99E-155
LINGO2	9:28987757	-0.0969	0.00684	1.02E-44
LINS	15:101110565	-0.0806	0.00644	2.07E-35
LIPA	10:91002804	-0.296	0.00553	<1.0E-314
LIPC	15:58770523	-0.125	0.00658	2.77E-78
LIPH	3:185265185	0.0635	0.0105	1.37E-09
LIPT1	2:99804278	0.0788	0.00431	1.85E-72
LITAF	16:11722729	-0.0648	0.00982	4.69E-11
LMAN1	18:56987447	-0.0947	0.00517	1.39E-72
LMBR1	7:156562243	0.137	0.0056	1.88E-125
LMBR1L	12:49484494	0.0347	0.00392	1.02E-18
LMBRD1	6:70514496	-0.0547	0.00479	7.04E-30
LMLN	3:197762004	-0.0357	0.00524	1.08E-11
LMNB1	5:126160582	-0.0849	0.00558	3.58E-51
LMO7	13:76323507	0.0436	0.00617	1.90E-12
LMOD1	1:201910609	0.043	0.00444	4.83E-22
LMTK2	7:97757101	0.109	0.00627	3.62E-66
LNPEP	5:96355448	-0.0711	0.00286	6.45E-129
LNX2	13:28172775	0.132	0.00507	2.26E-141
LOC100128252	19:57048240	0.153	0.0117	2.30E-38
LOC100130691	2:178297740	0.0642	0.00611	1.46E-25
LOC100130950	17:5134814	0.161	0.00538	2.05E-182

LOC152217	3:196665864	-0.127	0.00687	6.69E-74
LOC284757	20:58894287	0.113	0.00617	1.09E-72
LOC285696	5:17213102	0.064	0.00839	2.70E-14
LOC286367	9:107525011	-0.0604	0.00906	2.88E-11
LOC338799	12:122251228	0.0716	0.00629	9.67E-30
LOC339803	2:61405795	0.0533	0.00562	3.66E-21
LOC554206	16:25037540	0.0446	0.00567	4.10E-15
LOC554223	6:29769921	-0.0924	0.00757	7.75E-34
LOC654433	2:113978650	0.224	0.00552	<1.0E-314
LOC728175	4:185275108	-0.0966	0.00874	4.28E-28
LOC728558	10:97504093	-0.249	0.0133	5.30E-76
LOC79015	20:43273635	0.136	0.0131	9.92E-25
LOC93622	4:6677430	-0.0983	0.0102	1.04E-21
LOH12CR1	12:12624336	-0.0729	0.00746	2.05E-22
LONP1	19:5666336	0.013	0.00209	4.93E-10
LONP2	16:48390512	-0.091	0.00426	3.30E-97
LONRF1	8:12623036	0.0642	0.00749	1.39E-17
LOXHD1	18:44157489	0.179	0.00387	<1.0E-314
LPAR1	9:113848169	0.128	0.00781	3.12E-59
LPAR2	19:19748572	-0.0865	0.0067	1.50E-37
LPAR3	1:85361653	-0.194	0.00767	9.16E-134
LPCAT1	5:1494439	-0.0291	0.00393	1.39E-13
LPCAT2	16:55562466	0.056	0.00514	2.42E-27
LPCAT3	12:7145853	0.0578	0.00749	1.41E-14
LPGAT1	1:212008208	0.033	0.00402	3.18E-16
LPIN1	2:11936798	0.0692	0.00388	2.93E-69
LPIN2	18:3059805	-0.116	0.00528	5.18E-103
LPL	8:19860197	-0.16	0.00772	1.10E-91
LPP	3:187870778	-0.0436	0.00396	5.95E-28
LPPR2	19:11431901	-0.0405	0.00503	1.14E-15
LPXN	11:58286886	0.176	0.0128	6.16E-42
LRBA	4:151706926	0.0438	0.00501	3.37E-18
LRCH1	13:47238854	-0.0331	0.00405	4.14E-16
LRGUK	7:133770621	0.0834	0.00386	3.70E-99
LRIG1	3:66436182	-0.0175	0.00283	7.49E-10
LRMP	12:25242333	0.0389	0.0042	2.74E-20
LRP10	14:23321112	-0.0446	0.0048	2.19E-20
LRP12	8:105544860	0.0727	0.00577	5.85E-36
LRP2BP	4:186236239	-0.0466	0.0045	7.59E-25

LRP6	12:12426161	-0.139	0.00803	4.46E-65
LRP8	1:53813684	0.017	0.00271	4.31E-10
LRPAP1	4:3527185	0.0427	0.00375	9.44E-30
LRPPRC	2:44141849	0.0227	0.00319	1.17E-12
LRR1	14:50084394	-0.0598	0.00434	1.51E-42
LRRC1	6:53637762	-0.0321	0.00349	5.84E-20
LRRC14	8:145756170	-0.0381	0.00512	1.14E-13
LRRC16A	6:25398426	-0.21	0.00539	1.23E-291
LRRC17	7:102618968	-0.325	0.0221	4.19E-48
LRRC18	10:50119054	0.0406	0.00586	4.82E-12
LRRC2	3:46518016	0.083	0.00862	8.69E-22
LRRC23	12:7007806	-0.0458	0.00388	1.21E-31
LRRC25	19:18510925	0.0671	0.00372	1.69E-70
LRRC27	10:134158940	-0.107	0.00739	1.42E-46
LRRC28	15:99788247	-0.0653	0.00454	5.77E-46
LRRC33	3:196415355	0.0393	0.00655	2.02E-09
LRRC34	3:169551558	0.1	0.0145	4.65E-12
LRRC37BP1	17:28952286	-0.0679	0.00886	2.18E-14
LRRC39	1:100603706	-0.117	0.0116	1.08E-23
LRRC4	7:127625250	-0.0861	0.00694	6.97E-35
LRRC47	1:3700119	0.0336	0.00314	2.22E-26
LRRC48	17:17894674	-0.0428	0.00577	1.36E-13
LRRC57	15:42836558	-0.115	0.00711	2.29E-57
LRRC6	8:133694084	-0.556	0.00952	<1.0E-314
LRRC61	7:150004244	-0.0304	0.00394	1.24E-14
LRRC7	1:70179004	-0.102	0.00364	2.41E-160
LRRC8B	1:89941533	-0.257	0.00587	<1.0E-314
LRRC8D	1:90282382	0.0516	0.00384	1.40E-40
LRRCC1	8:86098018	0.059	0.00567	4.59E-25
LRRFIP1	2:238586472	-0.0642	0.0042	1.11E-51
LRRFIP2	3:37055712	0.0652	0.0032	1.07E-88
LRRIQ3	1:74667686	0.129	0.00546	6.69E-118
LRRK1	15:101468792	0.0247	0.00361	9.10E-12
LRRN1	3:3865370	-0.15	0.0101	1.16E-48
LRRTM2	5:138155959	0.136	0.00664	2.41E-89
LRWD1	7:102065322	0.0451	0.00471	1.66E-21
LSG1	3:194377712	-0.216	0.00579	2.01E-270
LSM1	8:38043758	-0.0537	0.00642	8.16E-17
LSM6	4:147106801	0.0444	0.00582	2.60E-14

LSMD1	17:7742601	-0.0926	0.0145	1.92E-10
LSR	19:35714581	0.0195	0.00283	6.25E-12
LSS	21:47652549	0.0537	0.00342	3.07E-54
LST1	6:31552850	0.0704	0.00467	2.26E-50
LTA4H	12:96419941	0.285	0.0105	3.27E-151
LTBP3	11:65326154	-0.025	0.00241	6.47E-25
LTBR	12:6451590	-0.0229	0.00244	8.05E-21
LTF	3:46490250	-0.0368	0.00614	2.35E-09
LTN1	21:30310639	-0.0282	0.0031	1.17E-19
LUC7L	16:249987	0.0656	0.00384	1.06E-63
LUZP1	1:23432346	0.0227	0.00312	4.10E-13
LUZP6	7:135628370	0.0565	0.00749	5.52E-14
LXN	3:158393178	-0.0647	0.00511	3.22E-36
LY6G5B	6:31643251	0.0728	0.0063	1.40E-30
LY6G5C	6:31630241	0.0768	0.00355	9.55E-100
LY75	2:160689916	-0.166	0.00403	<1.0E-314
LY86	6:6579484	-0.129	0.00643	1.00E-85
LY96	8:74906729	-0.158	0.0102	6.22E-53
LYAR	4:4271886	-0.0798	0.00669	2.31E-32
LYG1	2:99866877	-0.0557	0.00456	6.52E-34
LYG2	2:99863928	-0.0757	0.00733	1.03E-24
LYN	8:56785685	0.0327	0.0046	1.37E-12
LYNX1	8:143879290	-0.0215	0.00245	2.74E-18
LYPD2	8:143838669	-0.0352	0.00506	3.96E-12
LYPLA1	8:54950307	0.338	0.0527	1.51E-10
LYPLAL1	1:219394080	-0.101	0.00573	6.18E-67
LYRM4	6:5260694	-0.247	0.016	9.81E-53
LYRM5	12:25361756	-0.155	0.0201	1.43E-14
LYRM7	5:130550343	-0.143	0.0111	2.65E-37
LYSMD3	5:89814604	0.268	0.00562	<1.0E-314
LYSMD4	15:100273607	-0.0352	0.00327	9.77E-27
LYVE1	11:10563090	0.245	0.00803	1.98E-188
LYZ	12:69692420	0.195	0.00458	<1.0E-314
LZTFL1	3:45883992	-0.129	0.00791	3.28E-58
M6PR	12:9110832	-0.0577	0.00414	2.66E-43
MAB21L3	1:116626228	-0.195	0.0117	5.52E-61
MACF1	1:39662654	0.0922	0.00751	3.41E-34
MACROD2	20:14024361	0.0316	0.00468	1.47E-11
MADD	11:47365276	-0.0522	0.0046	1.53E-29

MAEL	1:166911149	-0.0242	0.00269	4.18E-19
MAF1	8:145126375	0.131	0.0148	1.21E-18
MAGEF1	3:184427787	-0.0429	0.0043	3.11E-23
MAGOHB	12:10762757	-0.0594	0.00557	2.54E-26
MAK	6:10842660	0.108	0.00871	1.21E-34
MAL	2:95647049	-0.0381	0.00636	2.34E-09
MAML3	4:140793731	0.0498	0.00306	4.34E-58
MAN1A1	6:119575001	0.0813	0.00488	9.47E-61
MAN1A2	1:117959250	-0.0489	0.00338	1.61E-46
MAN1B1	9:139956356	-0.0592	0.00462	5.64E-37
MAN2A1	5:109171997	0.0354	0.00278	1.73E-36
MAN2A2	15:91498127	-0.026	0.00401	1.02E-10
MAN2B2	4:6610622	0.0231	0.00304	3.93E-14
MAN2C1	15:75681000	-0.0608	0.00266	3.64E-110
MANBA	4:103561709	-0.0413	0.00323	5.22E-37
MANBAL	20:35945343	0.0391	0.00424	4.39E-20
MANEA	6:96051383	-0.198	0.00492	<1.0E-314
MANF	3:51469107	0.0389	0.00496	5.75E-15
MAP1LC3B	16:87451617	0.292	0.0146	5.58E-86
MAP2K1	15:66702345	0.0529	0.00496	2.65E-26
MAP2K5	15:67909279	0.107	0.00328	5.38E-212
MAP3K11	11:65387378	-0.019	0.00253	8.30E-14
MAP3K5	6:137099008	0.0587	0.00308	2.43E-78
MAP3K7	6:91243924	0.0175	0.00259	1.91E-11
MAP3K8	10:30759454	-0.0288	0.00344	7.30E-17
MAP3K9	14:71276206	-0.036	0.00294	3.94E-34
MAP4	3:48079577	0.0257	0.00305	4.49E-17
MAP4K5	14:50845231	-0.0845	0.00572	2.27E-48
MAP6D1	3:183487393	-0.0243	0.00329	1.74E-13
MAP7	6:136882518	-0.105	0.0147	9.12E-13
MAP7D1	1:36627542	-0.0366	0.00229	4.32E-56
MAPK3	16:30081367	-0.231	0.011	9.46E-94
MAPK8	10:49564120	0.0718	0.00434	8.53E-60
MAPK9	5:179699504	-0.0935	0.00517	4.77E-71
MAPKAPK2	1:206884655	-0.0296	0.00444	2.71E-11
MAPKAPK3	3:50735933	0.0833	0.00554	4.73E-50
MAPRE1	20:31403206	0.0841	0.0061	1.66E-42
MAPRE2	18:32729627	-0.0634	0.00518	6.33E-34
MARCH1	1:220960137	-0.287	0.0143	8.23E-86

MARCH3	5:126171999	-0.0945	0.00641	3.27E-48
MARCH5	10:94129862	-0.0207	0.00351	3.83E-09
MARCO	2:119718028	0.117	0.00405	8.79E-170
MARK3	14:103850424	-0.0605	0.00514	1.32E-31
MARK4	19:45793115	-0.0519	0.00204	7.29E-135
MARS2	2:198569051	-0.084	0.00422	7.70E-85
MASP2	1:11092646	-0.0537	0.0041	1.15E-38
MASTL	10:27399117	-0.247	0.00455	<1.0E-314
MAT2A	2:85805373	-0.143	0.0116	1.32E-34
MAT2B	5:162936464	0.0405	0.00488	1.50E-16
MATR3	5:138656848	0.0405	0.00354	5.07E-30
MAX	14:65546361	-0.0471	0.00285	1.23E-59
MB21D1	6:74155346	0.0316	0.00495	2.02E-10
MB21D2	3:192627060	0.343	0.0434	3.39E-15
MBD2	18:51771171	0.0318	0.00486	6.56E-11
MBD5	2:148937370	0.035	0.00375	1.64E-20
MBLAC1	7:99732948	-0.0696	0.00694	1.82E-23
MBNL1	3:152184573	0.0243	0.00408	2.60E-09
MBOAT1	6:20124860	0.056	0.00517	5.29E-27
MBOAT2	2:9140487	0.0925	0.0101	9.65E-20
MBP	18:74728800	-0.191	0.0245	7.88E-15
MBTPS1	16:84095451	-0.0703	0.00287	2.75E-125
MCAT	22:43528240	-0.0247	0.00373	3.71E-11
MCC	5:112336015	0.0382	0.00254	3.51E-50
MCCC2	5:70923006	0.0354	0.00486	3.47E-13
MCEE	2:71353963	0.0401	0.00621	1.12E-10
MCF2L2	3:183146433	0.0306	0.00272	4.83E-29
MCFD2	2:47150575	0.0602	0.00588	2.44E-24
MCHR1	22:41116466	-0.113	0.00392	4.51E-170
MCL1	1:150595537	0.0238	0.00387	8.35E-10
MCM10	10:13209783	0.0208	0.00297	2.60E-12
MCM3	6:52192485	-0.0252	0.00364	5.35E-12
MCM3AP	21:47694712	-0.0536	0.00249	9.91E-99
MCM3AP-AS1	21:47708037	-0.021	0.00349	1.97E-09
MCM5	22:35806238	0.0691	0.00483	1.46E-45
MCM6	2:136617524	0.105	0.00411	1.94E-135
MCM7	7:99725146	-0.054	0.00836	1.15E-10
MCM8	20:5938207	0.1	0.00518	1.87E-80
MCM9	6:119254373	0.19	0.00923	2.28E-90

MCMBP	10:121652073	0.0462	0.00343	1.05E-40
MCOLN2	1:85403145	-0.105	0.00733	1.15E-45
MCOLN3	1:85465026	0.0333	0.00322	9.79E-25
MCPH1	8:6276063	-0.0456	0.00382	2.43E-32
MDC1	6:30686838	-0.305	0.0191	2.45E-56
MDFIC	7:114639833	0.0352	0.0049	8.27E-13
MDGA1	6:37663624	-0.091	0.0026	1.08E-241
MDM1	12:68720627	-0.0679	0.0036	5.06E-77
MDM2	12:69181478	0.188	0.00459	<1.0E-314
MDM4	1:204462050	-0.0499	0.00346	2.24E-46
MDN1	6:90336469	-0.0274	0.00337	5.44E-16
ME2	18:48362371	-0.0401	0.00355	3.48E-29
ME3	11:86383679	-0.0595	0.00423	4.24E-44
MEA1	6:42980218	-0.0992	0.0144	5.51E-12
MEAF6	1:37974497	0.0952	0.00575	6.07E-60
MED1	17:37575705	-0.0398	0.00641	5.61E-10
MED10	5:6375337	-0.0407	0.00336	2.96E-33
MED12L	3:151060764	-0.0173	0.00245	2.03E-12
MED15	22:20935946	0.04	0.0037	7.51E-27
MED19	11:57435255	-0.0753	0.00452	7.45E-61
MED21	12:27187211	-0.195	0.00947	8.67E-91
MED23	6:131898208	0.0755	0.00404	1.92E-75
MED24	17:38130492	0.0266	0.00353	5.12E-14
MED30	8:118551613	-0.0303	0.00472	1.54E-10
MED31	17:6553456	-0.115	0.00772	3.25E-49
MED4	13:48631483	-0.0512	0.00784	7.18E-11
MED6	14:71093669	0.0467	0.00728	1.46E-10
MEF2A	15:100186866	-0.0517	0.00576	3.80E-19
MEF2B	19:19286687	0.0266	0.00219	2.60E-33
MEFV	16:3292085	-0.0567	0.00343	4.80E-60
MEGF9	9:123501451	0.0338	0.0057	3.45E-09
MEI1	22:42108253	-0.0551	0.00509	4.77E-27
MEOX1	17:41734439	0.0438	0.00693	2.95E-10
MERTK	2:112781917	-0.27	0.0108	3.49E-130
MESDC2	15:81237906	0.037	0.00464	1.74E-15
METAP2	12:95896810	-0.059	0.00531	2.04E-28
METTL10	10:126500541	0.116	0.00784	1.42E-48
METTL13	1:171736641	0.0308	0.0041	7.02E-14
METTL14	4:119588308	0.0327	0.00399	3.12E-16

METTL17	14:21457945	0.0579	0.00407	3.75E-45
METTL18	1:169761370	0.3	0.00862	8.06E-239
METTL20	12:31803861	-0.0402	0.00576	3.27E-12
METTL21B	12:58171726	-0.0476	0.00341	1.18E-43
METTL21D	14:50588245	-0.074	0.00602	2.52E-34
METTL25	12:82888221	0.0647	0.0053	7.61E-34
METTL2B	7:128148180	0.0617	0.00576	1.65E-26
METTL3	14:21957934	-0.0592	0.00325	7.31E-72
METTL4	18:2558723	-0.0713	0.00357	1.50E-85
METTL6	3:15486118	0.0676	0.00812	1.09E-16
METTL7A	12:51270456	0.1	0.00526	3.97E-78
METTL8	2:172237959	0.0381	0.00565	1.80E-11
METTL9	16:21620547	-0.0721	0.00529	1.21E-41
MFF	2:228237579	0.111	0.00752	4.80E-48
MFGE8	15:89456948	-0.0424	0.00308	2.07E-42
MFHAS1	8:8639575	0.0693	0.0052	8.00E-40
MFN1	3:179094386	-0.385	0.015	4.98E-137
MFN2	1:12045555	-0.188	0.00376	<1.0E-314
MFSD1	3:158522463	-0.0456	0.00474	1.03E-21
MFSD5	12:53634662	-0.0737	0.00906	5.21E-16
MFSD6	2:191241196	-0.0633	0.00371	1.05E-63
MFSD9	2:103308802	-0.32	0.00582	<1.0E-314
MGAM	7:141796048	-0.106	0.00994	2.72E-26
MGAT1	5:180225919	-0.0529	0.00467	2.47E-29
MGAT2	14:50112712	-0.0223	0.00333	2.48E-11
MGAT4B	5:179216813	0.0967	0.0114	3.48E-17
MGC2752	19:59102379	0.0795	0.00452	1.96E-67
MGC39372	6:2877331	0.0962	0.0101	3.25E-21
MGC72080	7:97549283	0.905	0.0213	<1.0E-314
MGMT	10:131445027	0.0834	0.00385	4.70E-100
MGP	12:15071242	-0.0485	0.00437	2.75E-28
MGRN1	16:4692981	0.0384	0.00455	4.00E-17
MGST1	12:16504845	0.0966	0.0133	4.65E-13
MGST2	4:140592662	-0.176	0.00786	1.30E-105
MGST3	1:165600267	0.185	0.00473	5.79E-293
MICALCL	11:12383555	-0.0895	0.0132	1.30E-11
MICB	6:31478960	0.185	0.00797	4.24E-113
MIER1	1:67388945	0.148	0.0132	4.19E-29
MIER3	5:56276198	0.0399	0.00621	1.44E-10

MIF	22:24237221	0.0512	0.00512	2.44E-23
MIF4GD	17:73275747	0.0287	0.00381	6.89E-14
MINA	3:97650404	0.0335	0.00358	1.37E-20
MINK1	17:4825623	0.0538	0.00343	3.51E-54
MIOS	7:7607667	-0.0386	0.0055	2.75E-12
MIPEP	13:24436762	0.0271	0.00414	6.88E-11
MIS12	17:5413616	-0.166	0.00535	3.99E-194
MITD1	2:99814298	-0.0365	0.00484	6.14E-14
MKKS	20:10414950	0.0692	0.00382	2.60E-71
MKRN2	3:12614107	-0.0437	0.00299	1.58E-47
MLEC	12:121155966	0.0645	0.00493	1.38E-38
MLF1IP	4:185612953	0.0834	0.00415	1.07E-86
MLH1	3:37034946	-0.0614	0.00453	3.08E-41
MLH3	14:75504454	0.0915	0.00401	6.55E-110
MLKL	16:74735148	-0.11	0.00949	1.03E-30
MLLT1	19:6211876	-0.0278	0.0047	3.70E-09
MLLT10	10:21834536	0.0438	0.00311	3.31E-44
MLLT4	6:168190097	-0.122	0.0104	1.57E-31
MLLT6	17:36860072	0.0266	0.00444	2.11E-09
MLX	17:40716235	-0.0511	0.00319	1.61E-56
MLXIP	12:122607951	-0.0713	0.00661	7.57E-27
MMAA	4:146565740	-0.126	0.011	3.23E-30
MMAB	12:110003672	0.044	0.00366	7.77E-33
MMADHC	2:150423752	-0.144	0.00743	2.88E-81
MMD	17:53494994	-0.0627	0.0102	7.38E-10
MME	3:154781945	0.196	0.0248	2.57E-15
MMP24	20:33871661	0.0471	0.00428	7.00E-28
MMP25	16:3106424	-0.112	0.00874	6.69E-37
MMP8	11:102559962	-0.109	0.0161	1.66E-11
MMP9	20:44645339	-0.0633	0.00944	2.28E-11
MMS22L	6:97746897	0.0321	0.00395	5.14E-16
MNT	17:2285171	0.0735	0.00622	8.52E-32
MOCS1	6:39895689	-0.0797	0.0109	3.43E-13
MOCS2	5:52389455	-0.0403	0.00498	7.52E-16
MOG	6:29659124	-0.0219	0.00334	5.20E-11
MOGS	2:74716140	-0.0269	0.00404	2.88E-11
MON1B	16:77264217	0.0433	0.00313	9.33E-43
MON2	12:62950769	0.0254	0.00294	8.85E-18
MORC2	22:31340299	0.0317	0.00404	5.80E-15

MORC3	21:37660564	-0.142	0.00711	5.01E-86
MORN3	12:122112586	-0.0966	0.00466	7.00E-92
MORN4	10:99422648	0.0235	0.0039	1.94E-09
MOSPD3	7:100217810	0.0257	0.00346	1.35E-13
MOV10	1:113181739	-0.0405	0.00437	2.44E-20
MPC1	6:166802299	-0.0306	0.00442	4.92E-12
MPC2	1:167842652	0.0388	0.00649	2.37E-09
MPDU1	17:7526887	-0.067	0.00459	2.55E-47
MPHOSPH6	16:82166941	-0.281	0.00655	<1.0E-314
MPO	17:56367065	-0.0403	0.00676	2.51E-09
MPP5	14:67756236	0.0363	0.00558	8.61E-11
MPP7	10:28617810	0.16	0.00602	2.33E-145
MPPE1	18:11917083	0.196	0.00997	1.97E-83
MPPED2	11:30600383	-0.0505	0.00475	3.95E-26
MPRIP	17:17091746	-0.161	0.0109	3.31E-48
MPZ	1:161306663	0.127	0.0141	2.26E-19
MPZL1	1:167737311	0.19	0.0123	8.13E-53
MPZL2	11:118128455	0.184	0.00577	2.33E-204
MPZL3	11:118123517	0.0799	0.0062	1.79E-37
MR1	1:181025110	-0.0461	0.00563	3.56E-16
MRAS	3:138070901	0.0346	0.00524	4.58E-11
MRE11A	11:94210967	-0.11	0.00337	1.09E-211
MREG	2:216868598	0.036	0.00544	4.11E-11
MRGBP	20:61433141	-0.0843	0.00845	3.38E-23
MRI1	19:13881701	-0.0466	0.0051	1.01E-19
MROH8	20:35803303	0.0152	0.00215	1.63E-12
MRPL10	17:45889404	0.113	0.00681	3.83E-60
MRPL14	6:44081718	-0.0348	0.00473	2.21E-13
MRPL18	6:160188891	-0.197	0.00437	<1.0E-314
MRPL19	2:75864616	0.0283	0.00409	4.73E-12
MRPL21	11:68615600	0.228	0.00552	<1.0E-314
MRPL22	5:154283385	0.0584	0.00951	8.66E-10
MRPL27	17:48442121	0.0342	0.00298	4.18E-30
MRPL28	16:431609	0.125	0.0142	2.47E-18
MRPL30	2:99759865	-0.0841	0.00447	2.47E-76
MRPL32	7:42973722	-0.0459	0.00768	2.41E-09
MRPL35	2:86452673	-0.136	0.00948	9.69E-46
MRPL37	1:54683856	-0.0272	0.00374	3.71E-13
MRPL39	21:26997490	-0.0471	0.00515	8.26E-20

MRPL40	22:19421293	0.0625	0.00767	4.79E-16
MRPL42	12:93916772	-0.166	0.00771	5.16E-99
MRPL43	10:102746503	-0.106	0.00352	7.65E-185
MRPL44	2:224788346	0.0503	0.00504	3.01E-23
MRPL45	17:36464561	0.174	0.0251	4.83E-12
MRPL48	11:73480930	-0.0438	0.00552	2.35E-15
MRPL51	12:6613076	-0.11	0.00452	9.88E-125
MRPL53	2:74681502	-0.0536	0.00583	5.87E-20
MRPL54	19:3761652	0.0715	0.00454	1.24E-54
MRPS10	6:42173975	-0.0467	0.00488	1.78E-21
MRPS18C	4:84351153	-0.333	0.00751	<1.0E-314
MRPS21	1:150255587	-0.0941	0.00694	3.54E-41
MRPS22	3:139051919	0.0204	0.00297	7.13E-12
MRPS24	7:43906513	0.0432	0.00737	5.01E-09
MRPS25	3:15111397	0.0226	0.00343	4.73E-11
MRPS6	21:35401161	0.029	0.00325	6.08E-19
MRPS7	17:73240009	0.0853	0.00505	1.70E-62
MRPS9	2:105688786	0.0445	0.00407	1.60E-27
MRS2	6:24414976	-0.0539	0.00492	1.06E-27
MRTO4	1:19587878	-0.0607	0.00632	1.19E-21
MRVI1	11:10581127	0.0912	0.013	2.76E-12
MRVI1-AS1	11:10563090	0.0699	0.00624	8.82E-29
MS4A2	11:59877697	-0.094	0.0108	5.74E-18
MS4A4A	11:60039917	0.109	0.0156	2.62E-12
MS4A6A	11:59936979	0.087	0.00576	1.91E-50
MSANTD4	11:105905847	0.0634	0.00952	3.05E-11
MSC	8:72754639	-0.0367	0.00485	4.72E-14
MSH2	2:47590390	-0.279	0.00678	<1.0E-314
MSH3	5:79902336	-0.185	0.00436	<1.0E-314
MSH4	1:76385839	-0.319	0.0147	1.31E-100
MSH5	6:31711124	0.033	0.00353	1.38E-20
MSH6	2:48070873	0.0586	0.00429	9.14E-42
MSMO1	4:166264119	-0.137	0.00605	4.43E-109
MSR1	8:16038003	0.2	0.0152	7.74E-39
MSRA	8:9945667	-0.131	0.0106	1.18E-34
MSRB2	10:23361114	0.32	0.0137	5.63E-115
MT1A	16:56644650	0.0878	0.00668	7.86E-39
MT1H	16:56684241	-0.044	0.00741	3.02E-09
MT1L	16:56655171	-0.739	0.0307	2.08E-121

MTCH1	6:36911511	0.0332	0.00563	3.89E-09
MTCH2	11:47646640	0.0389	0.00409	2.96E-21
MTERFD1	8:97239126	0.0468	0.00504	2.10E-20
MTERFD2	2:242054129	0.125	0.00498	1.21E-130
MTERFD3	12:107370590	0.121	0.0127	3.05E-21
MTF2	1:93508182	-0.0364	0.00383	3.14E-21
MTFMT	15:65258834	0.0349	0.0045	1.17E-14
MTFR1	8:66524904	-0.2	0.00719	2.32E-158
MTHFD2	2:74436225	-0.0395	0.00652	1.42E-09
MTHFR	1:11839201	-0.0875	0.00614	3.08E-45
MTHFS	15:80130958	0.0754	0.00659	5.82E-30
MTIF2	2:55498584	0.416	0.0223	8.56E-75
MTMR10	15:31245880	-0.0264	0.00355	1.17E-13
MTMR12	5:32233963	-0.13	0.0131	5.78E-23
MTMR3	22:30236395	-0.0203	0.00323	3.56E-10
MTMR6	13:25859816	-0.0873	0.00442	9.79E-84
MTO1	6:74195981	0.0577	0.00366	6.66E-55
MTOR	1:11289161	-0.031	0.00314	8.94E-23
MTPAP	10:30597434	-0.186	0.00462	<1.0E-314
MTRF1	13:41845719	0.0594	0.00557	2.96E-26
MTRR	5:7892173	0.0561	0.00472	3.48E-32
MTSS1	8:125576094	-0.0473	0.00595	2.14E-15
MTUS1	8:17660151	-0.29	0.00878	9.94E-218
MUC20	3:195476085	-0.153	0.00794	9.75E-80
MUL1	1:20834610	-0.0763	0.00658	1.03E-30
MUT	6:49443984	-0.126	0.00366	1.06E-234
MVB12B	9:129206832	-0.0629	0.00401	2.39E-54
MVP	16:29845685	-0.0623	0.00381	7.87E-59
MX1	21:42793800	0.233	0.0174	3.06E-40
MX2	21:42743821	0.182	0.0166	2.02E-27
MXI1	10:112035723	-0.0612	0.00828	1.66E-13
MYBL2	20:42313552	-0.0376	0.00509	1.81E-13
MYBPC3	11:47397353	0.0257	0.00202	1.55E-36
MYBPH	1:203156080	0.0329	0.00304	6.18E-27
MYCT1	6:153043035	0.108	0.0114	7.37E-21
MYEF2	15:48462320	-0.767	0.0448	6.51E-64
MYH10	17:8426602	0.017	0.0023	1.52E-13
MYH11	16:15748380	-0.113	0.00252	<1.0E-314
MYH3	17:10610213	-0.0177	0.00255	4.79E-12

MYL6B	12:56564811	-0.0848	0.00582	3.12E-47
MYLIP	6:16111361	0.0396	0.00577	6.93E-12
MYO10	5:16927336	0.0285	0.00236	2.96E-33
MYO15B	17:73585569	-0.213	0.0074	2.30E-169
MYO18A	17:27517415	-0.132	0.0121	1.71E-27
MYO1D	17:31119037	-0.0842	0.0041	4.37E-90
MYO1E	15:59699057	0.0488	0.00412	6.14E-32
MYO3B	2:171076374	0.0142	0.00228	4.15E-10
MYO5A	15:52746722	-0.0828	0.00633	1.73E-38
MYO5C	15:52591752	-0.0933	0.00619	2.41E-50
MYO6	6:76488642	-0.038	0.00466	4.97E-16
MYO7B	2:128353649	0.0747	0.00747	2.24E-23
MYOF	10:95203701	0.108	0.00986	1.35E-27
MYOM1	18:3248888	0.0692	0.00308	1.42E-106
MYOM2	8:2082015	-0.163	0.00369	<1.0E-314
MYOZ2	4:120155096	-0.0285	0.00427	2.66E-11
MYRIP	3:40335616	-0.0297	0.00263	2.29E-29
MYSM1	1:59201213	-0.0341	0.00347	1.41E-22
MZT2A	2:132243501	0.308	0.0141	4.93E-101
N4BP2	4:40060368	-0.055	0.00458	8.29E-33
N4BP2L2	13:33126139	0.0244	0.00305	1.59E-15
N6AMT2	13:21378024	-0.18	0.00975	8.71E-74
NAA15	4:140236505	0.0259	0.00331	6.23E-15
NAA16	13:41876544	0.0432	0.00456	4.14E-21
NAA25	12:112479140	-0.0506	0.00685	1.79E-13
NAA38	7:117824239	-0.143	0.0055	5.03E-141
NAA60	16:3523626	0.0914	0.00767	2.46E-32
NAAA	4:76805267	-0.195	0.00475	<1.0E-314
NAALAD2	11:89861586	-0.0177	0.00288	7.87E-10
NAB1	2:191539496	0.0338	0.0049	6.23E-12
NADKD1	5:36242354	0.0577	0.00455	2.82E-36
NADSYN1	11:71132868	-0.0232	0.00338	7.84E-12
NAE1	16:66865559	-0.0291	0.00446	7.29E-11
NAF1	4:164083253	-0.0435	0.00585	1.13E-13
NAGK	2:71272082	0.234	0.00542	<1.0E-314
NANS	9:100824752	-0.0525	0.00429	5.46E-34
NAP1L1	12:76428875	-0.0581	0.00751	1.18E-14
NAP1L5	4:89621072	0.101	0.00674	2.08E-49
NAPB	20:23401586	-0.106	0.00684	1.67E-52

NAPG	18:10547559	-0.107	0.00373	1.46E-167
NAPRT1	8:144681777	0.0545	0.00247	1.32E-103
NAPSA	19:50863023	0.0367	0.00458	1.25E-15
NARS	18:55248890	-0.0313	0.00326	1.09E-21
NARS2	11:78282632	0.15	0.00519	7.01E-171
NAT8B	2:73927931	-0.115	0.00664	1.41E-65
NAV3	12:78427904	0.0279	0.0032	3.73E-18
NBAS	2:15720486	0.0194	0.00287	1.36E-11
NBEA	13:35662801	-0.178	0.0148	8.94E-33
NBN	8:90958530	0.0942	0.00583	3.23E-57
NBPF11	1:146488976	0.121	0.00919	5.13E-39
NBPF3	1:21795388	0.0579	0.00359	4.39E-57
NBR1	17:41370072	0.0369	0.00467	3.56E-15
NBR2	17:41298499	0.111	0.0042	1.97E-143
NCALD	8:103118484	-0.0824	0.00969	2.44E-17
NCAM1	11:112826186	0.116	0.0174	2.56E-11
NCAM2	21:22428841	-0.0409	0.00584	2.83E-12
NCAPD2	12:6627889	-0.105	0.00365	1.40E-167
NCAPD3	11:134023859	-0.0567	0.00491	1.43E-30
NCAPG2	7:158478691	-0.263	0.0161	2.53E-58
NCAPH2	22:50964153	-0.138	0.012	2.68E-30
NCBP2	3:196620788	-0.0315	0.00506	5.16E-10
NCEH1	3:172347134	0.0276	0.00428	1.27E-10
NCF2	1:183564287	-0.0379	0.00406	1.57E-20
NCK1	3:136571480	0.0305	0.00441	5.04E-12
NCK2	2:106509202	-0.0644	0.00356	8.99E-71
NCKAP1	2:183916217	-0.0788	0.00511	1.67E-52
NCKAP1L	12:54890515	-0.0384	0.00349	9.17E-28
NCLN	19:3186493	-0.0221	0.00336	5.34E-11
NCOA1	2:24986564	0.0268	0.00384	3.10E-12
NCOA2	8:71146050	-0.0378	0.00433	3.63E-18
NCOA3	20:46269884	-0.0649	0.006	5.32E-27
NCOA7	6:126162143	-0.0546	0.0036	6.93E-51
NCOR1	17:16131927	0.087	0.00516	5.04E-62
NCR3	6:31543827	0.0751	0.00553	2.39E-41
NCSTN	1:160317021	0.0674	0.0027	1.06E-129
NDC80	18:2585077	0.107	0.00527	3.97E-88
NDE1	16:15810211	-0.0596	0.00476	1.88E-35
NDFIP1	5:141466059	0.0262	0.00331	3.09E-15

NDN	15:23944930	-0.0523	0.00573	9.34E-20
NDRG1	8:134296742	0.0381	0.00318	1.19E-32
NDRG3	20:35237202	0.0607	0.0101	1.67E-09
NDST1	5:149868870	0.215	0.00534	<1.0E-314
NDST2	10:75572103	0.0288	0.0037	8.32E-15
NDUFA10	2:240894871	-0.042	0.00306	3.75E-42
NDUFA12	12:95397552	-0.098	0.00506	8.69E-81
NDUFA2	5:140030306	0.0461	0.0046	1.91E-23
NDUFA3	19:54631097	-0.05	0.0075	2.76E-11
NDUFA6	22:42486080	-0.24	0.0139	7.09E-65
NDUFAF1	15:41685917	0.0851	0.00515	7.92E-60
NDUFAF4	6:97354319	-0.0835	0.0111	5.59E-14
NDUFAF5	20:13786587	-0.126	0.00525	2.17E-121
NDUFAF6	8:96020877	-0.038	0.00599	2.41E-10
NDUFAF7	2:37465055	0.088	0.0091	6.44E-22
NDUFB1	14:92587277	-0.0423	0.00394	1.44E-26
NDUFB10	16:2007740	-0.0573	0.00736	8.08E-15
NDUFB2	7:140348291	0.0598	0.007	1.67E-17
NDUFB9	8:125562029	-0.0501	0.00726	5.77E-12
NDUFC2	11:77797626	0.168	0.0059	1.87E-166
NDUFS1	2:207039604	0.0208	0.00278	7.41E-14
NDUFS2	1:161186313	0.0285	0.0032	5.97E-19
NDUFS4	5:52954819	0.0437	0.00727	1.97E-09
NDUFS5	1:39520552	0.113	0.00506	5.49E-106
NDUFS8	11:67802907	-0.0404	0.00483	8.29E-17
NDUFV3	21:44323720	0.0959	0.00614	9.25E-54
NEB	2:152619186	-0.139	0.00723	3.71E-80
NEBL	10:21486604	-1.3	0.0267	<1.0E-314
NECAB2	16:84081330	-0.0409	0.00347	9.95E-32
NECAP1	12:8214856	-0.081	0.00469	6.43E-65
NECAP2	1:16795685	-0.0788	0.00356	3.89E-104
NEDD1	12:97338862	-0.0774	0.00353	2.49E-102
NEDD4	15:56216037	-0.149	0.00615	2.44E-123
NEDD4L	18:55723899	-0.106	0.00866	6.31E-34
NEDD8	14:24686145	0.297	0.0134	1.95E-104
NEDD9	6:11188939	-0.034	0.00384	1.15E-18
NEFL	8:24820164	0.0508	0.00488	3.77E-25
NEGR1	1:72752230	0.0558	0.00305	1.60E-72
NEIL2	8:11642545	-0.0189	0.00319	3.75E-09

NEIL3	4:178260872	0.094	0.0101	2.81E-20
NEK1	4:170533736	-0.097	0.00629	1.82E-52
NEK11	3:130703761	-0.0644	0.00395	2.54E-58
NEK3	13:52704624	-0.0442	0.00426	6.14E-25
NEK6	9:126985858	0.112	0.00393	1.87E-165
NELL2	12:45220936	0.23	0.0118	1.52E-81
NENF	1:212611054	0.037	0.00432	1.39E-17
NEO1	15:73343575	0.0955	0.00414	3.82E-112
NF1	17:29724708	0.0184	0.00296	5.07E-10
NFAM1	22:42796914	-0.0361	0.00416	4.90E-18
NFAT5	16:69686912	0.0191	0.00324	4.22E-09
NFATC2	20:50092287	0.0493	0.0056	1.75E-18
NFE2	12:54685880	0.207	0.0171	3.09E-33
NFIB	9:14201012	0.0369	0.00574	1.39E-10
NFIX	19:13200977	-0.55	0.0562	1.97E-22
NFKB1	4:103475444	-0.0222	0.0035	2.70E-10
NFKBIA	14:35847480	-0.112	0.00834	1.54E-40
NFRKB	11:129695058	0.0556	0.00362	3.53E-52
NFU1	2:69598243	-0.0307	0.00453	1.33E-11
NFXL1	4:47812183	-0.655	0.0127	<1.0E-314
NICN1	3:49480908	0.0254	0.00417	1.24E-09
NID1	1:236260164	0.0334	0.00542	7.29E-10
NID2	14:52491754	0.0135	0.00225	2.30E-09
NIF3L1	2:201768238	0.029	0.00424	8.45E-12
NINJ2	12:697095	0.102	0.00493	2.52E-91
NINL	20:25524743	-0.0276	0.00305	1.81E-19
NIP7	16:69374461	0.0532	0.00604	1.71E-18
NIPA1	15:23051312	-0.0402	0.00318	4.68E-36
NIPA2	15:23033076	0.0485	0.00314	1.86E-52
NIPAL2	8:99305363	0.391	0.00928	<1.0E-314
NIPAL3	1:24739220	0.0346	0.00391	1.09E-18
NIPSNAP1	22:29978331	-0.0506	0.00357	7.04E-45
NIPSNAP3A	9:107527916	-0.0925	0.00537	8.77E-65
NIPSNAP3B	9:107525011	-0.163	0.00962	1.41E-62
NIT1	1:161094834	-0.0625	0.0035	3.00E-69
NKG7	19:51885010	-0.0947	0.0124	3.13E-14
NKTR	3:42656578	0.0497	0.00431	1.75E-30
NKX3-1	8:23533898	-0.239	0.0156	4.26E-52
NLE1	17:33493071	-0.0366	0.00476	1.78E-14

NLN	5:65038564	-0.121	0.00447	1.67E-152
NLRC4	2:32489851	0.0341	0.00561	1.34E-09
NLRC5	16:57061189	-0.043	0.00317	3.46E-41
NLRP1	17:5480145	0.108	0.0105	1.83E-24
NLRP3	1:247612202	0.0551	0.00562	1.57E-22
NLRP6	11:268020	0.0391	0.00469	8.80E-17
NMD3	3:160936364	-0.0951	0.00432	5.31E-103
NME1	17:49231180	-0.149	0.0051	5.56E-174
NME4	16:449554	0.137	0.0146	8.38E-21
NME7	1:169214822	0.111	0.0141	3.59E-15
NME8	7:37875986	0.117	0.006	1.84E-81
NMI	2:152130288	-0.0398	0.00633	3.77E-10
NMNAT3	3:139396758	0.0434	0.00391	2.53E-28
NMRAL1	16:4525265	-0.071	0.00361	3.76E-83
NMRK1	9:77684690	-0.241	0.0057	<1.0E-314
NMT1	17:43169281	-0.0247	0.00386	1.62E-10
NMT2	10:15238886	-0.119	0.00597	4.94E-85
NMUR1	2:232386999	0.0233	0.00361	1.22E-10
NNT	5:43726241	0.109	0.00748	3.77E-47
NOA1	4:57843295	0.064	0.00789	6.34E-16
NOC3L	10:96104086	-0.0941	0.00598	1.61E-54
NOD2	16:50714029	0.256	0.00419	<1.0E-314
NOL10	2:10752617	-0.0469	0.00764	9.17E-10
NOL12	22:38085523	0.0275	0.00325	3.43E-17
NOL6	9:33520629	-0.0201	0.00305	4.54E-11
NOL9	1:6585804	0.0633	0.00625	6.96E-24
NOP10	15:34631927	0.0774	0.00822	6.61E-21
NOP14	4:3015001	0.0342	0.00335	3.23E-24
NOP16	5:175824324	-0.0555	0.00863	1.42E-10
NOS1AP	1:162019673	-0.0766	0.0087	1.67E-18
NOTCH1	9:139456353	0.0476	0.0054	1.63E-18
NOV	8:120460700	0.135	0.0146	3.95E-20
NOXRED1	14:77872979	0.0373	0.00616	1.44E-09
NPAS2	2:101423151	-0.0274	0.003	8.55E-20
NPAT	11:108021205	0.0444	0.00387	3.53E-30
NPC1	18:21107628	-0.037	0.00439	5.01E-17
NPC2	14:74966763	0.0462	0.00494	1.10E-20
NPDC1	9:139949830	-0.0568	0.00759	8.33E-14
NPEPL1	20:57292123	-0.0468	0.00436	1.24E-26

NPEPPS	17:45635239	-0.0693	0.0063	8.34E-28
NPHP3	3:132441268	-0.104	0.00304	7.29E-231
NPHP4	1:6053630	0.0147	0.00176	7.66E-17
NPIPL3	16:21368513	0.334	0.0556	2.02E-09
NPPA	1:11873512	-0.127	0.0141	2.72E-19
NPRL3	16:178543	-0.227	0.0122	1.91E-74
NQO2	6:3003970	0.29	0.00851	1.14E-229
NR1D1	17:38248354	-0.0619	0.00591	1.99E-25
NR1D2	3:23956575	-0.0681	0.00699	3.18E-22
NR1H3	11:47315917	0.0261	0.0031	4.56E-17
NR2F6	19:17340459	-0.0416	0.00583	1.14E-12
NR6A1	9:127410515	-0.0294	0.00387	3.33E-14
NRBF2	10:64873883	0.0364	0.00541	1.90E-11
NRBP1	2:27656823	0.0231	0.0027	1.57E-17
NRD1	1:52346354	0.052	0.00334	2.65E-53
NRDE2	14:90740588	-0.0181	0.003	1.68E-09
NREP	5:111091087	-0.0631	0.00815	1.21E-14
NRF1	7:129313989	-0.0225	0.00273	2.24E-16
NRG1	8:32432796	0.151	0.00394	1.25E-281
NRIP3	11:9024460	-0.0319	0.0044	4.77E-13
NRN1	6:5989938	0.088	0.00541	4.49E-58
NS3BP	11:750238	0.0685	0.00582	1.33E-31
NSFL1C	20:1451628	0.12	0.00429	4.44E-160
NSG1	4:4350655	0.189	0.0064	2.06E-176
NSL1	1:212955231	0.0875	0.00383	6.94E-110
NSMAF	8:59501767	-0.0206	0.00324	2.28E-10
NSMCE4A	10:123731471	0.0406	0.00421	9.44E-22
NSUN2	5:6605532	-0.102	0.00305	3.73E-224
NSUN4	1:46806429	-0.105	0.00336	6.07E-198
NSUN6	10:18852539	0.045	0.00434	6.12E-25
NSUN7	4:40731514	-0.0988	0.00498	2.24E-84
NT5C	17:73172526	-0.0277	0.00328	4.12E-17
NT5C2	10:104860053	0.0631	0.00421	1.21E-49
NT5C3	7:33035342	0.14	0.00935	2.22E-49
NT5C3L	17:39975164	-0.294	0.00495	<1.0E-314
NT5DC2	3:52573096	0.0165	0.00204	7.08E-16
NT5DC3	12:104180872	-0.229	0.00467	<1.0E-314
NT5E	6:86132619	0.17	0.007	3.25E-123
NT5M	17:17183513	-0.0613	0.004	6.60E-52

NTAN1	16:15151361	0.0789	0.00623	2.92E-36
NTNG1	1:107678268	-0.0594	0.00919	1.14E-10
NTPCR	1:233086882	-0.247	0.00659	6.30E-274
NUAK2	1:205304806	-0.0403	0.00379	3.61E-26
NUBP2	16:1846089	-0.0424	0.00433	2.23E-22
NUBPL	14:32297243	-0.0436	0.00516	3.90E-17
NUCB2	11:17299969	-0.0495	0.00594	9.28E-17
NUCKS1	1:205689807	0.027	0.00395	9.19E-12
NUDCD1	8:110283353	-0.0508	0.00737	5.79E-12
NUDCD3	7:44538713	-0.0287	0.00366	5.96E-15
NUDT1	7:2260715	-0.0687	0.00437	1.94E-54
NUDT12	5:102871079	0.258	0.00752	2.44E-233
NUDT14	14:105648963	-0.0834	0.011	3.57E-14
NUDT16P1	3:131081669	0.161	0.0115	6.59E-44
NUDT2	9:34320315	0.104	0.00542	7.59E-79
NUDT3	6:34335285	0.0391	0.00588	3.11E-11
NUDT5	10:12261852	-0.0937	0.00794	9.75E-32
NUDT9	4:88376782	0.0273	0.00362	5.53E-14
NUFIP2	17:27600063	0.037	0.0045	2.14E-16
NUMA1	11:71739423	-0.0156	0.00245	2.49E-10
NUMB	14:73788804	0.0289	0.00403	8.04E-13
NUP107	12:69079909	0.0886	0.00372	4.96E-119
NUP133	1:229627666	0.0524	0.00306	3.21E-64
NUP153	6:17716485	-0.0374	0.00472	2.66E-15
NUP210L	1:154127549	-0.697	0.0235	2.34E-179
NUP35	2:184048695	0.0381	0.00617	7.10E-10
NUP43	6:150003226	0.0525	0.00379	5.07E-43
NUP50	22:45570114	0.181	0.00779	5.08E-114
NUP54	4:77046810	-0.0503	0.00794	2.46E-10
NUP62	19:50405218	0.0171	0.00244	2.36E-12
NUP88	17:5282761	-0.127	0.00431	4.67E-177
NUP98	11:3812346	-0.0257	0.00343	7.47E-14
NUPL2	7:23229774	0.0238	0.00331	7.22E-13
NUSAP1	15:41630770	-0.131	0.00654	8.85E-86
NVL	1:224515776	0.0955	0.00637	9.48E-50
NXF1	11:62592195	0.0245	0.00377	8.54E-11
NXPE3	3:101498077	0.201	0.00906	1.04E-104
OAS1	12:113348870	0.196	0.0107	8.68E-73
OASL	12:121478277	0.074	0.00779	2.91E-21

OAT	10:126100850	-0.107	0.00682	4.45E-54
OAZ3	1:151741871	0.0207	0.003	5.30E-12
OBFC1	10:105613417	-0.0478	0.00539	1.12E-18
OCEL1	19:17335485	0.0516	0.00605	2.08E-17
OCLM	1:186381770	-0.064	0.00719	7.54E-19
ODF2L	1:86827633	-0.0602	0.00443	2.59E-41
OGFOD1	16:56491511	-0.037	0.00398	2.23E-20
OGFR	20:61443636	0.03	0.00346	5.42E-18
OGFRL1	6:71952461	0.102	0.00631	8.80E-58
OGG1	3:9823302	0.0167	0.00253	4.61E-11
OGN	9:95096811	-0.241	0.00527	<1.0E-314
OIP5	15:41590298	0.0577	0.00502	3.05E-30
OLA1	2:175090564	0.0387	0.00473	3.84E-16
OLFM4	13:53602462	0.232	0.01	3.38E-113
OLFML1	11:7521640	0.0494	0.00547	2.09E-19
OLIG1	21:34439206	-0.0548	0.00411	7.27E-40
OLIG2	21:34432792	-0.0372	0.00534	3.43E-12
OMD	9:95167578	-0.351	0.00954	1.64E-264
OPN3	1:241804070	0.0465	0.00475	1.70E-22
OPRL1	20:62692060	-0.0433	0.00552	5.39E-15
OPTN	10:13142780	-0.0936	0.0113	1.39E-16
OR10AD1	12:48557922	-0.151	0.00623	7.33E-123
OR13D1	9:107457532	-0.141	0.0113	4.60E-35
OR1J1	9:125254013	-0.0658	0.0063	3.08E-25
OR1L8	9:125338710	-0.0877	0.014	4.34E-10
OR1N1	9:125254013	-0.115	0.0145	2.75E-15
OR1N2	9:125359393	-0.084	0.00932	2.71E-19
OR2AE1	7:99481416	-0.129	0.00713	2.55E-71
OR2AK2	1:248128929	-0.204	0.00598	1.32E-230
OR2B11	1:247614617	-0.136	0.00614	1.76E-103
OR2L2	1:248196602	-0.266	0.0401	3.91E-11
OR2L8	1:248112627	0.249	0.013	5.01E-79
OR3A2	17:3203768	-0.0387	0.00461	6.79E-17
OR3A4P	17:3233600	-0.0408	0.00627	8.36E-11
OR4D1	17:56233639	0.115	0.0136	3.05E-17
OR52B6	11:5641710	-0.109	0.0074	2.77E-48
OR52K1	11:4491786	0.0967	0.0107	1.67E-19
OR52K2	11:4420543	-0.0962	0.00649	1.10E-48
OR52N4	11:5751392	-0.282	0.0233	2.55E-33

OR52W1	11:6221214	-0.112	0.00867	2.05E-37
OR56B1	11:5761819	-0.127	0.019	2.57E-11
OR6K3	1:158717628	0.0488	0.00659	1.45E-13
OR9A4	7:141639589	-0.127	0.0211	1.70E-09
ORAI2	7:102088338	-0.132	0.0104	2.58E-36
ORAOV1	11:69476293	0.0657	0.00437	5.68E-50
ORC3	6:88286127	0.0627	0.00962	7.56E-11
ORC4	2:148792900	-0.0558	0.00765	3.71E-13
ORM1	9:117078173	0.556	0.0164	9.37E-229
ORM2	9:117078286	-0.0998	0.00801	3.89E-35
ORMDL1	2:190597241	-0.0807	0.00423	2.31E-78
ORMDL3	17:38029120	0.099	0.00473	1.46E-93
OSBPL11	3:125313904	-0.0346	0.00435	2.31E-15
OSBPL1A	18:21924107	-0.039	0.00526	1.41E-13
OSBPL2	20:60845695	-0.0412	0.00357	1.91E-30
OSBPL3	7:25068398	0.0366	0.00388	5.34E-21
OSBPL5	11:3160207	-0.019	0.00236	1.04E-15
OSBPL6	2:179143827	0.0261	0.00303	9.11E-18
OSBPL8	12:76981753	-0.516	0.017	1.56E-187
OSBPL9	1:52047439	-0.0564	0.00707	1.78E-15
OSCAR	19:54549026	0.0694	0.0047	2.76E-48
OSCP1	1:36919898	-0.0236	0.00394	2.05E-09
OSGEP	14:20911822	-0.0843	0.00419	4.58E-87
OSGIN2	8:90989323	-0.0413	0.00446	3.28E-20
OSTF1	9:77756950	-0.0466	0.00432	7.28E-27
OTUB1	11:63710016	0.0912	0.0122	8.09E-14
OTUD6B	8:92086692	0.0709	0.0042	2.10E-62
OTX1	2:63263931	-0.043	0.00347	8.19E-35
OVCH1	12:29535715	-0.0673	0.00567	4.11E-32
OVGP1	1:111973893	-0.0221	0.00317	3.21E-12
OXA1L	14:23237835	-0.0375	0.00452	1.28E-16
OXCT1	5:41909115	-0.0333	0.00452	2.20E-13
OXNAD1	3:16303488	-0.0532	0.00757	2.32E-12
P2RX1	17:3797488	0.423	0.00906	<1.0E-314
P2RX4	12:121660302	-0.0906	0.00566	2.84E-56
P2RX5	17:3601495	-0.0588	0.00933	3.04E-10
P2RX7	12:121547431	0.148	0.0158	1.34E-20
P2RY12	3:151051983	-0.178	0.0103	1.20E-64
P2RY13	3:151056598	-0.0922	0.00748	2.00E-34

P2RY14	3:151010351	0.189	0.0126	2.43E-50
P4HA1	10:74796759	-0.11	0.00932	1.25E-31
P4HA2	5:131578608	0.0647	0.0073	1.06E-18
PAAF1	11:73611349	0.162	0.00854	1.27E-77
PABPC4	1:40035928	0.03	0.00432	4.41E-12
PABPN1	14:23797201	-0.0599	0.00825	4.29E-13
PACRGL	4:20714653	0.0525	0.00416	5.91E-36
PACS1	11:65854561	0.0401	0.00365	8.76E-28
PACSIN2	22:43343485	-0.031	0.00438	1.63E-12
PADI2	1:17397704	0.194	0.0047	<1.0E-314
PADI4	1:17619279	0.102	0.00581	2.98E-67
PAFAH1B2	11:117008345	0.109	0.00812	9.56E-41
PAFAH2	1:26330924	0.0333	0.00418	2.10E-15
PAG1	8:82034453	-0.128	0.00644	5.71E-85
PAK1	11:77060092	0.0777	0.00907	1.32E-17
PAK2	3:196496687	-0.0269	0.00387	4.09E-12
PALLD	4:169853125	0.0342	0.00311	8.85E-28
PAM	5:102151674	-0.379	0.00432	<1.0E-314
PAN2	12:56750872	-0.0517	0.00739	3.03E-12
PAN3	13:28771621	-0.0503	0.00625	1.11E-15
PANK1	10:91326997	-0.0209	0.00282	1.64E-13
PANK2	20:3873092	0.0522	0.00388	1.91E-40
PANK3	5:168020360	-0.0221	0.00345	1.66E-10
PANX1	11:93862020	0.122	0.00447	1.12E-152
PAPD5	16:50277613	-0.039	0.00657	3.30E-09
PAPD7	5:6710582	0.0677	0.0113	2.54E-09
PAPOLA	14:97045171	0.043	0.00411	2.21E-25
PAPSS1	4:108609143	-0.361	0.00537	<1.0E-314
PAPSS2	10:89419143	0.0434	0.00431	1.27E-23
PAQR6	1:156208230	0.122	0.00301	<1.0E-314
PAQR8	6:52198785	0.0593	0.00574	8.24E-25
PARD3B	2:206246134	-0.0254	0.00312	4.91E-16
PARK7	1:8056453	0.0361	0.00346	3.44E-25
PARL	3:183558402	0.0595	0.00399	3.40E-49
PARP1	1:226609436	-0.0473	0.00549	9.30E-18
PARP11	12:3937663	0.05	0.00663	5.80E-14
PARP14	3:122426474	0.153	0.0146	2.02E-25
PARP15	3:122296834	0.121	0.00628	1.58E-79
PARP16	15:65565632	0.0622	0.00365	2.54E-63

PARP2	14:20812467	-0.0512	0.00491	3.28E-25
PARP4	13:25049203	0.061	0.00475	3.68E-37
PARP8	5:49956668	0.0794	0.00545	4.42E-47
PARS2	1:55231955	-0.0198	0.00307	1.26E-10
PART1	5:59837767	0.0478	0.00793	1.81E-09
PARVA	11:12388984	0.0288	0.00458	3.68E-10
PARVB	22:44424191	-0.0432	0.00418	1.05E-24
PARVG	22:44637473	0.0507	0.00734	5.80E-12
PASK	2:242044910	0.0968	0.00318	1.35E-187
PATZ1	22:31750013	0.0269	0.00371	4.88E-13
PAX8	2:113993385	0.0539	0.0023	2.21E-115
PBLD	10:70042115	0.0443	0.00715	6.36E-10
PBX1	1:164520400	-0.0764	0.00948	9.44E-16
PBX2	6:32153409	-0.0682	0.00747	9.19E-20
PBX3	9:128593132	0.0217	0.00367	3.56E-09
PBXIP1	1:154888348	-0.0199	0.00291	9.26E-12
PCBP3	21:47309593	-0.0302	0.0031	2.67E-22
PCCA	13:101149072	-0.0273	0.00404	1.54E-11
PCCB	3:136056861	-0.0292	0.0029	1.21E-23
PCDH12	5:141328745	0.0263	0.0029	1.64E-19
PCDH8	13:53409474	-0.0287	0.00435	4.40E-11
PCDH9	13:67787267	0.0365	0.00615	3.13E-09
PCGF2	17:36904739	-0.0343	0.00338	6.27E-24
PCGF3	4:783314	-0.111	0.00383	2.41E-171
PCID2	13:113840822	-0.0908	0.0113	1.33E-15
PCK2	14:24525274	-0.0262	0.0036	3.40E-13
PCM1	8:17778924	-0.0735	0.00425	4.00E-65
PCMT1	6:150093682	-0.0411	0.00417	1.05E-22
PCMTD1	8:52732154	0.0492	0.00532	3.10E-20
PCMTD2	20:62905851	0.143	0.00696	5.28E-90
PCNA	20:5113847	0.0703	0.00898	5.95E-15
PCNT	21:47793235	-0.0387	0.00297	2.81E-38
PCNX	14:71353205	0.0955	0.00589	1.02E-57
PCNXL4	14:60641082	-0.0544	0.00499	2.45E-27
PCSK5	9:78487243	-0.0354	0.00432	3.34E-16
PCSK7	11:117096652	-0.0348	0.00427	4.20E-16
PCTP	17:53838522	-0.0636	0.00814	6.43E-15
PCYOX1L	5:148764710	-0.0569	0.00329	4.43E-65
PCYT1A	3:195952900	-0.0693	0.00408	4.47E-63

PDC	1:186407267	-0.193	0.00896	1.60E-98
PDCD1LG2	9:5466085	-0.0762	0.00856	7.54E-19
PDCD4	10:112658148	0.0585	0.0078	7.77E-14
PDCD5	19:33074614	-0.0417	0.005	9.92E-17
PDCD6IP	3:33882134	0.056	0.00288	1.84E-81
PDCD7	15:65411709	-0.249	0.0229	2.70E-27
PDE1B	12:54944618	-0.0398	0.0043	2.81E-20
PDE3B	11:14768384	-0.0444	0.00436	4.11E-24
PDE4B	1:66841180	0.0234	0.00392	2.60E-09
PDE4D	5:59544653	-0.072	0.00364	2.92E-84
PDE6D	2:232617205	0.0475	0.005	2.89E-21
PDE6H	12:15097497	-0.0525	0.0082	1.62E-10
PDE7A	8:66732061	-0.0484	0.00543	6.21E-19
PDE8A	15:85520329	0.0356	0.00507	2.46E-12
PDE8B	5:76733379	0.0266	0.0027	1.14E-22
PDE9A	21:44158405	-0.0751	0.00311	2.25E-122
PDGFC	4:157674780	0.0495	0.00518	1.69E-21
PDGFD	11:104023820	-0.0938	0.0144	8.66E-11
PDHB	3:58375403	0.0358	0.00367	2.90E-22
PDHX	11:34982148	-0.0529	0.00426	8.41E-35
PDIA5	3:122841062	0.0237	0.00341	4.33E-12
PDIA6	2:10933249	0.0558	0.00373	1.66E-49
PDK1	2:173371508	0.132	0.0071	3.79E-75
PDK2	17:48158049	-0.0612	0.00773	2.96E-15
PDK4	7:95173570	-0.0671	0.00718	1.31E-20
PDLIM1	10:97037578	-0.042	0.00698	1.83E-09
PDLIM5	4:95568956	-0.0265	0.00371	1.16E-12
PDP1	8:94916102	-0.0429	0.00435	1.00E-22
PDPR	16:70117111	0.136	0.021	1.24E-10
PDS5A	4:39820977	0.0277	0.00278	3.36E-23
PDSS1	10:26983410	-0.0544	0.00646	4.47E-17
PDXK	21:45152500	-0.0835	0.00389	6.37E-98
PDZD8	10:119154977	-0.154	0.00394	1.20E-293
PDZK1IP1	1:47655816	0.494	0.0165	1.49E-181
PEA15	1:160141286	-0.0413	0.00638	1.04E-10
PEBP1	12:118583232	-0.149	0.00463	3.44E-208
PECR	2:216887593	0.053	0.00502	7.90E-26
PEG10	7:94253617	0.0253	0.0031	4.72E-16
PELI1	2:64318988	-0.11	0.0131	4.43E-17

PELI2	14:56585620	0.0233	0.00308	5.00E-14
PELI3	11:66258006	0.016	0.00274	5.13E-09
PER3	1:7897228	-0.0629	0.0045	1.35E-43
PERP	6:138412313	0.109	0.00754	1.22E-46
PES1	22:30993934	-0.027	0.00405	2.93E-11
PEX1	7:92182429	-0.064	0.0109	4.09E-09
PEX14	1:10580275	0.0196	0.00257	2.40E-14
PEX5	12:7329996	-0.0337	0.00327	1.10E-24
PEX6	6:42897074	0.122	0.00294	<1.0E-314
PEX7	6:137205972	0.0356	0.00539	4.39E-11
PF4V1	4:74713950	-1.08	0.0224	<1.0E-314
PFKFB2	1:207245080	0.0343	0.00563	1.18E-09
PFKM	12:48582520	-0.0231	0.00292	3.17E-15
PFKP	10:3138996	-0.0599	0.00366	1.13E-58
PFN2	3:149692597	0.137	0.00622	2.92E-102
PGAM2	7:44071980	0.073	0.012	1.42E-09
PGAP1	2:197808980	0.0736	0.00968	3.36E-14
PGAP2	11:3858616	-0.0256	0.00361	1.57E-12
PGAP3	17:37829571	0.0228	0.00325	2.38E-12
PGBD4	15:34388399	-0.0698	0.01	4.06E-12
PGGT1B	5:114548071	0.256	0.0089	1.19E-168
PGLYRP1	19:46520945	0.0592	0.0046	2.41E-37
PGM1	1:64128758	-0.0244	0.00313	6.79E-15
PGM2	4:37846643	-0.0573	0.00531	8.09E-27
PGM2L1	11:74039452	-0.0781	0.00681	4.07E-30
PGM3	6:83828724	0.0581	0.00452	2.93E-37
PGM5	9:71175565	-0.24	0.00613	2.79E-294
PGPEP1	19:18499238	0.0392	0.00526	1.08E-13
PGS1	17:76383671	0.0498	0.00534	1.51E-20
PHF10	6:170075364	0.0667	0.00626	3.04E-26
PHF11	13:50105215	-0.0982	0.00531	3.26E-74
PHF17	4:129817261	-0.0368	0.00357	1.32E-24
PHF19	9:123605711	-0.0292	0.00452	1.11E-10
PHF20L1	8:133791175	0.0604	0.00316	5.00E-79
PHF5A	22:41909618	-0.0616	0.00552	1.23E-28
PHGDH	1:120255370	-0.035	0.0042	1.02E-16
PHIP	6:79595097	0.0418	0.00337	9.41E-35
PHKB	16:47526756	0.107	0.00957	1.07E-28
PHLDB2	3:111617861	-0.028	0.00456	9.33E-10

PHLPP1	18:60595572	0.0682	0.00376	3.47E-71
PHOSPHO1	17:47349743	-0.0619	0.00872	1.48E-12
PHOSPHO2	2:170600408	0.0427	0.00661	1.14E-10
PHTF2	7:77540136	0.0438	0.00715	9.62E-10
PHYH	10:13363046	0.0325	0.0044	1.71E-13
PI16	6:36915094	-0.162	0.00656	3.30E-128
PI3	20:43803708	0.403	0.0156	3.65E-138
PI4K2A	10:99385842	-0.0341	0.00577	3.83E-09
PI4K2B	4:25251215	-0.0562	0.00482	5.55E-31
PIAS2	18:44494527	-0.0776	0.00391	1.04E-84
PIAS4	19:3989692	-0.027	0.00409	4.46E-11
PICALM	11:85690012	-0.0505	0.00647	7.47E-15
PICK1	22:38456680	0.0614	0.00418	6.35E-48
PID1	2:230182148	0.197	0.0134	8.82E-48
PIEZO1	16:88783449	0.0239	0.00276	5.44E-18
PIGB	15:55570932	-0.044	0.00575	2.52E-14
PIGC	1:172425529	0.0713	0.00416	2.74E-64
PIGF	2:46818992	0.0589	0.00765	1.66E-14
PIGG	4:525060	-0.0449	0.00397	2.76E-29
PIGK	1:77731287	0.0349	0.00517	1.54E-11
PIGM	1:159983400	-0.158	0.012	1.08E-38
PIGN	18:59814324	-0.118	0.0045	5.36E-142
PIGP	21:38452379	0.0608	0.00441	1.56E-42
PIGU	20:33220196	0.0297	0.00427	4.38E-12
PIGV	1:27172633	0.348	0.0272	4.48E-37
PIGW	17:34843839	0.0708	0.00529	4.32E-40
PIGX	3:196430450	0.159	0.011	1.09E-46
PIGY	4:89417500	-0.074	0.00722	1.81E-24
PIH1D1	19:49960479	-0.0826	0.00463	3.46E-69
PIK3C2A	11:17115254	-0.174	0.0066	8.52E-144
PIK3C3	18:39530811	0.0558	0.00456	6.72E-34
PIK3CA	3:178925321	0.038	0.00434	2.99E-18
PIK3IP1	22:31659827	0.0441	0.00444	4.46E-23
PIK3R3	1:46595868	0.232	0.00648	1.14E-251
PILRB	7:99947657	-0.152	0.00475	1.75E-204
PIM1	6:37142422	0.0494	0.00682	4.76E-13
PINK1	1:20949488	0.0572	0.00574	3.35E-23
PINX1	8:10623884	0.0354	0.0056	2.77E-10
PIP4K2A	10:22826093	0.0713	0.00723	1.01E-22

PIP4K2B	17:36926731	0.0583	0.00335	7.39E-66
PISD	22:31982241	-0.125	0.00338	1.31E-264
PITPNA	17:1464466	-0.129	0.00667	3.71E-80
PITPNB	22:28357674	-0.0405	0.00658	8.36E-10
PITPNC1	17:65381347	-0.0493	0.00578	2.00E-17
PITRM1	10:3215646	-0.0574	0.00337	1.80E-63
PIWIL4	11:94323376	-0.0243	0.00348	3.20E-12
PJA2	5:108752743	0.0743	0.00353	1.59E-94
PKD1L1	7:48023705	0.0154	0.00154	2.35E-23
PKD1L3	16:72051369	-0.0172	0.00237	4.37E-13
PKD2	4:88933519	-0.305	0.0128	9.64E-119
PKD2L1	10:102044312	-0.0326	0.00543	1.95E-09
PKD2L2	5:137244311	-0.0157	0.00213	1.51E-13
PKDREJ	22:46652372	0.0401	0.00408	1.36E-22
PKHD1L1	8:110582366	0.0341	0.00521	6.69E-11
PKIA	8:79482385	0.0579	0.00402	3.33E-46
PKIB	6:123017838	0.0329	0.00429	1.86E-14
PKNOX1	21:44473867	0.12	0.00526	9.45E-111
PKP2	12:33057444	-0.0252	0.0036	3.31E-12
PKP4	2:159332885	0.0514	0.0045	7.67E-30
PLA2G16	11:63385451	0.163	0.0127	2.67E-37
PLA2G4A	1:186947729	-0.0522	0.00466	8.67E-29
PLA2G4C	19:48622545	-0.131	0.00477	6.63E-156
PLA2G7	6:46745076	0.495	0.0266	3.96E-75
PLAC4	21:42521161	0.0695	0.00598	7.29E-31
PLAGL1	6:144286687	-0.384	0.0195	1.95E-83
PLAUR	19:44151621	0.0375	0.00615	1.09E-09
PLB1	2:28677086	-0.53	0.00938	<1.0E-314
PLBD1	12:14742575	-0.349	0.0437	1.80E-15
PLCB1	20:8408544	0.145	0.00508	6.59E-167
PLCB3	11:64044284	0.0146	0.00218	2.63E-11
PLCG2	16:81964977	0.0382	0.00421	1.61E-19
PLCL1	2:198882952	0.16	0.00562	1.54E-166
PLCL2	3:16908186	-0.0503	0.00584	9.81E-18
PLD1	3:171546161	-0.039	0.00511	2.72E-14
PLD2	17:4721376	-0.148	0.0096	2.68E-52
PLD3	19:40851678	0.0187	0.00268	3.58E-12
PLD6	17:17158343	-0.088	0.00425	1.11E-91
PLEC	8:145031968	0.028	0.00164	2.58E-63

PLEK	2:68637710	0.148	0.00453	2.39E-214
PLEKHA1	10:124192430	-0.0982	0.00492	1.49E-85
PLEKHA3	2:179368982	0.0473	0.00568	9.95E-17
PLEKHA5	12:19317082	0.102	0.00536	7.63E-78
PLEKHA7	11:17025712	-0.0162	0.00222	2.90E-13
PLEKHF2	8:96209862	-0.155	0.0137	1.98E-29
PLEKHG2	19:39967355	0.0284	0.0033	1.07E-17
PLEKHG6	12:6451590	-0.0135	0.00186	4.56E-13
PLEKHH2	2:43863274	-0.0647	0.00228	4.14E-165
PLEKHM1	17:43493834	-0.0938	0.0068	1.56E-42
PLEKHO2	15:65117709	-0.0601	0.00331	2.42E-71
PLGRKT	9:5383460	-0.0514	0.00619	1.16E-16
PLIN2	9:19127489	-0.205	0.00593	1.61E-236
PLIN3	19:4847874	-0.0443	0.00605	2.93E-13
PLK1	16:23697679	-0.057	0.00453	1.06E-35
PLK1S1	20:21142523	-0.136	0.00443	3.99E-189
PLN	6:118885950	0.15	0.00623	3.28E-122
PLOD1	1:12048595	-0.0929	0.003	6.29E-194
PLRG1	4:155456390	0.0325	0.00377	8.38E-18
PLS1	3:142396296	-0.119	0.0164	5.79E-13
PLSCR1	3:146250397	0.145	0.00922	8.44E-55
PLSCR3	17:7301193	0.0373	0.00467	1.63E-15
PLTP	20:44540178	-0.0228	0.00252	2.07E-19
PLVAP	19:17428176	-0.0747	0.00999	9.09E-14
PLXDC1	17:37248796	0.0606	0.00406	2.18E-49
PLXDC2	10:20108228	0.0875	0.00578	9.89E-51
PLXNA2	1:208408890	0.0178	0.00243	2.45E-13
PLXNC1	12:94517858	-0.0551	0.00714	1.50E-14
PMF1	1:156206121	0.0189	0.00296	1.95E-10
PMM2	16:8930666	0.0877	0.0061	4.29E-46
PMP22	17:15147127	-0.033	0.00558	3.41E-09
PMS1	2:190620957	-0.0704	0.00464	7.77E-51
PMS2	7:6056901	-0.113	0.00645	1.50E-66
PNKD	2:219195799	0.0251	0.00313	1.32E-15
PNKP	19:50368361	-0.0184	0.00182	9.24E-24
PNLDC1	6:160206106	-0.0514	0.00647	2.50E-15
PNMAL1	19:46974752	-0.0359	0.00523	7.84E-12
PNN	14:39651193	-0.0248	0.00404	8.76E-10
PNP	14:20940626	0.0815	0.00904	2.49E-19

PNPLA1	6:36258667	0.0317	0.004	3.23E-15
PNPLA8	7:108101701	-0.167	0.0154	4.41E-27
PNRC1	6:89783713	-0.0334	0.00332	1.36E-23
POC1B	12:89907010	0.205	0.00701	6.31E-174
POC5	5:74998892	-0.091	0.00478	4.83E-78
POFUT2	21:46655385	-0.0585	0.00625	1.08E-20
POGLUT1	3:119143383	-0.142	0.00747	5.32E-78
POGZ	1:151414924	-0.0611	0.00776	4.08E-15
POLDIP3	22:42982275	-0.0583	0.00825	1.71E-12
POLE2	14:50133218	0.0626	0.00837	8.53E-14
POLE4	2:75185856	-0.0736	0.00526	1.36E-43
POLI	18:51779019	-0.174	0.00405	<1.0E-314
POLM	7:44111258	0.0322	0.00329	1.87E-22
POLN	4:2223070	-0.0446	0.00561	2.29E-15
POLR1A	2:86332656	0.0556	0.00353	8.72E-55
POLR1B	2:113300283	-0.129	0.00635	3.18E-88
POLR1D	13:28172776	0.212	0.00688	2.01E-191
POLR1E	9:37490307	0.13	0.00593	4.36E-102
POLR2B	4:57895214	-0.0293	0.00294	2.79E-23
POLR2C	16:57515188	-0.0276	0.0038	4.57E-13
POLR2D	2:128615390	0.024	0.00389	7.10E-10
POLR2J	7:102085206	0.343	0.0161	7.73E-97
POLR2L	11:837661	-0.431	0.0201	5.12E-98
POLR3B	12:106729425	-0.0961	0.0104	3.00E-20
POLR3E	16:22345191	-0.0206	0.00298	4.77E-12
POLR3F	20:18464673	-0.046	0.0054	2.14E-17
POLR3K	16:136142	0.131	0.0104	7.58E-36
POM121	7:72300332	-0.364	0.0291	2.82E-35
POMT1	9:134389070	-0.0372	0.00413	3.05E-19
POMT2	14:77754530	-0.0285	0.00468	1.16E-09
PON2	7:95063702	-0.0577	0.0053	2.48E-27
POP5	12:120994872	-0.0931	0.00579	6.31E-57
POPDC2	3:119375441	0.083	0.00393	3.82E-95
POR	7:75664921	-0.03	0.00335	5.30E-19
PP2D1	3:20021515	0.0792	0.00827	1.58E-21
PP7080	5:466458	0.0651	0.0077	3.79E-17
PPA1	10:72002499	-0.136	0.00652	1.15E-92
PPA2	4:106325900	-0.0526	0.00288	3.57E-72
PPAPDC2	9:4670187	-0.0855	0.00596	9.90E-46

PPARG	3:12511376	-0.0218	0.00291	8.56E-14
PPARGC1A	4:23868647	-0.116	0.00498	6.75E-115
PPAT	4:57230687	0.0817	0.00494	7.29E-60
PPCS	1:42910624	0.0441	0.00436	8.60E-24
PPDPF	20:62162917	0.113	0.0159	1.13E-12
PPFIA1	11:70238030	0.0589	0.00384	4.04E-52
PPFIBP2	11:7548423	0.107	0.00469	1.81E-110
PPHLN1	12:42854205	-0.0355	0.00306	9.84E-31
PPIA	7:44836314	-0.0619	0.00744	1.14E-16
PPIE	1:40200894	-0.314	0.00692	<1.0E-314
PPIF	10:81103247	-0.0595	0.00351	9.48E-63
PPIG	2:170457451	0.0517	0.00431	1.08E-32
PPIH	1:43111741	0.0559	0.00686	4.75E-16
PPIL2	22:22049783	-0.0263	0.00275	1.67E-21
PPIL3	2:201730687	0.28	0.0067	<1.0E-314
PPIL4	6:149811693	0.0925	0.00405	4.80E-110
PPIP5K2	5:102415468	0.206	0.00428	<1.0E-314
PPL	16:4949815	-0.0386	0.00231	2.12E-61
PPM1F	22:22301254	0.0742	0.00371	9.27E-86
PPM1K	4:89150178	-0.0486	0.00693	2.48E-12
PPM1L	3:160512511	0.0942	0.0134	2.59E-12
PPM1M	3:52287468	-0.0216	0.00332	8.90E-11
PPM1N	19:46006212	0.0349	0.00585	2.66E-09
PPP1CC	12:111187119	0.14	0.0155	2.72E-19
PPP1R11	6:29986684	-0.0659	0.00594	2.94E-28
PPP1R14A	19:38741577	-0.0949	0.0123	1.13E-14
PPP1R15A	19:49378328	0.0621	0.00652	2.56E-21
PPP1R17	7:31728180	0.038	0.00446	2.23E-17
PPP1R2	3:195284917	-0.381	0.0149	3.01E-136
PPP1R21	2:48682513	0.0405	0.00471	1.06E-17
PPP1R3B	8:8969374	-0.0506	0.00606	8.89E-17
PPP1R7	2:242120608	-0.0648	0.00471	2.41E-42
PPP2CB	8:30649380	-0.273	0.0115	1.50E-117
PPP2R1B	11:111621399	0.0579	0.0031	1.94E-75
PPP2R2D	10:133766943	0.0279	0.00467	2.56E-09
PPP2R3A	3:135800693	-0.0192	0.00284	1.34E-11
PPP2R3C	14:35625217	0.194	0.00534	3.39E-259
PPP2R4	9:131884041	0.07	0.00434	4.37E-57
PPP2R5A	1:212440206	-0.21	0.00524	2.02E-306

PPP2R5C	14:102369553	-0.0302	0.00411	2.63E-13
PPP3CA	4:102268940	0.0286	0.00358	1.55E-15
PPP4R1	18:9626179	0.0733	0.00507	1.61E-46
PPP4R4	14:94616008	-0.0801	0.00299	1.84E-148
PPP5C	19:46860562	0.0914	0.0031	6.49E-177
PPP6C	9:127952746	0.0312	0.00304	1.49E-24
PPT1	1:40559052	0.434	0.00862	<1.0E-314
PPT2	6:32113571	-0.0182	0.00274	3.44E-11
PPTC7	12:110996263	0.04	0.00578	5.02E-12
PPWD1	5:64828559	-0.068	0.00383	1.04E-68
PQLC1	18:77711157	0.0355	0.00424	7.34E-17
PQLC3	2:11325331	-0.0457	0.00392	3.87E-31
PRAM1	19:8540159	0.0351	0.00413	2.21E-17
PRC1	15:91525197	0.0625	0.00631	6.59E-23
PRDM15	21:43220036	-0.0345	0.00343	1.47E-23
PRDM4	12:108082066	-0.0587	0.0038	1.19E-52
PRDM5	4:121693006	0.207	0.00604	1.52E-231
PRDM8	4:81132765	-0.0344	0.00313	7.29E-28
PRDX1	1:45987574	0.0592	0.00767	1.44E-14
PRDX3	10:120952037	-0.0512	0.00456	6.32E-29
PRDX5	11:64082807	-0.25	0.00694	4.48E-254
PRDX6	1:173484385	-0.301	0.00916	2.25E-215
PRELID2	5:145210955	-0.0467	0.00784	2.80E-09
PREP	6:105776312	0.0231	0.00391	3.65E-09
PREX1	20:47373809	0.0724	0.0047	1.94E-52
PRG4	1:186291941	0.0409	0.00538	3.55E-14
PRH1	12:11072615	-0.258	0.0115	3.42E-106
PRH2	12:11131212	0.224	0.0116	7.67E-80
PRICKLE1	12:43002908	0.0681	0.0111	1.02E-09
PRICKLE4	6:41782107	-0.0269	0.00325	1.49E-16
PRIM1	12:57144533	-0.122	0.00476	1.10E-136
PRKAB1	12:120146925	-0.0684	0.0064	2.29E-26
PRKAB2	1:146632970	-0.0673	0.00426	7.40E-55
PRKAG2	7:151250455	-0.0361	0.00366	1.00E-22
PRKAG3	2:219666228	-0.0143	0.00224	1.89E-10
PRKAR2B	7:106655758	0.112	0.0178	4.02E-10
PRKCA	17:64283468	0.0346	0.00551	3.52E-10
PRKCB	16:23865170	0.0898	0.00331	9.36E-152
PRKCD	3:53205494	-0.0373	0.00506	1.93E-13

PRKCDBP	11:6343486	-0.0291	0.00492	3.26E-09
PRKCE	2:45886261	-0.0212	0.0036	4.09E-09
PRKD1	14:30314078	-0.0339	0.00289	1.96E-31
PRKD2	19:47205707	-0.0611	0.00365	3.00E-61
PRKD3	2:37479959	-0.0758	0.00378	1.92E-86
PRKG1	10:53673125	0.0445	0.00438	4.27E-24
PRKG2	4:82082419	0.0984	0.00446	3.26E-103
PRKRA	2:179340710	-0.0793	0.0109	4.32E-13
PRKRIP1	7:102086396	0.145	0.0144	1.49E-23
PRMT2	21:48039367	-0.0903	0.00411	1.67E-102
PRMT5	14:23422186	0.166	0.00465	1.46E-250
PRMT6	1:107596443	0.0598	0.00509	1.77E-31
PROCA1	17:27053176	0.0282	0.00473	2.55E-09
PROK2	3:71854587	0.128	0.00943	4.63E-41
PROS1	3:93639320	0.135	0.012	5.14E-29
PRPF18	10:13653653	0.0846	0.00403	3.19E-94
PRPF19	11:60633250	0.0186	0.0029	1.76E-10
PRPF6	20:62667554	-0.0675	0.00636	5.40E-26
PRPF8	17:1574532	0.0327	0.00522	3.99E-10
PRPSAP1	17:74293388	-0.056	0.00401	1.68E-43
PRPSAP2	17:18783052	0.0666	0.00442	2.89E-50
PRR13	12:53815974	-0.0858	0.00935	6.31E-20
PRR4	12:11051749	-0.0612	0.00546	7.02E-29
PRR5-ARHGAP8	22:45262790	0.0198	0.00291	1.23E-11
PRR5L	11:36432830	0.101	0.0151	2.61E-11
PRRC2A	6:31591918	-0.0384	0.0023	7.54E-61
PRRC2B	9:134346315	-0.0553	0.00899	8.19E-10
PRRG4	11:32891548	-0.134	0.00763	1.86E-67
PRSS21	16:2872116	0.0457	0.00732	4.61E-10
PRSS30P	16:2911320	0.0525	0.00406	1.37E-37
PRUNE	1:151006539	-0.0515	0.00669	1.53E-14
PRUNE2	9:79304020	-0.299	0.00548	<1.0E-314
PSAP	10:73555926	-0.0349	0.00333	2.09E-25
PSAT1	9:80966916	-0.0722	0.0101	8.43E-13
PSD4	2:113943470	0.0694	0.00284	1.35E-124
PSEN1	14:73662629	0.0759	0.00392	1.28E-80
PSEN2	1:227042462	-0.0198	0.00282	2.77E-12
PSIP1	9:15441648	0.0289	0.00474	1.18E-09
PSMA2	7:42975857	-0.0585	0.00758	1.48E-14

PSMA4	15:78814681	0.101	0.00594	1.35E-62
PSMB10	16:67936975	0.065	0.00709	6.42E-20
PSMB3	17:36927055	-0.0828	0.00791	2.11E-25
PSMB4	1:151406375	0.0778	0.00504	1.68E-52
PSMB7	9:127163623	-0.0312	0.00357	2.75E-18
PSMB8	6:32811645	-0.0706	0.0114	5.59E-10
PSMB9	6:32830013	-0.0519	0.00463	7.74E-29
PSMC3IP	17:40686342	-0.0268	0.00407	4.59E-11
PSMC4	19:40486606	0.0791	0.0106	1.23E-13
PSMC5	17:61948203	-0.025	0.00422	3.06E-09
PSMD12	17:65332728	0.0472	0.00429	8.13E-28
PSMD13	11:202072	-0.144	0.00341	<1.0E-314
PSMD2	3:184011515	-0.0316	0.00495	1.82E-10
PSMD3	17:38144241	-0.0197	0.00282	3.08E-12
PSMD5	9:123610288	0.0516	0.00395	1.73E-38
PSME2	14:24604399	0.0775	0.0119	8.63E-11
PSME4	2:54198181	0.0663	0.00829	1.55E-15
PSMF1	20:1130627	0.102	0.0155	5.38E-11
PSMG1	21:40536790	0.116	0.00447	1.08E-139
PSMG2	18:12672008	0.0478	0.00317	3.25E-50
PSPC1	13:20352275	0.0621	0.00349	1.34E-68
PSRC1	1:109817590	-0.0179	0.00293	9.72E-10
PSTPIP2	18:43569556	-0.174	0.0104	5.48E-61
PTBP2	1:97266007	0.0568	0.00383	7.71E-49
PTBP3	9:115008349	0.0321	0.00357	3.47E-19
PTER	10:16482481	0.0632	0.00384	2.28E-59
PTGDR	14:52687634	-0.0686	0.00843	5.24E-16
PTGER2	14:52781195	0.0501	0.00693	5.59E-13
PTGES	9:132542547	-0.107	0.00984	3.41E-27
PTGFR	1:79002544	0.0366	0.00539	1.25E-11
PTGIR	19:47123402	0.0306	0.00496	7.20E-10
PTGR1	9:114376753	0.0285	0.0044	9.57E-11
PTGR2	14:74318714	-0.256	0.0102	1.71E-131
PTK2B	8:27195121	-0.0677	0.00311	1.48E-100
PTP4A1	6:64241021	0.0612	0.0102	2.34E-09
PTP4A2	1:32410737	-0.0661	0.00761	4.99E-18
PTPLA	10:17707993	-0.0421	0.00431	2.29E-22
PTPN12	7:77300726	0.0815	0.00428	4.33E-78
PTPN18	2:131112343	0.0789	0.00658	1.07E-32

PTPN2	18:12857002	0.02	0.00338	3.81E-09
PTPN22	1:114425590	-0.0668	0.00408	8.84E-59
PTPN7	1:202171923	0.0434	0.00485	4.81E-19
PTPRB	12:70889323	0.0253	0.00392	1.26E-10
PTPRE	10:129845903	0.0705	0.0037	2.77E-78
PTPRJ	11:48013484	-0.0996	0.00512	1.70E-81
PTPRM	18:8019465	0.144	0.00688	2.70E-93
PTPRN2	7:157289684	-0.0777	0.0052	2.33E-49
PTPRS	19:5207292	-0.0223	0.0035	2.18E-10
PTTG1IP	21:46294986	-0.0266	0.0032	1.18E-16
PTTG2	4:37962186	-0.299	0.0102	2.72E-176
PTTG3P	8:67670011	0.273	0.0306	6.88E-19
PUM1	1:31577776	-0.0268	0.00404	3.61E-11
PUM2	2:20532201	0.0712	0.00561	1.89E-36
PUS10	2:61244410	-0.0965	0.00553	3.00E-66
PUS3	11:125776259	-0.068	0.00814	8.66E-17
PUS7	7:105162293	-0.12	0.00826	1.12E-46
PUS7L	12:44082341	0.0331	0.00461	8.58E-13
PVALB	22:37249491	-0.252	0.0157	1.47E-56
PVR	19:45122146	0.0349	0.00424	2.36E-16
PVRIG	7:99804125	-0.147	0.00748	5.69E-83
PVRL2	19:45371188	-0.667	0.0155	<1.0E-314
PWP1	12:108075138	-0.0816	0.00491	1.97E-60
PWP2	21:45490684	0.0443	0.00282	2.14E-54
PXDC1	6:3715608	-0.066	0.0101	5.80E-11
PXK	3:58411088	0.0979	0.00519	1.09E-76
PYGB	20:25241345	0.0436	0.00267	1.46E-58
PYGL	14:51389918	-0.0436	0.00644	1.37E-11
PYHIN1	1:158910194	0.0524	0.0073	7.92E-13
PYROXD2	10:100144007	-0.0315	0.00249	3.73E-36
PZP	12:9258821	0.226	0.00866	8.43E-141
QDPR	4:17518920	0.174	0.00488	1.97E-249
QKI	6:163894045	0.219	0.00701	1.77E-197
QRICH1	3:49106536	0.0431	0.00575	8.05E-14
QRSL1	6:107077502	0.034	0.00531	1.62E-10
QSER1	11:32911368	-0.154	0.0129	2.77E-32
QSOX1	1:180140101	-0.0578	0.00289	1.13E-85
QTRTD1	3:113785149	-0.0539	0.00523	1.03E-24
R3HCC1L	10:99955345	0.027	0.00368	2.62E-13

R3HDM1	2:136288273	-0.0316	0.0041	1.39E-14
RAB11FIP3	16:518074	-0.0249	0.00281	1.03E-18
RAB11FIP4	17:29821612	0.215	0.00995	2.96E-99
RAB14	9:123961418	0.0324	0.00345	8.79E-21
RAB15	14:65449458	-0.0875	0.0115	3.58E-14
RAB18	10:27851166	0.0404	0.005	7.60E-16
RAB1B	11:66040049	-0.0271	0.00363	1.03E-13
RAB20	13:111175244	-0.075	0.00398	6.66E-77
RAB23	6:57069606	-0.145	0.00835	2.03E-65
RAB27A	15:55502516	0.0467	0.00476	1.58E-22
RAB2A	8:61525780	-0.0437	0.00442	7.85E-23
RAB2B	14:21927638	0.111	0.012	4.28E-20
RAB31	18:9850581	-0.0513	0.00472	3.11E-27
RAB34	17:27054641	0.0467	0.00656	1.23E-12
RAB38	11:87931463	-0.438	0.0207	2.72E-95
RAB3GAP2	1:220409506	0.0544	0.00467	4.56E-31
RAB3IP	12:70145471	-0.262	0.0183	9.72E-46
RAB44	6:36698294	0.0567	0.00387	1.57E-47
RAB5A	3:20045701	-0.0823	0.00393	1.07E-93
RAB5C	17:40297658	0.0325	0.00443	2.54E-13
RAB6B	3:133602195	0.0205	0.00321	1.95E-10
RAB7L1	1:205757798	-0.045	0.00443	4.89E-24
RAB8A	19:16223287	0.0662	0.00471	4.93E-44
RABAC1	19:42441795	-0.0855	0.00553	7.52E-53
RABEP1	17:5302203	0.0354	0.00343	8.74E-25
RABEPK	9:127914865	0.0501	0.00326	2.60E-52
RABGAP1	9:125851363	-0.036	0.00426	4.05E-17
RABGAP1L	1:174086292	0.0291	0.00314	2.66E-20
RABGGTA	14:24733689	0.0315	0.00343	5.67E-20
RAC1	7:6416360	-0.247	0.00641	7.95E-286
RAC2	22:37619290	0.0357	0.0039	6.56E-20
RAD17	5:68733107	0.054	0.00755	9.77E-13
RAD18	3:9005078	-0.0478	0.00366	1.57E-38
RAD23B	9:110094733	0.059	0.00417	1.39E-44
RAD51AP1	12:4665500	-0.185	0.0289	1.60E-10
RAD51C	17:56725052	-0.302	0.0053	<1.0E-314
RAD51D	17:33493071	-0.0216	0.00348	5.35E-10
RAD52	12:1058738	0.0967	0.00599	2.97E-57
RAD54B	8:95440423	-0.101	0.00359	3.94E-161

RAE1	20:55953682	-0.0231	0.00388	2.79E-09
RAF1	3:12690343	-0.0279	0.00412	1.33E-11
RALA	7:39706470	-0.054	0.00323	4.99E-61
RALB	2:121003612	0.119	0.00636	1.10E-75
RALBP1	18:9488704	-0.0782	0.00442	4.27E-68
RALGAPA1	14:36132999	-0.0962	0.0104	3.40E-20
RALGDS	9:135966340	-0.082	0.00814	1.18E-23
RALGPS1	9:129672061	-0.0481	0.00305	9.66E-55
RAMP3	7:45257184	-0.0469	0.00635	1.85E-13
RAN	12:131364071	0.0237	0.00344	5.81E-12
RANBP17	5:170382559	0.0497	0.00349	3.30E-45
RAP1A	1:112184499	0.0256	0.00371	5.75E-12
RAP1GAP	1:22023238	0.178	0.00597	1.05E-179
RAP2A	13:98115192	-0.0678	0.0061	2.24E-28
RAPGEF1	9:134565964	0.107	0.003	1.27E-250
RAPGEF3	12:48078806	-0.0354	0.00348	4.40E-24
RAPGEFL1	17:38374131	-0.284	0.0163	1.14E-65
RARB	3:25635743	-0.0193	0.00313	7.74E-10
RARRES1	3:158405899	-0.0448	0.00362	8.54E-35
RARS	5:167950108	-0.101	0.00443	2.41E-110
RARS2	6:88225863	-0.039	0.00301	1.12E-37
RASGRF2	5:80289783	-0.0522	0.005	3.04E-25
RASGRP3	2:33702902	0.141	0.0127	1.75E-28
RASSF2	20:4797284	-0.0377	0.00401	7.86E-21
RASSF3	12:65035405	-0.0621	0.00514	3.57E-33
RASSF4	10:45432352	0.0741	0.00671	4.92E-28
RASSF5	1:206729593	-0.0279	0.00324	9.46E-18
RASSF6	4:74406101	-0.0573	0.00863	3.39E-11
RAVER2	1:65301611	-0.0355	0.00426	1.16E-16
RB1	13:48897520	0.0298	0.00375	2.01E-15
RB1CC1	8:53582514	0.0327	0.00493	3.65E-11
RBAK	7:5154778	-0.0831	0.00839	5.86E-23
RBBP5	1:205050775	-0.029	0.00473	9.65E-10
RBBP8	18:20516135	-0.116	0.00968	1.76E-32
RBCK1	20:392262	-0.0333	0.00508	5.60E-11
RBFOX2	22:36335309	0.0478	0.0047	4.61E-24
RBL2	16:53428284	0.145	0.00338	<1.0E-314
RBM11	21:15613808	-0.0639	0.00625	2.35E-24
RBM12B-AS1	8:94754865	-0.0579	0.00711	5.15E-16

RBM14	11:66401373	0.0735	0.00736	2.57E-23
RBM15	1:110888948	0.11	0.00532	7.08E-92
RBM18	9:125027119	0.128	0.0127	1.36E-23
RBM19	12:114396518	0.0369	0.00401	4.75E-20
RBM23	14:23388231	0.206	0.0145	3.16E-45
RBM26	13:79986065	0.0314	0.00326	8.91E-22
RBM33	7:155513837	0.0438	0.00419	2.52E-25
RBM39	20:34253842	-0.0544	0.00717	4.05E-14
RBM43	2:152118275	0.168	0.00793	7.07E-96
RBM44	2:238681902	-0.0639	0.00482	1.83E-39
RBM47	4:40638708	-0.0332	0.00484	7.93E-12
RBM48	7:92109199	0.0834	0.0137	1.19E-09
RBM5	3:50144951	-0.0242	0.00263	5.17E-20
RBM6	3:50009176	0.0975	0.0038	3.16E-137
RBM8A	1:145507253	0.0828	0.0132	4.35E-10
RBMS1	2:161136656	0.0387	0.00366	8.52E-26
RBMS2	12:56984606	-0.229	0.0147	5.26E-53
RBP5	12:7262024	-0.0862	0.00561	4.20E-52
RBP7	1:10059375	0.112	0.0119	8.80E-21
RBPMS	8:30454132	0.0338	0.00436	1.02E-14
RBPMS2	15:65022906	-0.408	0.00938	<1.0E-314
RC3H2	9:125610975	0.051	0.00744	8.24E-12
RCAN3	1:24809462	-0.0373	0.00544	7.91E-12
RCBTB1	13:50142643	0.167	0.0047	1.05E-248
RCBTB2	13:49049232	0.0788	0.0113	3.82E-12
RCC1	1:28888794	-0.209	0.03	3.45E-12
RCHY1	4:76359426	-0.0489	0.00484	8.74E-24
RCL1	9:4860980	-0.0953	0.00554	1.12E-64
RCN1	11:32112532	0.195	0.0184	3.84E-26
RCN2	15:77241542	0.051	0.00782	7.32E-11
RCOR1	14:103058067	-0.0401	0.0064	4.02E-10
RCSD1	1:167549747	-0.0265	0.00283	1.08E-20
RCVRN	17:9814423	-0.113	0.00534	8.43E-96
RDH13	19:55519940	-0.152	0.00399	1.72E-280
RDM1	17:34251240	0.0434	0.00711	1.08E-09
REC8	14:24641526	-0.0321	0.00402	1.78E-15
RECQL	12:21654407	0.0562	0.00458	4.19E-34
REEP1	2:86453919	-0.0746	0.00463	6.60E-57
REEP3	10:65271488	-0.0681	0.00834	4.07E-16

REEP4	8:21995097	-0.2	0.0102	1.01E-82
REEP5	5:112234957	-0.0691	0.00402	1.94E-64
REL	2:61077103	-0.0245	0.00409	2.14E-09
RELN	7:103485921	-0.00974	0.00161	1.54E-09
RELT	11:73148498	-0.0229	0.00256	4.28E-19
REPS1	6:139290015	-0.154	0.0074	9.56E-93
RERE	1:8490603	0.0448	0.00298	3.55E-50
RETSAT	2:85547429	0.0806	0.00337	3.32E-120
REV1	2:99995804	-0.0349	0.00267	1.69E-38
RFC1	4:39301334	0.0428	0.00338	2.37E-36
RFC4	3:186497254	0.0788	0.00518	3.20E-51
RFFL	17:33424508	-0.0272	0.00359	3.63E-14
RFTN1	3:16362129	-0.053	0.00386	4.12E-42
RFTN2	2:198568076	-0.0772	0.00393	3.05E-83
RFWD3	16:74699293	0.103	0.00365	7.73E-163
RFX7	15:56523595	-0.0388	0.00453	1.41E-17
RFXANK	19:19346396	-0.0814	0.00941	6.53E-18
RGCC	13:42073408	0.0585	0.00933	3.84E-10
RGL1	1:183718530	-0.0388	0.00383	7.35E-24
RGL4	22:24004914	-0.0253	0.00344	2.47E-13
RGP1	9:35766116	0.0638	0.0035	4.72E-72
RGS10	10:121344255	0.185	0.0145	7.54E-37
RGS17	6:153344786	0.0578	0.00873	3.75E-11
RGS18	1:192079104	0.0373	0.00635	4.60E-09
RGS3	9:116209603	0.0223	0.00269	1.39E-16
RGS5	1:163223811	0.0358	0.00516	4.18E-12
RGS6	14:72466285	-0.0747	0.00558	3.30E-40
RHBDD2	7:75500959	0.0444	0.00546	4.61E-16
RHD	1:25687901	-0.709	0.0394	4.35E-70
RHNO1	12:2994134	-0.0411	0.00365	5.76E-29
RHOBTB1	10:62697642	0.0449	0.00449	2.31E-23
RHOBTB3	5:95148129	-0.0829	0.00721	2.91E-30
RHOT1	17:30456747	0.0606	0.00504	7.94E-33
RIC3	11:8133837	0.0585	0.00769	3.42E-14
RIC8A	11:204147	0.0303	0.00319	3.74E-21
RIF1	2:152225877	0.0238	0.00338	2.27E-12
RIMKLB	12:8869396	-0.132	0.0178	1.34E-13
RIN1	11:66091424	0.0537	0.00456	1.13E-31
RIN3	14:92955385	0.0372	0.00247	3.73E-50

RIOK1	6:7435888	-0.0399	0.0038	1.49E-25
RIOK2	5:96514440	0.0633	0.00771	2.77E-16
RIPK1	6:3085129	-0.0724	0.0121	2.64E-09
RIPK2	8:90763759	-0.0263	0.00378	3.51E-12
RIPK3	14:24809957	-0.0324	0.00525	7.14E-10
RIPK4	21:43185345	-0.0315	0.00383	2.49E-16
RIT1	1:155860609	0.0361	0.00486	1.44E-13
RLN1	9:5330464	-0.073	0.00744	1.52E-22
RMI1	9:86626769	-0.0674	0.00518	4.54E-38
RMI2	16:11429939	0.0523	0.00333	2.57E-54
RNASE1	14:21266957	-0.0672	0.00901	1.00E-13
RNASE2	14:21433342	0.27	0.0141	1.29E-78
RNASE3	14:21347911	-0.427	0.0271	9.76E-55
RNASE6	14:21248764	0.126	0.0103	7.76E-34
RNASEH2A	19:12876964	-0.0388	0.00645	2.03E-09
RNASEH2B	13:51472638	0.336	0.0181	3.99E-74
RNASEH2C	11:65477954	0.0363	0.00453	1.41E-15
RNASEK	17:6894691	0.0737	0.00733	1.54E-23
RNASET2	6:167360389	0.275	0.00552	<1.0E-314
RNF111	15:59280119	0.155	0.0117	2.79E-39
RNF114	20:48519702	0.0616	0.0036	6.04E-64
RNF115	1:145691300	0.0369	0.00569	1.05E-10
RNF122	8:33413413	0.113	0.0187	1.57E-09
RNF13	3:149575345	-0.0995	0.00472	8.43E-95
RNF139	8:125459090	0.0588	0.00903	8.00E-11
RNF14	5:141348289	-0.107	0.00867	1.00E-34
RNF141	11:10583600	0.198	0.0223	9.72E-19
RNF144B	6:18412469	-0.107	0.00619	2.12E-65
RNF145	5:158599358	0.0242	0.00384	3.28E-10
RNF149	2:101904406	-0.0422	0.00404	2.54E-25
RNF152	18:59484202	0.0279	0.00454	7.91E-10
RNF166	16:88751207	0.0695	0.00588	6.62E-32
RNF167	17:4824339	-0.155	0.0178	3.81E-18
RNF168	3:196231122	-0.101	0.00436	2.10E-112
RNF17	13:25354456	0.0226	0.00333	1.11E-11
RNF175	4:154691588	0.0599	0.00596	1.72E-23
RNF181	2:85826132	-0.0292	0.00436	2.48E-11
RNF182	6:13889438	1.28	0.0293	<1.0E-314
RNF185	22:31641683	-0.365	0.0274	6.37E-40

RNF19A	8:101370427	-0.0347	0.00462	6.98E-14
RNF19B	1:33440850	-0.0979	0.00829	9.62E-32
RNF2	1:185114834	0.126	0.00929	5.59E-41
RNF20	9:104309445	0.112	0.00549	1.18E-89
RNF212	4:1107478	0.036	0.00425	3.44E-17
RNF216	7:5748370	-0.0604	0.00584	7.38E-25
RNF217	6:125265999	-0.0834	0.00786	5.05E-26
RNF219	13:79183874	0.0904	0.00543	1.09E-60
RNF24	20:4001653	0.0427	0.00431	5.82E-23
RNF32	7:156518195	0.0541	0.00531	3.67E-24
RNF34	12:121859000	-0.0439	0.00539	4.62E-16
RNF38	9:36414837	0.0389	0.00532	2.76E-13
RNF4	4:2470892	0.0387	0.00547	1.70E-12
RNF43	17:56440474	-0.0309	0.00493	4.24E-10
RNF44	5:175968614	-0.0598	0.00957	4.44E-10
RNF6	13:26790174	-0.0351	0.00401	2.62E-18
RNF8	6:37348920	-0.0491	0.00327	4.95E-50
RNFT2	12:117327651	-0.0208	0.00355	4.50E-09
RNGTT	6:89672913	-0.115	0.00757	4.27E-51
RNMT	18:13708328	-0.0953	0.0049	1.29E-81
RNPEP	1:201944214	0.2	0.00391	<1.0E-314
RNPS1	16:2348208	0.0536	0.00739	4.48E-13
ROBO1	3:78726203	0.0161	0.00251	1.55E-10
ROBO3	11:124741772	-0.0121	0.00165	2.14E-13
ROCK2	2:11498147	0.0625	0.0105	2.68E-09
ROGDI	16:4860105	0.104	0.00698	7.69E-49
ROPN1B	3:125731092	0.126	0.00763	7.33E-60
RP9P	7:32961023	-0.114	0.0112	8.49E-24
RPA1	17:1800600	-0.0732	0.00482	4.44E-51
RPA2	1:28211384	0.107	0.00485	2.13E-103
RPAIN	17:5325366	-0.0496	0.00435	1.03E-29
RPAP1	15:41819716	-0.0263	0.00198	1.25E-39
RPAP3	12:48077073	0.172	0.0092	3.02E-75
RPE	2:210913067	0.0484	0.00512	4.65E-21
RPF1	1:84949225	0.0761	0.0102	8.10E-14
RPH3A	12:113276968	0.111	0.00274	<1.0E-314
RPIA	2:89026096	-0.139	0.00909	6.47E-52
RPL13	16:89628870	-0.0989	0.0042	1.43E-116
RPL14	3:40497437	-0.124	0.00628	7.18E-84

RPL15	3:23999756	0.0822	0.0057	3.36E-46
RPL17	18:47005435	-0.193	0.018	1.43E-26
RPL23	17:37001974	-0.0478	0.00735	8.43E-11
RPL27A	11:8719955	-0.0724	0.00687	9.94E-26
RPL28	19:55897327	0.217	0.0131	2.32E-60
RPL31	2:101622728	0.352	0.00842	<1.0E-314
RPL34-AS1	4:109551077	0.0734	0.00439	3.22E-61
RPL36AL	14:50095335	0.0827	0.00472	7.15E-67
RPL37A	2:217349605	0.0429	0.00632	1.36E-11
RPL39L	3:186865979	-0.0845	0.0107	4.07E-15
RPL5	1:93356823	-0.0648	0.00446	5.05E-47
RPL8	8:146012260	0.115	0.00717	1.56E-56
RPN1	3:128399623	-0.052	0.0035	5.84E-49
RPP14	3:58310620	-0.0649	0.00512	2.35E-36
RPP21	6:30263069	0.0498	0.00562	1.08E-18
RPP25L	9:34585385	0.0724	0.00889	4.76E-16
RPRM	2:154338708	-0.044	0.00562	5.90E-15
RPS15A	16:18801583	0.573	0.0192	2.31E-181
RPS16	19:39918729	0.0646	0.00459	3.46E-44
RPS18	6:33239869	0.0614	0.00951	1.20E-10
RPS20	8:56946642	-0.0384	0.00455	4.08E-17
RPS23	5:81610680	0.0602	0.00453	1.20E-39
RPS24	10:79763775	-0.0254	0.00361	2.16E-12
RPS25	11:118857809	0.0966	0.00871	2.84E-28
RPS6	9:19378204	0.0398	0.00631	3.09E-10
RPS6KA1	1:26883511	0.0285	0.00383	1.20E-13
RPS6KA5	14:91576892	-0.188	0.0123	1.78E-51
RPS6KB1	17:58037690	0.0303	0.00485	4.82E-10
RPS6KB2	11:67200812	0.0666	0.00879	4.20E-14
RPTOR	17:78670044	0.0401	0.00337	4.15E-32
RPUSD4	11:126081403	0.034	0.00379	3.87E-19
RQCD1	2:219408054	0.077	0.00369	4.40E-93
RRAGA	9:19049596	0.0293	0.00484	1.54E-09
RRAGD	6:90094844	-0.0715	0.00584	5.30E-34
RREB1	6:7153152	-0.0168	0.00218	1.91E-14
RRH	4:110740865	0.103	0.00717	5.53E-46
RRM1	11:4084113	-0.0305	0.0034	4.33E-19
RRM2B	8:103244557	0.144	0.00811	2.76E-68
RRN3	16:15149240	0.345	0.0409	3.72E-17

RRN3P1	16:21778925	-0.395	0.0154	5.07E-137
RRN3P3	16:22435908	0.176	0.0125	6.26E-44
RRP1	21:45235270	0.0445	0.0034	1.40E-38
RRP12	10:99100432	-0.116	0.00261	<1.0E-314
RRP15	1:218458557	-0.0454	0.00733	6.60E-10
RRP1B	21:45120994	-0.0398	0.0034	2.79E-31
RRP36	6:42986363	0.0635	0.00783	6.34E-16
RSAD1	17:48568230	0.0325	0.00317	2.29E-24
RSF1	11:77360142	0.138	0.017	5.34E-16
RSL1D1	16:11947636	-0.0494	0.00483	2.60E-24
RSPH10B	7:6884357	-0.0502	0.00719	3.34E-12
RSPH3	6:159413299	0.226	0.01	2.44E-107
RSRC1	3:157894615	0.111	0.00656	4.18E-62
RSRC2	12:123006368	-0.0233	0.00353	4.74E-11
RSU1	10:16848473	0.254	0.00478	<1.0E-314
RTF1	15:41693455	0.0435	0.00478	1.11E-19
RTFDC1	20:55101317	0.0363	0.00363	2.41E-23
RTKN2	10:63975930	-0.175	0.00663	5.74E-145
RTN1	14:60239294	-0.0736	0.00485	7.26E-51
RTN3	11:63541285	-0.0965	0.00993	3.83E-22
RTN4	2:55270485	0.0291	0.00247	8.30E-32
RTN4IP1	6:107077502	-0.0499	0.0049	3.84E-24
RTP4	3:187081097	-0.0782	0.00886	1.48E-18
RUFY1	5:179001200	0.137	0.00439	3.26E-195
RUFY2	10:70141482	-0.0464	0.00475	2.39E-22
RUNDC3A	17:42384367	-0.0951	0.00582	1.33E-58
RUNX1-IT1	21:36388176	-0.0812	0.00786	9.58E-25
RUNX1T1	8:93088151	-0.0238	0.00358	3.08E-11
RUNX2	6:45397605	-0.076	0.00953	1.83E-15
RUVBL2	19:49522075	0.042	0.00638	4.89E-11
RWDD2B	21:30376145	-0.176	0.00574	1.91E-190
RWDD3	1:95715508	0.0649	0.0072	2.67E-19
RWDD4	4:184560260	-0.136	0.0132	8.95E-25
RXFP2	13:32380134	-0.0798	0.00366	4.84E-101
RXRA	9:137203271	-0.215	0.0279	1.72E-14
RYBP	3:72504654	-0.0782	0.00444	1.72E-67
RYK	3:133986994	0.0419	0.00496	4.12E-17
S100A10	1:151931004	-0.0646	0.0105	7.43E-10
S100A12	1:153372890	-0.382	0.0124	1.14E-192

S100A13	1:153606294	-0.197	0.00817	1.37E-121
S100A8	1:153372890	-0.0317	0.00536	3.61E-09
S100A9	1:153358685	-0.0889	0.00916	4.51E-22
S100B	21:48039367	-0.49	0.0188	1.32E-141
S100P	4:6692657	-0.697	0.0171	<1.0E-314
S100PBP	1:33245802	0.147	0.00674	1.92E-100
S100Z	5:76144774	-0.115	0.00651	1.39E-67
SAAL1	11:18116894	0.0753	0.00381	8.45E-84
SACM1L	3:45723890	-0.0568	0.00336	2.36E-62
SAMD12	8:119624556	-0.658	0.0147	<1.0E-314
SAMD3	6:130505374	0.154	0.00924	9.83E-61
SAMD9L	7:92820113	0.371	0.0208	6.80E-69
SAMHD1	20:35558914	-0.0545	0.00678	1.12E-15
SAMM50	22:44349236	-0.114	0.00481	1.56E-118
SAP130	2:128785663	0.0465	0.00692	1.88E-11
SAP18	13:21709658	-0.0362	0.00322	5.96E-29
SAR1A	10:71952447	-0.0347	0.00321	4.63E-27
SAR1B	5:133956668	0.0594	0.00798	1.14E-13
SARS	1:109707911	0.0267	0.00349	2.09E-14
SARS2	19:39411716	-0.0281	0.00245	4.72E-30
SART3	12:108922285	0.0273	0.00392	3.30E-12
SASH1	6:148702844	-0.0501	0.00434	2.14E-30
SASS6	1:100528819	-0.115	0.00882	1.97E-38
SATB1	3:18444171	-0.0406	0.00584	4.12E-12
SAV1	14:51182036	0.0357	0.00523	1.05E-11
SAYSD1	6:39069268	-0.0756	0.00431	6.70E-67
SBDS	7:66453476	0.224	0.037	1.53E-09
SBF2	11:10277058	-0.0612	0.00356	2.12E-64
SBNO1	12:123799974	-0.0296	0.00388	2.80E-14
SBNO2	19:1127981	-0.0298	0.00261	6.93E-30
SC5DL	11:121179549	-0.126	0.00969	7.38E-38
SCAF11	12:46322449	0.046	0.00426	7.01E-27
SCAF8	6:155118812	-0.0363	0.00265	9.43E-42
SCAI	9:127803160	-0.0415	0.00492	4.04E-17
SCAMP1	5:77646075	-0.0374	0.00429	3.31E-18
SCAMP2	15:75202205	-0.0427	0.0036	4.60E-32
SCAND2	15:85168758	0.0583	0.00419	2.95E-43
SCAP	3:47409614	0.0234	0.00228	1.70E-24
SCAPER	15:76831236	-0.109	0.00389	3.65E-161

SCARB1	12:125334375	-0.0149	0.00228	6.02E-11
SCCPDH	1:246884464	0.0995	0.00617	4.42E-57
SCD5	4:83764403	-0.067	0.00372	2.90E-70
SCFD1	14:31103012	-0.113	0.00412	1.13E-155
SCFD2	4:54044696	-0.0452	0.00502	2.83E-19
SCGB1A1	11:62193981	-0.048	0.00512	1.09E-20
SCGB3A1	5:179981603	-0.11	0.00632	4.32E-66
SCGB3A2	5:147246760	-0.284	0.00746	1.84E-280
SCIMP	17:5132316	0.165	0.00716	5.54E-112
SCLT1	4:129876588	-0.0492	0.00536	6.37E-20
SCLY	2:238994676	-0.0229	0.00297	1.73E-14
SCMH1	1:41544279	-0.0184	0.00305	1.55E-09
SCML4	6:108047036	-0.0682	0.00524	3.57E-38
SCN8A	12:51966950	-0.0457	0.00383	2.05E-32
SCN9A	2:167237057	0.0827	0.00307	1.97E-149
SCOC	4:141287386	-0.0791	0.0108	2.63E-13
SCP2	1:53343880	-0.165	0.00397	<1.0E-314
SCRG1	4:174323131	-0.0357	0.0045	2.82E-15
SCRN1	7:30063476	0.0804	0.00606	1.35E-39
SCRN2	17:45884026	0.0342	0.00535	1.73E-10
SCRN3	2:175287256	-0.123	0.00492	8.26E-131
SCRT2	20:643919	0.0621	0.0056	3.09E-28
SCYL3	1:169784165	-0.0669	0.00587	9.57E-30
SDCCAG3	9:139311471	0.0722	0.00421	2.88E-64
SDCCAG8	1:243420388	-0.1	0.00395	1.91E-134
SDF4	1:1124750	-0.0953	0.00703	3.32E-41
SDHA	5:215395	0.494	0.0318	2.58E-53
SDK1	7:3718417	-0.0104	0.00164	2.89E-10
SDK2	17:71587577	0.0329	0.00403	3.82E-16
SEC11A	15:85301836	-0.0225	0.00318	1.68E-12
SEC11C	18:56771813	0.0561	0.00811	5.32E-12
SEC13	3:10314602	-0.0516	0.00332	3.70E-53
SEC14L1	17:75170281	0.0605	0.00695	4.52E-18
SEC14L2	22:30794719	-0.0273	0.00329	1.55E-16
SEC14L3	22:30853363	-0.0847	0.00379	1.21E-105
SEC22C	3:42608030	0.062	0.00401	9.65E-53
SEC23A	14:39531992	-0.0319	0.00367	4.27E-18
SEC23B	20:18470664	0.102	0.00406	1.57E-130
SEC23IP	10:121639683	0.0522	0.00586	7.50E-19

SEC31B	10:102278128	0.0764	0.00309	1.04E-127
SEC61A1	3:127740172	0.021	0.00355	3.21E-09
SEC61A2	10:12190321	0.126	0.00448	5.52E-161
SEC62	3:169688367	-0.0504	0.0083	1.34E-09
SECISBP2L	15:49293194	0.0255	0.00412	7.26E-10
SECTM1	17:80317656	0.163	0.0187	3.58E-18
SEH1L	18:13002306	0.0346	0.00457	4.37E-14
SEL1L3	4:25881205	-0.0637	0.00553	2.61E-30
SELK	3:53926017	-0.114	0.00645	5.51E-68
SELL	1:169665632	0.123	0.00581	4.19E-95
SELP	1:169572645	-0.052	0.00784	3.75E-11
SELPLG	12:109013319	0.0767	0.00461	1.25E-60
SELRC1	1:53142832	-0.0303	0.00502	1.75E-09
SELT	3:150341842	-0.108	0.00546	2.05E-83
SEMA3A	7:83803086	-0.0879	0.00655	2.38E-40
SEMA3C	7:80357963	0.0804	0.00758	5.39E-26
SEMA4A	1:156153464	-0.0557	0.00444	1.38E-35
SEMA4B	15:90767860	-0.0525	0.00478	1.05E-27
SEMA4D	9:92077132	-0.0422	0.00395	2.47E-26
SEMA4G	10:102700479	-0.0217	0.00302	7.31E-13
SEMA5A	5:9547958	-0.0611	0.0038	7.69E-57
SEMA6A	5:115740153	0.0339	0.00381	8.24E-19
SEMG1	20:43824279	0.0724	0.0101	7.48E-13
SENP1	12:48489117	0.0613	0.00316	7.43E-81
SENP3	17:7475233	0.0286	0.00358	1.57E-15
SENP6	6:76274825	0.121	0.00356	8.02E-229
SENP7	3:101198604	-0.095	0.00398	6.89E-120
SEPP1	5:42751797	-0.0418	0.00473	1.44E-18
SEPSECS	4:25159390	-0.0644	0.00772	9.49E-17
SEPT10	2:110316285	0.056	0.0083	1.63E-11
SEPT11	4:77915302	-0.059	0.00439	1.72E-40
SEPT2	2:242237945	-0.343	0.00756	<1.0E-314
SEPT8	5:132099999	0.0393	0.00585	2.09E-11
SEPW1	19:48262121	-0.0251	0.00385	7.81E-11
SERAC1	6:158577049	-0.105	0.0041	5.65E-137
SERHL	22:42946371	-0.113	0.00905	3.21E-35
SERHL2	22:43001128	-0.0508	0.0052	2.32E-22
SERINC1	6:122827891	0.0645	0.00553	4.78E-31
SERINC2	1:31901133	0.0354	0.0046	1.72E-14

SERINC3	20:43150309	0.162	0.0124	2.59E-38
SERINC5	5:79419712	0.0654	0.00918	1.25E-12
SERPINA1	14:94854852	-0.102	0.00483	3.79E-94
SERPINB1	6:2856192	-0.0396	0.00485	3.84E-16
SERPINB10	18:61562371	-0.0978	0.00591	5.23E-60
SERPINB2	18:61562690	0.0773	0.00526	6.09E-48
SERPINB6	6:2940666	0.139	0.00472	9.71E-176
SERPINB8	18:61626118	0.101	0.00483	1.83E-93
SERPINB9	6:2857442	0.0734	0.00476	1.26E-52
SERPIND1	22:21118650	-0.0621	0.00909	9.45E-12
SERPINE2	2:224893921	0.123	0.00487	1.29E-132
SERPING1	11:57399511	-0.103	0.011	6.48E-21
SERPINH1	11:75293939	-0.035	0.00403	5.02E-18
SERPINI1	3:167407378	-0.045	0.00749	2.10E-09
SERTAD2	2:64865804	0.0357	0.00497	7.86E-13
SERTAD3	19:40947373	0.126	0.00707	1.37E-68
SESN1	6:109328432	-0.122	0.0065	4.69E-76
SESN3	11:94886632	0.0895	0.00714	1.47E-35
SESTD1	2:180060658	-0.142	0.00612	4.79E-114
SETD4	21:37409835	0.0613	0.00385	9.10E-56
SETD9	5:56237155	0.223	0.00568	4.02E-295
SETDB1	1:150937661	0.0207	0.0032	1.07E-10
SETMAR	3:4403537	-0.121	0.00435	2.09E-159
SETX	9:135210753	-0.0414	0.00607	9.76E-12
SF3A1	22:30742623	-0.0226	0.00336	1.77E-11
SF3B1	2:198255495	-0.0353	0.00307	3.54E-30
SF3B2	11:65785211	0.0442	0.00358	1.10E-34
SFMBT1	3:53083742	-0.0649	0.007	2.93E-20
SFN	1:27221700	0.0247	0.00411	1.95E-09
SFR1	10:105908597	0.113	0.00797	1.44E-44
SFRP2	4:154691808	-0.112	0.0097	1.62E-30
SFT2D1	6:166736518	-0.106	0.0086	2.07E-34
SFXN3	10:102791911	-0.054	0.00642	5.25E-17
SFXN4	10:120907808	0.228	0.00521	<1.0E-314
SGCB	4:52900422	-0.0981	0.00854	3.52E-30
SGCE	7:94166160	-0.0386	0.00362	2.88E-26
SGIP1	1:66999215	0.12	0.00472	5.77E-134
SGK1	6:134585412	-0.126	0.0117	1.62E-26
SGMS1	10:52370312	-0.0235	0.00299	4.69E-15

SGOL1	3:20187080	0.0698	0.00966	5.63E-13
SGPL1	10:72602314	-0.0757	0.00746	5.57E-24
SGSH	17:78184393	-0.0364	0.00296	2.66E-34
SGTB	5:65053753	-0.0465	0.00515	2.30E-19
SH3BGRL2	6:80325857	0.0871	0.00996	2.88E-18
SH3BGRL3	1:26603883	0.145	0.00577	6.29E-131
SH3BP1	22:38029723	-0.0481	0.00359	2.25E-40
SH3BP2	4:2813503	-0.0194	0.00281	5.68E-12
SH3BP5	3:15321396	-0.0355	0.0038	1.31E-20
SH3GLB2	9:131782964	0.0352	0.00404	3.88E-18
SH3PXD2B	5:171878751	0.0976	0.00842	1.01E-30
SH3RF1	4:170217661	-0.0429	0.0032	2.03E-40
SH3YL1	2:264621	0.162	0.00528	1.07E-189
SHARPIN	8:145128896	-0.0721	0.00822	2.50E-18
SHC1	1:154922599	-0.0815	0.00703	9.00E-31
SHISA4	1:201871833	-0.0609	0.00654	1.68E-20
SHKBP1	19:41111069	-0.152	0.00404	4.82E-273
SHMT1	17:18264449	-0.0622	0.0036	3.15E-65
SIAH1	16:48390512	-0.0574	0.00546	1.61E-25
SIAH2	3:150439774	0.18	0.0248	4.55E-13
SIDT1	3:113208224	0.121	0.00623	8.72E-81
SIDT2	11:117002403	0.278	0.0063	<1.0E-314
SIGLEC10	19:51912018	0.13	0.00819	4.74E-55
SIGLEC11	19:50463982	-0.0496	0.00411	4.54E-33
SIGLEC12	19:52004074	-0.0235	0.00354	3.78E-11
SIGLEC5	19:52130830	-0.0821	0.00604	2.27E-41
SIGLEC6	19:52047077	0.0277	0.00367	5.23E-14
SIGLEC7	19:51642784	-0.0307	0.00443	4.75E-12
SIGLEC9	19:51642784	-0.0509	0.00445	5.99E-30
SIGMAR1	9:34638080	0.0423	0.00632	2.35E-11
SIK2	11:111493176	-0.0309	0.00252	5.30E-34
SIMC1	5:175797242	0.0623	0.00601	6.26E-25
SIN3B	19:16913358	0.0586	0.00756	1.12E-14
SIPA1L1	14:72023372	-0.0311	0.00425	2.64E-13
SIRPA	20:1894335	0.143	0.00457	3.34E-197
SIRPB1	20:1546175	-0.264	0.00546	<1.0E-314
SIRPB2	20:1457045	-0.241	0.00641	2.45E-273
SIRPD	20:1539165	-0.06	0.00525	6.06E-30
SIRPG	20:1626479	-0.0377	0.00584	1.18E-10

SIRT1	10:69644217	-0.119	0.00336	1.31E-246
SIRT3	11:218640	-0.0324	0.00332	2.77E-22
SIRT4	12:120785685	-0.0774	0.0127	1.28E-09
SIRT5	6:13580885	0.12	0.00373	6.09E-206
SKA3	13:21747051	0.0531	0.00774	7.52E-12
SKAP1	17:46347508	-0.0439	0.00521	4.96E-17
SKAP2	7:26833021	0.201	0.00469	<1.0E-314
SKIV2L	6:31934372	-0.0464	0.00329	2.25E-44
SKP1	5:133514425	-0.101	0.00864	3.82E-31
SKP2	5:36179044	-0.0515	0.005	1.18E-24
SLA	8:134106641	-0.183	0.0152	5.77E-33
SLAIN1	13:78362028	-0.0471	0.00572	2.36E-16
SLAIN2	4:48429269	0.0542	0.0039	3.15E-43
SLAMF8	1:159801155	0.0421	0.00594	1.48E-12
SLBP	4:1747523	0.0366	0.00448	3.82E-16
SLC11A1	2:219230213	0.228	0.011	2.95E-91
SLC12A1	15:48520590	1.92	0.0276	<1.0E-314
SLC12A2	5:127433047	0.0529	0.00386	5.52E-42
SLC12A4	16:68024995	-0.0227	0.00302	6.74E-14
SLC12A6	15:34580322	0.046	0.00328	8.05E-44
SLC12A7	5:1107428	0.112	0.00656	6.59E-64
SLC12A9	7:100446365	0.0284	0.00412	6.35E-12
SLC13A4	7:135349273	-0.0485	0.00302	8.58E-57
SLC14A1	18:43311483	0.235	0.0213	6.39E-28
SLC15A2	3:121566498	-0.0621	0.00534	6.56E-31
SLC15A4	12:129272242	0.139	0.0114	1.14E-33
SLC16A1	1:113443510	-0.0952	0.00586	5.69E-58
SLC16A3	17:80149901	-0.106	0.00846	3.07E-35
SLC16A4	1:110938530	0.0991	0.0106	1.63E-20
SLC16A6	17:66267650	-0.0905	0.00699	1.08E-37
SLC17A3	6:25883714	0.0359	0.00364	9.72E-23
SLC17A5	6:74278062	-0.0585	0.00644	1.45E-19
SLC18A1	8:20031409	-0.0484	0.00662	3.08E-13
SLC18A2	10:118988797	-0.0567	0.00859	4.45E-11
SLC18B1	6:133119816	0.404	0.0118	3.81E-230
SLC19A2	1:169477574	-0.0634	0.0069	5.70E-20
SLC1A3	5:36702864	0.0395	0.00386	2.43E-24
SLC1A4	2:65218647	-0.0568	0.0052	1.64E-27
SLC1A5	19:47312547	-0.109	0.014	1.16E-14

SLC20A1	2:113457049	0.0984	0.00371	3.12E-145
SLC20A2	8:42425673	-0.0465	0.00294	3.49E-55
SLC22A1	6:160524578	0.102	0.00533	5.46E-79
SLC22A15	1:116518811	-0.151	0.00545	1.98E-157
SLC22A16	6:110797905	-0.111	0.00571	6.29E-82
SLC22A18	11:2929659	0.0267	0.00278	1.23E-21
SLC22A23	6:3354974	-0.0265	0.0034	8.71E-15
SLC22A4	5:131673267	-0.114	0.00517	5.26E-103
SLC22A5	5:131677047	0.0697	0.00304	9.43E-111
SLC23A2	20:4953230	-0.0394	0.00452	3.92E-18
SLC23A3	2:220045035	-0.0386	0.00364	4.38E-26
SLC24A3	20:19281291	0.0433	0.00351	1.42E-34
SLC24A4	14:92955385	0.0633	0.00444	2.82E-45
SLC24A5	15:48462320	-0.226	0.0217	4.20E-25
SLC25A1	22:19180652	-0.1	0.00599	5.59E-61
SLC25A12	2:172694629	-0.0206	0.00322	1.90E-10
SLC25A13	7:95956217	-0.0444	0.004	2.16E-28
SLC25A21	14:37688167	-0.0469	0.00378	7.57E-35
SLC25A24	1:108743059	0.103	0.00426	3.20E-122
SLC25A26	3:66424979	0.0687	0.00407	2.11E-62
SLC25A28	10:101381564	-0.0369	0.00485	3.18E-14
SLC25A29	14:100810426	-0.0562	0.0051	6.01E-28
SLC25A3	12:98987445	-0.0402	0.00515	7.53E-15
SLC25A32	8:104461878	-0.0767	0.00403	3.10E-78
SLC25A37	8:23457200	0.0746	0.00906	2.31E-16
SLC25A44	1:156181615	0.0297	0.00472	3.50E-10
SLC25A46	5:110025473	0.0762	0.00643	5.74E-32
SLC25A51	9:37837140	0.153	0.0195	4.98E-15
SLC26A5	7:103012705	-0.318	0.00775	<1.0E-314
SLC26A6	3:48678011	-0.0323	0.00385	6.69E-17
SLC26A8	6:35961891	-0.0812	0.00609	7.15E-40
SLC27A3	1:153748219	-0.0597	0.00664	3.29E-19
SLC28A2	15:45561868	0.0406	0.00287	1.09E-44
SLC28A3	9:86983368	0.0934	0.00852	1.17E-27
SLC29A3	10:73135743	0.0327	0.00456	8.19E-13
SLC2A1	1:43440783	0.0433	0.00495	2.53E-18
SLC30A4	15:45730926	0.0319	0.00508	3.56E-10
SLC30A5	5:68419908	0.018	0.00287	3.71E-10
SLC30A6	2:32391231	0.0265	0.00422	3.87E-10

SLC30A9	4:41992752	0.315	0.0182	1.07E-65
SLC31A1	9:116025982	0.112	0.00753	2.69E-49
SLC31A2	9:115917783	-0.0699	0.00572	7.36E-34
SLC33A1	3:155535765	0.0517	0.00385	1.65E-40
SLC35A1	6:88190108	-0.0378	0.00393	8.91E-22
SLC35A5	3:112242208	0.0648	0.00846	2.25E-14
SLC35B2	6:44205845	-0.0337	0.00421	1.42E-15
SLC35B3	6:8435883	0.0281	0.00374	6.35E-14
SLC35C1	11:45834201	0.0316	0.00529	2.52E-09
SLC35D1	1:67529243	0.0955	0.00608	1.87E-54
SLC35D2	9:99173448	0.0436	0.00544	1.24E-15
SLC35E1	19:16699813	0.0502	0.00405	9.37E-35
SLC35E3	12:69184350	0.0677	0.00425	8.02E-56
SLC35F3	1:234451159	-0.0302	0.00274	5.39E-28
SLC35F5	2:114541449	0.0237	0.00351	1.55E-11
SLC36A1	5:150883158	-0.132	0.00507	1.74E-141
SLC37A2	11:124958397	-0.0362	0.00398	1.26E-19
SLC37A3	7:140098605	0.112	0.00689	6.79E-58
SLC38A10	17:79245770	-0.0278	0.0031	4.46E-19
SLC38A11	2:165834674	-0.0719	0.0114	3.05E-10
SLC38A4	12:47139555	-0.0508	0.00505	1.23E-23
SLC38A6	14:61514673	-0.0494	0.00668	1.78E-13
SLC39A1	1:153895936	-0.0448	0.00468	1.50E-21
SLC39A11	17:71027543	0.0653	0.00625	2.78E-25
SLC39A6	18:33709217	-0.0237	0.00353	2.24E-11
SLC39A8	4:103225513	-0.0574	0.0051	4.94E-29
SLC39A9	14:69934611	-0.167	0.00812	6.32E-91
SLC40A1	2:190441317	-0.0753	0.00604	3.70E-35
SLC41A1	1:205799987	-0.0171	0.00255	2.11E-11
SLC41A2	12:105353244	0.0592	0.00681	4.68E-18
SLC41A3	3:125794616	0.0446	0.00288	5.78E-53
SLC44A2	19:10703339	0.113	0.00532	1.28E-95
SLC44A4	6:31836827	-0.247	0.00463	<1.0E-314
SLC44A5	1:75911113	-0.168	0.00451	1.40E-270
SLC45A4	8:142277620	0.136	0.0135	1.17E-23
SLC46A2	9:115673533	0.0503	0.00398	3.84E-36
SLC47A1	17:19525492	0.0333	0.00314	5.68E-26
SLC48A1	12:48169526	-0.0815	0.00434	3.76E-76
SLC4A1	17:42313512	-0.0912	0.013	2.91E-12

SLC4A7	3:27493730	0.0832	0.0119	3.51E-12
SLC4A8	12:51847800	-0.0331	0.00376	1.79E-18
SLC5A11	16:24835168	-0.0398	0.0033	4.12E-33
SLC5A3	21:35401161	0.0617	0.00781	3.52E-15
SLC5A4	22:32599716	0.0359	0.00379	4.02E-21
SLC5A6	2:27458790	0.0546	0.00405	6.96E-41
SLC6A12	12:329131	-0.0244	0.00385	2.44E-10
SLC6A16	19:49867913	0.0406	0.00511	2.26E-15
SLC6A6	3:14520560	-0.1	0.00539	4.45E-75
SLC7A1	13:30202377	-0.067	0.00614	2.30E-27
SLC7A11	4:139147725	-0.147	0.0144	2.97E-24
SLC7A6	16:68317061	0.096	0.00432	1.34E-104
SLC7A6OS	16:68317061	0.0324	0.00326	4.47E-23
SLC7A7	14:23285439	0.0787	0.00584	1.04E-40
SLC7A8	14:23631372	-0.323	0.0116	1.73E-160
SLC8A1	2:40687437	0.0955	0.00572	4.87E-61
SLC9A2	2:103347094	-0.0492	0.00345	2.22E-45
SLC9A3R1	17:72767435	-0.0366	0.00278	4.83E-39
SLC9B1	4:103832044	-0.0505	0.00516	1.98E-22
SLC9B2	4:104010619	0.0291	0.00387	6.53E-14
SLCO3A1	15:92399657	0.0415	0.0035	5.25E-32
SLCO4C1	5:101604320	-0.101	0.00591	3.15E-63
SLFN11	17:33727598	-0.0812	0.0098	1.47E-16
SLFN12	17:33753808	-0.241	0.00925	1.58E-140
SLFN13	17:33801032	-0.0602	0.00582	8.09E-25
SLFN5	17:33563302	-0.149	0.00483	1.76E-193
SLIRP	14:78207178	0.172	0.015	3.93E-30
SLIT1	10:98993813	-0.0144	0.00165	3.00E-18
SLK	10:105723183	-0.0327	0.00463	1.76E-12
SLMO2	20:57593158	0.0367	0.00525	3.17E-12
SLPI	20:43888106	0.114	0.0163	3.36E-12
SMAD1	4:146392431	0.0305	0.00376	5.31E-16
SMAD4	18:48553225	-0.0269	0.00257	2.18E-25
SMAD5	5:135516203	-0.115	0.0049	3.37E-115
SMAP1	6:71375961	-0.059	0.00282	8.90E-94
SMAP2	1:40838636	-0.0293	0.00466	3.62E-10
SMARCA4	19:11054715	0.0261	0.00385	1.48E-11
SMARCAD1	4:95208714	0.0713	0.00378	4.42E-77
SMARCAL1	2:217275483	-0.0294	0.00331	8.82E-19

SMARCB1	22:24180449	0.0612	0.00661	2.85E-20
SMARCD2	17:61887693	0.0272	0.00266	2.57E-24
SMARCE1	17:38782343	0.0986	0.0042	3.43E-116
SMC1B	22:45818758	-0.0641	0.00318	5.65E-87
SMC2	9:106849347	0.0497	0.00554	3.82E-19
SMC4	3:160161111	-0.0386	0.0049	3.97E-15
SMC6	2:17981258	0.0439	0.00534	2.28E-16
SMCR8	17:18205750	-0.049	0.00455	8.54E-27
SMG1	16:18936355	0.0727	0.00464	3.26E-54
SMG5	1:156208230	0.122	0.00251	<1.0E-314
SMG7	1:183570545	-0.0207	0.00303	9.22E-12
SMG8	17:57339990	-0.041	0.00653	3.72E-10
SMIM11	21:35741702	-0.0995	0.0159	3.86E-10
SMIM7	19:16807428	0.0812	0.0114	1.16E-12
SMIM8	6:88043372	0.16	0.0058	9.82E-156
SMNDC1	10:112053599	0.0531	0.0035	7.14E-51
SMOX	20:4157072	0.116	0.0068	7.38E-64
SMPD2	6:109736614	-0.0402	0.00361	1.46E-28
SMPDL3A	6:123065057	-0.142	0.0115	1.28E-34
SMUG1	12:54573816	0.039	0.00633	7.41E-10
SMURF1	7:98678486	-0.055	0.00868	2.62E-10
SMURF2	17:62569580	-0.0729	0.01	4.37E-13
SMYD2	1:214497080	0.0861	0.00384	1.10E-106
SMYD3	1:246670297	-0.0296	0.00444	2.86E-11
SMYD4	17:1696244	0.0403	0.00341	7.42E-32
SNAP29	22:21293653	0.0976	0.00879	2.41E-28
SNAP91	6:84320225	-0.816	0.0512	8.63E-56
SNAPC1	14:62275354	-0.14	0.00512	1.95E-154
SNAPC3	9:15434792	0.0735	0.00563	2.27E-38
SNAPIN	1:153635425	-0.0337	0.00496	1.28E-11
SNCA	4:90757840	-0.0865	0.00759	9.55E-30
SND1-IT1	7:127643968	-0.127	0.0152	9.90E-17
SNF8	17:47006493	0.0264	0.00422	4.61E-10
SNIP1	1:38047385	0.0382	0.00471	6.43E-16
SNRNP25	16:145884	0.127	0.0159	1.83E-15
SNRNP27	2:70141519	-0.0459	0.00451	3.69E-24
SNRNP40	1:31761703	0.0644	0.00875	2.21E-13
SNRNP48	6:7576368	-0.0406	0.00417	3.03E-22
SNRPB	20:2457887	-0.0435	0.00481	2.36E-19

SNRPB2	20:16710791	0.0832	0.014	2.80E-09
SNRPC	6:34748893	0.0524	0.00623	5.66E-17
SNRPD3	22:24935250	-0.0932	0.00397	3.16E-116
SNRPN	15:25217611	0.112	0.00388	5.64E-170
SNTB2	16:69194699	-0.0395	0.00431	7.81E-20
SNUPN	15:75863915	-0.0529	0.00891	2.96E-09
SNW1	14:78202777	-0.0839	0.00868	6.10E-22
SNX1	15:64441179	-0.0433	0.00437	6.65E-23
SNX11	17:46178674	0.0689	0.00374	2.77E-73
SNX16	8:82720700	-0.187	0.00492	9.53E-280
SNX18	5:53815722	-0.122	0.00435	3.84E-160
SNX19	11:130749351	-0.0924	0.00294	2.68E-199
SNX2	5:122209192	-0.0289	0.00393	2.11E-13
SNX20	16:50703698	0.136	0.0192	1.51E-12
SNX25	4:186172793	-0.112	0.00463	1.20E-121
SNX32	11:65623739	-0.0504	0.00316	3.41E-56
SNX4	3:125197161	-0.0831	0.00612	2.66E-41
SNX6	14:35105227	-0.156	0.00482	2.28E-209
SNX7	1:99127205	-0.151	0.0126	1.09E-32
SOAT1	1:179312235	0.0762	0.00387	3.06E-83
SOCS5	2:46947440	0.089	0.006	1.00E-48
SOD2	6:160087409	0.115	0.00518	3.42E-105
SOGA3	6:127889330	-0.0276	0.00406	1.11E-11
SOHLH2	13:36830298	-0.058	0.00562	1.06E-24
SORCS3	10:106740404	-0.0671	0.00735	9.04E-20
SORT1	1:109851126	0.034	0.00519	6.77E-11
SOS2	14:50712797	-0.0738	0.00636	1.06E-30
SP110	2:231042276	0.0361	0.00471	2.17E-14
SP140	2:231091223	0.0565	0.00731	1.26E-14
SP140L	2:231265405	0.0748	0.00456	3.87E-59
SP2	17:45974285	-0.0196	0.00313	3.96E-10
SPAG1	8:101214189	-0.0948	0.00315	4.02E-184
SPAG7	17:4865928	-0.117	0.00632	3.95E-74
SPAG8	9:35808334	-0.0659	0.0103	1.65E-10
SPAG9	17:49054439	-0.026	0.00429	1.49E-09
SPATA13	13:24877550	0.0649	0.00366	2.62E-68
SPATA20	17:48616114	-0.153	0.00376	<1.0E-314
SPATA32	17:43335413	0.0359	0.00493	3.78E-13
SPATA5	4:124024947	-0.0282	0.0043	6.40E-11

SPATA5L1	15:45644685	-0.181	0.00448	<1.0E-314
SPATA6	1:48739068	0.0798	0.00931	1.36E-17
SPATA6L	9:4661019	0.0616	0.00367	2.18E-61
SPATA7	14:88950847	0.157	0.00429	1.58E-260
SPATA9	5:94965599	-0.0714	0.00393	1.60E-71
SPATS2L	2:201163239	-0.0487	0.00735	3.79E-11
SPCS3	4:177256937	-0.0744	0.00577	1.44E-37
SPDL1	5:168970932	0.206	0.0108	1.08E-78
SPECC1	17:19980772	0.0626	0.00645	4.48E-22
SPECC1L	22:24844883	-0.112	0.00869	1.80E-37
SPEF2	5:35656945	-0.023	0.00301	2.69E-14
SPG20	13:36886170	-0.158	0.00437	2.20E-256
SPHK1	17:74378242	-0.0457	0.0078	4.96E-09
SPICE1	3:113190164	0.083	0.00823	9.55E-24
SPINT2	19:38754948	0.0323	0.00428	5.08E-14
SPNS3	17:4386669	0.0285	0.00262	2.51E-27
SPOCK2	10:73823298	-0.0385	0.00318	2.60E-33
SPON1	11:14309434	0.0203	0.00297	8.86E-12
SPOP	17:47718188	0.0216	0.00318	1.36E-11
SPP1	4:88897106	0.181	0.0065	3.99E-159
SPPL3	12:121215911	-0.0249	0.00337	1.66E-13
SPRED2	2:65684152	0.0186	0.00314	3.46E-09
SPRTN	1:231484361	-0.0738	0.00397	8.50E-75
SPSB1	1:9347552	-0.0252	0.00318	2.33E-15
SPSB2	12:7017043	-0.052	0.0053	1.74E-22
SPSB3	16:1827836	-0.037	0.00449	2.15E-16
SPTBN1	2:54787592	-0.17	0.00883	1.13E-79
SPTLC2	14:78023519	-0.0784	0.00419	8.09E-76
SPTLC3	20:12988132	0.0347	0.00571	1.28E-09
SQLE	8:126031754	-0.0521	0.00763	9.51E-12
SQRDL	15:45922030	-0.0445	0.0055	6.71E-16
SQSTM1	5:179254340	-0.0407	0.00263	7.67E-53
SRA1	5:139937146	0.0893	0.00373	1.81E-120
SRBD1	2:45817382	-0.0276	0.00448	7.87E-10
SRD5A1	5:6634631	-0.0321	0.00543	3.53E-09
SRD5A3	4:56217015	-0.221	0.00684	5.61E-210
SREBF1	17:17750558	-0.0192	0.00169	1.89E-29
SRGAP3	3:9290344	-0.0229	0.0038	1.97E-09
SRGN	10:70805373	-0.058	0.00568	3.07E-24

SRI	7:87835469	-0.0339	0.0052	7.33E-11
SRP54	14:35402379	-0.0429	0.00319	1.31E-40
SRP68	17:74060687	-0.0297	0.00475	4.47E-10
SRP72	4:57357244	0.134	0.0053	3.10E-133
SRPK1	6:35834129	-0.0993	0.0058	4.15E-64
SRPK2	7:105017276	0.0555	0.00628	1.39E-18
SRPRB	3:133524717	-0.0449	0.00638	2.10E-12
SRR	17:2203175	0.0612	0.00451	2.67E-41
SRRT	7:100486035	-0.023	0.00343	2.37E-11
SRSF12	6:89788797	0.0427	0.00405	9.94E-26
SRSF3	6:36558964	-0.0474	0.00489	4.96E-22
SRSF4	1:29434780	-0.0325	0.00463	2.40E-12
SRSF9	12:120901765	0.706	0.0264	7.42E-148
SRXN1	20:633149	0.106	0.00485	7.13E-102
SSB	2:170661333	0.0764	0.00903	3.22E-17
SSBP2	5:80747220	0.0352	0.00385	7.83E-20
SSH1	12:109260526	-0.0369	0.00359	1.50E-24
SSH2	17:28178724	-0.0852	0.00388	2.96E-102
SSH3	11:67021930	0.0957	0.0106	2.74E-19
SSR1	6:7278046	0.0295	0.00334	1.27E-18
SSR3	3:156262753	0.0666	0.00696	1.69E-21
SSU72	1:1505215	-0.274	0.0192	3.80E-45
SSX2IP	1:85133731	0.126	0.00828	2.86E-51
ST14	11:130019574	0.0686	0.00583	1.65E-31
ST3GAL1	8:134552618	0.0683	0.00538	2.19E-36
ST3GAL4	11:126181592	-0.0296	0.00418	1.57E-12
ST3GAL5	2:86094686	0.0805	0.00527	1.95E-51
ST3GAL6	3:98492380	-0.0305	0.00504	1.59E-09
ST6GAL1	3:186739677	0.0335	0.00535	4.51E-10
ST6GALNAC1	17:74674556	-0.045	0.00405	2.08E-28
ST6GALNAC2	17:74595847	0.238	0.0107	5.60E-104
ST6GALNAC3	1:76518693	0.0636	0.00434	1.14E-47
ST6GALNAC6	9:130668957	0.122	0.0102	8.99E-33
ST7L	1:113089006	0.0371	0.00313	5.06E-32
ST8SIA4	5:100258523	0.129	0.0112	4.94E-30
STAB1	3:52528292	0.0733	0.00523	8.14E-44
STAG3L4	7:66767623	-0.103	0.00501	9.33E-91
STAM	10:17748365	0.0604	0.00537	5.15E-29
STAM2	2:153024245	0.037	0.0032	1.41E-30

STAMBP	2:74057096	-0.111	0.0132	4.68E-17
STAMBPL1	10:90683124	0.0414	0.00465	6.50E-19
STARD10	11:72506324	-0.0566	0.00322	2.58E-67
STARD3NL	7:38203783	0.0838	0.00537	1.26E-53
STARD4	5:110848319	0.115	0.0076	7.58E-51
STARD5	15:81607503	-0.05	0.00562	7.59E-19
STARD7	2:96872400	0.0629	0.0046	7.05E-42
STAT3	17:40450170	0.0305	0.00483	2.89E-10
STAT5A	17:40456010	0.0385	0.00373	1.11E-24
STAT6	12:57489648	0.039	0.00402	4.56E-22
STEAP3	2:120016461	0.022	0.00262	5.02E-17
STEAP4	7:87859018	0.328	0.00797	<1.0E-314
STIM1	11:4107584	0.0356	0.00289	2.93E-34
STIM2	4:26884408	0.0396	0.00295	2.22E-40
STK10	5:171533782	0.0945	0.00344	2.20E-155
STK17B	2:197084587	-0.0287	0.00359	1.63E-15
STK24	13:99236283	0.0212	0.00301	2.03E-12
STK32C	10:134073053	-0.053	0.00439	3.92E-33
STK33	11:8524933	-0.0662	0.0043	2.76E-52
STK35	20:2127609	-0.0369	0.00373	6.38E-23
STK38L	12:27363643	-0.0471	0.008	4.01E-09
STK39	2:169041386	0.0671	0.00442	5.37E-51
STK4	20:43713593	-0.0205	0.00266	1.64E-14
STOM	9:124118776	-0.077	0.0119	1.04E-10
STON1-GTF2A1L	2:48790832	-0.029	0.00308	7.38E-21
STOX1	10:70599126	-0.149	0.0098	2.48E-51
STPG1	1:24765537	-0.0213	0.00363	4.75E-09
STPG2	4:98959282	0.0398	0.00354	5.28E-29
STRADA	17:61817048	-0.0659	0.00324	2.87E-88
STRN	2:37167191	-0.0265	0.00343	1.22E-14
STRN3	14:31394171	0.0566	0.00441	3.85E-37
STT3A	11:125513097	0.0602	0.00385	5.06E-54
STT3B	3:31524844	0.0496	0.00459	7.13E-27
STX16	20:57249771	-0.0439	0.00625	2.35E-12
STX17	9:102721679	-0.041	0.00486	4.05E-17
STX1A	7:73114440	0.0196	0.0028	3.01E-12
STX2	12:131319810	0.123	0.00626	1.35E-82
STX4	16:31054607	-0.0548	0.00343	3.99E-56
STX5	11:62640727	0.111	0.00875	2.17E-36

STX6	1:180981800	0.0376	0.00617	1.19E-09
STX7	6:132834518	0.0429	0.00438	2.09E-22
STX8	17:9334409	-0.0732	0.00633	1.54E-30
STXBP1	9:130371107	0.0242	0.0035	5.00E-12
STXBP3	1:109323270	0.0593	0.00462	3.04E-37
STXBP4	17:53045243	-0.163	0.00559	7.96E-173
STXBP5	6:147640599	-0.0245	0.00386	2.29E-10
STYX	14:53238046	0.162	0.0243	2.73E-11
STYXL1	7:75657553	-0.262	0.00654	1.08E-307
SUCLA2	13:48519113	-0.0489	0.00622	4.66E-15
SUCLG2	3:67421182	-0.0995	0.00784	2.11E-36
SUCO	1:172453840	-0.0999	0.00437	2.43E-110
SUDS3	12:118854407	0.0608	0.00581	2.15E-25
SUFU	10:104382673	-0.0166	0.00237	2.54E-12
SUGP1	19:19350103	0.0301	0.00397	4.24E-14
SUGT1	13:53255012	0.0366	0.00513	1.09E-12
SUGT1P1	9:33529314	-0.132	0.0226	4.50E-09
SUGT1P3	13:41484599	0.181	0.0274	4.31E-11
SULF2	20:46356316	0.201	0.0103	1.35E-81
SULT1A1	16:28614472	-0.12	0.0106	2.37E-29
SULT1B1	4:70586672	0.0549	0.00667	2.32E-16
SULT1E1	4:70675023	-0.0229	0.003	2.44E-14
SUMF1	3:4515991	0.0477	0.00282	1.23E-62
SUMO1P1	20:52498570	-0.173	0.0128	1.37E-40
SUMO3	21:46244045	0.04	0.00628	2.20E-10
SUMO4	6:149686518	-0.0709	0.00643	5.63E-28
SUN2	22:39147235	-0.0329	0.00461	1.04E-12
SUOX	12:56401085	0.0694	0.00433	2.04E-56
SUPT20H	13:37595173	0.201	0.00482	<1.0E-314
SUPT3H	6:45275674	0.104	0.00534	5.17E-81
SUPT4H1	17:56423114	0.0629	0.00317	2.42E-84
SUPV3L1	10:70975053	-0.0479	0.00475	1.05E-23
SUSD1	9:114937234	0.0922	0.00413	2.93E-105
SUSD3	9:95784485	-0.0187	0.00312	1.98E-09
SUV39H2	10:14951811	0.032	0.0048	3.11E-11
SUZ12P1	17:29109927	0.0816	0.00996	3.27E-16
SV2A	1:149891323	-0.16	0.0205	7.27E-15
SVIL	10:30022551	-0.039	0.00359	3.34E-27
SWT1	1:185194409	-0.0855	0.00716	1.78E-32

SYCP2	20:58552237	-0.204	0.0173	1.62E-31
SYCP2L	6:10850173	-0.358	0.00868	<1.0E-314
SYCP3	12:102090080	-0.107	0.00447	1.26E-120
SYK	9:93559351	0.0709	0.00395	6.10E-70
SYN3	22:32872258	-0.0138	0.00236	5.07E-09
SYNE1	6:152875862	-0.0548	0.00871	3.48E-10
SYNE2	14:64278769	0.101	0.0141	7.18E-13
SYNE3	14:95981610	0.026	0.003	6.69E-18
SYNGR1	22:39751251	-0.0305	0.00348	2.76E-18
SYNGR2	17:76186187	0.0965	0.0101	2.16E-21
SYNJ2	6:158501843	-0.0528	0.00416	2.78E-36
SYNJ2BP	14:70834418	0.0844	0.0124	1.31E-11
SYNM	15:99647605	0.112	0.00338	7.44E-220
SYS1	20:44012451	0.0359	0.00415	6.81E-18
SYT9	11:7526600	-0.0277	0.00277	1.78E-23
SYTL2	11:85499983	0.21	0.013	1.47E-57
SZRD1	1:16664373	0.0299	0.00438	9.30E-12
TAB2	6:149611269	0.0252	0.00409	8.55E-10
TAC3	12:57404858	-0.086	0.0141	9.99E-10
TACC1	8:38742856	0.0273	0.00334	3.62E-16
TACC3	4:1748216	0.0944	0.00431	1.23E-101
TACSTD2	1:58991470	-0.213	0.00503	<1.0E-314
TADA2A	17:35807445	-0.0861	0.0046	1.48E-75
TADA3	3:9819922	-0.0476	0.00351	3.34E-41
TAF10	11:6632450	0.0678	0.00415	2.02E-58
TAF11	6:34904488	-0.0478	0.00496	9.43E-22
TAF13	1:109598452	-0.0953	0.0109	3.42E-18
TAF1B	2:10048233	0.144	0.00499	1.22E-169
TAF1C	16:84209184	0.0198	0.00283	3.06E-12
TAF1D	11:93422613	-0.0465	0.00401	1.37E-30
TAF2	8:120821264	-0.0783	0.0032	4.30E-125
TAF7	5:140685921	0.125	0.013	1.13E-21
TAF8	6:42052817	0.0491	0.00723	1.17E-11
TAF9	5:68638941	0.0572	0.00731	5.80E-15
TAGAP	6:159463452	0.0904	0.00795	1.31E-29
TAGLN	11:117070547	0.108	0.00376	3.41E-169
TAGLN2	1:159892088	0.0588	0.00709	1.48E-16
TAL1	1:47706970	-0.0436	0.00364	1.16E-32
TAMM41	3:11828825	-0.265	0.00659	<1.0E-314

TANGO2	22:20066611	0.0355	0.00447	2.33E-15
TANK	2:162025322	0.179	0.00468	1.37E-283
TAOK3	12:118749798	-0.0307	0.00387	2.76E-15
TAP2	6:32766496	0.0887	0.0134	4.14E-11
TAPBP	6:33283766	0.0277	0.00289	1.22E-21
TAPBPL	12:6570821	-0.0507	0.00296	2.88E-64
TAPT1	4:16155058	-0.0386	0.00383	1.36E-23
TARBP1	1:234610539	0.0403	0.00322	2.45E-35
TARDBP	1:11100898	-0.0447	0.00438	3.50E-24
TARP	7:38358102	-0.276	0.0126	5.82E-102
TARSL2	15:102236087	-0.0288	0.00313	3.86E-20
TAS2R10	12:11028507	-0.088	0.00916	1.20E-21
TAS2R13	12:11093703	0.202	0.0123	7.91E-59
TAS2R14	12:11051749	-0.236	0.0082	5.60E-170
TAS2R19	12:11170074	-0.213	0.0132	7.03E-57
TAS2R20	12:11131212	0.426	0.0105	<1.0E-314
TAS2R3	7:141489866	-0.0624	0.00788	3.14E-15
TAS2R31	12:11166536	0.302	0.0124	3.71E-124
TAS2R4	7:141446818	-0.16	0.0126	1.25E-36
TAS2R41	7:143126796	-0.177	0.0101	1.26E-66
TAS2R46	12:11216855	-0.608	0.0221	1.37E-155
TAS2R5	7:141463914	-0.104	0.0075	2.69E-43
TAS2R50	12:11138683	-0.477	0.0132	1.78E-256
TAS2R60	7:143111112	-0.356	0.00888	1.45E-306
TATDN2	3:10320865	-0.0467	0.00353	1.89E-39
TBC1D1	4:37966182	0.0361	0.00332	3.92E-27
TBC1D14	4:6919674	0.0668	0.00399	2.58E-61
TBC1D15	12:72322443	0.0682	0.00672	5.29E-24
TBC1D17	19:50370846	-0.0211	0.00311	1.38E-11
TBC1D2	9:100985826	-0.0241	0.00375	1.42E-10
TBC1D20	20:416149	-0.028	0.00425	4.45E-11
TBC1D22A	22:47399473	-0.0211	0.00304	4.00E-12
TBC1D2B	15:78367474	-0.0435	0.00487	5.81E-19
TBC1D4	13:75884290	-0.0473	0.00332	2.75E-45
TBC1D5	3:17729450	-0.0305	0.00429	1.31E-12
TBC1D7	6:13327777	-0.139	0.005	2.13E-159
TBC1D8	2:101777393	-0.0665	0.00621	1.61E-26
TBC1D9B	5:179335060	0.0525	0.00567	3.09E-20
TBCB	19:36605875	-0.153	0.0242	2.40E-10

TBCD	17:80822769	0.0227	0.00264	1.03E-17
TBCE	1:235600129	-0.039	0.00406	1.08E-21
TBCK	4:107251278	0.0903	0.00416	4.50E-100
TBK1	12:64845715	0.0592	0.00748	2.81E-15
TBKBP1	17:45791123	-0.0353	0.00217	5.01E-58
TBP	6:170821226	-0.0483	0.00726	3.21E-11
TBPL1	6:134268822	0.11	0.00564	1.55E-82
TBRG4	7:45146584	-0.0324	0.00272	2.35E-32
TC2N	14:92332548	-0.0894	0.00739	3.34E-33
TCAIM	3:44408255	-0.0493	0.00607	6.33E-16
TCEA3	1:23751140	0.0841	0.00602	1.51E-43
TCEB3	1:24051614	0.0778	0.00549	1.05E-44
TCERG1	5:145855288	0.0403	0.00407	5.28E-23
TCF19	6:31123359	-0.12	0.00555	5.79E-99
TCF25	16:89922485	-0.0927	0.00569	2.85E-58
TCF7L1	2:85546615	0.0288	0.00208	5.52E-43
TCF7L2	10:114711755	-0.0869	0.0065	3.79E-40
TCFL5	20:61452263	0.122	0.00922	1.61E-39
TCIRG1	11:67804156	0.0456	0.00522	2.93E-18
TCL1A	14:96162418	0.276	0.0406	1.17E-11
TCL1B	14:96104174	-0.0398	0.00638	4.87E-10
TCN1	11:59570967	-0.208	0.0258	9.84E-16
TCN2	22:31004127	-0.0223	0.00318	2.26E-12
TCP11L1	11:33052654	-0.211	0.00522	<1.0E-314
TCTEX1D2	3:196066227	-0.0649	0.00476	1.21E-41
TCTN2	12:124167006	-0.0682	0.00413	1.06E-59
TCTN3	10:97374883	-0.0804	0.00799	1.26E-23
TDP1	14:90422664	-0.0329	0.00381	7.90E-18
TDP2	6:24650671	-0.057	0.00648	1.81E-18
TDRD6	6:46618415	0.0174	0.00291	2.43E-09
TDRD7	9:100197911	-0.0408	0.0053	1.73E-14
TDRD9	14:104428576	-0.219	0.00744	1.85E-176
TDRKH	1:151742105	0.0691	0.00397	3.41E-66
TEAD1	11:13010440	-0.139	0.00759	1.32E-72
TEC	4:48112291	-0.0675	0.00652	7.54E-25
TECPR1	7:97896175	0.0203	0.00189	1.20E-26
TEDDM1	1:182362259	0.0934	0.00838	1.53E-28
TEF	22:41777315	-0.031	0.00341	1.42E-19
TEK	9:27193847	-0.0931	0.00339	1.60E-155

TEN1	17:74015595	0.0318	0.00382	1.04E-16
TEP1	14:20881527	0.0398	0.00299	8.91E-40
TES	7:115851177	0.453	0.0212	7.33E-97
TESC	12:117492691	-0.133	0.014	2.78E-21
TESK2	1:45954599	0.0595	0.00411	1.16E-46
TESPA1	12:55395712	0.108	0.00905	1.58E-32
TEX10	9:103108941	0.0609	0.00494	1.94E-34
TEX101	19:43870954	-0.0359	0.00566	2.23E-10
TEX9	15:56746569	-0.113	0.0131	8.84E-18
TFAM	10:60144073	-0.133	0.00397	1.57E-222
TFB1M	6:155625104	-0.112	0.00449	4.51E-130
TFB2M	1:246707709	-0.0501	0.00524	1.84E-21
TFDP2	3:141742127	-0.13	0.0111	5.15E-31
TFEC	7:115665688	0.0556	0.00713	7.10E-15
TFG	3:100439823	-0.137	0.00358	9.77E-284
TFIP11	22:26904541	0.178	0.00424	<1.0E-314
TFPT	19:54625765	0.0676	0.00506	5.28E-40
TFRC	3:195835404	-0.292	0.0317	3.99E-20
TG	8:133877623	0.0423	0.00188	1.08E-106
TGFB2	1:218660024	-0.0567	0.00583	3.77E-22
TGFBI	5:135335229	0.0978	0.00644	5.65E-51
TGFBR1	9:101911125	0.0562	0.00466	4.57E-33
TGFBR2	3:30690393	-0.0788	0.00492	2.74E-56
TGFBRAP1	2:105897519	-0.0408	0.00622	5.83E-11
TGM3	20:2281284	0.0557	0.00417	5.68E-40
TGOLN2	2:85542686	0.0583	0.00347	1.34E-61
TGS1	8:56685933	0.057	0.00534	2.50E-26
TH1L	20:57564695	0.108	0.00761	1.09E-44
THADA	2:43732347	0.019	0.00253	7.58E-14
THAP7-AS1	22:21366206	0.0836	0.0082	3.62E-24
THBD	20:23078996	0.196	0.00721	8.73E-153
THBS3	1:155184975	0.1	0.00412	7.03E-124
THEM4	1:151865858	0.1	0.0138	4.87E-13
THEMIS2	1:28209362	0.198	0.00501	1.29E-299
THG1L	5:157160630	-0.0783	0.0111	1.52E-12
THNSL1	10:25332461	-0.0357	0.00564	2.61E-10
THNSL2	2:88453718	-0.102	0.00435	5.27E-116
THOC5	22:29933627	0.0265	0.00395	2.13E-11
THOC7	3:63842629	-0.0818	0.00654	2.20E-35

THRA	17:38252660	-0.0714	0.00745	1.52E-21
THRAP3	1:36791837	0.0513	0.00601	1.79E-17
THUMPD1	16:20745255	0.03	0.00425	2.03E-12
THUMPD2	2:40006430	0.0444	0.00345	1.96E-37
THUMPD3	3:9438746	0.0884	0.00381	3.05E-113
TIAL1	10:121392582	-0.0427	0.00637	2.16E-11
TIAM1	21:32817485	0.101	0.00471	2.08E-97
TIAM2	6:155601105	-0.749	0.0236	1.62E-201
TIFA	4:113196906	-0.0716	0.00534	2.55E-40
TIGD2	4:90040847	0.0972	0.0103	4.71E-21
TIGD7	16:3338167	-0.0506	0.00624	5.64E-16
TIGIT	3:114013479	-0.0415	0.00617	1.99E-11
TIMD4	5:156435250	-0.0665	0.00665	2.18E-23
TIMM10	11:57283988	-0.286	0.00718	3.97E-303
TIMM13	19:2418264	-0.149	0.00762	5.19E-82
TIMM21	18:71814750	-0.0787	0.0125	3.29E-10
TIMM44	19:7999372	-0.0412	0.00665	6.22E-10
TIMMDC1	3:119236179	0.148	0.0112	2.85E-39
TIMP2	17:76907666	0.113	0.00639	2.08E-68
TIPARP	3:156425057	0.0611	0.0101	1.43E-09
TIPIN	15:66625724	0.297	0.0114	1.43E-140
TIPRL	1:168137725	0.0952	0.00877	3.55E-27
TIRAP	11:126133182	0.0437	0.00361	3.33E-33
TJP2	9:71787927	0.112	0.0032	4.62E-243
TJP3	19:3749971	-0.0162	0.00199	4.29E-16
TK2	16:66583994	-0.498	0.0125	8.23E-303
TKT	3:53286932	0.111	0.00446	5.07E-130
TLE3	15:70349578	-0.0317	0.0035	2.13E-19
TLK1	2:172031967	0.0426	0.00667	1.87E-10
TLR1	4:38802913	0.217	0.00736	2.33E-177
TLR10	4:38773109	0.129	0.00734	1.23E-67
TLR4	9:120463139	0.159	0.00591	1.41E-149
TLR5	1:223282292	0.128	0.00711	4.91E-70
TLR6	4:38880313	-0.11	0.00636	2.10E-65
TM2D1	1:62189541	-0.0528	0.00546	6.12E-22
TM2D2	8:38841052	-0.0448	0.00481	1.77E-20
TM2D3	15:102225621	0.0621	0.00365	3.60E-63
TM6SF1	15:83762285	-0.0778	0.00588	2.29E-39
TM7SF3	12:27103588	-0.0366	0.00569	1.39E-10

TM9SF1	14:24636025	-0.0333	0.00476	2.72E-12
TM9SF3	10:98357129	0.0323	0.00451	9.69E-13
TM9SF4	20:30770988	0.0244	0.00305	1.39E-15
TMA16	4:164418356	0.0795	0.00481	6.32E-60
TMBIM1	2:219133137	0.0767	0.00364	8.37E-95
TMBIM4	12:66510122	0.0314	0.00316	5.34E-23
TMC4	19:54674742	0.0969	0.00646	8.77E-50
TMC5	16:19486687	0.0347	0.00372	1.46E-20
TMC8	17:76135412	0.0275	0.00392	2.58E-12
TMCC3	12:95074582	0.0577	0.00629	6.33E-20
TMCO3	13:114175004	-0.0549	0.00408	1.26E-40
TMED2	12:124080619	-0.042	0.00409	1.89E-24
TMED5	1:93673898	0.0288	0.00454	2.41E-10
TMED6	16:69377553	0.0507	0.00577	2.00E-18
TMEFF1	9:103189338	0.0351	0.00478	2.59E-13
TMEM101	17:42071537	0.0641	0.00894	8.32E-13
TMEM106B	7:12257169	-0.0904	0.00382	6.31E-118
TMEM106C	12:48358279	-0.0601	0.00422	4.61E-45
TMEM109	11:60667504	-0.0325	0.00315	1.29E-24
TMEM110	3:52871929	-0.0469	0.00477	1.41E-22
TMEM116	12:112446710	-0.0842	0.00797	8.55E-26
TMEM119	12:108987230	-0.0971	0.0126	1.70E-14
TMEM123	11:102322459	-0.134	0.0207	8.87E-11
TMEM127	2:96917588	0.0318	0.00331	1.09E-21
TMEM135	11:86933855	0.0467	0.00755	6.71E-10
TMEM140	7:134855725	-0.0459	0.00546	5.56E-17
TMEM144	4:159092633	0.242	0.00786	3.12E-191
TMEM147	19:36038390	0.0345	0.00394	2.69E-18
TMEM14A	6:52535274	0.0572	0.007	3.84E-16
TMEM14E	3:152094201	0.0466	0.00519	3.58E-19
TMEM154	4:153619325	-0.146	0.0186	5.84E-15
TMEM156	4:39036648	0.133	0.0117	1.19E-29
TMEM158	3:45280071	0.0241	0.00391	7.25E-10
TMEM159	16:21192098	-0.0751	0.00618	1.58E-33
TMEM163	2:135206340	0.0558	0.00825	1.53E-11
TMEM165	4:56331332	-0.0589	0.00401	6.35E-48
TMEM169	2:216955910	-0.0297	0.00437	1.19E-11
TMEM170A	16:75477714	-0.0648	0.00764	3.02E-17
TMEM171	5:72367166	0.0594	0.0066	3.01E-19

TMEM176A	7:150457308	-0.874	0.00868	<1.0E-314
TMEM176B	7:150455295	0.664	0.0159	<1.0E-314
TMEM18	2:677213	0.0317	0.00419	4.66E-14
TMEM180	10:104237007	0.0438	0.00262	6.58E-61
TMEM184B	22:38622598	0.048	0.0045	3.02E-26
TMEM185B	2:121012836	-0.0534	0.00736	4.72E-13
TMEM189	20:48757132	0.0212	0.00268	3.24E-15
TMEM199	17:26670627	0.0563	0.00417	7.44E-41
TMEM2	9:74377528	-0.054	0.00698	1.17E-14
TMEM204	16:1583634	-0.15	0.00523	7.78E-169
TMEM205	19:11431901	-0.0975	0.0108	3.29E-19
TMEM206	1:212521242	-0.0304	0.00428	1.29E-12
TMEM209	7:129861638	0.132	0.00857	3.79E-52
TMEM216	11:61165280	-0.0871	0.00653	6.67E-40
TMEM220	17:10618661	-0.115	0.00443	6.65E-141
TMEM241	18:21043106	-0.0236	0.00399	3.40E-09
TMEM242	6:157707709	-0.181	0.0129	2.84E-44
TMEM245	9:111889172	-0.102	0.00451	1.82E-107
TMEM252	9:71175565	-0.185	0.00816	5.21E-109
TMEM254	10:81830894	-0.0509	0.00556	7.38E-20
TMEM258	11:61560081	-0.186	0.00472	1.97E-298
TMEM30A	6:76004562	0.0396	0.00394	1.20E-23
TMEM33	4:41960607	0.0726	0.00749	4.73E-22
TMEM43	3:14167761	0.0264	0.00391	1.53E-11
TMEM45A	3:100211602	0.0502	0.00451	2.03E-28
TMEM45B	11:129702471	0.281	0.0093	8.60E-185
TMEM50B	21:34842725	-0.127	0.00376	8.79E-225
TMEM51	1:15462172	-0.0718	0.00476	2.02E-50
TMEM55A	8:91959989	0.0292	0.00494	3.66E-09
TMEM60	7:77397379	-0.064	0.00497	2.18E-37
TMEM62	15:43510025	0.0441	0.00742	2.89E-09
TMEM65	8:125287350	-0.0351	0.00528	3.16E-11
TMEM66	8:29927856	-0.0471	0.00323	1.97E-47
TMEM68	8:56660888	0.0325	0.00533	1.16E-09
TMEM70	8:74888049	0.0263	0.00408	1.27E-10
TMEM71	8:133715804	-0.0835	0.00466	9.28E-70
TMEM80	11:695842	-0.118	0.00602	2.30E-82
TMEM81	1:205039288	0.0435	0.00661	5.03E-11
TMEM87B	2:112803111	0.116	0.00471	8.89E-126

TMEM8A	16:430469	0.135	0.00479	7.43E-162
TMEM9	1:201125326	-0.032	0.00315	4.93E-24
TMEM91	19:41888850	0.198	0.016	1.24E-34
TMEM92	17:48360903	0.0844	0.012	2.32E-12
TMEM97	17:26688663	0.0615	0.00569	5.80E-27
TMEM98	17:31298330	-0.0742	0.00759	2.11E-22
TMEM99	17:39006842	-0.0975	0.00701	2.89E-43
TMEM9B	11:9010134	0.159	0.00838	3.95E-78
TMOD2	15:52048251	-0.118	0.00537	5.16E-103
TMOD3	15:52076222	0.0698	0.00859	5.64E-16
TMPRSS11D	4:68688583	-0.315	0.0251	1.18E-35
TMPRSS3	21:43824106	0.0258	0.00279	2.88E-20
TMSB10	2:85133320	0.0742	0.00791	9.09E-21
TMTC1	12:29905322	-0.392	0.00708	<1.0E-314
TMTC2	12:83328642	-0.043	0.00635	1.39E-11
TMTC3	12:88639690	-0.175	0.0201	4.66E-18
TMTC4	13:101344407	-0.109	0.00363	1.82E-181
TMX1	14:51720038	-0.0758	0.00469	2.09E-57
TMX3	18:66391816	-0.0596	0.00565	8.87E-26
TMX4	20:7980390	0.0956	0.00496	4.09E-80
TNF	6:31536224	-0.0421	0.00393	1.35E-26
TNFAIP2	14:103606468	0.0392	0.00539	3.85E-13
TNFAIP6	2:152164587	-0.292	0.0102	1.07E-168
TNFAIP8	5:118717753	0.0599	0.00406	2.90E-48
TNFRSF10A	8:23082971	0.0329	0.00497	4.13E-11
TNFRSF10B	8:22941923	-0.125	0.00396	1.05E-201
TNFRSF10C	8:22957606	0.125	0.0068	7.35E-73
TNFRSF13C	22:42337040	-0.0521	0.00721	5.80E-13
TNFRSF17	16:12071341	0.114	0.0129	1.52E-18
TNFRSF1A	12:6445329	0.0712	0.00392	2.02E-71
TNFRSF1B	1:12243654	0.0274	0.0039	2.52E-12
TNFRSF9	1:8002098	0.108	0.0126	8.26E-18
NFSF12-TNFSF1	17:7460517	-0.0551	0.00374	3.17E-48
TNFSF13B	13:108916613	0.286	0.0334	1.59E-17
TNFSF14	19:6669934	-0.148	0.00889	1.49E-60
TNFSF15	9:117568766	-0.0523	0.00508	1.29E-24
TNFSF4	1:173162218	0.178	0.00927	1.25E-79
TNFSF8	9:117624799	0.0942	0.00592	1.12E-55
TNIP1	5:150436158	0.0271	0.00397	9.47E-12

TNKS2	10:93650630	-0.0386	0.00422	8.08E-20
TNNC2	20:44423843	-0.059	0.00539	1.26E-27
TNNI3	19:55672784	-0.0612	0.00942	8.95E-11
TNNI3K	1:74689511	-0.0272	0.00286	3.07E-21
TNNT1	19:55690751	-0.334	0.0206	1.14E-57
TNNT3	11:1942575	0.0979	0.0063	2.30E-53
TNPO1	5:72124610	0.0254	0.00404	3.62E-10
TNPO3	7:128723327	0.0978	0.0038	1.03E-137
TNRC6A	16:24842933	0.046	0.00596	1.32E-14
TNS1	2:218710924	-0.0394	0.0051	1.36E-14
TNS3	7:47319723	-0.0207	0.00249	9.23E-17
TOB1	17:48973032	0.0503	0.00475	6.72E-26
TOB2	22:41850500	0.0271	0.00408	3.65E-11
TOM1	22:35707236	0.0736	0.00296	6.95E-129
TOM1L2	17:17848602	0.0299	0.00445	2.00E-11
TOMM34	20:43589041	0.109	0.00648	2.11E-61
TOMM40L	1:161179877	0.0299	0.00411	3.86E-13
TOMM7	7:22848987	-0.0577	0.00413	1.31E-43
TOP1MT	8:144404964	0.0579	0.00888	7.53E-11
TOP3B	22:22301254	0.0342	0.00417	2.95E-16
TOR1A	9:132557210	0.0524	0.00368	3.69E-45
TOR1AIP1	1:179858333	-0.0455	0.00346	7.24E-39
TOR1B	9:132568500	0.173	0.00517	1.35E-223
TOX	8:60078682	-0.085	0.00766	2.73E-28
TOX4	14:21946473	-0.0368	0.00402	7.93E-20
TP53BP2	1:223954617	0.0472	0.00545	5.85E-18
TP53I3	2:24347093	0.0874	0.00539	7.60E-58
TP53INP1	8:95966531	-0.0351	0.00395	8.43E-19
TPCN2	11:68863048	0.0287	0.00246	4.78E-31
TPD52	8:81049437	0.0732	0.00488	8.88E-50
TPGS2	18:34324376	0.139	0.00903	1.85E-52
TPI1	12:7014962	-0.252	0.0147	7.11E-64
TPK1	7:144547618	0.113	0.0045	1.28E-131
TPM1	15:63338457	0.157	0.00847	3.45E-74
TPM2	9:35693121	0.208	0.00622	5.03E-222
TPMT	6:18117868	-0.068	0.00941	5.63E-13
TPP2	13:103284767	-0.0249	0.00308	7.96E-16
TPRG1	3:188868715	0.15	0.0116	7.87E-38
TPRG1L	1:3563428	0.101	0.00666	2.65E-51

TPST1	7:65823230	0.228	0.0103	2.99E-104
TPST2	22:27033321	0.0249	0.0036	4.63E-12
TPX2	20:30351560	0.0377	0.00427	1.57E-18
TRABD	22:50619920	-0.0427	0.00409	3.39E-25
TRAF3	14:103243408	0.0356	0.00387	5.22E-20
TRAF3IP1	2:239235857	-0.0255	0.00381	2.27E-11
TRAF3IP2	6:111904737	0.0254	0.0031	2.99E-16
TRAF3IP3	1:209930585	0.0831	0.00379	2.64E-102
TRAF5	1:211530044	-0.0349	0.00456	2.54E-14
TRAK1	3:42231638	-0.0335	0.00361	2.87E-20
TRAM1	8:71489682	0.0513	0.00381	9.16E-41
TRAM2	6:52442880	-0.0295	0.00407	4.58E-13
TRAP1	16:3743046	0.0838	0.00347	2.44E-122
TRAPPC10	21:45416953	0.0301	0.00427	2.08E-12
TRAPPC11	4:184636217	0.0271	0.00324	7.23E-17
TRAPPC12	2:3487331	-0.0399	0.00497	1.10E-15
TRAPPC2L	16:88919457	0.0437	0.0052	5.81E-17
TRAPPC4	11:118880668	-0.113	0.00563	4.34E-87
TRAPPC6B	14:39587438	0.0433	0.00593	3.10E-13
TRAPPC8	18:29432624	0.0291	0.00282	1.13E-24
TRAPPC9	8:141488651	0.114	0.00584	1.08E-81
TRAT1	3:108530225	-0.0823	0.00981	6.24E-17
TRDMT1	10:17237954	0.125	0.00544	6.78E-111
TREM1	6:41239936	0.328	0.0113	2.75E-172
TREML2	6:41163700	-0.0342	0.00421	5.39E-16
TREML4	6:41183349	-0.447	0.00852	<1.0E-314
TRERF1	6:42190556	-0.281	0.00842	2.95E-221
TRIB2	2:12858078	-0.0656	0.00538	9.03E-34
TRIM10	6:30122657	0.0977	0.00869	5.47E-29
TRIM13	13:50590291	0.0312	0.00499	4.27E-10
TRIM14	9:100862339	0.0719	0.00653	6.60E-28
TRIM2	4:154142936	-0.0258	0.00401	1.34E-10
TRIM21	11:4422642	-0.0409	0.00525	7.53E-15
TRIM22	11:5721286	0.0497	0.00736	1.53E-11
TRIM26	6:30163955	-0.0708	0.00368	9.52E-80
TRIM27	6:28891458	-0.0811	0.00307	1.55E-144
TRIM32	9:119471408	0.04	0.00576	4.03E-12
TRIM33	1:115084850	-0.121	0.0147	2.16E-16
TRIM35	8:27168902	0.0372	0.00278	4.53E-40

TRIM36	5:114542108	-0.0359	0.00365	1.36E-22
TRIM37	17:57010225	0.131	0.00306	<1.0E-314
TRIM38	6:25953274	-0.037	0.0046	1.02E-15
TRIM4	7:99500911	-0.0542	0.00378	7.17E-46
TRIM41	5:180661980	0.0417	0.00672	6.01E-10
TRIM5	11:5701001	0.0506	0.00605	8.26E-17
TRIM52	5:180703001	-0.0409	0.00532	1.85E-14
TRIM58	1:248011866	0.123	0.00901	8.22E-42
TRIM68	11:4621485	0.0857	0.0083	9.69E-25
TRIM6-TRIM34	11:5664684	-0.0695	0.00888	6.12E-15
TRIM8	10:104421679	-0.0291	0.00435	2.31E-11
TRIM9	14:51528533	-0.0662	0.00687	9.17E-22
TRIOBP	22:38122462	-0.0414	0.00313	2.34E-39
TRIP11	14:92518233	0.123	0.0204	1.55E-09
TRIP12	2:230826145	-0.0513	0.00488	1.56E-25
TRIT1	1:40295408	-0.0869	0.00465	1.95E-75
TRMT10A	4:100525934	-0.0556	0.0059	6.44E-21
TRMT10B	9:37756833	0.066	0.00595	2.84E-28
TRMT112	11:64078943	0.154	0.0119	8.87E-38
TRMT12	8:125461287	0.139	0.00425	3.96E-214
TRMT1L	1:185125043	0.0418	0.00702	2.77E-09
TRMT44	4:8454639	-0.0305	0.00366	1.02E-16
TRMU	22:46698662	-0.0793	0.0119	3.22E-11
TRNT1	3:3185386	-0.0389	0.00356	1.95E-27
TROVE2	1:193054154	0.0956	0.00469	7.28E-89
TRPC1	3:142436807	0.07	0.00673	4.35E-25
TRPC3	4:122899165	-0.0452	0.00338	3.46E-40
TRPC4AP	20:33584289	-0.02	0.00269	1.14E-13
TRPC6	11:101384612	-0.078	0.00387	3.97E-87
TRPM6	9:77486124	0.128	0.00566	1.57E-107
TRPM7	15:50942452	-0.0489	0.00293	4.22E-61
TRPT1	11:63990302	0.0461	0.00646	1.08E-12
TRRAP	7:98541261	-0.0374	0.00506	1.63E-13
TSEN15	1:184023529	0.0356	0.0049	4.31E-13
TSEN2	3:12525656	-0.0943	0.00644	1.21E-47
TSEN34	19:54699252	-0.0597	0.00753	2.63E-15
TSGA10	2:99704273	0.24	0.00462	<1.0E-314
TSHR	14:81660997	-0.0195	0.00279	2.93E-12
TSHZ2	20:52130969	-0.451	0.0278	1.04E-57

TSHZ3	19:31842495	-0.0969	0.0113	1.24E-17
TSPAN14	10:82277341	-0.0331	0.00399	1.43E-16
TSPAN15	10:71215799	-0.0428	0.00409	2.36E-25
TSPAN16	19:11431901	0.353	0.0126	1.13E-159
TSPAN18	11:44835771	-0.0389	0.00591	4.93E-11
TSPAN2	1:115602498	0.142	0.0124	6.80E-30
TSPAN3	15:77369520	-0.175	0.00563	9.31E-195
TSPAN33	7:128777461	0.0614	0.00892	6.47E-12
TSPO	22:43559137	-0.0656	0.00604	3.67E-27
TSPYL1	6:116597724	-0.0211	0.00273	1.21E-14
TSPYL4	6:116530856	0.0226	0.00378	2.41E-09
TSPYL5	8:98267908	0.103	0.0137	5.46E-14
TSSC1	2:3188755	-0.0458	0.00565	6.74E-16
TST	22:37421469	-0.054	0.00668	7.52E-16
TTBK2	15:43241798	0.0263	0.00267	1.13E-22
TTC12	11:113187949	-0.139	0.00539	1.03E-137
TTC13	1:231087722	0.0315	0.00262	1.04E-32
TTC14	3:180272509	-0.0417	0.00583	9.92E-13
TTC17	11:43505707	0.0381	0.0037	1.09E-24
TTC18	10:75092353	0.0428	0.00678	2.92E-10
TTC19	17:15945608	-0.024	0.00325	1.62E-13
TTC21B	2:166727401	-0.0689	0.00474	5.31E-47
TTC23	15:99783965	0.0413	0.00303	1.08E-41
TTC25	17:40046186	0.0717	0.00921	8.47E-15
TTC26	7:138798004	-0.0668	0.00545	4.44E-34
TTC27	2:32903548	0.0548	0.0056	2.20E-22
TTC3	21:38503126	-0.121	0.00447	7.36E-152
TTC33	5:40698397	-0.0495	0.00591	6.41E-17
TTC37	5:94841313	-0.121	0.00586	1.01E-91
TTC38	22:46685017	-0.057	0.00862	3.93E-11
TTC39B	9:15294667	0.218	0.00532	<1.0E-314
TTC39C	18:21620065	-0.0521	0.00594	2.17E-18
TTC4	1:55182300	-0.11	0.00487	6.54E-108
TTF2	1:117605336	-0.224	0.00781	2.41E-168
TTK	6:80741418	-0.0199	0.00313	2.28E-10
TTLL1	22:43470175	-0.04	0.00546	2.49E-13
TTLL11	9:124756264	-0.0148	0.00228	9.76E-11
TTLL12	22:43553417	-0.0177	0.00297	2.82E-09
TTLL2	6:167767152	-0.0325	0.00478	1.28E-11

TTLL3	3:9871393	0.0263	0.00305	8.80E-18
TTLL4	2:219634722	-0.0654	0.00372	2.84E-67
TTLL5	14:76151532	0.0454	0.00329	1.49E-42
TUBA3FP	22:21365474	0.0454	0.00457	5.10E-23
TUBB	6:30690938	0.256	0.00948	2.73E-150
TUBB2A	6:3111956	2.13	0.0476	<1.0E-314
TUBB6	18:12303726	-0.133	0.00665	1.07E-85
TUBD1	17:57932216	0.0981	0.00648	8.35E-51
TUBE1	6:112414035	0.0266	0.0043	6.85E-10
TUBG2	17:40786248	-0.0432	0.00565	2.48E-14
TUBGCP3	13:113198538	0.0657	0.00511	3.04E-37
TUBGCP4	15:43680212	0.0741	0.00473	5.45E-54
TUBGCP5	15:22804640	0.0358	0.00518	5.06E-12
TUFM	16:28847246	-0.139	0.00458	1.08E-185
TUSC3	8:15524123	-0.0759	0.00929	3.95E-16
TVP23C	17:15389632	-0.0553	0.00692	1.67E-15
TWF1	12:44199961	-0.0476	0.00746	2.02E-10
TWISTNB	7:19747915	0.0414	0.00539	1.88E-14
TWSG1	18:9343958	-0.288	0.0254	1.90E-29
TXK	4:48089461	0.0959	0.00648	1.39E-48
TXNDC12	1:52520858	0.251	0.0255	1.18E-22
TXNDC15	5:134279940	-0.167	0.00488	1.85E-231
TXNL1	18:54281790	0.0918	0.00855	1.38E-26
TXNL4A	18:77752801	0.187	0.00619	1.33E-185
TXNRD1	12:104693089	0.0253	0.00322	4.78E-15
TXNRD2	22:19881556	0.0222	0.00251	1.42E-18
TYK2	19:10464687	0.0226	0.00304	1.42E-13
TYMP	22:50978520	-0.0568	0.00372	1.87E-51
TYMS	18:672043	-0.0707	0.00589	8.36E-33
TYW1	7:66633428	-0.214	0.022	3.62E-22
TYW3	1:75220929	0.0589	0.00594	6.44E-23
U2AF1	21:44508390	0.1	0.00474	7.86E-95
U2AF1L4	19:36234489	-0.33	0.0085	3.54E-290
UACA	15:71038162	-0.0731	0.00583	1.52E-35
UAP1L1	9:139958583	0.0825	0.00542	3.76E-51
UBA6	4:68556488	0.0445	0.00377	8.45E-32
UBA7	3:49798920	0.0802	0.00384	4.67E-93
UBAC2	13:99899478	0.0148	0.00219	1.55E-11
UBALD2	17:74261677	0.0439	0.0042	2.98E-25

UBASH3A	21:43855067	-0.122	0.00413	1.10E-176
UBASH3B	11:122583862	-0.0342	0.00454	5.15E-14
UBE2B	5:133672655	0.181	0.0215	4.53E-17
UBE2D1	10:60144073	-0.272	0.00578	<1.0E-314
UBE2D3	4:103683546	-0.0444	0.0038	3.95E-31
UBE2E2	3:23412528	-0.0473	0.00509	2.32E-20
UBE2F	2:238912478	-0.132	0.00941	3.76E-44
UBE2G2	21:46219184	-0.0368	0.0047	6.37E-15
UBE2I	16:1378788	-0.0579	0.00527	8.01E-28
UBE2K	4:39700173	0.0245	0.00352	3.44E-12
UBE2L3	22:21938863	-0.0422	0.00473	5.83E-19
UBE2O	17:74461577	-0.0777	0.0107	3.61E-13
UBE2Z	17:47026431	0.0811	0.00392	2.00E-91
UBE3B	12:109910798	-0.104	0.00635	5.43E-59
UBE3C	7:156935591	0.0372	0.00294	2.85E-36
UBE4A	11:118230359	-0.0612	0.00518	9.31E-32
UBE4B	1:10174189	0.0577	0.00517	1.24E-28
UBL3	13:30442076	-0.0318	0.00486	6.64E-11
UBLCP1	5:158726535	-0.0465	0.00556	7.58E-17
UBN1	16:4911239	-0.0348	0.0033	8.44E-26
UBQLN4	1:156007177	0.351	0.012	3.22E-175
UBR1	15:43220785	0.0223	0.00292	2.50E-14
UBR2	6:42537608	0.0605	0.00733	1.80E-16
UBR3	2:170872617	-0.0281	0.00332	3.15E-17
UBXN2A	2:24126904	-0.101	0.00616	2.07E-59
UBXN6	19:4400925	0.1	0.0102	2.28E-22
UCK1	9:134386425	-0.0253	0.00403	3.74E-10
UCP2	11:73686859	0.136	0.00484	5.93E-162
UEVLD	11:18574323	0.0631	0.00593	3.42E-26
UFC1	1:161128644	-0.177	0.0275	1.49E-10
UFM1	13:38926390	-0.147	0.0118	2.17E-35
UFSP2	4:186350063	-0.0554	0.00508	2.26E-27
UGDH	4:39518110	0.154	0.00458	4.03E-225
UGGT2	13:96681165	0.204	0.00891	7.48E-111
UGP2	2:64022346	0.0587	0.00835	2.42E-12
UGT2B28	4:70123614	0.64	0.0366	9.87E-67
UGT8	4:115519824	-0.0532	0.00575	2.90E-20
UHRF1BP1	6:34743432	-0.1	0.00284	3.78E-244
UHRF1BP1L	12:100519182	0.164	0.00837	5.49E-83

UIMC1	5:176427377	0.0335	0.00529	2.40E-10
ULK2	17:19697976	0.0344	0.00347	6.95E-23
ULK3	15:75128501	-0.0409	0.003	1.54E-41
ULK4	3:41765955	-0.162	0.00402	<1.0E-314
UMPS	3:124445764	-0.0694	0.00438	2.69E-55
UNC119B	12:121149560	0.188	0.0255	1.82E-13
UNG	12:109537423	0.0577	0.00476	2.34E-33
UPB1	22:24885927	-0.0492	0.00297	3.14E-60
UPF2	10:12100781	0.0394	0.00446	1.42E-18
UPF3A	13:115032817	-0.0523	0.0049	2.82E-26
UPK1B	3:118946797	-0.133	0.00786	4.55E-63
UPK3A	22:45679633	-0.0401	0.00365	8.94E-28
UPP1	7:48134719	0.0534	0.00354	3.12E-50
UQCR10	22:30187702	0.0345	0.00446	1.29E-14
UQCR11	19:1589064	-0.0782	0.00668	3.06E-31
UQCRC1	3:48636507	-0.162	0.00855	1.48E-77
UROD	1:45498181	0.0445	0.00482	3.65E-20
UROS	10:127496359	0.119	0.00453	2.07E-143
USB1	16:58027898	-0.113	0.00624	3.72E-71
USF1	1:161019040	-0.0348	0.00335	4.82E-25
USF2	19:35818609	-0.0155	0.00265	5.09E-09
USMG5	10:105125451	-0.0528	0.00533	6.14E-23
USO1	4:76643274	0.0548	0.00491	1.31E-28
USP1	1:62891354	-0.126	0.0179	2.26E-12
USP10	16:84740884	0.0561	0.00492	7.78E-30
USP12	13:27678791	-0.0858	0.0105	4.81E-16
USP15	12:62665787	-0.0623	0.00397	3.43E-54
USP16	21:30430978	-0.0417	0.00582	9.33E-13
USP20	9:132636601	0.0164	0.00213	1.83E-14
USP24	1:55575670	-0.0335	0.00416	1.00E-15
USP3	15:63794279	-0.0537	0.00319	5.62E-62
USP30	12:109490601	-0.0552	0.00506	2.07E-27
USP33	1:78129593	0.0502	0.00634	3.25E-15
USP34	2:61467379	0.017	0.00287	3.78E-09
USP36	17:76785240	-0.0467	0.00307	3.56E-51
USP37	2:219328577	-0.0252	0.0037	1.11E-11
USP38	4:144163755	0.0319	0.00287	2.18E-28
USP39	2:85860448	0.0966	0.00584	6.32E-60
USP4	3:49326178	0.0431	0.00478	2.54E-19

USP44	12:95960102	0.0825	0.00758	2.36E-27
USP45	6:99966805	-0.0337	0.00469	8.08E-13
USP47	11:11889754	-0.0315	0.00421	8.87E-14
USP48	1:22109370	-0.026	0.00312	1.06E-16
USP53	4:120106348	-0.525	0.0111	<1.0E-314
USP6	17:5083228	-0.104	0.0158	4.77E-11
USP6NL	10:11697424	0.0656	0.00948	5.12E-12
USP8	15:50734694	0.0761	0.00462	1.85E-59
UST	6:149166343	0.0855	0.00839	3.71E-24
UTS2	1:7911302	-1.42	0.033	<1.0E-314
UVSSA	4:1332142	-0.269	0.0205	1.26E-38
UXS1	2:106810767	-0.0422	0.007	1.70E-09
VAMP1	12:6580066	0.0539	0.00405	7.45E-40
VAMP2	17:8071352	-0.089	0.00793	6.45E-29
VAMP5	2:85801137	0.117	0.00838	9.21E-44
VAMP8	2:85794297	0.147	0.00434	1.53E-226
VAPA	18:9866979	-0.0526	0.00379	4.15E-43
VAPB	20:56955371	-0.0471	0.00625	5.66E-14
VASH1	14:77182302	-0.116	0.00277	<1.0E-314
VASH2	1:213213567	-0.0193	0.00256	4.78E-14
VAV2	9:136651629	-0.0286	0.00331	7.87E-18
VAV3	1:108297209	-0.0838	0.00613	7.36E-42
VCP	9:35011279	-0.041	0.00315	4.03E-38
VDR	12:48347133	-0.0222	0.00361	7.55E-10
VEGFA	6:43710959	-0.0644	0.00608	6.64E-26
VEPH1	3:157261335	-0.0409	0.00413	6.29E-23
VEZT	12:95687486	-0.154	0.00941	2.23E-58
VIL1	2:219279673	0.0276	0.00329	6.76E-17
VIM	10:17254832	0.031	0.00396	5.87E-15
VIPR1	3:42531939	0.0437	0.0034	3.31E-37
VKORC1L1	7:65337976	-0.195	0.0132	7.64E-49
VLDLR	9:2622278	-0.138	0.00727	2.57E-78
VN1R1	19:57961191	-0.0616	0.00558	4.35E-28
VNN1	6:133015662	-0.364	0.00902	<1.0E-314
VNN2	6:133076489	0.175	0.00606	9.01E-171
VNN3	6:133060195	0.247	0.0078	4.23E-201
VOPP1	7:55610829	0.0436	0.00622	2.90E-12
VPS11	11:118920453	0.0484	0.00432	7.28E-29
VPS13A	9:79819030	0.0765	0.00701	1.92E-27

VPS13C	15:62352374	-0.0618	0.00387	4.21E-56
VPS26A	10:70885320	0.105	0.00492	1.07E-96
VPS28	8:145635534	-0.212	0.00972	2.72E-101
VPS29	12:110939421	-0.0642	0.00424	7.25E-51
VPS33A	12:122679121	0.0295	0.00441	2.46E-11
VPS33B	15:91557186	0.0414	0.00392	7.25E-26
VPS36	13:52983981	0.0535	0.00337	1.19E-55
VPS37B	12:123361501	-0.0302	0.00481	3.72E-10
VPS41	7:38758326	-0.0473	0.0039	2.35E-33
VPS53	17:618701	0.121	0.00419	1.61E-169
VPS54	2:64139834	0.0369	0.00361	2.36E-24
VPS8	3:184775860	0.166	0.0101	3.71E-59
VRK1	14:97269197	-0.12	0.00852	6.06E-44
VRK2	2:58338086	-0.057	0.00401	5.89E-45
VRK3	19:50526994	0.0273	0.00344	2.27E-15
VSIG10	12:118501598	0.0746	0.00682	1.44E-27
VSTM1	19:54544575	-0.872	0.0201	<1.0E-314
VWA5A	11:123943259	0.0606	0.00467	7.05E-38
VWF	12:6205767	-0.165	0.0155	2.31E-26
WAC	10:28869729	0.0209	0.00321	8.80E-11
WAPAL	10:88160039	-0.0585	0.00626	1.33E-20
WARS	14:100840596	-0.288	0.0076	1.35E-277
WARS2	1:119577837	-0.0276	0.00389	1.36E-12
WASF1	6:110500355	0.0448	0.00534	5.98E-17
WASF3	13:27184184	-0.0241	0.00348	4.50E-12
WASL	7:123360007	0.0336	0.0034	8.38E-23
WBP11	12:14979169	0.0466	0.0054	8.65E-18
WBP2NL	22:42416987	0.032	0.00441	4.34E-13
WBP4	13:41673457	-0.0701	0.00772	1.52E-19
WBSCR22	7:73114645	-0.152	0.0106	6.33E-46
WBSCR27	7:73242908	-0.0228	0.00293	7.62E-15
WDFY1	2:224787704	0.0569	0.00407	8.57E-44
WDFY4	10:49933974	-0.243	0.00753	1.61E-208
WDPCP	2:63495875	0.0306	0.00346	1.14E-18
WDR1	4:10100801	-0.0947	0.00309	1.41E-190
WDR11	10:122601203	-0.14	0.00656	1.71E-96
WDR17	4:177020806	-0.151	0.0163	2.80E-20
WDR19	4:39140277	-0.0562	0.00371	9.41E-51
WDR25	14:100825670	-0.0315	0.00239	4.83E-39

WDR27	6:170063010	-0.0534	0.00499	2.02E-26
WDR3	1:118515496	-0.0692	0.00954	4.45E-13
WDR33	2:128414113	0.0341	0.00431	2.89E-15
WDR35	2:20134248	-0.0425	0.00688	7.40E-10
WDR36	5:110467753	-0.0256	0.00309	1.77E-16
WDR37	10:1102796	0.0213	0.00264	8.62E-16
WDR41	5:76780524	0.0911	0.00364	4.62E-131
WDR52	3:113196702	-0.0551	0.00409	8.58E-41
WDR55	5:140079408	0.0238	0.00325	2.85E-13
WDR5B	3:122163022	-0.0793	0.00456	5.66E-66
WDR6	3:49035885	-0.0579	0.00338	5.49E-64
WDR60	7:158676826	0.107	0.00766	7.85E-44
WDR61	15:78606170	-0.0549	0.00589	1.49E-20
WDR63	1:85514841	0.0332	0.00268	5.77E-35
WDR66	12:122400813	-0.0286	0.00241	3.39E-32
WDR67	8:124154275	0.0585	0.00564	6.37E-25
WDR7	18:54319099	0.0412	0.00392	1.40E-25
WDR73	15:85180319	0.0579	0.00443	2.31E-38
WDR75	2:190319341	0.111	0.00961	1.15E-30
WDR81	17:1690303	0.0147	0.00246	2.40E-09
WDR83OS	19:12780204	0.521	0.0558	1.29E-20
WDR85	9:140453479	0.0249	0.0042	3.32E-09
WDR91	7:134880884	0.0345	0.00286	4.86E-33
WDR92	2:68426624	0.0511	0.00348	9.56E-48
WDSUB1	2:160087815	-0.087	0.00572	3.60E-51
WDYHV1	8:124455097	0.052	0.00457	1.18E-29
WEE1	11:9592477	-0.0344	0.00509	1.63E-11
WFDC3	20:44402869	0.0943	0.00466	8.24E-88
WHSC1L1	8:38164650	0.0348	0.00429	6.29E-16
WIPI1	17:66449844	0.15	0.00649	5.37E-112
WIPI2	7:5253775	0.161	0.0124	5.43E-38
WLS	1:68737383	-0.493	0.0137	6.23E-254
WNT4	1:22446108	0.0228	0.00308	1.44E-13
WRB	21:40741846	-0.28	0.00667	<1.0E-314
WRN	8:30957765	0.0362	0.00561	1.18E-10
WRNIP1	6:2765697	0.0511	0.00274	3.85E-75
WSB1	17:25647880	0.0753	0.00411	1.00E-72
WSB2	12:118501598	0.0688	0.00734	9.69E-21
WTAP	6:160182796	-0.169	0.0107	1.69E-54

WWC2	4:184019859	-0.106	0.0101	1.41E-25
WWOX	16:78282458	0.0226	0.00297	2.60E-14
WWP1	8:87446737	0.217	0.0198	1.74E-27
WWP2	16:69978687	-0.0766	0.00401	1.68E-78
XBP1	22:29234593	-0.0512	0.00384	6.82E-40
XIRP2	2:167962531	-0.0259	0.00413	3.77E-10
XKR3	22:17233296	-1.21	0.0267	<1.0E-314
XKR6	8:10964373	0.0885	0.00946	1.26E-20
XKR8	1:28278138	0.0253	0.00347	3.36E-13
XPNPEP1	10:111669132	0.0588	0.00616	1.98E-21
XPO1	2:61763207	0.0262	0.00425	7.41E-10
XPO4	13:21442321	-0.0209	0.00339	8.02E-10
XPO6	16:28241942	-0.0487	0.00379	3.12E-37
XPO7	8:21739832	0.067	0.0113	2.88E-09
XRCC1	19:44011568	0.0442	0.00541	3.98E-16
XRCC2	7:152337646	-0.0762	0.00472	3.03E-57
XRCC3	14:104131033	0.0309	0.00294	1.71E-25
XRCC4	5:82637324	0.0553	0.00858	1.25E-10
XRCC5	2:216980258	0.0257	0.00289	8.07E-19
XRCC6BP1	12:58342196	0.0706	0.00403	7.66E-67
XRN2	20:21258707	0.0261	0.00415	3.71E-10
XRRA1	11:74513449	-0.227	0.00299	<1.0E-314
XYLB	3:38487802	-0.0851	0.00304	5.71E-161
XYLT1	16:17542995	0.0411	0.00365	5.16E-29
YAF2	12:42637999	0.0313	0.00409	2.21E-14
YEATS4	12:69732105	0.21	0.00487	<1.0E-314
YIF1B	19:38796578	-0.0523	0.00398	8.19E-39
YIPF1	1:54337886	-0.0262	0.00414	2.46E-10
YIPF3	6:43534399	-0.0628	0.00891	1.95E-12
YIPF5	5:143550217	0.124	0.00449	1.35E-156
YME1L1	10:27392343	0.0316	0.00416	3.99E-14
YPEL1	22:22055280	-0.0842	0.00481	8.11E-67
YPEL3	16:30118345	-0.0944	0.00799	9.02E-32
YTHDC2	5:112937962	-0.0664	0.00316	4.69E-94
YTHDF3	8:64084388	0.0754	0.00472	3.35E-56
YWHAB	20:43520287	0.081	0.00408	1.38E-84
YWHAH	22:32355011	-0.0694	0.00603	2.52E-30
ZACN	17:74077797	0.0293	0.00317	3.58E-20
ZADH2	18:72910687	0.125	0.00381	7.57E-216

ZAK	2:174030566	0.0813	0.00573	6.84E-45
ZBED4	22:50249677	-0.0307	0.00424	5.33E-13
ZBED5	11:10874594	0.148	0.00847	7.30E-67
ZBTB20	3:114792654	0.146	0.00671	3.25E-100
ZBTB25	14:64960631	0.046	0.00596	1.38E-14
ZBTB39	12:57403340	0.101	0.0123	4.11E-16
ZBTB40	1:22809338	-0.0335	0.00313	2.36E-26
ZBTB43	9:129575594	0.0359	0.00547	5.63E-11
ZBTB49	4:4306263	-0.0386	0.00351	7.33E-28
ZBTB7B	1:154989595	-0.0313	0.0043	3.69E-13
ZC2HC1A	8:79555186	0.301	0.00692	<1.0E-314
ZC3H13	13:46641466	-0.0358	0.00528	1.34E-11
ZC3H15	2:187346255	-0.029	0.00394	1.90E-13
ZC3H18	16:88680855	0.0183	0.00298	8.13E-10
ZC3H6	2:113007757	0.0646	0.00618	2.60E-25
ZC3H7A	16:11878033	-0.0313	0.00374	8.10E-17
ZC3HAV1	7:138815913	-0.0721	0.00818	1.54E-18
ZC3HAV1L	7:138724105	-0.0446	0.00474	6.80E-21
ZCCHC11	1:52964160	-0.119	0.0136	2.67E-18
ZCCHC14	16:87426667	-0.033	0.00307	1.02E-26
ZCCHC17	1:31755501	0.0543	0.00508	2.32E-26
ZCCHC7	9:37362160	0.0429	0.00355	3.50E-33
ZCCHC9	5:80575775	-0.0253	0.00348	4.25E-13
ZCRB1	12:42767121	-0.0309	0.00475	8.00E-11
ZDHHC13	11:19140053	-0.0944	0.00536	1.62E-67
ZDHHC14	6:157975829	0.0257	0.00416	6.54E-10
ZDHHC17	12:77153644	0.133	0.0151	1.55E-18
ZDHHC18	1:27104612	-0.072	0.00744	5.54E-22
ZDHHC2	8:17013199	-0.209	0.00927	6.70E-108
ZDHHC20	13:22009299	0.0752	0.00449	2.02E-61
ZDHHC21	9:14694059	-0.1	0.00564	8.77E-69
ZDHHC4	7:6591041	-0.0679	0.00391	9.61E-66
ZDHHC6	10:114186157	0.0599	0.00514	5.48E-31
ZDHHC7	16:85039732	-0.0492	0.00444	3.16E-28
ZFAND1	8:82613459	0.0521	0.00643	7.11E-16
ZFAND2A	7:1199117	0.115	0.00427	1.10E-150
ZFAND3	6:38136736	-0.0514	0.00504	3.45E-24
ZFAND4	10:46172739	-0.12	0.00818	1.21E-47
ZFAND5	9:75021526	-0.0434	0.00477	1.22E-19

ZFAT	8:135613194	0.0309	0.00305	5.28E-24
ZFP1	16:75187465	0.0547	0.00422	7.76E-38
ZFP161	18:5278970	0.0459	0.00417	6.26E-28
ZFP28	19:57092369	0.0833	0.00358	3.61E-114
ZFP30	19:38132334	-0.054	0.00376	7.12E-46
ZFP36L1	14:69206887	0.0607	0.0082	1.49E-13
ZFP36L2	2:43452793	-0.0405	0.00598	1.43E-11
ZFP82	19:36833798	-0.0405	0.0055	2.14E-13
ZFP90	16:68577567	0.178	0.00602	2.05E-177
ZFP91	11:58346153	-0.0281	0.00362	9.71E-15
ZFYVE1	14:73455189	0.0252	0.00325	1.22E-14
ZFYVE19	15:41112360	0.022	0.00258	2.33E-17
ZFYVE27	10:99492671	0.0227	0.0035	9.46E-11
ZG16B	16:2880614	0.0715	0.00423	1.80E-62
ZKSCAN1	7:99608029	-0.0288	0.00367	5.58E-15
ZKSCAN3	6:28386739	0.0465	0.00496	9.79E-21
ZKSCAN5	7:99102911	0.0683	0.00431	3.99E-55
ZMAT2	5:140032947	-0.0667	0.00743	3.54E-19
ZMAT3	3:178777166	0.0268	0.00345	9.24E-15
ZMAT4	8:40618018	-0.131	0.00875	1.20E-49
ZMPSTE24	1:40676406	0.0532	0.00819	8.98E-11
ZMYM5	13:20376135	0.0824	0.00502	3.64E-59
ZMYND12	1:42912305	0.0317	0.00373	2.54E-17
ZNF10	12:133761543	0.043	0.00554	1.07E-14
ZNF100	19:21873996	-0.343	0.00738	<1.0E-314
ZNF107	7:64208563	-0.136	0.0102	3.27E-40
ZNF121	19:9688068	0.124	0.0136	7.00E-20
ZNF124	1:247289279	0.0875	0.00484	5.20E-71
ZNF131	5:43147919	0.0622	0.00796	6.71E-15
ZNF132	19:58939389	-0.0763	0.00442	3.98E-65
ZNF134	19:58134272	0.0714	0.00901	2.86E-15
ZNF137P	19:53092008	0.159	0.00943	3.49E-62
ZNF138	7:64274517	0.238	0.00588	<1.0E-314
ZNF141	4:388013	0.061	0.00993	8.51E-10
ZNF143	11:9497952	-0.171	0.00708	2.84E-122
ZNF146	19:36724666	-0.048	0.0069	4.02E-12
ZNF154	19:58230279	-0.292	0.0277	1.12E-25
ZNF155	19:44488352	0.337	0.00899	2.06E-272
ZNF160	19:53589049	0.104	0.00563	6.13E-74

ZNF167	3:44592979	-0.0563	0.00488	2.13E-30
ZNF169	9:97033949	-0.0384	0.0035	1.26E-27
ZNF175	19:52056246	0.0706	0.00549	2.50E-37
ZNF177	19:9434872	0.332	0.016	5.89E-92
ZNF180	19:45004693	0.0446	0.0054	1.67E-16
ZNF189	9:104161942	-0.0489	0.00697	2.46E-12
ZNF19	16:71558970	-0.0388	0.00627	6.61E-10
ZNF192	6:28084733	-0.059	0.00685	8.94E-18
ZNF193	6:28197493	-0.165	0.0139	3.12E-32
ZNF195	11:3378063	0.0709	0.00733	5.41E-22
ZNF197	3:44737806	-0.0663	0.00455	3.84E-47
ZNF200	16:3310883	0.0475	0.0043	4.03E-28
ZNF204P	6:27377843	-0.0362	0.00424	1.60E-17
ZNF205	16:3168836	-0.0162	0.00217	9.19E-14
ZNF207	17:30692396	0.0415	0.00547	3.85E-14
ZNF208	19:22161490	-0.431	0.0176	3.32E-125
ZNF211	19:58154473	0.0697	0.00649	1.30E-26
ZNF213	16:3148130	0.0196	0.00215	1.22E-19
ZNF215	11:6948139	-0.107	0.00323	4.53E-218
ZNF217	20:52186784	-0.0402	0.00435	4.02E-20
ZNF223	19:44567510	-0.228	0.00816	6.31E-160
ZNF226	19:44719996	-0.0328	0.00444	1.74E-13
ZNF227	19:44732658	0.123	0.0159	8.91E-15
ZNF23	16:71436836	-0.0352	0.00476	1.68E-13
ZNF230	19:44510933	0.0851	0.00548	3.27E-53
ZNF234	19:44645652	0.0477	0.00487	1.90E-22
ZNF236	18:74568937	0.0199	0.00208	1.52E-21
ZNF239	10:44038969	0.039	0.0051	2.21E-14
ZNF24	18:32957283	0.0603	0.00338	2.70E-69
ZNF248	10:38066592	-0.0671	0.00837	1.24E-15
ZNF253	19:19954714	-0.0398	0.00659	1.61E-09
ZNF260	19:36986063	-0.0735	0.00573	4.42E-37
ZNF266	19:9473201	0.265	0.00433	<1.0E-314
ZNF271	18:32905710	-0.079	0.0054	1.74E-47
ZNF273	7:64415252	-0.0509	0.00761	2.51E-11
ZNF280D	15:57014578	-0.0226	0.00317	1.14E-12
ZNF281	1:200326810	-0.0482	0.00393	4.50E-34
ZNF286A	17:15592879	-0.843	0.0178	<1.0E-314
ZNF30	19:35455130	0.0568	0.00882	1.31E-10

ZNF300P1	5:150280562	-0.0356	0.0052	8.42E-12
ZNF302	19:35153457	0.166	0.00689	1.54E-121
ZNF304	19:57909297	0.0483	0.00686	2.22E-12
ZNF317	19:9259777	-0.0383	0.0046	1.15E-16
ZNF318	6:43385975	-0.0783	0.00683	4.90E-30
ZNF321P	19:53448098	-0.064	0.00701	9.39E-20
ZNF323	6:28325308	-0.0215	0.00308	3.02E-12
ZNF329	19:58605735	-0.052	0.00704	1.77E-13
ZNF331	19:54017032	0.0396	0.0066	2.10E-09
ZNF333	19:14803329	-0.0632	0.00262	2.25E-122
ZNF337	20:25661716	0.0999	0.0101	4.98E-23
ZNF33A	10:38336324	-0.0665	0.00818	5.58E-16
ZNF34	8:146017782	-0.0225	0.00341	4.33E-11
ZNF343	20:2488941	0.0333	0.00464	8.90E-13
ZNF347	19:53667147	0.0418	0.00632	4.26E-11
ZNF354A	5:178094484	0.279	0.00686	<1.0E-314
ZNF354B	5:178295339	-0.0491	0.00795	6.96E-10
ZNF354C	5:178447321	-0.0346	0.00454	3.28E-14
ZNF362	1:33723211	0.028	0.00331	3.91E-17
ZNF37A	10:38461675	-0.0651	0.00487	4.29E-40
ZNF37BP	10:43095722	-0.131	0.00855	3.63E-52
ZNF395	8:28259089	-0.0231	0.00381	1.57E-09
ZNF415	19:53651845	-0.183	0.0097	3.30E-77
ZNF417	19:58447904	-0.0594	0.00835	1.34E-12
ZNF418	19:58464224	-0.136	0.0105	8.89E-38
ZNF425	7:148823366	0.059	0.00518	1.19E-29
ZNF429	19:21664201	0.723	0.0178	<1.0E-314
ZNF438	10:31366929	0.048	0.00655	2.65E-13
ZNF443	19:12534589	0.0674	0.00696	5.47E-22
ZNF45	19:44380536	-0.0377	0.00524	7.38E-13
ZNF460	19:57798198	-0.111	0.00499	1.68E-105
ZNF467	7:149476324	0.0385	0.00587	5.81E-11
ZNF468	19:53367338	-0.147	0.0135	1.91E-27
ZNF470	19:57092369	0.0641	0.00445	3.34E-46
ZNF471	19:57060087	-0.0478	0.00423	2.26E-29
ZNF473	19:50529054	-0.0258	0.00429	1.87E-09
ZNF480	19:52826693	0.182	0.0273	3.32E-11
ZNF483	9:114297244	-0.0837	0.0114	2.33E-13
ZNF487P	10:44024549	0.0399	0.00545	2.70E-13

ZNF493	19:21646167	-0.0969	0.00411	6.80E-117
ZNF501	3:44762830	-0.0511	0.00674	3.83E-14
ZNF502	3:44762830	-0.153	0.00763	2.01E-86
ZNF506	19:19887108	0.0639	0.0107	2.59E-09
ZNF507	19:32829306	-0.0582	0.00612	2.69E-21
ZNF512	2:27848586	-0.0283	0.00365	1.01E-14
ZNF514	2:95822447	0.0919	0.0042	1.23E-101
ZNF516	18:74253644	0.0457	0.00386	6.56E-32
ZNF519	18:14133319	-0.0649	0.00737	1.71E-18
ZNF528	19:52923595	0.0817	0.00794	1.39E-24
ZNF529	19:37115575	-0.0563	0.00452	4.35E-35
ZNF532	18:56656824	-0.0449	0.00621	5.76E-13
ZNF544	19:58791213	-0.0468	0.0039	8.57E-33
ZNF547	19:57871141	-0.0374	0.00508	2.20E-13
ZNF548	19:57924420	-0.0683	0.00555	2.24E-34
ZNF550	19:58113074	-0.0506	0.00533	3.09E-21
ZNF552	19:58291847	-0.0794	0.00607	1.55E-38
ZNF555	19:2863551	-0.0567	0.00646	2.38E-18
ZNF558	19:8910658	0.0395	0.00608	8.99E-11
ZNF561	19:9757469	0.107	0.00855	4.16E-35
ZNF566	19:36999787	0.0537	0.00636	3.81E-17
ZNF577	19:52399559	0.0652	0.00705	3.08E-20
ZNF584	19:58934457	0.23	0.036	1.88E-10
ZNF586	19:58291847	-0.0667	0.00379	1.90E-67
ZNF587	19:58389384	0.149	0.00839	8.13E-69
ZNF589	3:48276077	-0.109	0.00402	5.11E-153
ZNF592	15:85357727	-0.0424	0.0035	3.39E-33
ZNF593	1:26496370	-0.0483	0.00418	1.80E-30
ZNF595	4:72048	-0.11	0.00705	7.91E-54
ZNF596	8:197042	0.0467	0.00641	3.92E-13
ZNF597	16:3488773	-0.0522	0.00412	2.64E-36
ZNF600	19:53280650	-0.143	0.0106	1.17E-40
ZNF605	12:133498857	-0.188	0.0142	3.51E-39
ZNF606	19:58484344	-0.0343	0.00482	1.34E-12
ZNF607	19:38150755	0.0318	0.00343	2.76E-20
ZNF613	19:52430739	0.049	0.00595	2.04E-16
ZNF614	19:52517520	0.0941	0.00533	9.07E-68
ZNF615	19:52500769	-0.0494	0.00569	4.72E-18
ZNF622	5:16512469	0.0252	0.00385	6.09E-11

ZNF623	8:144725063	0.0418	0.00378	3.83E-28
ZNF624	17:16587287	0.171	0.0126	3.19E-41
ZNF625	19:12274521	0.0304	0.00462	5.31E-11
ZNF638	2:71651939	0.0578	0.00294	5.78E-83
ZNF639	3:179048224	0.0595	0.006	5.60E-23
ZNF641	12:48757856	-0.227	0.00599	6.85E-278
ZNF646	16:31082025	0.0336	0.00258	3.34E-38
ZNF649	19:52402357	-0.048	0.00665	6.41E-13
ZNF664	12:124430812	0.0226	0.00249	1.64E-19
ZNF665	19:53682988	0.103	0.00715	5.40E-46
ZNF669	1:247267668	-0.0348	0.0041	3.00E-17
ZNF670	1:247227819	-0.0978	0.0101	8.64E-22
ZNF671	19:58286522	-0.0538	0.0063	1.72E-17
ZNF675	19:23870110	-0.168	0.0278	1.52E-09
ZNF677	19:53770764	0.0729	0.00867	5.48E-17
ZNF682	19:20101950	-0.136	0.0203	2.40E-11
ZNF683	1:26696636	0.0303	0.00497	1.11E-09
ZNF695	1:247134691	-0.0207	0.0032	1.18E-10
ZNF7	8:146045454	0.0208	0.00309	2.02E-11
ZNF700	19:12069427	0.1	0.0138	5.37E-13
ZNF701	19:53083020	0.19	0.0171	1.83E-28
ZNF702P	19:53497662	-0.0852	0.00865	1.08E-22
ZNF713	7:55964292	-0.233	0.0113	2.89E-90
ZNF718	4:146963	0.216	0.00601	7.47E-254
ZNF720	16:31711742	-0.079	0.00592	4.59E-40
ZNF721	4:438538	0.301	0.015	3.16E-86
ZNF737	19:20774421	0.278	0.045	6.57E-10
ZNF738	19:21518961	0.0811	0.00681	2.84E-32
ZNF740	12:53554283	-0.0622	0.00501	7.05E-35
ZNF75A	16:3364867	0.0718	0.0054	1.03E-39
ZNF76	6:35202883	0.0197	0.00287	6.81E-12
ZNF761	19:53944732	-0.0965	0.0108	5.88E-19
ZNF765	19:53916008	-0.0384	0.00651	3.81E-09
ZNF767	7:149311533	0.107	0.00406	7.91E-144
ZNF768	16:30548565	0.038	0.00435	3.35E-18
ZNF77	19:2933030	-0.0832	0.0134	5.23E-10
ZNF770	15:35272173	0.124	0.0165	6.40E-14
ZNF781	19:38141758	-0.04	0.00374	1.86E-26
ZNF792	19:35451330	-0.0337	0.00436	1.22E-14

ZNF793	19:38017173	-0.0449	0.00462	3.81E-22
ZNF80	3:113970906	-0.0783	0.00727	8.28E-27
ZNF800	7:126962488	0.027	0.00346	6.70E-15
ZNF808	19:53067279	0.261	0.0147	1.10E-68
ZNF818P	19:53713328	0.287	0.0141	7.80E-89
ZNF83	19:53134276	0.261	0.00742	2.12E-243
ZNF830	17:33260938	-0.0399	0.00425	9.56E-21
ZNF839	14:102831817	0.0285	0.00424	2.03E-11
ZNF84	12:133688942	-0.0463	0.00559	1.53E-16
ZNF85	19:21145434	0.0467	0.00586	1.83E-15
ZNF91	19:23572701	-0.167	0.0125	5.66E-40
ZNF92	7:64895194	0.119	0.00806	1.43E-48
ZNFX1	20:47849216	0.0615	0.00557	5.39E-28
ZNHIT1	7:100860636	-0.0395	0.00453	3.60E-18
ZNHIT3	17:34866915	0.0626	0.00408	4.11E-52
ZNHIT6	1:86074379	-0.0724	0.00885	3.48E-16
ZNRD1	6:29998062	-0.113	0.0103	5.85E-28
ZNRF1	16:75031931	-0.0238	0.00271	2.32E-18
ZP3	7:76036380	-0.0337	0.00401	5.96E-17
ZRANB1	10:126680467	0.102	0.00948	1.22E-26
ZRANB2	1:71596192	-0.0259	0.00373	4.44E-12
ZRANB3	2:135943900	0.037	0.00475	8.34E-15
ZSCAN18	19:58598784	0.0193	0.00318	1.41E-09
ZSCAN2	15:85170583	-0.0212	0.00316	2.13E-11
ZSCAN29	15:43617264	0.0887	0.00462	1.65E-79
ZUFSP	6:117011380	0.0388	0.00498	7.81E-15
ZXDC	3:126170695	0.0268	0.00277	5.47E-22
ZYG11B	1:53180716	0.0674	0.00481	7.90E-44
ZYX	7:143081942	0.0976	0.00586	8.82E-61
ZZEF1	17:3922255	0.0256	0.00304	4.28E-17
ZZZ3	1:78070458	0.0261	0.00423	7.33E-10

			ROSMAP (brain) eQTLs					ROSMA	
Beta	Std Error	P-value	Gene	Top eSNP Position	Beta	Std Error	P-value	Gene	Top eSNP Position
2.73	0.416	5.29E-11	AADACL4	1:121141967	-0.0391	0.00578	1.34E-11	AADACL4	1:121365903
-3.29	0.439	6.67E-14	AADAT	4:171060635	-0.124	0.0159	6.25E-15	ABCA6	17:67067705
-9.31	0.903	6.35E-25	AARD	8:117952068	0.106	0.0118	2.63E-19	ABHD10	3:111274523
8.62	0.833	4.27E-25	ABCA4	1:94525623	-0.124	0.0149	8.64E-17	ABHD17B	9:74601777
9.47	1.37	4.76E-12	ABCA6	17:67142616	0.324	0.023	4.57E-45	ABI1	10:26675370
-6.35	0.913	3.52E-12	ABCA8	17:66879927	0.506	0.07	4.88E-13	ABRA	8:107749502
-0.757	0.0681	1.05E-28	ABCC6	16:16515295	0.122	0.017	7.15E-13	AC002472.13	22:21400619
0.872	0.0682	1.97E-37	ABL2	1:179098770	0.0901	0.011	2.59E-16	AC008686.1	19:14325845
0.702	0.0933	5.31E-14	ABLIM3	5:148601243	-0.158	0.0125	1.27E-36	AC008686.1	19:13964816
-2.93	0.463	2.48E-10	AC002472.13	22:21400282	0.208	0.0133	3.94E-55	AC008686.1	19:14274633
-0.291	0.0279	1.81E-25	AC008060.7	7:155195759	0.128	0.0129	3.32E-23	AC008686.1	19:14259846
-0.585	0.0831	1.93E-12	AC009065.1	16:33048628	-0.125	0.0162	1.20E-14	AC009065.1	16:32849619
-0.272	0.0293	1.64E-20	AC010536.1	16:87764267	0.0576	0.00752	1.87E-14	AC011239.1	2:241449578
2.44	0.321	2.93E-14	AC015688.3	17:25303954	-0.213	0.00944	9.88E-113	AC011239.1	2:235165520
3.22	0.51	2.72E-10	AC021218.2	7:155753306	-0.00578	0.000875	3.96E-11	AC015688.3	17:25303954
-0.418	0.0495	3.05E-17	AC021860.1	4:38612708	-0.235	0.0344	8.41E-12	AC015688.3	17:25303954
-0.991	0.144	5.90E-12	AC022498.1	3:187898523	0.287	0.0112	8.03E-145	AC015688.3	17:25303954
-1.47	0.201	2.60E-13	AC022532.1	10:72209538	0.113	0.0149	3.35E-14	AC015688.3	17:25303954
0.272	0.0369	1.69E-13	AC104667.3	2:238499997	0.0233	0.00268	3.50E-18	AC015688.3	17:25303954
-0.149	0.0156	1.28E-21	AC135178.1	17:8277215	0.175	0.0163	6.88E-27	AC022498.1	3:187898523
-0.754	0.109	4.60E-12	AC142381.1	16:32157698	-0.141	0.0092	5.12E-53	AC022498.1	3:187898523
-0.18	0.0251	7.43E-13	ACACA	17:35845916	0.0697	0.00995	2.47E-12	AC023469.1	2:151781053
6.52	0.847	1.38E-14	ACAP2	3:195076877	-0.105	0.0136	1.16E-14	AC090186.1	8:5217941
-3.59	0.431	8.12E-17	ACBD3	1:226352498	-0.0811	0.0104	6.29E-15	AC114783.1	2:127505482
-4.21	0.677	5.02E-10	ACCS	11:44096238	0.275	0.0301	6.47E-20	AC136604.1	5:178945856
-1.79	0.229	5.43E-15	ACOT1	14:74006855	-0.75	0.0442	1.41E-64	AC140481.2	2:130853936
0.193	0.0219	1.22E-18	ACOT2	14:73972535	-0.18	0.0193	1.10E-20	AC142381.1	16:33047273
2.27	0.297	2.12E-14	ACOT4	14:74073492	0.116	0.015	1.05E-14	AC142381.1	16:33047273
-0.208	0.0315	4.02E-11	ACOT6	14:74095256	-0.144	0.0145	3.05E-23	AC142381.1	16:33047273
-0.845	0.104	4.47E-16	ACPP	3:132057834	0.179	0.0191	7.14E-21	AC142381.1	16:33047273
-0.747	0.11	1.11E-11	ACSM2A	16:20420488	0.0217	0.00306	1.33E-12	AC142381.1	16:33047273
-3.24	0.333	2.25E-22	ACTR3C	7:149963399	-0.216	0.0256	3.24E-17	AC145676.2	7:100937654
-1.39	0.176	2.84E-15	ACY3	11:67414828	0.345	0.0483	9.14E-13	AC145676.2	7:120266824
-0.124	0.0159	6.25E-15	ADAD2	16:84208891	-0.262	0.0343	2.20E-14	AC145676.2	7:100937654
0.211	0.0309	8.58E-12	ADAL	15:43629310	-0.265	0.0198	7.52E-41	ACBD3	1:226327483

3.31	0.451	2.15E-13	ADAMTS13	9:136218590	-0.172	0.0224	1.61E-14	ACCSL	11:44270522
-0.203	0.0296	6.98E-12	ADAMTS14	10:72423243	0.268	0.0236	6.93E-30	ACCSL	11:44340570
-0.725	0.0621	1.72E-31	ADAMTS18	16:77390128	-0.187	0.00905	7.46E-95	ACCSL	11:44270522
-1.32	0.104	6.52E-37	ADAT1	16:75572713	0.117	0.0156	6.38E-14	ACOT1	14:73972289
-1.01	0.157	1.25E-10	ADCY4	14:24775846	-0.22	0.0323	9.68E-12	ACP1	2:100182584
-0.219	0.0309	1.37E-12	ADHFE1	8:67378564	0.248	0.0331	6.76E-14	ACSF3	16:90158184
-2.28	0.214	1.67E-26	ADORA2B	17:15876655	-0.228	0.0311	2.28E-13	ACSF3	16:90158184
1.76	0.177	2.69E-23	AEBP1	7:44143124	-0.447	0.0517	5.33E-18	ADAM2	8:39233892
3.61	0.413	2.31E-18	AGA	4:178363378	0.181	0.0182	2.65E-23	ADAMTS18	16:77392867
4.98	0.726	6.91E-12	AGAP10	10:48235892	-0.592	0.0307	7.41E-83	ADAMTS18	16:77390996
-0.364	0.0477	2.33E-14	AGAP4	10:46320813	-0.799	0.0275	1.35E-185	ADRB3	8:3881249
0.203	0.0327	5.37E-10	AGAP7	10:51346756	1.26	0.0613	6.99E-94	AFMID	17:75535140
0.242	0.0341	1.28E-12	AGAP9	10:48235892	-0.419	0.0378	1.49E-28	AGAP10	10:48235892
-0.14	0.0218	1.34E-10	AHI1	6:135623833	-0.306	0.0329	1.39E-20	AGAP4	10:46308566
0.217	0.024	1.54E-19	AHSP	16:32105098	1.5	0.205	2.53E-13	AGAP4	10:46320813
-0.234	0.0321	3.11E-13	AIDA	1:222825088	0.149	0.0168	7.38E-19	AGAP4	10:46320813
-0.155	0.0244	2.12E-10	AIFM2	10:71900246	-0.184	0.0159	5.69E-31	AGAP7	10:51223750
-1.57	0.244	1.24E-10	AK5	1:78025230	-0.187	0.0257	3.43E-13	AGAP7	10:51570152
0.508	0.0798	1.94E-10	AK8	9:135713638	0.184	0.0269	7.91E-12	AGAP7	10:51541635
-1.21	0.189	1.53E-10	AKR1C1	10:4982779	0.344	0.0371	1.82E-20	AGBL3	7:135017101
-2.19	0.33	3.22E-11	AKR1CL1	10:42602860	-0.204	0.0284	6.81E-13	AGMAT	1:149594238
1.58	0.235	1.78E-11	AL049840.1	14:104083434	0.083	0.0107	8.70E-15	AHR	7:17247167
2.37	0.38	4.46E-10	AL050302.1	21:14472722	-0.324	0.0257	1.93E-36	AHR	7:18284237
-2.03	0.299	1.13E-11	AL139099.1	14:50066542	-0.108	0.0139	7.86E-15	AHSP	16:32138743
-0.622	0.0958	8.43E-11	AL353791.1	9:40585357	0.152	0.0193	3.39E-15	AICDA	12:85427047
-3.3	0.509	8.97E-11	AL445665.1	9:70570952	0.508	0.0185	5.36E-166	AICDA	12:78330
-4.3	0.661	7.75E-11	AL590822.1	1:121113600	-0.826	0.107	1.17E-14	AIPL1	17:55555888
-0.193	0.0277	3.23E-12	AL592284.1	1:144521099	-0.197	0.0166	1.75E-32	AIPL1	17:62990309
0.728	0.0458	6.84E-57	ALDH8A1	6:135320063	-0.315	0.0194	2.76E-59	AKIP1	11:94860472
1.11	0.134	1.20E-16	ALG1L	3:125733081	-0.469	0.0296	1.53E-56	AKIP1	11:89709864
1.43	0.199	6.68E-13	ALLC	2:3624799	0.679	0.017	<1.0E-314	AKR1CL1	10:42602860
1.15	0.142	5.56E-16	ALOX12	17:6903944	-0.0411	0.00578	1.15E-12	AKR1CL1	10:42602860
-0.652	0.104	3.63E-10	ALPK3	15:85264067	0.0579	0.00846	7.70E-12	AKR1CL1	10:42602860
-0.853	0.0592	4.56E-47	ALS2CL	3:46719486	0.218	0.0235	1.75E-20	AKR1CL1	10:42602860
-0.713	0.107	2.67E-11	AMACR	5:34004707	0.228	0.0177	5.73E-38	AKR1CL1	10:42602860
-0.763	0.113	1.46E-11	ANAPC4	4:25374371	-0.104	0.0151	5.68E-12	AKT1	14:106128340
-4.38	0.474	2.45E-20	ANGPTL7	1:11088781	0.243	0.0187	1.31E-38	AKT1	14:106128340
3.38	0.386	2.01E-18	ANKK1	11:113187949	0.214	0.0144	5.89E-50	AL050302.1	21:14472722
5.29	0.662	1.34E-15	ANKRD20A4	9:69476094	0.498	0.0203	6.73E-133	AL050302.1	21:14472722

7.85	1.24	2.44E-10	ANKRD30BL	2:133018360	-0.415	0.0451	3.52E-20	AL050302.1	21:14566779
0.362	0.052	3.37E-12	ANKRD36	2:98166631	0.163	0.0231	1.71E-12	AL138764.1	10:135460942
0.174	0.0266	6.10E-11	ANKS1A	6:35009196	-0.105	0.0143	2.09E-13	AL138764.1	10:135474118
0.159	0.0203	4.78E-15	ANKS3	16:46483083	-2.15	0.314	7.53E-12	AL138764.1	10:135456218
0.532	0.0629	2.72E-17	ANO7	2:242157087	0.423	0.0236	7.70E-72	AL354898.1	9:133003291
0.398	0.0317	3.72E-36	ANXA9	1:150945607	-0.0959	0.0112	1.10E-17	AL354898.1	9:133038642
0.769	0.0966	1.71E-15	AP000350.4	22:24266726	0.125	0.00757	2.98E-61	AL354993.1	20:51661874
-0.194	0.0268	4.53E-13	AP003068.23	11:64944182	-0.243	0.0153	8.40E-57	AL354993.1	20:51661874
3.1	0.475	6.74E-11	AP3D1	19:2106333	-0.142	0.0141	7.43E-24	AL359878.1	10:17859923
-2.15	0.205	9.83E-26	APOA1BP	1:156552711	-0.186	0.0164	8.18E-30	AL359878.1	10:18111461
-1.64	0.0669	1.04E-132	APOL2	22:36635756	-0.141	0.0213	3.60E-11	AL445665.1	9:70570952
-2.3	0.234	8.44E-23	ARHGAP25	2:68962137	-0.221	0.0185	6.82E-33	AL445665.1	9:70570952
-2.35	0.295	1.64E-15	ARHGAP26	5:142604421	-0.129	0.0181	1.03E-12	AL445665.1	9:68777951
-2.26	0.199	6.86E-30	ARHGEF16	1:3353890	-0.233	0.0357	6.73E-11	AL445989.1	13:65314986
-3.42	0.283	1.27E-33	ARHGEF3	3:56767673	0.206	0.0293	2.05E-12	AL590822.1	1:121113600
0.543	0.0704	1.23E-14	ARHGEF35	7:143870422	-0.0582	0.00657	8.11E-19	AL590822.1	1:121113600
-5.44	0.862	2.77E-10	ARID3C	9:34623718	0.125	0.0148	3.02E-17	AL590822.1	1:121113600
-0.79	0.0989	1.37E-15	ARL16	17:79649515	-0.255	0.0262	2.18E-22	AL590822.1	1:145247557
1.51	0.168	2.52E-19	ARL17A	17:44173215	0.503	0.0343	1.08E-48	AL590822.1	1:121113600
2.02	0.27	7.35E-14	ARL17B	17:44443036	-0.461	0.0353	5.61E-39	ALG1L	3:125765603
-0.804	0.126	1.76E-10	ARL8B	3:5201615	-0.122	0.0115	2.72E-26	ALLC	2:3624799
0.234	0.0298	4.08E-15	ARSB	5:78160933	-0.0859	0.0122	1.91E-12	ALLC	2:3624799
-0.271	0.0336	7.30E-16	ART4	12:158028	-0.208	0.0309	1.68E-11	ALLC	2:3624799
-0.625	0.0975	1.45E-10	ART5	11:3663220	0.0979	0.00967	4.32E-24	ALPI	2:233291956
-0.254	0.0364	2.99E-12	ARTN	1:44394983	-0.185	0.0206	2.69E-19	ALPK2	18:56311186
-0.793	0.0641	3.74E-35	AS3MT	10:104611764	-0.268	0.0253	3.22E-26	AMACR	5:34004707
1.11	0.166	2.28E-11	ASB1	2:239345394	-0.205	0.0212	4.05E-22	ANKLE2	12:133833832
-1.45	0.23	2.89E-10	ASB5	4:177155788	0.0585	0.00854	7.38E-12	ANKRD20A4	9:69476094
1.72	0.226	2.73E-14	ASB8	12:48548342	0.0848	0.0128	3.47E-11	ANKRD20A4	9:69482227
7.86	0.975	7.53E-16	ASRGL1	11:62162389	0.27	0.0337	1.13E-15	ANKS3	16:46465847
1.04	0.138	4.84E-14	ATF6	1:161926569	-0.117	0.0104	2.32E-29	ANKS3	16:46483083
-0.285	0.0439	8.47E-11	ATG16L1	2:234207828	-0.133	0.0158	3.84E-17	ANKS3	16:46483083
-1.18	0.182	8.96E-11	ATP10B	5:160244938	-0.207	0.0255	4.75E-16	ANKS3	16:46483083
0.843	0.121	3.24E-12	ATP5S	14:50783898	-0.181	0.0175	4.51E-25	ANKS3	16:46483083
-6.31	0.964	5.92E-11	ATP6V0B	1:44443874	-0.437	0.0394	1.38E-28	AP3D1	19:24531262
0.19	0.0238	1.43E-15	ATP6V1E2	2:46760263	-0.121	0.0152	1.71E-15	AP3D1	19:27829622
2.51	0.394	1.88E-10	ATRIP	3:48487795	-0.115	0.0161	9.14E-13	AP3D1	19:27829622
0.384	0.0501	1.79E-14	ATRN	20:3622772	-0.0974	0.0126	1.07E-14	APBA3	19:35742760
-2.33	0.371	3.38E-10	ATXN7L3B	12:74934945	-0.207	0.0174	1.23E-32	APOF	12:56657216

0.163	0.0216	4.48E-14	AVP	20:25835641	-2.83	0.286	4.37E-23	APOF	12:56747778
-0.6	0.0768	5.61E-15	B3GNTL1	17:81009636	-0.234	0.03	6.19E-15	APOH	17:63933653
-0.287	0.0231	1.93E-35	B4GALT7	5:177032050	-0.201	0.0178	1.44E-29	APOH	17:64405875
-2.32	0.306	3.41E-14	BAALC	8:104129424	0.14	0.0209	2.10E-11	AQP10	1:154835734
0.563	0.0846	2.84E-11	BAAT	9:104146951	-0.156	0.0175	4.91E-19	AQP10	1:15509852
0.17	0.0202	3.90E-17	BATF3	1:212873074	0.244	0.0351	3.61E-12	AQP10	1:154835734
-1.73	0.273	2.34E-10	BBS9	7:33194826	-0.093	0.0142	5.78E-11	ARL17B	17:44409787
-0.387	0.0549	1.80E-12	BCR	22:23652201	-0.239	0.0176	5.30E-42	ARMC10	7:102217297
-0.221	0.0303	3.01E-13	BHLHA9	17:21526192	-1.58	0.209	4.04E-14	ARMC10	7:102217297
-0.599	0.0405	1.70E-49	BHMT	5:78411324	-0.144	0.0123	1.17E-31	ART4	12:158028
-0.846	0.126	1.89E-11	BIN3	8:22525980	-0.129	0.0148	2.88E-18	ART4	12:158028
1.25	0.116	4.48E-27	BIRC7	20:61870075	0.108	0.0134	7.65E-16	ART4	12:158028
1.26	0.192	5.29E-11	BIVM	13:103477485	0.0737	0.0109	1.37E-11	ART4	12:158028
-0.192	0.0157	2.17E-34	BLMH	17:28588401	-0.104	0.0103	5.69E-24	AS3MT	10:104623053
-0.432	0.0536	7.65E-16	BLOC1S2	10:101908193	-0.337	0.0319	4.36E-26	ASB16	17:41382047
-0.313	0.0451	3.92E-12	BLVRA	7:43823125	-0.0884	0.0131	1.50E-11	ASB16	17:41463753
0.557	0.0637	2.25E-18	BNIP1	5:172591337	-0.152	0.018	3.05E-17	ATG14	14:56654212
-0.256	0.041	4.27E-10	BPHL	6:3138194	0.165	0.0184	3.04E-19	ATXN7L3B	12:75271460
0.35	0.0495	1.54E-12	BSND	1:55468040	0.0241	0.00283	1.65E-17	ATXN7L3B	12:74934945
-1.34	0.135	3.21E-23	BSPRY	9:116085602	0.237	0.0152	8.24E-55	AUH	9:93180398
-1.07	0.0437	2.13E-132	BTBD1	15:83792713	-0.214	0.0171	6.21E-36	AVP	20:25835641
-0.89	0.135	4.32E-11	BTN2A2	6:26384669	0.189	0.0266	1.20E-12	AVP	20:25835641
-1.25	0.2	4.10E-10	BTNL3	5:180430797	0.0846	0.00413	2.97E-93	AVP	20:25835641
-2.2	0.191	1.07E-30	BTNL9	5:180485949	0.256	0.0378	1.27E-11	AVP	20:25835641
-1.14	0.135	3.05E-17	BX255923.1	9:68711007	-0.262	0.0399	5.15E-11	AVP	20:25835641
-0.893	0.133	1.89E-11	C10orf107	10:63422876	0.434	0.0282	1.91E-53	BCCIP	10:127583983
-0.756	0.0463	6.21E-60	C10orf82	10:118429887	-0.106	0.0137	1.02E-14	BCR	22:23660893
-1.24	0.192	1.06E-10	C12orf60	12:14965332	-0.152	0.0131	3.98E-31	BCR	22:23661565
-1.02	0.132	1.10E-14	C12orf73	12:104385191	-0.155	0.021	1.57E-13	BHLHA9	17:21526192
-0.521	0.0808	1.13E-10	C14orf166	14:52471263	-0.218	0.0259	3.86E-17	BHLHA9	17:21526192
-0.408	0.0621	5.03E-11	C14orf178	14:78124797	0.138	0.0173	1.50E-15	BHLHA9	17:21526192
-0.185	0.0212	2.63E-18	C15orf38	15:90448376	0.11	0.0171	1.25E-10	BHLHA9	17:21526192
-0.42	0.0643	6.49E-11	C15orf57	15:40930449	0.115	0.0125	3.58E-20	BHLHA9	17:21526192
-0.312	0.0458	9.61E-12	C16orf3	16:90103087	0.0154	0.00225	7.68E-12	BHLHE23	20:61925237
-0.529	0.0354	1.72E-50	C16orf59	16:33941694	-2.8	0.357	4.39E-15	BLOC1S2	10:101908193
-0.732	0.108	1.22E-11	C16orf93	16:30747191	-0.212	0.0274	1.02E-14	BLOC1S5	6:7457640
-0.14	0.0207	1.35E-11	C17orf50	17:34091078	0.0999	0.0143	2.83E-12	BLOC1S5	6:90595698
-10.5	1.42	1.42E-13	C17orf67	17:54911028	0.332	0.031	9.17E-27	BPIFC	22:32894025
-0.941	0.0924	2.34E-24	C17orf70	17:79506890	-0.168	0.0232	4.44E-13	BPIFC	22:32894025

1.06	0.0918	7.66E-31	C19orf53	19:13884469	-0.138	0.021	4.98E-11	BPNT1	1:220133673
1.16	0.124	8.37E-21	C19orf83	19:46145025	-0.054	0.00817	3.85E-11	BTNL3	5:180430797
9	1.38	6.95E-11	C19orf84	19:51894327	0.0872	0.00985	8.54E-19	BTNL3	5:180455372
-5.85	0.722	5.38E-16	C1orf194	1:109645420	-0.439	0.0559	4.05E-15	BVES	6:104902232
-0.136	0.0207	5.03E-11	C1orf195	1:145312917	0.156	0.0192	4.47E-16	BX255923.1	9:68711007
-0.22	0.0319	5.33E-12	C1orf204	1:159823363	-0.136	0.0171	1.82E-15	C14orf1	14:75861238
0.636	0.0917	4.04E-12	C1QTNF6	22:37580334	-0.238	0.027	1.20E-18	C14orf178	14:78156031
-2.65	0.318	7.86E-17	C1QTNF9B	13:24514619	0.3	0.0268	4.36E-29	C14orf178	14:78121942
2.28	0.324	1.96E-12	C21orf128	21:43519470	0.331	0.0213	1.86E-54	C16orf59	16:33941694
-0.707	0.114	5.58E-10	C22orf34	22:50033362	0.21	0.0217	3.76E-22	C16orf59	16:33941694
-3.22	0.422	2.34E-14	C2orf27B	2:133011215	-0.432	0.0644	1.97E-11	C16orf59	16:33941694
2.96	0.413	7.66E-13	C2orf40	2:106687456	-0.261	0.0293	5.20E-19	C16orf59	16:33991563
1.42	0.106	6.36E-41	C2orf74	2:61379625	0.469	0.0165	1.01E-177	C16orf59	16:33991563
-0.126	0.0178	1.46E-12	C2orf82	2:233735693	0.612	0.0426	8.43E-47	C16orf87	16:46402985
-0.402	0.0424	2.52E-21	C4orf6	4:49242504	-0.121	0.0158	1.89E-14	C16orf87	16:46402985
-3.57	0.488	2.56E-13	C5orf17	5:24091732	0.304	0.0224	5.92E-42	C16orf87	16:46402985
-5.85	0.877	2.55E-11	C6orf195	6:2614822	0.046	0.00533	6.11E-18	C16orf92	16:29065793
-0.371	0.0403	3.39E-20	C6orf229	6:25647375	-0.4	0.0568	1.89E-12	C16orf92	16:29065793
0.246	0.0306	9.04E-16	C6orf57	6:71291014	-0.224	0.0347	1.08E-10	C17orf105	17:41382047
1.05	0.132	1.80E-15	C7orf13	7:156429648	-0.267	0.0145	1.02E-75	C17orf61-PLSCR3	17:81131315
0.932	0.123	3.53E-14	C7orf73	7:135349273	0.151	0.0206	2.30E-13	C17orf80	17:71333186
0.25	0.036	3.80E-12	C8orf31	8:144125300	0.258	0.0344	6.38E-14	C1orf100	1:245201465
-0.613	0.0952	1.20E-10	C8orf58	8:22457804	0.109	0.0162	1.72E-11	C1orf174	1:3578444
-0.303	0.0346	2.00E-18	C9orf142	9:139883948	0.147	0.0228	1.14E-10	C1orf195	1:148849427
-0.245	0.028	2.13E-18	C9orf152	9:113007289	-0.0295	0.00372	2.19E-15	C1orf195	1:148849427
-0.271	0.0294	3.04E-20	C9orf72	9:27565936	-0.148	0.0177	6.19E-17	C1orf195	1:148849427
0.296	0.0451	5.27E-11	C9orf89	9:95875575	0.168	0.0186	1.68E-19	C1orf195	1:148849427
-0.794	0.106	6.86E-14	CA12	15:63682331	0.817	0.0648	1.91E-36	C1orf195	1:149024078
-0.181	0.0284	1.85E-10	CA5A	16:87935184	0.107	0.0145	1.59E-13	C20orf196	20:59849622
0.221	0.0312	1.41E-12	CA9	9:35884027	-2.02	0.314	1.25E-10	C21orf128	21:43526509
-1.49	0.175	1.68E-17	CAB39L	13:49945410	0.214	0.0193	1.43E-28	C21orf128	21:43529428
1.63	0.169	5.16E-22	CABYR	18:21760784	0.141	0.022	1.46E-10	C2orf27B	2:132797395
2.44	0.369	3.78E-11	CACNG6	19:54514785	0.148	0.0148	1.52E-23	C2orf27B	2:133015374
-0.257	0.0311	1.41E-16	CAMLG	5:134005332	0.444	0.0282	7.47E-56	C2orf27B	2:133018682
2.56	0.412	5.18E-10	CAPN11	6:44134581	0.0459	0.00613	7.01E-14	C2orf27B	2:133015374
-0.615	0.0831	1.35E-13	CAPN8	1:223812933	-0.154	0.0169	8.06E-20	C2orf27B	2:133021477
0.269	0.0416	1.00E-10	CAPN9	1:230891248	0.161	0.015	7.10E-27	C2orf74	2:61400816
-0.134	0.0145	2.43E-20	CASP16	16:34015836	0.321	0.0449	8.73E-13	C2orf74	2:61385100
-4.21	0.519	4.99E-16	CASQ1	1:160162959	-0.329	0.0251	2.98E-39	C2orf82	2:233735693

-4.56	0.359	5.77E-37	CAT	11:34459717	0.185	0.0285	8.51E-11	C3orf65	3:186339617
5.35	0.364	6.66E-49	CBLN3	14:24899473	0.544	0.0314	3.06E-67	C3orf65	3:186339617
3.23	0.271	9.44E-33	CBR3	21:37513596	-0.183	0.028	6.33E-11	C4orf6	4:49242504
-1.77	0.281	3.00E-10	CBWD2	2:114207406	-0.185	0.0165	3.56E-29	C4orf6	4:49242504
7.31	0.774	3.57E-21	CCBL2	1:89426277	-0.301	0.0223	1.61E-41	C4orf6	4:49242504
-0.264	0.0309	1.30E-17	CCDC103	17:42916399	0.27	0.0286	3.71E-21	C4orf6	4:49242504
-0.463	0.0712	7.88E-11	CCDC110	4:186401223	-0.144	0.0191	4.73E-14	C4orf6	4:49242504
2.18	0.301	4.40E-13	CCDC13	3:42814668	0.174	0.0205	2.11E-17	C6orf201	6:45545843
-2.18	0.301	4.40E-13	CCDC144NL	17:20827039	0.0894	0.00939	1.72E-21	C6orf229	6:25647375
0.165	0.0214	1.26E-14	CCDC163P	1:45969036	-0.576	0.0225	1.53E-144	C6orf229	6:25647375
-0.124	0.0199	4.63E-10	CCDC169-SOHLH	13:36802672	0.016	0.00198	6.43E-16	C6orf229	6:25647375
-0.162	0.0202	1.06E-15	CCDC170	6:151815864	0.123	0.019	9.56E-11	C6orf229	6:25647375
0.554	0.0609	9.30E-20	CCDC173	2:170463156	-0.621	0.0304	9.50E-93	C6orf229	6:25647375
0.183	0.0265	5.00E-12	CCDC181	1:169373833	-0.203	0.0268	3.60E-14	C6orf47	6:31421567
-0.597	0.0778	1.67E-14	CCDC23	1:43233484	0.2	0.0186	5.76E-27	C7orf13	7:156429648
-0.12	0.0184	6.95E-11	CCDC25	8:27617390	0.144	0.0208	4.42E-12	C7orf34	7:142459962
0.473	0.0622	2.86E-14	CCDC40	17:78021037	0.442	0.0208	3.30E-100	C7orf34	7:142461940
-0.721	0.109	3.72E-11	CCDC66	3:56579538	-0.133	0.0146	8.27E-20	C8orf86	8:38470006
-0.383	0.0528	4.05E-13	CCDC8	19:46938483	-0.17	0.0229	1.14E-13	C9orf171	9:136370017
-2.16	0.314	6.03E-12	CCDC82	11:96108105	0.177	0.0227	6.32E-15	CACTIN	19:27866520
-0.489	0.0415	4.77E-32	CCL3L3	17:34728537	0.906	0.0404	2.21E-111	CACTIN	19:27866520
-0.947	0.145	6.53E-11	CCNO	5:54498792	-0.318	0.0395	8.24E-16	CACTIN	19:27866520
-1.36	0.215	2.52E-10	CCZ1	7:6884764	0.324	0.0143	1.18E-113	CADM2	3:84159305
0.71	0.109	7.33E-11	CD274	9:5460801	-0.0942	0.014	1.71E-11	CALML5	10:46946870
1.05	0.163	1.18E-10	CD52	1:26645666	-0.483	0.0477	4.25E-24	CAMLG	5:134005332
-0.693	0.0898	1.19E-14	CD6	11:60776209	-0.31	0.0137	2.31E-113	CAMLG	5:133916719
-0.541	0.0263	5.06E-94	CD82	11:44626461	0.238	0.0365	7.01E-11	CASP16	16:34015836
-0.637	0.0812	4.34E-15	CDA	1:20934283	0.349	0.0341	1.39E-24	CASP16	16:34015836
0.315	0.0433	3.47E-13	CDC25B	20:3792226	0.0874	0.0128	8.60E-12	CASP16	16:34015836
5.08	0.673	4.41E-14	CDC37L1	9:45769289	1.24	0.186	2.62E-11	CASP16	16:34015836
2.8	0.366	2.01E-14	CDC7	1:92021650	-0.137	0.0162	2.75E-17	CASP16	16:34015836
-3.4	0.493	5.33E-12	CDH9	5:26978780	-0.228	0.0257	7.21E-19	CBLN3	14:24899473
-0.353	0.0545	9.35E-11	CDHR1	10:85954220	0.331	0.0168	2.06E-86	CBWD2	2:114264918
-0.199	0.0311	1.57E-10	CDHR3	7:105545505	0.126	0.0162	7.38E-15	CCDC163P	1:45976587
-0.528	0.034	2.19E-54	CDK10	16:89744756	0.188	0.0257	2.57E-13	CCDC163P	1:45969036
-0.676	0.101	2.19E-11	CDK13	7:40060751	0.0872	0.0135	1.05E-10	CCDC163P	1:45999773
0.62	0.0997	5.01E-10	CDK19	6:111038433	0.132	0.0174	3.29E-14	CCDC163P	1:45976263
-2.22	0.343	9.65E-11	CDON	11:125843140	0.165	0.0106	1.24E-54	CCDC173	2:170528009
-1.31	0.149	1.47E-18	CEACAM19	19:45165912	0.261	0.0329	2.14E-15	CCDC173	2:170519369

-0.878	0.0474	1.34E-76	CELA2A	1:149026684	0.193	0.0279	4.59E-12	CCDC40	17:78021037
-0.909	0.136	2.33E-11	CELA2B	1:149735408	-0.232	0.0316	2.11E-13	CCDC40	17:78007062
-1.47	0.229	1.37E-10	CENPM	22:42333330	0.304	0.0275	2.08E-28	CCDC71L	7:106538478
-1.5	0.139	3.78E-27	CENPP	9:94966486	0.211	0.0108	5.32E-85	CCDC79	16:65792665
0.393	0.0579	1.14E-11	CENPV	17:16248906	0.179	0.0218	2.19E-16	CCDC79	16:66080024
-1.08	0.149	4.22E-13	CEP19	3:196433623	-0.139	0.0194	7.78E-13	CCL20	2:229374659
-0.508	0.0489	2.80E-25	CEP250	20:34111861	-0.112	0.016	2.56E-12	CCL20	2:228463878
-1.12	0.156	7.00E-13	CERS5	12:50570127	-0.163	0.0182	3.37E-19	CCL22	16:57937788
-0.974	0.146	2.54E-11	CES1	16:55866900	0.586	0.0339	5.98E-67	CCL25	19:81769
-2.31	0.203	5.30E-30	CGB2	19:49550077	0.059	0.0085	3.89E-12	CCL3	17:34591375
-0.15	0.0219	7.42E-12	CGB8	19:49558509	0.0501	0.00461	1.64E-27	CCL3L3	17:34729655
-0.969	0.138	2.19E-12	CHAD	17:48553916	0.457	0.0262	3.91E-68	CCL3L3	17:34729655
-0.346	0.0409	2.68E-17	CHAF1A	19:4393294	-0.173	0.0189	5.51E-20	CCL4	17:34762804
-0.683	0.103	3.33E-11	CHCHD2	7:56155219	-0.129	0.0145	5.76E-19	CCL8	17:3188133
-0.857	0.137	3.96E-10	CHIT1	1:203189634	-0.203	0.0239	2.00E-17	CCR4	3:33130780
-0.225	0.0302	9.31E-14	CHKB-CPT1B	22:51021599	0.141	0.0214	4.43E-11	CCZ1	7:6884764
2.22	0.356	4.49E-10	CHRM5	15:34276895	0.264	0.0151	1.92E-68	CCZ1	7:6877519
-5.6	0.772	4.05E-13	CHRNA1	2:175615590	0.11	0.0118	1.14E-20	CD274	9:4537048
-6.48	0.837	9.79E-15	CHRNA5	15:78842239	-0.185	0.0101	6.07E-75	CD34	1:207904445
-0.323	0.0434	9.89E-14	CHRNE	17:4802317	0.307	0.0153	1.48E-89	CD48	1:160667203
2.14	0.19	1.99E-29	CHST12	7:152104360	1.54	0.221	3.21E-12	CD48	1:160667203
0.166	0.0228	3.32E-13	CHST13	3:126242669	-0.0767	0.01	1.72E-14	CD48	1:160666685
-0.486	0.0592	2.22E-16	CIB2	15:78423596	0.245	0.0196	7.47E-36	CD48	1:160667203
0.174	0.021	1.17E-16	CINP	14:102809559	-0.188	0.0149	1.69E-36	CD6	11:60776781
-1.48	0.0782	6.99E-80	CISD2	4:103685471	-0.125	0.0117	1.21E-26	CD6	11:60776781
-7.04	1.06	3.10E-11	CKS2	9:91926088	-0.67	0.0596	2.55E-29	CD6	11:60776209
0.497	0.0647	1.57E-14	CLDN9	16:3059480	-0.274	0.0277	4.52E-23	CD68	17:72248980
0.56	0.0873	1.41E-10	CLEC18A	16:70115781	-0.185	0.0223	1.08E-16	CD7	17:79317035
-0.306	0.0344	5.82E-19	CLEC18C	16:70077720	0.189	0.0169	4.92E-29	CDA	1:20934283
-7.68	0.951	6.71E-16	CLHC1	2:55427398	0.136	0.0212	1.41E-10	CDC37L1	9:45769289
6.63	0.746	6.26E-19	CLPSL1	6:35764570	0.294	0.0298	5.86E-23	CDC37L1	9:45769289
8.94	1.19	5.80E-14	CLPSL2	6:35744303	0.125	0.0174	6.77E-13	CDC37L1	9:45769289
-1.52	0.188	6.21E-16	CLTCL1	22:19277809	0.474	0.0361	2.21E-39	CDC42	1:21790318
2.11	0.2	5.08E-26	CNDP2	18:72164097	0.0999	0.0129	9.62E-15	CDC42	1:21790318
-0.65	0.0805	6.77E-16	CNGA1	4:48013316	0.122	0.0121	6.59E-24	CDC42	1:21790318
2.52	0.38	3.32E-11	CNR2	1:248601069	-0.0712	0.00917	8.20E-15	CDCP1	3:45194509
-1.22	0.123	3.45E-23	COA6	1:234507977	0.141	0.0153	3.09E-20	CDH16	16:66348814
-1.94	0.295	4.82E-11	COCH	14:31343494	0.184	0.0229	9.36E-16	CDH16	16:66348814
-3.2	0.411	6.92E-15	COL18A1	21:46825723	0.168	0.0235	8.75E-13	CDHR1	10:85954220

-6.43	0.978	4.88E-11	COL24A1	1:86619342	0.256	0.0324	2.76E-15	CDHR1	10:85954220
-0.388	0.0607	1.64E-10	COL28A1	7:7572215	0.186	0.0181	9.02E-25	CDKAL1	6:22176923
-2.38	0.341	2.96E-12	COL6A1	21:47419012	0.205	0.0218	5.27E-21	CELA2A	1:148671016
0.172	0.0255	1.53E-11	COMTD1	10:76962774	-0.285	0.0433	4.64E-11	CELA2A	1:149026684
0.346	0.0411	3.81E-17	COQ5	12:120930798	0.0779	0.0113	5.43E-12	CELA2A	1:149026684
0.3	0.0313	9.28E-22	CORO7	16:4460049	0.195	0.021	1.61E-20	CELA2A	1:149026684
-2.13	0.281	3.45E-14	CORO7-PAM16	16:33941694	-3.14	0.402	5.68E-15	CELA2A	1:149026684
2.39	0.295	5.42E-16	CPT1B	22:51017082	0.207	0.0265	5.66E-15	CELA2B	1:148671016
-1.1	0.175	3.26E-10	CPVL	7:29188870	-0.302	0.0331	7.25E-20	CELA2B	1:149735408
-0.329	0.0209	7.84E-56	CR1	1:207684192	-0.096	0.00692	9.25E-44	CELA2B	1:149735408
6.78	1.04	7.07E-11	CRABP1	15:78633870	0.326	0.0321	3.12E-24	CELA2B	1:149735408
0.448	0.0681	4.75E-11	CRIPT	2:46858321	0.135	0.0129	1.25E-25	CELA2B	1:149735408
-0.769	0.0977	3.52E-15	CRLF3	17:29064135	-0.208	0.0241	6.10E-18	CENPL	1:173077158
-1.81	0.253	8.42E-13	CRYBG3	3:97495510	-0.133	0.0189	1.96E-12	CENPP	9:95117419
-2.61	0.335	6.65E-15	CRYGA	2:209030050	0.155	0.0155	1.52E-23	CENPP	9:94966486
3.24	0.376	6.87E-18	CRYGD	2:208985903	0.134	0.0203	4.08E-11	CEP104	1:3577254
5.52	0.755	2.65E-13	CRYZ	1:75167477	-0.532	0.0565	4.69E-21	CHAD	17:48553916
-2.29	0.242	3.00E-21	CSGALNACT1	8:19315034	0.206	0.0186	1.65E-28	CHMP1B	18:11882
2.61	0.307	1.87E-17	CSGALNACT2	10:43640013	-0.206	0.0176	1.21E-31	CHP1	15:40526383
3.16	0.458	5.22E-12	CSRP2	12:77437382	-0.197	0.0264	8.51E-14	CHP1	15:40531147
4.07	0.653	4.58E-10	CTAGE8	7:143952243	-0.0321	0.00493	7.46E-11	CHRNA5	15:78843051
-0.0992	0.0158	3.42E-10	CTD-2162K18.4	19:37263878	-0.158	0.0186	1.99E-17	CHRNB1	17:80989217
0.314	0.0445	1.71E-12	CTD-2210P24.4	11:45830198	-0.252	0.0361	2.94E-12	CHRNE	17:4770443
0.215	0.0317	1.18E-11	CTD-2368P22.1	19:58513807	0.196	0.0208	4.38E-21	CHST12	7:152104360
-3.67	0.56	5.62E-11	CTDNEP1	17:7145981	-0.0909	0.0135	1.66E-11	CHST12	7:152104360
0.248	0.0248	1.52E-23	CTIF	18:46384073	-0.229	0.029	2.87E-15	CHST12	7:152104360
-0.371	0.032	4.43E-31	CTNS	17:3564687	-0.241	0.02	1.94E-33	CHST12	7:152104360
-0.459	0.0618	1.11E-13	CTRB1	16:75268120	-0.0617	0.00669	2.90E-20	CHST12	7:152104360
0.139	0.0206	1.50E-11	CTSH	15:79263141	0.376	0.0557	1.47E-11	CIAO1	2:96308288
0.446	0.0579	1.33E-14	CWH43	4:49225999	-0.247	0.019	1.22E-38	CIAO1	2:97202490
-0.443	0.0597	1.17E-13	CYB561	17:61518005	-0.436	0.0194	7.42E-112	CLEC5A	7:142143328
0.156	0.0217	6.53E-13	CYP21A2	6:31914935	0.247	0.0348	1.27E-12	CNEP1R1	16:50241875
-3.23	0.498	8.82E-11	CYP24A1	20:52789743	-0.0813	0.00887	4.92E-20	CNEP1R1	16:49558966
-1.68	0.18	1.03E-20	CYP2D6	22:42485671	0.479	0.0233	6.53E-94	CNOT2	12:69986
-0.411	0.0445	2.56E-20	CYP4B1	1:47308949	-0.152	0.0231	4.70E-11	CNOT2	12:69986
-3.56	0.545	6.48E-11	CYSTM1	5:139543379	-0.155	0.0237	6.15E-11	CNR2	1:248601069
-0.298	0.0441	1.41E-11	DBH	9:136500515	0.217	0.0157	1.89E-43	CNR2	1:235844
-2.56	0.294	3.11E-18	DCAKD	17:43128906	0.127	0.019	2.32E-11	CNR2	1:248601069
2.22	0.269	1.55E-16	DCBLD2	3:98515161	-0.098	0.0124	2.72E-15	CNTF	11:58450609

2.52	0.391	1.16E-10	DDRGK1	20:26102364	-1.76	0.213	1.42E-16	COL6A5	3:129235558
-3.82	0.482	2.28E-15	DDT	22:24266726	0.165	0.0146	1.29E-29	COL6A5	3:129235558
-2.11	0.147	1.01E-46	DDTL	22:24266954	-0.36	0.0111	9.58E-231	COPB1	11:134855811
0.179	0.0265	1.43E-11	DDX11	12:31225474	-0.594	0.0172	2.40E-261	COPZ1	12:54462997
0.774	0.0936	1.35E-16	DDX24	14:94518415	-0.166	0.025	3.14E-11	COPZ1	12:54507057
8.57	1.12	1.98E-14	DDX51	12:132610313	-0.191	0.0256	8.59E-14	COQ10B	2:19858623
-2.55	0.333	1.89E-14	DEFA5	8:7544824	0.22	0.0307	7.72E-13	CORO7-PAM16	16:33991563
3.57	0.556	1.36E-10	DEFB119	20:29617870	-0.152	0.00711	2.13E-101	CORO7-PAM16	16:33941694
0.348	0.0353	6.31E-23	DEFB121	20:29422202	-0.143	0.0112	2.48E-37	CORO7-PAM16	16:33941694
0.3	0.0363	1.40E-16	DEFB135	8:12406084	0.0909	0.0127	8.22E-13	CORO7-PAM16	16:33941694
0.763	0.0696	5.78E-28	DFFB	1:3791533	0.204	0.022	1.81E-20	CORO7-PAM16	16:33941694
1.38	0.197	2.47E-12	DFNA5	7:24749429	0.176	0.0143	8.23E-35	COX6B1	19:36635239
0.248	0.0374	3.33E-11	DHFR	5:79951133	0.419	0.0208	3.02E-90	COX6B1	19:36159808
0.579	0.076	2.57E-14	DHODH	16:72042682	-0.114	0.0151	4.36E-14	COX8A	11:64729109
-0.18	0.0253	1.12E-12	DHRS1	14:24790465	-0.168	0.022	2.23E-14	CR1L	1:207907085
0.391	0.0368	2.28E-26	DHRS11	17:34913982	-0.217	0.0299	3.94E-13	CRBN	3:24291336
-0.845	0.125	1.38E-11	DHRS4L2	14:24433294	-0.424	0.0408	2.69E-25	CRCP	7:6526147
4.14	0.666	5.09E-10	DHX33	17:5351791	-0.101	0.0111	9.11E-20	CRCP	7:6526147
-0.204	0.0323	2.69E-10	DIRC3	2:218150948	-0.0622	0.00826	5.06E-14	CRLF3	17:28843496
-0.967	0.143	1.36E-11	DNAH3	16:21203042	0.032	0.00348	3.74E-20	CSHL1	17:61018091
-0.172	0.0227	3.53E-14	DNAJC15	13:43598707	-0.273	0.0168	2.23E-59	CSHL1	17:61018091
-1.6	0.254	2.99E-10	DND1	5:140030306	-0.115	0.0157	2.39E-13	CSHL1	17:61018091
1.65	0.266	5.54E-10	DNLZ	9:139239807	0.381	0.053	6.54E-13	CSNK1D	17:80989217
-0.585	0.0849	5.56E-12	DPM2	9:130724828	-0.21	0.0227	2.22E-20	CTC-512J12.6	19:44211098
0.339	0.049	4.57E-12	DPY19L2	12:64072856	0.121	0.0182	2.96E-11	CTD-2132N18.3	17:39606109
-0.527	0.0583	1.57E-19	DPYSL5	2:27097194	-0.28	0.0188	3.63E-50	CTD-2600O9.1	16:58696420
-1.38	0.184	6.38E-14	DRAXIN	1:121113600	-1.6	0.222	5.71E-13	CTD-2600O9.1	16:58515425
-1.74	0.232	6.38E-14	DRD2	11:113566207	-0.0914	0.0136	1.81E-11	CTSG	14:25405621
11.8	1.76	2.02E-11	DSC1	18:28749716	0.0884	0.0062	3.99E-46	CTSG	14:25405621
-0.327	0.041	1.52E-15	DSCC1	8:120868138	-0.295	0.0141	3.38E-97	CTSG	14:24231601
-0.585	0.0936	4.10E-10	DST	6:57447737	2.48	0.234	3.03E-26	CWC15	11:94860472
-2.04	0.233	2.03E-18	DUSP14	17:35744215	0.171	0.0191	3.46E-19	CWC15	11:94860472
0.377	0.0421	3.40E-19	DUX4L2	4:190897129	-0.194	0.0214	1.24E-19	CWH43	4:49245680
0.424	0.0389	1.16E-27	DYDC2	10:82195219	-0.168	0.0254	3.74E-11	CWH43	4:49245680
1.11	0.174	1.78E-10	ECE2	3:183999801	-0.172	0.0206	6.85E-17	CWH43	4:49245680
-0.28	0.0411	9.58E-12	ECHDC3	10:11784320	0.252	0.0181	4.61E-44	CWH43	4:49245680
-5.51	0.25	1.19E-107	EFCAB13	17:45385012	0.118	0.0108	8.67E-28	CWH43	4:49245680
-6.11	0.709	6.83E-18	EFCAB2	1:245133662	-0.251	0.0184	2.27E-42	CXCL10	4:76024963
-2.7	0.415	7.72E-11	EFEMP1	2:56193418	-0.302	0.0466	9.13E-11	CXCL10	4:77471246

-4.07	0.377	3.60E-27	EFHB	3:19987539	0.441	0.0199	8.21E-109	CXCL3	4:75316056
-7.12	0.335	3.04E-100	EIF2A	3:150292771	0.283	0.0252	2.90E-29	CXCL5	4:7540518
-11.6	1.03	2.02E-29	EIF3C	16:28414341	0.263	0.0282	1.10E-20	CXCL5	4:74601312
-7.66	0.994	1.30E-14	EIF3M	11:32753984	0.0882	0.0124	1.14E-12	CXCL6	4:74601312
-0.618	0.0906	9.03E-12	EME1	17:48462239	-0.165	0.016	6.19E-25	CXCL6	4:74601312
0.262	0.0391	2.07E-11	ENDOG	9:131575642	0.229	0.0252	1.02E-19	CXCL9	4:7614727
-0.325	0.0307	3.45E-26	EPCAM	2:47596853	0.923	0.0317	2.21E-186	CXCL9	4:77822371
-0.896	0.129	3.76E-12	EPG5	18:43468947	-0.0646	0.00862	6.67E-14	CXCR5	11:119083378
-1.05	0.0914	1.52E-30	EPHB6	7:142562736	-0.412	0.0526	4.78E-15	CYB561	17:61518005
-1.52	0.193	3.39E-15	EPPK1	8:144953999	-0.0243	0.00318	2.15E-14	CYB561	17:61518005
1.64	0.195	4.09E-17	ERAP2	5:96252432	0.548	0.0163	8.67E-248	CYP2D6	22:42539720
4.53	0.678	2.37E-11	ERCC3	2:128015367	0.101	0.0113	3.96E-19	CYP2D6	22:42540551
-1.14	0.13	1.80E-18	ERMAP	1:43303365	-0.187	0.0188	2.60E-23	DCDC2	6:23623830
-0.567	0.0439	3.67E-38	ERV3-1	7:64427532	0.18	0.0172	1.25E-25	DDRGK1	20:26102364
-0.817	0.128	1.74E-10	ESPNL	2:239008067	0.135	0.0121	6.62E-29	DDRGK1	20:26102364
4.31	0.683	2.78E-10	ESRRB	14:76624026	0.0369	0.00552	2.31E-11	DDRGK1	20:26102364
-4.56	0.642	1.22E-12	ETFDH	4:159601676	0.187	0.0225	9.48E-17	DDRGK1	20:26102364
5.8	0.799	3.90E-13	ETS2	21:40211235	-0.141	0.0205	6.07E-12	DDRGK1	20:26102364
4.85	0.724	2.10E-11	ETV7	6:36354213	0.14	0.0148	3.10E-21	DDT	22:25056085
-0.287	0.0407	1.77E-12	EVPL	17:73985074	0.107	0.0162	3.98E-11	DDTL	22:24295217
-1.14	0.17	2.00E-11	EXOSC10	1:121113600	-4.38	0.282	2.11E-54	DDTL	22:24267047
1.97	0.308	1.59E-10	EXOSC6	16:70297568	-0.23	0.0229	9.80E-24	DDTL	22:24266954
-1.94	0.302	1.33E-10	EYA3	1:28306250	0.0804	0.0103	5.91E-15	DDX11	12:31213631
1.45	0.226	1.40E-10	F11	4:187153511	0.15	0.0152	5.71E-23	DDX11	12:31225474
-3.3	0.384	8.42E-18	F2RL1	5:76108739	-0.0566	0.0071	1.56E-15	DDX11	12:31230300
7.15	0.701	1.99E-24	F5	1:169533346	0.117	0.0177	3.84E-11	DDX19A	16:70563271
7.54	1.09	4.60E-12	FABP1	2:88436001	-0.358	0.0234	7.75E-53	DEFA1	8:7756576
-1.82	0.271	1.87E-11	FADD	11:70051966	-0.117	0.014	6.43E-17	DEFA1B	8:74106526
-0.397	0.0526	4.44E-14	FAH	15:80478587	-0.386	0.0362	1.52E-26	DEFA3	8:7756576
2.56	0.347	1.61E-13	FAIM	3:138328016	0.159	0.0172	2.37E-20	DEFA4	8:7756576
-3.17	0.42	4.43E-14	FAM118A	22:45728978	-1.46	0.0541	2.10E-160	DEFA4	8:7756550
-1.68	0.243	4.73E-12	FAM131C	1:16391056	0.145	0.0211	6.33E-12	DEFA4	8:7756576
1.44	0.205	2.15E-12	FAM13A	4:89624853	-0.129	0.0199	9.03E-11	DEFA6	8:7132385
-1.53	0.213	6.81E-13	FAM149A	4:187098581	0.189	0.0188	8.89E-24	DEFA6	8:7457281
0.149	0.0204	2.79E-13	FAM153A	5:177181107	0.522	0.0318	1.49E-60	DEFB103A	8:6942954
-0.364	0.0285	2.35E-37	FAM153C	5:177431923	0.212	0.0308	5.86E-12	DEFB103A	8:86768114
-0.63	0.0833	3.94E-14	FAM154B	15:82595566	0.322	0.0313	8.02E-25	DEFB103B	8:64062283
0.355	0.0412	6.90E-18	FAM163A	1:179732402	0.211	0.0285	1.33E-13	DEFB103B	8:75220404
-0.26	0.0379	6.88E-12	FAM170A	5:118795690	0.0524	0.00577	1.07E-19	DEFB104A	8:74323571

-0.386	0.0517	8.26E-14	FAM178B	2:97572798	-0.276	0.0355	7.57E-15	DEFB104B	8:74323571
-0.901	0.135	2.49E-11	FAM184B	4:17645710	-0.092	0.0106	3.98E-18	DEFB119	20:29617870
-3.8	0.536	1.35E-12	FAM187A	17:42937171	0.26	0.0202	6.53E-38	DEFB119	20:29617870
-0.183	0.0271	1.45E-11	FAM193A	4:3597137	1.12	0.152	1.73E-13	DEFB119	20:29617870
-1.37	0.217	2.73E-10	FAM19A4	3:68796156	-0.0756	0.0111	9.71E-12	DEFB119	20:29617870
2.24	0.348	1.22E-10	FAM211A	17:16390287	-0.189	0.0281	1.74E-11	DEFB119	20:29617870
-0.366	0.0275	2.05E-40	FAM21B	10:47917064	-2.18	0.0501	<1.0E-314	DEFB121	20:29611261
-0.593	0.0937	2.47E-10	FAM21D	10:51190471	-0.375	0.0395	2.23E-21	DEFB121	20:29422202
-0.919	0.118	6.80E-15	FAM221A	7:23719322	0.22	0.0134	1.42E-60	DEFB121	20:29422202
0.807	0.124	7.61E-11	FAM26F	6:116770217	0.185	0.0269	6.10E-12	DEFB121	20:29422202
0.638	0.0411	2.42E-54	FAM27B	9:67785194	-0.52	0.0592	1.58E-18	DEFB121	20:29422202
0.877	0.119	1.71E-13	FAM27C	9:44990610	-0.268	0.0409	5.66E-11	DEFB127	20:10372984
0.772	0.124	4.79E-10	FAM27E2	9:45730241	-0.567	0.0437	1.70E-38	DEFB130	8:127658728
-0.326	0.0414	3.42E-15	FAM27E3	9:68400922	-4.95	0.371	1.31E-40	DEFB130	8:127658728
0.288	0.0334	6.54E-18	FAM3B	21:42697714	0.0683	0.00842	4.99E-16	DEFB135	8:11873610
0.629	0.0986	1.78E-10	FAM49A	2:16718894	-0.129	0.0198	7.26E-11	DEFB135	8:11873610
-0.255	0.0374	9.22E-12	FAM83H	8:144822591	-0.133	0.0173	1.50E-14	DEFB135	8:11873610
0.325	0.0433	6.11E-14	FAM86B2	8:11950424	0.0622	0.00746	7.57E-17	DEFB135	8:11873610
-0.193	0.0234	1.61E-16	FAM89A	1:231170389	0.355	0.0331	7.76E-27	DEGS1	1:223491775
-1.17	0.186	3.17E-10	FANK1	10:127680215	0.146	0.0224	7.13E-11	DEGS1	1:223491775
3.33	0.432	1.27E-14	FAS	10:90752018	0.287	0.0256	3.60E-29	DENND6A	3:58624750
1.85	0.26	1.12E-12	FBN2	5:127873114	0.032	0.00419	2.22E-14	DESI1	22:42445693
0.562	0.0886	2.25E-10	FBXL18	7:61821630	-2.64	0.312	2.64E-17	DESI1	22:42445693
-0.155	0.0229	1.30E-11	FBXO6	1:121113600	-1.75	0.228	1.65E-14	DFNA5	7:24757660
-0.252	0.0234	4.81E-27	FBXW10	17:18669817	-0.0327	0.00385	2.00E-17	DHFR	5:79951133
1.05	0.0614	1.46E-65	FCER1A	1:159222847	0.125	0.0108	5.58E-31	DHTKD1	10:127583983
1.42	0.198	7.41E-13	FCRLB	1:161696900	-0.172	0.0195	1.14E-18	DLEU7	13:51847622
-0.268	0.0336	1.51E-15	FDCSP	4:70249195	-0.136	0.0209	7.66E-11	DNAJA2	16:46402985
-0.383	0.036	1.96E-26	FEM1A	19:4795647	-0.173	0.0105	5.44E-61	DNAJA3	16:46402985
-1.16	0.155	7.22E-14	FEZ2	2:36781138	-0.233	0.0119	2.30E-85	DNAJC15	13:43608939
0.271	0.031	2.29E-18	FFAR1	19:35837428	-0.0903	0.0135	2.25E-11	DNAJC16	1:149582977
0.194	0.02	3.01E-22	FGF6	12:37876563	-0.0994	0.0118	3.65E-17	DNAJC16	1:148851853
0.561	0.0846	3.33E-11	FGFR4	5:176509971	-0.161	0.0229	2.06E-12	DNAJC16	1:149582977
-3.16	0.382	1.31E-16	FGGY	1:60228310	-0.374	0.0267	1.40E-44	DPPA5	6:74062263
-0.883	0.135	6.12E-11	FHIT	3:59735965	-0.129	0.0186	4.05E-12	DRAXIN	1:120154679
-0.257	0.031	1.13E-16	FIGNL1	7:50485073	0.16	0.0114	9.51E-45	DRAXIN	1:121113265
-0.492	0.0486	4.35E-24	FKBP11	12:49365838	0.205	0.0227	1.70E-19	DRAXIN	1:121113600
-0.946	0.146	9.21E-11	FKBP2	11:64088562	-0.271	0.0288	4.97E-21	DRAXIN	1:121113600
-0.253	0.0325	6.99E-15	FLJ00104	16:87764267	0.0914	0.0107	1.32E-17	DRAXIN	1:121113600

-1.1	0.145	3.29E-14	FLT3	13:28598262	0.313	0.0197	7.63E-57	DSCC1	8:120868138
8.77	1.28	7.30E-12	FLYWCH1	16:2987074	0.245	0.0156	1.39E-55	DSCC1	8:120868138
0.284	0.0336	2.85E-17	FMO2	1:171167157	-0.0976	0.0117	7.31E-17	DSG3	18:29794353
0.227	0.0241	4.55E-21	FOPNL	16:15963922	-0.0936	0.013	6.02E-13	DSG3	18:29899067
-5.61	0.897	4.00E-10	FOXD4	9:110586	-0.138	0.0161	1.02E-17	DST	6:57447737
-0.214	0.0297	5.79E-13	FOXD4L1	2:114203158	-0.118	0.0112	5.91E-26	DST	6:57447737
1.67	0.239	2.80E-12	FOXD4L2	9:41947473	0.0804	0.0109	1.63E-13	DST	6:57447737
4.66	0.658	1.42E-12	FOXD4L4	9:70499939	-0.0538	0.00758	1.27E-12	DST	6:57447737
0.13	0.0207	3.38E-10	FOXD4L6	9:68412651	0.317	0.0384	1.52E-16	DST	6:57447737
0.227	0.0265	1.07E-17	FOXRED1	11:126093217	-0.144	0.0186	9.79E-15	DUX4L2	4:190897129
0.868	0.106	2.64E-16	FOXRED2	22:36795584	0.273	0.0345	2.51E-15	DUX4L2	4:190897129
2.43	0.372	6.48E-11	FP15737	8:23435117	0.426	0.036	2.63E-32	DUX4L2	4:190611445
4.29	0.633	1.22E-11	FRA10AC1	10:95460373	0.481	0.0279	1.33E-66	DUX4L2	4:190897129
-0.118	0.0161	2.32E-13	FREM2	13:39331723	-0.11	0.00622	5.48E-70	DUX4L2	4:190611445
0.166	0.0267	5.06E-10	FTCD	21:47593484	0.496	0.0463	8.87E-27	DYTN	2:20692947
-1.91	0.284	1.75E-11	FTCDNL1	2:200620763	-0.271	0.0158	6.08E-66	ECHDC3	10:11784320
-1.52	0.208	2.72E-13	FUT2	19:49199804	-0.329	0.0326	5.99E-24	ECHS1	10:135446672
0.258	0.0227	6.20E-30	FXN	9:71698978	-0.177	0.0149	1.52E-32	EFCAB2	1:245159567
-0.802	0.0576	4.55E-44	FZD3	8:28396329	-0.124	0.0146	2.01E-17	EFHB	3:19987539
-0.253	0.024	5.55E-26	GAA	17:78075228	-0.137	0.0202	1.18E-11	ENTHD1	22:40403443
-0.586	0.0702	6.97E-17	GAB4	22:17384102	-0.134	0.0146	4.39E-20	ENTHD1	22:40403443
-0.732	0.1	2.48E-13	GAL	11:68451973	-0.282	0.0314	2.69E-19	EPCAM	2:47596853
-2.6	0.36	5.11E-13	GAL3ST2	2:242703183	0.0625	0.00851	2.07E-13	EPCAM	2:47611644
-4.16	0.592	2.11E-12	GALC	14:88496080	-0.132	0.0192	6.20E-12	EPCAM	2:47596853
0.189	0.0272	3.69E-12	GALM	2:38887407	0.399	0.032	1.11E-35	ERAP2	5:96252432
-0.654	0.0979	2.38E-11	GALNT3	2:166734933	0.084	0.0116	4.44E-13	ERAP2	5:96274720
0.454	0.0724	3.59E-10	GALNT8	12:4958152	-0.348	0.0282	5.49E-35	ERLEC1	2:54642871
0.217	0.0333	7.20E-11	GAR1	4:110760941	0.137	0.016	1.10E-17	ERLEC1	2:54642871
-0.167	0.0217	1.41E-14	GAS8	16:90093075	0.367	0.0348	5.30E-26	ESCO2	8:27601280
-0.185	0.026	1.12E-12	GATAD2B	1:153773240	-0.082	0.00825	2.81E-23	ESCO2	8:27601280
0.212	0.0326	7.87E-11	GATC	12:121015907	0.264	0.029	8.75E-20	ESPL1	12:54564783
-0.285	0.0453	3.15E-10	GBP3	1:89455980	-0.361	0.0329	5.17E-28	ESPNL	2:239008067
-0.293	0.0327	3.24E-19	GCK	7:44183187	-0.179	0.0255	2.22E-12	EVC	4:49209318
-0.179	0.0227	3.13E-15	GCLM	1:94351328	-0.0933	0.0128	3.12E-13	EVC	4:49209318
-0.435	0.0688	2.57E-10	GDF5	20:34022387	0.0431	0.00636	1.23E-11	EXOC1	4:56126095
-0.489	0.0787	5.18E-10	GDF9	5:132201793	0.0736	0.0106	3.83E-12	EXOSC10	1:121113600
-0.2	0.0261	1.82E-14	GEMIN7	19:45594170	-0.161	0.018	3.74E-19	EXOSC10	1:121113600
-0.132	0.0134	6.80E-23	GEN1	2:17923343	0.102	0.0151	1.43E-11	EXOSC10	1:121113600
-0.252	0.0406	5.40E-10	GFRA4	20:3609652	0.0731	0.009	4.58E-16	EXOSC10	1:121113600

-25.8	1.65	4.12E-55	GGNBP2	17:34905455	0.0929	0.0129	5.95E-13	EXOSC10	1:121113600
-6.05	0.845	8.08E-13	GGTLC3	22:20378593	-0.171	0.0223	1.74E-14	EXOSC6	16:70563271
2.83	0.329	7.85E-18	GIMAP5	7:150437927	0.223	0.0347	1.31E-10	FABP1	2:88449963
6.21	0.864	6.60E-13	GINS1	20:25904151	-2.32	0.152	1.35E-52	FABP1	2:88438050
-11.1	0.797	4.33E-44	GLIPR1L2	12:75811234	-0.479	0.0164	1.57E-187	FAM118A	22:45728978
-26.7	2.06	2.03E-38	GLT8D2	12:104549512	0.221	0.0291	3.09E-14	FAM118A	22:45756081
16.7	1.06	6.37E-56	GMPPB	3:49813935	0.179	0.0229	5.43E-15	FAM134C	17:41382047
2.17	0.347	4.01E-10	GNA15	19:3149990	-0.318	0.0219	8.98E-48	FAM185A	7:102217297
-2.45	0.386	2.19E-10	GNAQ	9:80349435	0.0781	0.0109	7.77E-13	FAM185A	7:102217297
-4.35	0.692	3.25E-10	GNB3	12:6949026	-0.313	0.0261	3.90E-33	FAM186A	12:51788748
-4.78	0.478	1.52E-23	GNL3	3:52626443	-0.095	0.0106	3.18E-19	FAM186A	12:50290056
4.3	0.453	2.26E-21	GNLY	2:85922860	-0.463	0.037	6.30E-36	FAM193A	4:3597137
7.22	0.805	2.99E-19	GNMT	6:42928758	-0.505	0.0173	2.54E-187	FAM193A	4:3597137
-0.184	0.0195	3.88E-21	GOLGA4	3:37440726	0.123	0.0169	3.39E-13	FAM193A	4:3597137
-0.216	0.0263	2.16E-16	GOLGA6L10	15:82736362	0.164	0.0194	2.82E-17	FAM193A	4:3597137
0.228	0.0301	3.60E-14	GOLGA6L19	15:83003791	0.221	0.0248	5.04E-19	FAM193A	4:3597137
0.144	0.0223	1.07E-10	GOLGA6L20	15:82581264	0.224	0.0185	9.57E-34	FAM20B	1:179982938
1.17	0.168	3.30E-12	GOLGA6L6	15:20058655	-0.109	0.0168	8.69E-11	FAM20B	1:179861829
0.283	0.0428	3.79E-11	GOLGA6L9	15:83410535	0.428	0.0256	9.57E-63	FAM21B	10:47917284
-1.71	0.156	5.85E-28	GOLGA8H	15:30782439	0.0775	0.00652	1.39E-32	FAM21B	10:47917284
-0.144	0.0213	1.37E-11	GOLGA8O	15:32899868	0.218	0.0214	2.27E-24	FAM21B	10:47917284
-2.36	0.32	1.64E-13	GOSR1	17:28771703	-0.0844	0.0101	6.46E-17	FAM221A	7:23719322
1.62	0.226	7.60E-13	GP6	19:55500273	-0.168	0.0187	2.61E-19	FAM230A	22:21584355
-1.04	0.11	3.24E-21	GPA33	1:167035924	-0.0423	0.00572	1.41E-13	FAM230A	22:21584355
-0.773	0.0366	5.19E-99	GPAT2	2:96748054	-0.39	0.0574	1.09E-11	FAM27C	9:45367737
-1.27	0.112	8.38E-30	GPIHBP1	8:144295782	0.255	0.0398	1.48E-10	FAM27C	9:45367737
-0.821	0.108	2.92E-14	GPNMB	7:23276785	-0.55	0.0383	9.18E-47	FAM27C	9:45367737
-1.24	0.157	2.83E-15	GPR115	6:47632111	0.019	0.0028	1.16E-11	FAM27C	9:45766038
2.09	0.316	3.74E-11	GPR161	1:168090767	-0.11	0.0121	9.82E-20	FAM27C	9:45786830
-2.25	0.268	4.64E-17	GPR35	2:241561088	-0.217	0.0216	9.54E-24	FAM27D1	9:46457567
0.387	0.0558	4.05E-12	GPR39	2:133089144	0.13	0.0131	3.28E-23	FAM27D1	9:46279477
0.856	0.109	4.06E-15	GPR55	2:231795391	-0.286	0.0185	6.51E-54	FAM27E1	9:47175594
0.198	0.031	1.69E-10	GPX1	3:49317727	-0.171	0.018	2.10E-21	FAM27E1	9:47058326
1.09	0.149	2.57E-13	GREB1	2:11751261	-0.172	0.0215	1.24E-15	FAM27E2	9:44973508
-0.258	0.023	3.35E-29	GREM2	1:240699814	-0.18	0.0248	3.93E-13	FAM27E3	9:68400922
-0.311	0.0443	2.21E-12	GRID2IP	7:6585477	0.141	0.0155	9.31E-20	FAM27E3	9:68400922
0.16	0.0221	4.49E-13	GRK7	3:141549404	0.0421	0.00572	1.84E-13	FAM27E3	9:68400922
3.44	0.538	1.62E-10	GSKIP	14:96836973	-0.186	0.0269	4.70E-12	FAM27E3	9:68400922
0.233	0.037	3.03E-10	GSR	8:30536581	-0.0873	0.0128	9.08E-12	FAM27E3	9:68400922

-0.262	0.0256	1.39E-24	GSTA1	6:52663866	-0.207	0.015	2.55E-43	FBP2	9:96374277
-0.542	0.0849	1.73E-10	GSTM3	1:110260742	0.545	0.0286	5.86E-81	FBXL18	7:61821630
0.303	0.0468	9.52E-11	GSTT1	22:24334948	-2.36	0.0869	2.06E-162	FBXL18	7:61821560
-0.267	0.0316	2.93E-17	GSTT2	22:24267047	-0.782	0.0365	7.87E-102	FBXL18	7:61821560
-3.92	0.435	2.03E-19	GSTT2B	22:24267995	-0.859	0.047	1.27E-74	FBXL18	7:61821560
3.99	0.46	4.18E-18	GTF2A1L	2:48822381	0.0409	0.00623	5.20E-11	FBXL18	7:61821630
6.77	0.704	6.81E-22	GTF2IRD2	7:74350606	0.178	0.0262	1.09E-11	FBXL5	4:156014023
-4.32	0.695	5.11E-10	GTSF1	12:54845050	0.261	0.0329	2.14E-15	FBXL5	4:149003618
0.241	0.0321	6.01E-14	GUCA1A	6:42152050	0.319	0.0254	3.55E-36	FBXO6	1:121113600
2.40E-15	2.03E-16	2.98E-32	GUCA1B	6:42176743	0.253	0.0189	7.28E-41	FBXO6	1:121113600
0.261	0.021	1.83E-35	GUCA1C	3:108441584	0.12	0.0125	7.99E-22	FBXO6	1:121113600
0.381	0.0614	5.46E-10	GUF1	4:44667338	-0.264	0.0166	5.98E-57	FBXO6	1:120570084
0.473	0.0617	1.77E-14	GVQW1	9:32571346	-0.102	0.0128	1.60E-15	FBXO6	1:120570084
-1.68	0.223	4.93E-14	H3F3A	1:226271290	-0.185	0.0213	3.77E-18	FDCSP	4:70249195
-0.609	0.0697	2.38E-18	HACE1	6:105231257	-0.0996	0.0153	7.52E-11	FDCSP	4:70249195
-0.332	0.024	1.60E-43	HAUS3	4:190653610	2.74	0.197	5.61E-44	FDCSP	4:70249195
-0.691	0.0727	2.00E-21	HAUS4	14:23461583	0.166	0.024	4.62E-12	FEM1A	19:4797319
-0.671	0.099	1.22E-11	HBE1	11:5270343	0.0927	0.0108	9.22E-18	FEZ2	2:36781138
-0.903	0.103	1.84E-18	HBS1L	6:135325918	-0.0975	0.0134	3.44E-13	FEZ2	2:36779655
-0.492	0.0793	5.49E-10	HDHD3	9:116106910	0.107	0.0162	3.98E-11	FEZ2	2:36781138
-0.311	0.0282	2.79E-28	HEATR3	16:50099747	-0.172	0.0169	2.50E-24	FGF6	12:37878521
-0.838	0.111	4.37E-14	HEXDC	17:80389045	0.145	0.0215	1.54E-11	FGGY	1:60228310
0.138	0.0167	1.41E-16	HHLA3	1:70821757	-0.218	0.0248	1.49E-18	FGGY	1:60228310
0.998	0.118	2.73E-17	HIATL1	9:97095226	-0.187	0.0172	1.57E-27	FITM2	20:4268744
0.125	0.0144	3.94E-18	HIBCH	2:191189558	0.174	0.0203	1.02E-17	FITM2	20:4268744
-1.15	0.126	7.04E-20	HIGD1A	3:42832466	-0.139	0.02	3.65E-12	FKBP11	12:50005454
-0.754	0.119	2.36E-10	HIST1H2BA	6:25647151	-0.044	0.00539	3.26E-16	FKBP14	7:29426843
0.343	0.0531	1.05E-10	HIST1H3E	6:26208269	0.149	0.018	1.25E-16	FKBP15	9:115401368
-0.249	0.0354	2.01E-12	HIST1H4C	6:26102708	-0.406	0.0363	4.85E-29	FLT3	13:28603277
10.2	1.46	2.82E-12	HIST1H4F	6:26254160	0.232	0.0253	4.73E-20	FLYWCH1	16:2982341
-0.746	0.118	2.58E-10	HLA-A	6:29910286	-0.546	0.0454	2.58E-33	FOPNL	16:16470091
-2.95	0.407	4.22E-13	HLA-DOB	6:32796857	1.11	0.0359	6.59E-210	FP15737	8:23435117
-0.135	0.0187	5.23E-13	HLA-DQA2	6:32609453	0.0742	0.00562	8.45E-40	FTCDNL1	2:200620763
-0.547	0.05	7.42E-28	HLA-DQB2	6:32729014	0.102	0.00635	4.64E-58	FTCDNL1	2:200625581
0.791	0.0728	1.69E-27	HLA-DRB1	6:32538959	-2.05	0.0582	8.78E-272	FUT6	19:55907840
-3.81	0.559	9.38E-12	HLA-DRB5	6:32367017	-2.78	0.0702	<1.0E-314	FUT6	19:51493113
-0.23	0.0336	7.63E-12	HMBS	11:118950217	0.166	0.0154	4.32E-27	FZR1	19:27829622
-0.181	0.0279	8.73E-11	HMGCLL1	6:55299220	0.14	0.021	2.62E-11	FZR1	19:27829622
-0.114	0.0169	1.52E-11	HNRNPA1L2	13:53159256	-0.123	0.0158	6.98E-15	GAB4	22:17384102

2.22	0.338	5.10E-11	HNRNPCL1	1:13182567	0.186	0.00822	2.31E-113	GAB4	22:17384102
-0.978	0.136	6.42E-13	HOMER2	15:83503678	-0.183	0.0253	4.72E-13	GAB4	22:17387674
0.176	0.0216	3.70E-16	HP	16:72108093	1.2	0.0404	7.03E-194	GAB4	22:17384102
-0.793	0.115	5.36E-12	HPR	16:72108093	1.3	0.0512	3.20E-142	GABRR1	6:89146001
-0.236	0.0247	1.24E-21	HRCT1	9:35906471	-0.334	0.0447	7.90E-14	GALNT5	2:157816254
0.177	0.0248	9.53E-13	HS2ST1	1:87521319	-0.149	0.023	9.28E-11	GBP5	1:90218376
2.68	0.413	8.63E-11	hsa-mir-1199	19:14183577	0.197	0.021	6.54E-21	GFAP	17:42003548
-1.85	0.223	1.08E-16	HSBP1L1	18:77748525	-0.244	0.0242	6.59E-24	GFER	16:29086987
-0.351	0.0531	3.84E-11	HSD17B12	11:43877934	-0.207	0.0254	3.65E-16	GFER	16:16602943
-0.245	0.0322	2.77E-14	HSD17B13	4:88231392	0.22	0.0154	2.69E-46	GGT6	17:41466688
0.383	0.0504	2.98E-14	HSD17B6	12:57219622	0.35	0.0542	1.06E-10	GINS1	20:26109209
0.232	0.0337	5.81E-12	HSD3B1	1:120688329	-0.0742	0.00704	5.66E-26	GINS1	20:26109209
-0.301	0.0413	3.14E-13	HSPB7	1:16360316	-0.161	0.0237	1.10E-11	GINS1	20:26109209
-0.347	0.0505	6.36E-12	HTATIP2	11:20385606	0.142	0.0148	8.42E-22	GINS1	20:26109209
-1.35	0.217	4.93E-10	HYAL3	3:50332697	-0.206	0.0202	2.02E-24	GINS1	20:26109209
-3.2	0.509	3.24E-10	HYI	1:43911162	-0.126	0.0194	8.31E-11	GJA8	1:146471769
-0.781	0.0746	1.20E-25	IBSP	4:88732692	0.0427	0.00445	8.35E-22	GJA8	1:148346958
0.636	0.0758	4.84E-17	IDUA	4:190653610	2.39	0.368	8.33E-11	GJA8	1:148346958
2.64	0.32	1.58E-16	IFI27	14:94582130	-0.268	0.0373	6.72E-13	GLB1L3	11:134855811
0.684	0.0905	4.09E-14	IFI27L1	14:94568638	0.157	0.0222	1.53E-12	GLIPR1L2	12:75802773
-0.152	0.0221	6.08E-12	IFI27L2	14:94595989	-0.164	0.0247	3.14E-11	GLIPR1L2	12:75845789
0.283	0.0284	2.17E-23	IFIT5	10:91142586	-0.129	0.015	7.97E-18	GLIPR1L2	12:75853939
-0.671	0.0878	2.13E-14	IFNL3	19:39037400	0.936	0.0965	3.03E-22	GLT6D1	9:13861065
-0.301	0.0301	1.52E-23	IFT122	3:129240437	-0.176	0.0254	4.23E-12	GLT6D1	9:13907613
-0.62	0.0975	2.03E-10	IFT46	11:118453483	-0.147	0.02	1.98E-13	GLYAT	11:5794626
-1.62	0.246	4.54E-11	IGFLR1	19:36254702	-0.297	0.0455	6.69E-11	GNA11	19:23912867
1.86	0.273	9.55E-12	IL10RB	21:34640788	0.247	0.0219	1.68E-29	GNA15	19:3149990
-0.189	0.019	2.59E-23	IL18R1	2:102921783	-0.323	0.02	1.14E-58	GNMT	6:42928758
1.71	0.262	6.72E-11	IL1RL1	2:102951798	-1.07	0.0732	2.17E-48	GNMT	6:42928758
-2.38	0.233	1.71E-24	IL27	16:28620120	0.101	0.0143	1.63E-12	GNPAT	1:232068442
2.65	0.221	3.96E-33	IL32	16:3106003	-0.578	0.0854	1.30E-11	GOLGA6L9	15:83410535
3.14	0.364	6.33E-18	IL4	5:132424046	0.0133	0.00192	4.30E-12	GP2	16:20180187
-0.433	0.035	3.73E-35	INO80D	2:206970519	0.156	0.0107	3.80E-48	GP5	3:193749905
-0.738	0.0966	2.18E-14	INPP1	2:191231503	0.162	0.0153	3.38E-26	GP5	3:193749905
0.263	0.0418	3.14E-10	INPP5E	9:139322503	-0.126	0.0133	2.70E-21	GPR19	12:118275451
-2.03	0.261	7.38E-15	IPMK	10:60006922	-0.0782	0.0111	1.85E-12	GPR55	2:231798099
1.74	0.26	2.20E-11	IPP	1:46222493	-0.141	0.0179	3.35E-15	GPR87	3:1502342
-0.512	0.0768	2.62E-11	IQCB1	3:121480038	0.266	0.0214	1.80E-35	GPRIN2	10:4734320
-0.306	0.0314	1.93E-22	IQCG	3:197635749	0.144	0.0154	8.71E-21	GPRIN2	10:4734320

-1.12	0.0944	1.81E-32	IQCJ	3:159003145	0.164	0.0186	1.17E-18	GREB1	2:113172822
5.27	0.742	1.23E-12	IQGAP1	15:90952049	0.209	0.0317	4.31E-11	GRM6	5:1777840
-0.866	0.092	4.82E-21	IRF5	7:128589427	0.204	0.0226	1.77E-19	GSTM3	1:110260742
0.876	0.0975	2.60E-19	IRX1	5:29451631	-0.216	0.0302	8.53E-13	GSTT1	22:24334948
4.31	0.68	2.32E-10	IRX6	16:55317598	0.0163	0.00217	5.84E-14	GSTT1	22:24334948
-1.48	0.206	6.75E-13	ISCU	12:108949283	-0.172	0.0193	5.02E-19	GSTT2	22:24267047
0.128	0.0176	3.52E-13	ISG15	1:144599782	-4.39	0.656	2.20E-11	GSTT2	22:24399364
-2.62	0.272	5.84E-22	ITGB2	21:46328099	-0.755	0.0644	9.65E-32	GSTT2B	22:24295103
2.57	0.257	1.52E-23	ITGB3BP	1:63851551	-0.41	0.0224	7.75E-75	GUF1	4:44687401
-0.592	0.0573	5.07E-25	ITGB6	2:161126732	-0.219	0.0149	6.64E-49	HAUS3	4:190653610
0.199	0.0273	3.11E-13	ITIH4	3:52849493	-0.314	0.0431	3.21E-13	HAUS3	4:190653610
1.36	0.202	1.67E-11	ITPRIPL1	2:96769883	-0.0654	0.0098	2.50E-11	HAUS3	4:190653610
3.79	0.601	2.86E-10	JAKMIP3	10:133955427	0.283	0.0158	9.61E-72	HAUS3	4:190653610
0.305	0.0269	8.48E-30	KANK4	1:62739198	0.106	0.0103	7.72E-25	HAUS3	4:190653610
0.828	0.124	2.43E-11	KANSL2	12:49036033	0.119	0.016	1.03E-13	HES2	1:5735297
-1.55	0.1	3.47E-54	KATNAL2	18:44612975	-0.174	0.0248	2.28E-12	HES2	1:60244994
-0.229	0.0214	1.01E-26	KCTD20	6:36458320	-0.132	0.0129	1.42E-24	HIATL1	9:97097081
-2.19	0.241	1.02E-19	KCTD21	11:77864179	-0.118	0.0143	1.56E-16	HIATL1	9:97135769
3.57	0.393	1.05E-19	KDM7A	7:139887354	-0.0816	0.0122	2.25E-11	HIGD2A	5:175655452
2.8	0.39	7.00E-13	KHDC1L	6:73952377	0.195	0.022	7.74E-19	HIST1H2BA	6:25647151
-0.568	0.084	1.36E-11	KIAA1430	4:186125487	0.143	0.0144	3.07E-23	HIST1H2BA	6:25647151
0.131	0.015	2.47E-18	KIAA1586	6:57408345	1.33	0.201	3.67E-11	HIST3H3	1:229161
0.458	0.0494	1.84E-20	KIAA1614	1:180905694	-0.151	0.0221	8.34E-12	HIST3H3	1:228156857
0.887	0.112	2.38E-15	KIAA1841	2:61379944	0.125	0.0176	1.23E-12	HLA-DOB	6:32796857
-3.33	0.446	8.24E-14	KIAA1875	8:145164518	0.799	0.0309	2.00E-147	HLA-DOB	6:32796857
2.08	0.303	6.66E-12	KIAA2013	1:121113600	-1.87	0.167	4.19E-29	HLA-DRB1	6:32538512
-6.6	0.892	1.37E-13	KIF25	6:168398761	0.788	0.055	1.48E-46	HLA-DRB1	6:32538570
-0.218	0.0222	9.25E-23	KIF6	6:39693193	0.254	0.033	1.39E-14	HLA-DRB1	6:32542924
0.26	0.024	2.39E-27	KLC3	19:45844650	-0.142	0.0144	6.14E-23	HLA-DRB5	6:32553446
6.18	0.98	2.86E-10	KLHDC4	16:87764267	0.256	0.0252	3.03E-24	HLA-DRB5	6:32497655
-2.56	0.369	3.99E-12	KLHL18	3:47380499	-0.271	0.0202	4.88E-41	HLA-DRB5	6:32497655
-0.471	0.0644	2.60E-13	KLKB1	4:187119285	0.395	0.0297	2.33E-40	HNRNPCL1	1:13182567
0.841	0.0594	1.66E-45	KLRG2	7:139168560	0.0283	0.00431	5.16E-11	HNRNPCL1	1:13182567
1.11	0.155	7.99E-13	KNG1	3:186433574	-0.187	0.0119	1.21E-55	HNRNPCL1	1:13182567
-0.755	0.082	3.34E-20	KNOP1	16:19713315	0.55	0.0306	3.12E-72	HNRNPCL1	1:13182567
-1.88E-16	2.09E-17	2.36E-19	KRAS	12:25379637	-0.113	0.0109	3.50E-25	HNRNPCL1	1:13182567
13.1	2.1	4.43E-10	KRBA2	17:8279324	0.128	0.0157	3.55E-16	HOXB9	17:47180447
-3.46	0.334	3.80E-25	KREMEN2	16:33955968	-1.01	0.12	3.87E-17	HP	16:72108093
2.04	0.232	1.45E-18	KRR1	12:75884876	0.167	0.0219	2.43E-14	HP	16:72108093

3.03	0.389	6.74E-15	KRT31	17:39541804	0.268	0.0266	7.11E-24	HPR	16:72108093
0.25	0.0252	3.39E-23	KRT80	12:52595026	0.0384	0.00326	5.00E-32	HPR	16:72108093
0.86	0.128	1.83E-11	KRT81	12:52711776	0.0924	0.0107	5.85E-18	HS2ST1	1:87761387
0.179	0.0218	2.19E-16	KRT83	12:52702463	0.238	0.0192	2.75E-35	HSD3B1	1:120695304
6.2	0.999	5.43E-10	KRTAP5-7	11:71203436	-0.133	0.0204	7.05E-11	HSD3B1	1:120692572
-0.248	0.0237	1.26E-25	KRTAP5-9	11:71238453	0.174	0.0207	4.25E-17	HSD3B1	1:120688329
4.24	0.451	5.39E-21	L3HYPDH	14:59953981	-0.225	0.0177	5.08E-37	HSD3B1	1:120688329
2.31	0.245	4.16E-21	L3MBTL2	22:41492653	0.0845	0.0117	5.11E-13	IBSP	4:88718619
-2.27	0.329	5.21E-12	L3MBTL3	6:130374461	-0.101	0.0154	5.44E-11	ID2	2:87644591
0.787	0.113	3.29E-12	LACTB	15:63429062	0.0785	0.0118	2.88E-11	ID2	2:90446736
0.217	0.0338	1.36E-10	LAMC2	1:183155305	0.0428	0.00358	6.09E-33	ID2	2:91850402
-0.273	0.0311	1.66E-18	LARS2	3:45508718	0.144	0.0172	5.66E-17	IDO1	8:40533909
-2.3	0.347	3.40E-11	LAYN	11:111415800	-0.166	0.0257	1.05E-10	IDUA	4:190653610
0.181	0.0234	1.03E-14	LCN1	9:138415201	0.407	0.0174	5.30E-121	IFNA7	9:2150504
0.355	0.0347	1.45E-24	LCN15	9:139663458	-0.565	0.0344	1.28E-60	IFNAR1	21:34370749
-0.346	0.0316	6.69E-28	LCN8	9:139652080	-0.126	0.0146	6.13E-18	IFNL3	19:39037400
0.154	0.0229	1.76E-11	LCN9	9:138551902	0.078	0.0113	5.10E-12	IFNL3	19:39037400
-2.45	0.209	9.78E-32	LDHAL6A	11:18461054	-0.0825	0.00719	1.78E-30	IFNL3	19:39037400
-2.7	0.0705	<1.0E-314	LDHC	11:18417583	0.445	0.0116	<1.0E-314	IFNL3	19:39037400
-3.34	0.219	1.62E-52	LEFTY1	1:226099895	-0.229	0.0291	3.56E-15	IFNL3	19:39037400
-4.81	0.302	4.11E-57	LENG9	19:54951059	-0.0881	0.0135	6.76E-11	IFNLR1	1:248601069
-3.08	0.303	2.84E-24	LGR6	1:202185979	-0.0988	0.0121	3.21E-16	IL11	19:55325816
-2.76	0.21	1.87E-39	LHFPL5	6:35787417	-0.13	0.0164	2.25E-15	IL17REL	22:49594239
-3.45	0.307	2.66E-29	LIG3	17:33356907	-0.117	0.0109	7.05E-27	IL17REL	22:49594239
5.3	0.818	9.22E-11	LILRB1	19:55125147	-0.236	0.0344	6.86E-12	IL18R1	2:102953067
-3.95	0.572	5.00E-12	LIMD1	3:45700125	-0.132	0.0177	8.81E-14	IL1A	2:114152801
-2.22	0.317	2.50E-12	LIX1L	1:145194076	-2.37	0.336	1.74E-12	IL1RL1	2:102930147
1.18	0.159	1.16E-13	LNPEP	5:96223833	-0.0742	0.0102	3.48E-13	IL1RN	2:114152801
0.493	0.0753	5.86E-11	LOH12CR1	12:12619047	-0.207	0.026	1.70E-15	IL20	1:206698786
-0.295	0.0226	6.10E-39	LONP1	19:5691987	-0.201	0.0178	1.44E-29	IL20	1:206698786
0.523	0.0625	5.86E-17	LPIN1	2:11937158	0.233	0.0165	2.81E-45	IL20	1:207907085
-0.195	0.0271	6.22E-13	LPIN2	18:2944556	-0.0887	0.0136	6.93E-11	IL37	2:114343879
-1.4	0.195	7.00E-13	LRCH3	3:197609400	0.146	0.0212	5.71E-12	IL37	2:114343879
0.77	0.12	1.39E-10	LRP2BP	4:186310213	-0.248	0.0334	1.13E-13	IL4	5:132654801
-2.46	0.369	2.62E-11	LRPAP1	4:3505162	0.186	0.0194	9.01E-22	IL4	5:132424046
0.0885	0.0134	3.99E-11	LRRC2	3:46596094	-0.241	0.0236	1.75E-24	INADL	1:633277
0.37	0.0587	2.91E-10	LRRC27	10:134146084	0.243	0.0231	7.03E-26	INHBE	12:57901422
6.79	1.01	1.78E-11	LRRC37A2	17:44314261	1.21	0.0375	2.05E-228	INMT	7:29918301
0.172	0.0249	4.93E-12	LRRC37A3	17:62909742	0.321	0.0296	2.12E-27	INS	11:123253755

0.235	0.0324	4.07E-13	LRRC55	11:56473109	-2.86	0.279	1.17E-24	IQCF6	3:51286778
-3.04	0.457	2.89E-11	LRRC61	7:150037660	0.244	0.0236	4.69E-25	IREB2	15:79257278
-0.55	0.0864	1.94E-10	LRRCC1	8:86055093	0.128	0.0182	2.02E-12	IRG1	13:76567705
-0.178	0.0269	3.66E-11	LRRIQ3	1:74663709	-0.0919	0.00745	5.83E-35	IRX1	5:29451631
-9.94E-17	1.20E-17	1.20E-16	LSG1	3:194369579	0.299	0.0137	1.35E-105	IRX1	5:29451631
11.9	1.08	3.11E-28	LURAP1L	9:12744670	0.19	0.0163	2.13E-31	IRX1	5:29451631
9.31	1.39	2.12E-11	LXN	3:158403318	0.142	0.0216	4.90E-11	IRX2	5:29451631
-5	0.8	4.10E-10	LY6D	8:143877091	-0.397	0.0244	1.60E-59	IRX2	5:29451631
0.19	0.0182	1.64E-25	LY6G6C	6:31741659	0.0775	0.00866	3.58E-19	IRX2	5:29451631
0.181	0.0261	4.07E-12	LY6K	8:143792357	0.159	0.0207	1.58E-14	ISG15	1:142618462
4.92	0.506	2.40E-22	LYPD1	2:133346781	0.34	0.0417	3.54E-16	ISG15	1:144599782
2.18	0.247	1.09E-18	LYPD8	1:248818466	-0.471	0.058	4.64E-16	ISG15	1:144599782
0.806	0.109	1.42E-13	LYRM2	6:90342049	0.164	0.0147	6.66E-29	ITGB2	21:46328099
-3.33	0.397	4.95E-17	MADD	11:47875178	-0.204	0.0314	8.20E-11	ITGB2	21:46328099
3.27	0.404	5.77E-16	MAEL	1:166955312	-0.225	0.0184	2.20E-34	ITGB3BP	1:63851551
-1.05E-15	1.31E-16	1.10E-15	MAN2B2	4:6611238	-0.115	0.0126	7.04E-20	ITGB3BP	1:63941202
5.13E-17	7.92E-18	9.34E-11	MANBA	4:103680984	-0.185	0.0136	3.85E-42	ITK	5:156521293
0.193	0.0284	1.08E-11	MAP1LC3A	20:33136296	-0.228	0.0261	2.42E-18	IYD	6:151694262
-0.387	0.0376	7.61E-25	MAPK12	22:50695270	-0.248	0.0195	4.70E-37	IYD	6:151694265
-0.829	0.121	7.32E-12	MAPT	17:43666385	-0.134	0.0199	1.65E-11	JPH2	20:41861625
1.76	0.16	3.82E-28	MB	22:36001569	0.0928	0.0124	7.22E-14	KATNBL1	15:34099057
-0.838	0.114	1.97E-13	MCFD2	2:47136950	-0.179	0.0139	6.01E-38	KCNA7	19:49129486
-1.07	0.161	3.01E-11	MCM8	20:5961458	-0.126	0.0145	3.64E-18	KCNK6	19:39490978
-2.82	0.389	4.19E-13	MCOLN2	1:85474400	0.097	0.01	3.01E-22	KCTD13	16:29961344
2.21	0.341	9.12E-11	MCPH1	8:6302154	0.12	0.0182	4.30E-11	KCTD7	7:6526147
-0.23	0.0298	1.18E-14	MDH1B	2:207645464	0.145	0.0167	3.87E-18	KIAA0232	4:6761192
0.196	0.0271	4.74E-13	MEFV	16:33941694	-1.6	0.122	2.71E-39	KIAA0232	4:77694232
-2.66	0.381	2.92E-12	MEI1	22:42193741	0.258	0.0295	2.22E-18	KIAA1143	3:44126376
-0.2	0.0293	8.74E-12	METTL18	1:169758564	-0.284	0.0236	2.36E-33	KIAA1143	3:44126376
1.77	0.251	1.77E-12	METTL21B	12:58188696	0.146	0.0158	2.45E-20	KIAA1586	6:57408345
-1.97	0.239	1.68E-16	MGC10955	2:74347525	0.436	0.016	1.66E-163	KIAA1586	6:57447737
-2.38	0.256	1.45E-20	MGLL	3:127394280	0.114	0.0153	9.26E-14	KIAA1875	8:145164518
1.88	0.224	4.74E-17	MGMT	10:131265545	-0.498	0.0381	4.83E-39	KIAA1875	8:145175529
7.47	0.793	4.51E-21	MGRN1	16:4737383	0.0969	0.0142	8.86E-12	KIAA2013	1:121113600
-6.76	1.08	3.87E-10	MICB	6:31474000	0.116	0.0167	3.76E-12	KIAA2013	1:121113600
-0.157	0.0217	4.65E-13	MIIP	1:121113600	-3.75	0.403	1.34E-20	KIAA2013	1:121113600
-0.266	0.0294	1.46E-19	MLANA	9:68457329	-0.176	0.00742	2.26E-124	KIAA2013	1:121113600
-3.34	0.529	2.72E-10	MLH3	14:75463848	-0.152	0.0152	1.52E-23	KIAA2013	1:121113600
-1.66	0.26	1.72E-10	MLK4	1:233529472	0.327	0.0163	1.61E-89	KIDINS220	2:90446736

0.293	0.0427	6.80E-12	MMP26	11:51577085	-0.0374	0.0046	4.28E-16	KIDINS220	2:90446736
-1.27	0.168	4.05E-14	MMP7	11:102401633	0.0315	0.00473	2.75E-11	KIF25	6:168391579
2.65	0.393	1.55E-11	MMRN1	4:90811007	0.311	0.0288	3.49E-27	KLB	4:3860612
-4.35	0.545	1.44E-15	MOCS2	5:52399688	-0.24	0.0214	3.44E-29	KLB	4:40436032
-0.19	0.0276	5.82E-12	MON1B	16:77232746	-0.0889	0.013	8.00E-12	KLB	4:40436032
-2.82	0.423	2.62E-11	MPC2	1:167923678	-0.156	0.0236	3.84E-11	KRBA2	17:80989217
-3.99	0.581	6.54E-12	MPHOSPH6	16:82184200	-0.314	0.0184	2.69E-65	KRBA2	17:77160151
0.224	0.0314	9.77E-13	MPI	15:75206225	0.104	0.0146	1.05E-12	KRCC1	2:87644591
0.291	0.0393	1.32E-13	MPPE1	18:11905953	-0.177	0.0128	1.72E-43	KREMEN2	16:33533441
7.36	1.16	2.23E-10	MRC1	10:18229566	-0.112	0.00752	3.63E-50	KREMEN2	16:32104335
-1.24	0.173	7.63E-13	MRC1L1	10:18249664	0.0941	0.0101	1.20E-20	KREMEN2	16:33533441
-1.52	0.236	1.19E-10	MRFAP1L1	4:6709687	-0.128	0.00962	2.15E-40	KREMEN2	16:34015761
-0.723	0.105	5.75E-12	MRI1	19:13871830	-0.225	0.0231	2.03E-22	KREMEN2	16:34190292
-0.536	0.0463	5.41E-31	MRM1	17:34963333	-0.121	0.0172	1.99E-12	KRT27	17:38642290
0.541	0.0845	1.53E-10	MRPL10	17:45890898	-0.144	0.0122	3.75E-32	KRT75	12:52492131
0.139	0.0212	5.50E-11	MRPL18	6:160211445	-0.188	0.02	5.46E-21	KRT75	12:52613830
0.72	0.112	1.29E-10	MRPL19	2:75878511	-0.247	0.0186	3.04E-40	KRT83	12:52720482
0.213	0.0265	9.15E-16	MRPL21	11:68669496	-0.301	0.0215	1.56E-44	KRT84	12:53343231
-4.14	0.662	4.01E-10	MRPL24	1:156699977	-0.294	0.0386	2.60E-14	KRTAP10-2	21:46872370
-9.12E-15	9.54E-16	1.18E-21	MRPL53	2:74678921	-0.158	0.0216	2.58E-13	KRTAP1-4	17:39968701
-2.43	0.39	4.64E-10	MRPS10	6:42191176	0.162	0.014	5.75E-31	KRTAP19-2	21:31730168
-2.74	0.249	3.65E-28	MRPS17	7:56008918	-0.107	0.0133	8.62E-16	KRTAP19-6	21:31193155
4.89	0.717	9.10E-12	MRPS26	20:25904151	-1.36	0.175	7.76E-15	KRTAP22-2	21:32471803
4.04	0.553	2.76E-13	MRTO4	1:19579312	-0.194	0.0271	8.15E-13	KRTAP22-2	21:31562202
-4.3	0.526	2.96E-16	MS4A5	11:59988193	0.0109	0.00165	3.95E-11	KTN1	14:56654212
-5.7	0.883	1.08E-10	MSH3	5:79960955	-0.0833	0.00944	1.10E-18	L1TD1	1:633277
0.291	0.0393	1.32E-13	MSH4	1:76258518	0.0454	0.0062	2.43E-13	L3HYPDH	14:60018584
1.62	0.218	1.08E-13	MTFR1L	1:26143704	-0.0743	0.0111	2.18E-11	LAMC2	1:183155305
2.54	0.337	4.81E-14	MTHFR	1:121113600	-4.43	0.323	8.24E-43	LAPTM4A	2:194495397
-0.23	0.0334	5.73E-12	MTHFS	15:80121160	0.125	0.0171	2.67E-13	LAPTM4A	2:194495397
-0.165	0.0262	3.02E-10	MTOR	1:121113600	1.74	0.162	6.55E-27	LBP	20:36447486
-0.124	0.017	3.01E-13	MTRNR2L4	16:33941694	-0.179	0.0241	1.11E-13	LBP	20:36920560
-1.46	0.222	4.81E-11	MTRR	5:7862381	-0.14	0.0138	3.49E-24	LCE1E	1:153555057
0.978	0.157	4.69E-10	MUC4	3:195671091	-0.175	0.0174	8.52E-24	LCE2B	1:153602096
-0.567	0.0653	3.85E-18	MUL1	1:20826782	-0.15	0.0124	1.10E-33	LCE2D	1:152823789
-16.8	2.41	3.15E-12	MXD3	5:176740244	-0.211	0.0271	6.92E-15	LCE3A	1:152784023
-2.15	0.153	7.46E-45	MXRA7	17:74706380	-0.115	0.0104	2.01E-28	LCE3C	1:153485794
-2.99	0.462	9.68E-11	MYADML2	17:79898644	-0.252	0.0235	7.90E-27	LCE3E	1:152770613
-3.75	0.438	1.11E-17	MYLK4	6:2774052	0.0569	0.00743	1.89E-14	LCN1	9:138415201

0.178	0.0256	3.57E-12	MZT2A	2:132235137	-0.434	0.0366	1.96E-32	LCN1	9:138415201
-0.127	0.0161	3.07E-15	N6AMT1	21:30207782	-0.164	0.015	7.99E-28	LCN1	9:138415201
-0.983	0.149	4.19E-11	NAGS	17:42088389	-0.32	0.0295	2.05E-27	LDHAL6A	11:18417583
0.447	0.0507	1.18E-18	NAIP	5:70429598	0.483	0.0392	6.95E-35	LDHC	11:18432033
0.219	0.0339	1.05E-10	NAPRT1	8:144676862	0.838	0.0349	2.11E-127	LDHC	11:18421237
2.55	0.389	5.55E-11	NARS2	11:78282632	0.17	0.0201	2.73E-17	LDHC	11:18432033
-0.553	0.0697	2.12E-15	NBPF12	1:146391234	-0.423	0.0375	1.65E-29	LEPROTL1	8:2958662
-0.302	0.0359	4.02E-17	NBPF16	1:148531065	0.304	0.0284	9.72E-27	LGALS9B	17:21200341
-2.04	0.256	1.60E-15	NCBP2	3:196574607	-0.0884	0.0117	4.17E-14	LGALS9B	17:21326832
-0.441	0.0434	2.95E-24	NCKIPSD	3:48744484	0.176	0.0211	7.35E-17	LIAS	4:4019330
-2.57	0.35	2.09E-13	NDUFAF1	15:41626148	-0.183	0.0126	8.56E-48	LIAS	4:4019330
-9.2	1.48	5.09E-10	NFKBIB	19:39398416	0.125	0.0159	3.79E-15	LILRA3	19:5407203
-0.227	0.0171	3.24E-40	NHLH2	1:116372092	-0.108	0.014	1.22E-14	LIX1L	1:145194076
0.229	0.0281	3.66E-16	NINJ1	9:95882901	-0.174	0.0261	2.62E-11	LIX1L	1:145194076
0.186	0.0276	1.59E-11	NIPSNAP3A	9:107519449	-0.153	0.0197	8.07E-15	LIX1L	1:145194076
0.314	0.0373	3.82E-17	NKAIN1	1:31678626	0.345	0.02	1.12E-66	LIX1L	1:145194076
0.25	0.0314	1.70E-15	NKAIN4	20:61881911	0.577	0.0664	3.63E-18	LIX1L	1:145194076
0.441	0.054	3.17E-16	NLGN2	17:7322440	-0.106	0.0137	1.02E-14	LMCD1	3:77832420
1.17	0.137	1.34E-17	NLRP2	19:55474210	0.495	0.0303	5.42E-60	LOXHD1	18:4491075
0.284	0.0403	1.83E-12	NME5	5:137546642	-0.363	0.0509	9.92E-13	LRRC37A2	17:43678071
0.584	0.0673	4.04E-18	NME9	3:138033181	0.138	0.0184	6.38E-14	LRRC37A2	17:43666385
1.69	0.248	9.46E-12	NMRK1	9:77707300	-0.399	0.0153	6.41E-150	LRRC37A2	17:43687268
-0.227	0.0171	3.24E-40	NMUR1	2:232379712	-0.0286	0.00428	2.35E-11	LRRC47	1:39284122
0.229	0.0281	3.66E-16	NMUR2	5:151841898	-0.0815	0.0105	8.37E-15	LRRC55	11:56473109
0.186	0.0276	1.59E-11	NOB1	16:69782855	0.0783	0.0114	6.49E-12	LRRC55	11:56473109
0.314	0.0373	3.82E-17	NODAL	10:72155879	0.0901	0.0108	7.27E-17	LRRC55	11:56473109
0.25	0.0314	1.70E-15	NOMO2	16:18433187	-0.27	0.0409	4.07E-11	LRRC55	11:56473109
0.441	0.054	3.17E-16	NOP10	15:34632686	-0.295	0.0312	3.23E-21	LRRC55	11:56473109
1.17	0.137	1.34E-17	NOP14	4:3597137	-2.4	0.189	6.03E-37	LRRN4CL	11:62226112
0.284	0.0403	1.83E-12	NOS1	12:117902158	-0.0949	0.0137	4.30E-12	LSG1	3:194369579
0.584	0.0673	4.04E-18	NOTCH4	6:32178773	-0.127	0.0193	4.69E-11	LSG1	3:194369579
1.69	0.248	9.46E-12	NOXRED1	14:77863091	-0.0425	0.00553	1.53E-14	MAP1LC3C	1:242657380
			NPHP3	3:132438099	-0.372	0.0284	3.35E-39	MBNL2	13:97373613
			NPIPA1	16:15129940	-0.189	0.0267	1.46E-12	MDM4	1:204094781
			NPIPA2	16:15220753	0.836	0.0412	1.54E-91	MEFV	16:33991563
			NPIPA5	16:15045766	1.06	0.0714	7.39E-50	MEFV	16:33941694
			NPIPA8	16:18441762	1.09	0.0735	9.38E-50	MEFV	16:33941694
			NPIPB6	16:28708127	0.132	0.0184	7.29E-13	MEFV	16:33941694
			NPIPB7	16:28495752	-0.375	0.0255	5.91E-49	MEFV	16:33941694

NPIPB9	16:28823755	-0.19	0.0277	6.92E-12	MESDC1	15:80955534
NPM1	5:170808238	-0.179	0.0113	1.63E-56	MGC10955	2:74344685
NPSR1	7:34718523	0.0295	0.00386	2.13E-14	MGC10955	2:74342260
NPY2R	4:156084560	0.342	0.0178	2.85E-82	MICALCL	11:123453335
NRBF2	10:64914518	-0.177	0.0175	4.77E-24	MICALCL	11:123784212
NRG4	15:76308132	0.435	0.0256	9.38E-65	MIF	22:25056098
NSA2	5:74155991	0.475	0.0288	4.12E-61	MIIP	1:121113600
NSUN2	5:6618323	-0.517	0.0164	4.02E-218	MIIP	1:121113600
NSUN6	10:18939070	0.109	0.0162	1.72E-11	MIIP	1:121113600
NUP107	12:69077497	-0.12	0.0183	5.48E-11	MIIP	1:121113600
NUP210L	1:153666248	0.115	0.00897	1.26E-37	MIIP	1:121113600
NUP85	17:73192415	-0.201	0.0176	3.31E-30	MKRN1	7:139713109
NUPL2	7:23224367	-0.163	0.0148	3.29E-28	MKRN1	7:139713109
NUPR1L	7:56104688	0.38	0.0342	1.11E-28	MLANA	9:68457329
NUSAP1	15:41642036	0.158	0.0146	2.71E-27	MLANA	9:68457329
NWD1	19:16829435	-0.315	0.0389	5.60E-16	MLANA	9:68457329
OBP2A	9:138436299	-0.106	0.0098	2.88E-27	MLANA	9:68457329
OCLN	5:69783228	-0.112	0.0173	9.54E-11	MLANA	9:68457329
OPA3	19:46056620	-0.124	0.0152	3.41E-16	MLK4	1:233514934
OPN1SW	7:128479367	0.146	0.0205	1.06E-12	MLK4	1:233529472
OPN4	10:88381941	-0.329	0.0203	4.51E-59	MMP1	11:103154832
OR10H5	19:15885942	0.0457	0.0046	2.94E-23	MMP1	11:103154832
OR11H1	22:16880695	-0.0685	0.0102	1.87E-11	MMP26	11:51577085
OR1A2	17:21210337	-0.129	0.0201	1.38E-10	MPDU1	17:80989217
OR1D2	17:21538565	-0.0447	0.00693	1.12E-10	MPHOSPH6	16:82186383
OR1D5	17:25335635	-0.0935	0.0108	4.83E-18	MPHOSPH6	16:82167073
OR2L13	1:248128929	0.331	0.0251	1.04E-39	MPHOSPH6	16:82167278
OR2L2	1:248128929	0.0874	0.00934	8.16E-21	MPPE1	18:11905953
OR4A47	11:48388105	-0.107	0.0139	1.38E-14	MRFAP1L1	4:6709687
OR51A7	11:51575314	-0.07	0.0105	2.62E-11	MRGPRX3	11:1902983
OR51B4	11:48356870	-0.184	0.0212	3.98E-18	MRGPRX3	11:1902983
OR51E2	11:4755611	0.0845	0.00543	1.33E-54	MRPL10	17:45936427
OR51I1	11:48355553	-0.133	0.0183	3.65E-13	MRPL14	6:44207850
OR51I2	11:61862695	0.209	0.0183	3.29E-30	MRPL21	11:68681769
OR52B6	11:56470894	-0.0606	0.00814	9.72E-14	MRPS10	6:42164270
OR52E6	11:51577359	-0.0828	0.00981	3.16E-17	MRPS33	7:140367431
OR52K2	11:48350545	-0.0935	0.0141	3.33E-11	MS4A3	11:58985363
OR52N5	11:48373976	-0.122	0.0163	7.17E-14	MS4A5	11:59988193
OR56A1	11:56470865	-0.0834	0.011	3.41E-14	MT1B	16:57156709

OR5I1	11:55044296	-0.0238	0.00347	6.94E-12	MTFMT	15:64345880
OR6B3	2:240988851	0.14	0.00756	1.46E-76	MTHFR	1:121113600
OR6V1	7:142116439	-0.124	0.00911	3.43E-42	MTHFR	1:121113600
OR7C1	19:14932957	0.0671	0.0101	3.06E-11	MTHFR	1:121113600
ORC6	16:46402985	0.962	0.144	2.38E-11	MTHFR	1:121113600
OSBPL11	3:125467425	0.22	0.0336	5.85E-11	MTHFR	1:121113600
OSBPL5	11:3109284	-0.14	0.0166	3.35E-17	MTMR12	5:3127233
OSCP1	1:36886117	0.206	0.0245	4.16E-17	MTOR	1:121113600
OTUD3	1:20233086	0.365	0.0328	9.16E-29	MTOR	1:121113600
OVGP1	1:111933638	-0.166	0.0237	2.48E-12	MTOR	1:121113600
OVOL1	11:65551648	0.0559	0.00552	4.20E-24	MTOR	1:121113600
OXGR1	13:97647188	-0.0948	0.0142	2.45E-11	MTOR	1:121113600
OXTR	3:8811646	0.352	0.0351	1.14E-23	MTRNR2L4	16:33941694
PAAF1	11:73622667	-0.244	0.0327	8.54E-14	MTRNR2L4	16:33941694
PACRG	6:163037975	0.26	0.0256	3.11E-24	MTRNR2L4	16:33941694
PADI4	1:17673102	0.538	0.0188	4.13E-180	MTRNR2L4	16:33941694
PAH	12:103351826	0.177	0.0214	1.33E-16	MTRNR2L4	16:33941694
PAOX	10:135196301	0.128	0.0195	5.23E-11	MUC4	3:195412486
PAX8	2:113978650	-0.277	0.016	3.79E-67	MUC4	3:195670876
PCDHA10	5:140266942	-0.512	0.0381	3.61E-41	MUC4	3:195344123
PCDHA13	5:140239423	-0.253	0.0151	5.21E-63	MUC4	3:195344161
PCDHA7	5:140120733	-0.0804	0.012	2.08E-11	MUC6	11:13767506
PCDHA9	5:140229368	-0.0735	0.00735	1.52E-23	MUL1	1:21790318
PCDHAC1	5:140242479	0.102	0.0126	5.72E-16	MYBL2	20:41863339
PCDHB10	5:140522829	-0.0835	0.0121	5.17E-12	MYBPH	1:203714629
PCDHB12	5:140565046	-0.0847	0.0109	7.81E-15	MYL12B	18:33443
PCDHB2	5:140464559	0.142	0.017	6.66E-17	MYL12B	18:29937076
PCDHB3	5:140502343	0.0805	0.0119	1.34E-11	MYLK3	16:46464024
PCDHB8	5:140539727	0.127	0.012	3.56E-26	NAMPTL	10:36973081
PCDHGA6	5:140737947	-0.286	0.0429	2.62E-11	NANOG	12:73094439
PCNXL4	14:60477929	0.121	0.0188	1.23E-10	NANP	20:25904151
PCSK9	1:55505668	-0.0516	0.00797	9.53E-11	NANP	20:25904151
PDCD1LG2	9:5509678	0.0389	0.00516	4.74E-14	NANP	20:25904151
PDHB	3:58380465	0.0935	0.012	6.61E-15	NANP	20:25904151
PDLIM5	4:95576397	0.172	0.024	7.68E-13	NAPRT1	8:144676862
PDPR	16:70195967	0.573	0.0441	1.34E-38	NAPRT1	8:144683934
PDXDC1	16:15399294	0.106	0.0154	5.86E-12	NAPRT1	8:144683934
PDZK1IP1	1:47655816	0.159	0.0238	2.38E-11	NCF1	7:73549837
PDZRN4	12:41832683	0.182	0.018	4.93E-24	NDUFA8	9:124891476

PEBP4	8:22570783	-0.297	0.0311	1.30E-21	NDUFAF1	15:41648737
PEX6	6:42925683	0.238	0.0202	4.82E-32	NDUFC1	4:139948061
PGA3	11:60961842	-0.099	0.0149	3.05E-11	NDUFC1	4:139948055
PHLDA2	11:2988587	0.301	0.0354	1.85E-17	NFATC2IP	16:29092297
PI4K2B	4:25159390	-0.272	0.0301	1.62E-19	NKAIN1	1:31678626
PIF1	15:65136471	-0.179	0.0191	7.14E-21	NLRP6	11:1018012
PIGH	14:68066559	-0.0899	0.0112	1.00E-15	NLRP6	11:12087993
PIGN	18:59734744	-0.0853	0.0124	6.03E-12	NMRK1	9:77701908
PIGZ	3:196675994	0.15	0.0162	2.06E-20	NMRK1	9:77707300
PISD	22:32015162	0.142	0.0216	4.90E-11	NOL7	6:145416470
PKMYT1	16:3057622	0.135	0.0186	3.93E-13	NOL7	6:127760380
PKP1	1:201208962	-0.117	0.0159	1.86E-13	NOL9	1:5735297
PLCD1	3:38053206	-0.258	0.038	1.13E-11	NOL9	1:5735297
PLEKHH2	2:43881850	0.176	0.0257	7.48E-12	NOP14	4:3597137
PLXNA2	1:208372139	-0.128	0.0161	1.86E-15	NOP14	4:3597137
PM20D1	1:205767885	-0.0598	0.00447	8.12E-41	NOP14	4:3597137
PMF1-BGLAP	1:156307593	-0.257	0.0386	2.78E-11	NOP14	4:3597137
POLE3	9:116210763	-0.177	0.0229	1.08E-14	NOP14	4:3597137
POLM	7:44111189	0.196	0.0267	2.12E-13	NPIPA2	16:15220753
POLR1B	2:113305424	0.16	0.0162	5.26E-23	NPIPA2	16:15211229
POLR1D	13:28196033	0.176	0.0216	3.70E-16	NPIPA5	16:15045766
POLR1E	9:37489064	-0.109	0.0146	8.28E-14	NPIPB7	16:28539293
POLR2C	16:57439588	0.0899	0.0128	2.16E-12	NPM1	5:170803458
POLR2J	7:102114424	0.453	0.0232	6.62E-85	NPY2R	4:156084560
POM121C	7:75081418	0.116	0.0103	2.02E-29	NRG4	15:76308132
POM121L2	6:27454446	0.0323	0.00444	3.47E-13	NSA2	5:74002438
POMC	2:25384456	0.987	0.0584	4.45E-64	NSUN2	5:6619733
POMGNT2	3:43111239	0.109	0.0164	3.00E-11	NSUN2	5:6619733
POP5	12:120912240	0.119	0.0162	2.05E-13	NSUN2	5:6619733
POTEC	18:14535287	0.00901	0.00129	2.86E-12	NTN3	16:32117785
POTEM	14:19011416	-0.099	0.0134	1.49E-13	NTN3	16:32601856
PPA2	4:106286947	0.249	0.016	1.31E-54	NTN3	16:32117769
PPFIA1	11:70156584	-0.0977	0.0147	3.01E-11	NTN3	16:33941694
PPIL3	2:201754063	-0.283	0.0184	2.21E-53	NUBPL	14:31747848
PPM1M	3:52341215	-0.165	0.0189	2.54E-18	NUDT19	19:32801840
PPM1N	19:46006212	-0.164	0.02	2.40E-16	NUDT19	19:32815142
PPP2R1B	11:111648809	-0.199	0.0216	3.17E-20	NUDT19	19:32454194
PPP2R3C	14:35625068	-0.133	0.0183	3.65E-13	NUGGC	8:27601280
PPP3CC	8:22337996	-0.11	0.0105	1.11E-25	NUGGC	8:27710767

PPP4C	16:30092048	-0.0881	0.0129	8.52E-12	NUP210L	1:153666248
PRAMEF12	1:121363032	-0.0219	0.00334	5.49E-11	NXPE1	11:114003674
PRAMEF26	1:12908500	-0.0308	0.00355	4.10E-18	NXPE1	11:114003674
PRAMEF6	1:121141536	-0.0466	0.00579	8.39E-16	OARD1	6:405873
PRB2	12:11592075	0.258	0.0272	2.42E-21	OAT	10:126677160
PRDM15	21:43296188	0.119	0.0169	1.90E-12	OCIAD2	4:49566198
PRPH2	6:42672434	0.401	0.0186	4.35E-103	OCIAD2	4:49209318
PRR23D1	8:7640939	0.0417	0.00394	3.54E-26	OCIAD2	4:49209318
PRR23D2	8:7640939	0.0417	0.00394	3.54E-26	OPA1	3:193749905
PRSS1	7:142461921	-0.182	0.0169	4.81E-27	OR10A6	11:7835477
PRSS33	16:33941694	-0.528	0.0507	2.14E-25	OR11H1	22:16880695
PRSS36	16:31154146	-0.0998	0.0154	9.14E-11	OR11H1	22:16880695
PSCA	8:143773166	0.185	0.018	8.88E-25	OR11H1	22:16934964
PSORS1C1	6:31117075	0.213	0.0245	3.50E-18	OR11H1	22:16892695
PTGR1	9:114357659	-0.192	0.0192	1.52E-23	OR1A1	17:22249643
PTPN20A	10:46590033	-0.358	0.0208	2.18E-66	OR1D2	17:22260458
PTPN20B	10:48984205	-0.159	0.0221	6.27E-13	OR1D5	17:25335635
PTPRCAP	11:67205978	-0.303	0.0253	4.73E-33	OR1D5	17:25335635
PTPRN2	7:158380436	-0.18	0.0253	1.12E-12	OR1D5	17:25335635
QRSL1	6:107077502	-0.127	0.01	5.91E-37	OR1D5	17:25335635
QSOX2	9:139098514	-0.145	0.0194	7.77E-14	OR1D5	17:25335635
RAB17	2:238491258	0.189	0.0233	5.00E-16	OR2B2	6:27954826
RAB27A	15:55608051	0.246	0.0267	3.16E-20	OR2J2	6:2970312
RAB6C	2:130694072	0.145	0.0106	1.35E-42	OR2J2	6:2970312
RABEP1	17:5290703	-0.0732	0.00998	2.22E-13	OR2J2	6:2970312
RAC1	7:61821560	2.61	0.293	5.20E-19	OR2T12	1:248907328
RAD51C	17:56526096	-0.156	0.0182	1.02E-17	OR2T27	1:248814415
RAD9A	11:66899619	0.337	0.0405	8.72E-17	OR2T29	1:249205928
RADIL	7:4809336	0.343	0.0433	2.35E-15	OR2T5	1:249205928
RANBP6	9:68400922	1.52	0.178	1.35E-17	OR3A1	17:22246520
RARRES1	3:158450417	0.139	0.014	3.13E-23	OR3A1	17:22246520
RARS	5:168021502	-0.0826	0.0129	1.52E-10	OR4A47	11:48388105
RASGRP3	2:33754336	0.371	0.0419	8.41E-19	OR4A47	11:48388105
RAX2	19:27878681	1.07	0.113	2.82E-21	OR4A47	11:48388105
RBBP8NL	20:60992402	0.209	0.0147	7.12E-46	OR4A47	11:48388105
RBL2	16:53523947	-0.201	0.0202	2.51E-23	OR4A47	11:48388105
RBPMS2	15:64828504	0.423	0.0376	2.32E-29	OR4F15	15:102507456
RCBTB1	13:50101073	-0.126	0.0173	3.26E-13	OR4F15	15:102506525
RCHY1	4:76408064	0.205	0.0178	1.09E-30	OR4F4	15:102506525

RECK	9:36070025	0.0779	0.012	8.49E-11	OR4X2	11:48354957
RESP18	2:220191861	-0.573	0.0228	2.25E-139	OR51A7	11:48351005
REV3L	6:111593029	-0.127	0.0154	1.63E-16	OR51A7	11:51575314
RFPL3	22:32582654	0.0807	0.00625	3.85E-38	OR51A7	11:51575314
RFPL3S	22:32786217	-0.196	0.0199	6.91E-23	OR51A7	11:51575314
RFWD3	16:74548007	-0.108	0.0136	2.00E-15	OR51B4	11:48356870
RGPD8	2:113114004	-0.195	0.024	4.47E-16	OR51B4	11:48356870
RGR	10:86004238	0.709	0.0543	5.79E-39	OR51B4	11:48356870
RGS14	5:176798040	-0.31	0.0272	4.32E-30	OR51B4	11:48356870
RHOD	11:66824261	-0.415	0.0412	7.29E-24	OR51B4	11:48356870
RIBC2	22:45833924	-0.195	0.0114	1.36E-65	OR51I1	11:48355553
RIC3	11:8129023	-0.17	0.0254	2.19E-11	OR51I1	11:48355553
RIIAD1	1:151699416	-0.301	0.0381	2.78E-15	OR51I1	11:48355553
RIMS2	8:105262561	0.167	0.0166	8.28E-24	OR51I1	11:48355553
RLBP1	15:89763535	0.241	0.0325	1.21E-13	OR51I1	11:48355553
RMDN1	8:87338452	0.263	0.0205	1.12E-37	OR51I2	11:61862695
RMI2	16:11439303	-0.404	0.0177	2.60E-115	OR51I2	11:61862695
RNASEL	1:182567440	0.0975	0.0122	1.33E-15	OR51I2	11:61862695
RNF149	2:101922360	0.14	0.02	2.56E-12	OR51I2	11:61862695
RNF166	16:88797926	0.189	0.0292	9.63E-11	OR51I2	11:61862695
RNF186	1:206514654	-0.54	0.0433	1.07E-35	OR51J1	11:56468554
RNF213	17:78239532	0.144	0.022	5.93E-11	OR51J1	11:56468403
RNF39	6:29859804	0.0759	0.00485	3.35E-55	OR52B4	11:51160886
RNF4	4:3597137	-1.66	0.165	8.25E-24	OR52B6	11:51578565
RNPEP	1:201968263	-0.132	0.0166	1.84E-15	OR52B6	11:56470894
RORA	15:61468065	-0.23	0.019	9.91E-34	OR52B6	11:56470894
RP11-1026M7.	5:177218594	0.747	0.0255	1.23E-188	OR52B6	11:56470716
RP11-166B2.1	16:12070422	-0.198	0.0285	3.72E-12	OR52B6	11:56472983
RP11-176H8.1	14:31910733	0.227	0.0297	2.12E-14	OR52D1	11:48340727
RP11-195B21.3	9:67836868	0.302	0.0409	1.54E-13	OR52D1	11:48340727
RP11-298I3.5	14:23371964	-0.28	0.0411	9.58E-12	OR52D1	11:60813114
RP11-345J4.5	16:30321064	-0.348	0.0247	4.43E-45	OR52D1	11:60813114
RP11-45M22.4	17:17151664	0.157	0.0243	1.04E-10	OR52E6	11:51577359
RP11-507M3.1	2:24401315	-0.0635	0.00661	7.49E-22	OR52E6	11:51577359
RP11-514P8.6	7:102314422	0.258	0.027	1.23E-21	OR52E6	11:51577359
RP11-644F5.10	12:56115585	-0.211	0.017	2.26E-35	OR52E6	11:51577359
RP11-712L6.5	11:126163124	0.18	0.0244	1.62E-13	OR52E6	11:51577359
RP11-826N14.2	5:175446487	-0.312	0.0276	1.25E-29	OR52K2	11:48385372
RP1-66C13.4	17:26206414	-0.0634	0.00933	1.08E-11	OR52K2	11:48385372

RP9	7:32953216	0.177	0.0223	2.07E-15	OR52K2	11:48350545
RPA2	1:28241083	0.458	0.0282	2.58E-59	OR52N5	11:56470579
RPL11	1:248601069	0.757	0.107	1.50E-12	OR52N5	11:48373976
RPL12	9:130221698	0.137	0.019	5.57E-13	OR52N5	11:48373976
RPL22L1	3:170578721	-0.197	0.0273	5.35E-13	OR52N5	11:48373976
RPL36AL	14:50095335	0.145	0.0189	1.69E-14	OR52N5	11:48388105
RPL9	4:39446549	-2.16	0.048	<1.0E-314	OR56A1	11:56470865
RPP21	6:30230518	-0.338	0.0401	3.49E-17	OR56A1	11:56470865
RPRD2	1:150374447	0.239	0.0168	6.30E-46	OR56A1	11:56470865
RPS27L	15:63441057	-0.415	0.0373	9.37E-29	OR56A1	11:56470865
RPS9	19:54709377	-0.131	0.0189	4.17E-12	OR56A1	11:56470865
RRP1B	21:45112939	-0.0731	0.00988	1.37E-13	OR5A1	11:58221171
RRP7A	22:42942809	0.311	0.0263	2.90E-32	OR5I1	11:55044296
RSPH10B2	7:61821630	-1.91	0.199	8.15E-22	OR5I1	11:55044296
RSPO4	20:1000072	-0.199	0.0197	5.44E-24	OR5I1	11:54774691
RTN4	2:55204028	-0.187	0.0152	8.77E-35	OR5K1	3:9891365
RWDD2B	21:30378191	-0.426	0.0203	8.95E-98	OR6S1	14:20763161
RWDD3	1:95674384	-0.235	0.0364	1.07E-10	OR6V1	7:142116439
RXRB	6:33088084	-0.106	0.0156	1.08E-11	OR6V1	7:142116439
S100A7	1:153430249	-0.171	0.0215	1.81E-15	OR6V1	7:142116439
SAAL1	11:18101078	-0.131	0.0176	9.83E-14	OR6V1	7:142116439
SAT2	17:7525548	0.175	0.0173	4.71E-24	OR6V1	7:142116439
SCGB3A1	5:179997625	0.372	0.0286	1.12E-38	OR7A10	19:15128250
SCIMP	17:5126207	-0.102	0.0139	2.17E-13	OR8D1	11:123784212
SCLY	2:238990388	-0.0904	0.0118	1.84E-14	OR8D1	11:123784212
SCN11A	3:39073029	0.0537	0.00759	1.49E-12	ORC6	16:46402985
SCN7A	2:167331064	-0.049	0.00726	1.49E-11	ORC6	16:46465847
SEC14L4	22:30879710	-0.0749	0.011	9.82E-12	ORC6	16:46402985
SELL	1:169637078	-0.19	0.0253	5.92E-14	P2RX3	11:56474191
SELM	22:31476288	-0.213	0.033	1.09E-10	P2RX5-TAX1BP3	17:41382047
SEMA5B	3:122642724	-0.203	0.0274	1.27E-13	P2RX5-TAX1BP3	17:28843496
SENP2	3:185349425	-0.0699	0.0109	1.43E-10	PADI4	1:17673102
SEPT1	16:30376959	-0.189	0.0208	1.02E-19	PADI4	1:17673102
SEPT12	16:4846533	0.47	0.0221	2.30E-100	PADI4	1:17673102
SEPT15	1:87368095	0.0993	0.0155	1.49E-10	PADI4	1:17673102
SERF1B	5:69298177	0.352	0.0395	5.04E-19	PAOX	10:135446144
SERPINC1	1:173893805	0.0863	0.00982	1.52E-18	PCNA	20:55294708
SETD4	21:37366582	-0.0956	0.0141	1.20E-11	PDZK1IP1	1:47392596
SGCA	17:48243736	-0.316	0.0165	9.40E-82	PDZK1IP1	1:47419215

SGK2	20:42197892	0.464	0.0483	7.50E-22	PEX11B	1:145247557
SGTB	5:64910392	-0.178	0.0251	1.33E-12	PEX11B	1:145194076
SH2B2	7:101928495	-0.288	0.0255	1.40E-29	PFAS	17:80989217
SH3BGR	21:40842460	0.142	0.0206	5.45E-12	PGAM5	12:133838700
SHC3	9:91628082	0.294	0.0211	3.96E-44	PHF5A	22:42445693
SHISA3	4:42412405	0.162	0.0224	4.75E-13	PI3	20:4391341
SHISA4	1:201805751	-0.184	0.0171	5.30E-27	PI3	20:4391341
SHPK	17:3509158	0.358	0.0201	5.81E-71	PIBF1	13:73979274
SIDT1	3:113321199	0.282	0.0326	5.14E-18	PIGX	3:197315059
SIGLEC1	20:29589323	-1.49	0.185	8.01E-16	PIGX	3:197315059
SLC13A3	20:45191364	-0.239	0.0255	7.08E-21	PJA2	5:10909104
SLC18A1	8:20033107	0.0195	0.003	8.03E-11	PLA2G2A	1:206698786
SLC1A7	1:53600889	0.836	0.0526	7.03E-57	PLA2G2A	1:206698786
SLC22A18	11:2940492	-0.744	0.0536	8.30E-44	PLA2G2A	1:207907085
SLC22A18AS	11:2924850	-0.267	0.0267	1.52E-23	PLEKHB2	2:133047399
SLC22A3	6:160850273	0.113	0.0112	6.16E-24	PLEKHB2	2:133047399
SLC25A1	22:19206499	-0.273	0.0197	1.14E-43	PLEKHS1	10:115115136
SLC25A2	5:140681648	0.0668	0.00605	2.41E-28	PNLIP	10:118840388
SLC25A24	1:108740419	-0.121	0.0145	7.13E-17	PNO1	2:67891593
SLC25A34	1:16058698	-0.272	0.023	2.86E-32	POLH	6:43729267
SLC25A51	9:37880134	0.196	0.0209	6.72E-21	POLR2J	7:102114424
SLC26A8	6:35976778	-0.173	0.0222	6.55E-15	POLR2J	7:102102164
SLC27A6	5:128302850	-0.0466	0.00727	1.46E-10	POMC	2:25384456
SLC2A14	12:8082367	0.0948	0.00756	4.53E-36	POTEC	18:15403165
SLC35A1	6:88181046	-0.209	0.0197	2.70E-26	POTEH	22:16880695
SLC35G5	8:11188532	-0.152	0.017	3.85E-19	POTEM	14:19011416
SLC37A3	7:140034294	-0.089	0.0125	1.08E-12	POTEM	14:19011416
SLC38A8	16:84074978	-0.556	0.0305	3.01E-74	POTEM	14:19011416
SLC39A10	2:196703028	-0.203	0.0251	6.08E-16	POTEM	14:19011416
SLC39A2	14:21466781	-0.0808	0.00593	2.82E-42	POTEM	14:19011416
SLC44A3	1:95302957	-0.327	0.0504	8.69E-11	PPA2	4:106288220
SLC5A4	22:32747715	0.238	0.0172	1.52E-43	PPA2	4:106286465
SLC5A7	2:108603628	-0.00708	0.000947	7.65E-14	PPA2	4:106288220
SLC7A9	19:33366829	-0.316	0.0258	1.72E-34	PPA2	4:106286947
SLCO4C1	5:101625154	-0.0413	0.00601	6.34E-12	PPIA	7:45392654
SLFN5	17:33544766	0.0898	0.00932	5.68E-22	PPP1R9A	7:95467086
SLN	11:107593050	-0.267	0.0341	4.88E-15	PRAMEF12	1:121363032
SLX1B	16:30166626	-0.448	0.0404	1.42E-28	PRAMEF26	1:12908500
SLX4	16:33941694	-2.29	0.239	9.56E-22	PRAMEF26	1:12908500

SMAD9	13:37489555	0.169	0.0254	2.86E-11	PRAMEF26	1:12919505
SMARCB1	22:24164598	-0.153	0.0195	4.29E-15	PRAMEF26	1:12919505
SMC1B	22:45833924	-0.0189	0.00217	3.05E-18	PRAMEF6	1:121141553
SMC2	9:106856910	-0.171	0.0141	7.54E-34	PRH1	12:118819493
SMIM22	16:4845532	-0.143	0.0137	1.66E-25	PRM1	16:10613536
SMOC2	6:169016615	0.22	0.028	3.93E-15	PROL1	4:7132371
SNED1	2:241979264	0.182	0.0273	2.62E-11	PRPF18	10:135446144
SNUPN	15:75750425	-0.105	0.0117	2.85E-19	PRRC2C	1:17229951
SNX17	2:27648590	0.13	0.0119	8.82E-28	PRSS1	7:142461940
SNX19	11:130786592	-0.201	0.0188	1.12E-26	PRSS1	7:142461921
SNX31	8:101676363	0.0882	0.0136	8.86E-11	PRSS1	7:142461921
SNX32	11:65595697	0.33	0.0351	5.37E-21	PRSS1	7:142461910
SNX8	7:142116439	-2.21	0.254	3.30E-18	PRSS1	7:142461921
SOHLH2	13:36799495	0.238	0.0103	3.96E-118	PRSS33	16:33941694
SPAG11B	8:8018749	0.0224	0.00332	1.51E-11	PRSS33	16:33941694
SPAG7	17:4914028	0.189	0.0295	1.49E-10	PRSS33	16:33941694
SPATA33	16:89710378	-0.149	0.0153	2.06E-22	PRSS33	16:33941694
SPATA5L1	15:45652926	0.115	0.0179	1.32E-10	PRSS33	16:33941694
SPATA7	14:88862529	-0.237	0.0213	9.30E-29	PRSS58	7:142113650
SPATC1L	21:48028060	-0.734	0.0507	1.69E-47	PRSS58	7:142113650
SPECC1	17:20311541	0.126	0.0115	6.18E-28	PSMB2	1:3577254
SPEF2	5:35682606	0.178	0.0147	9.48E-34	PSMB7	9:12739598
SPG21	15:65315211	-0.099	0.011	2.26E-19	PSMC2	7:102217297
SPINK2	4:57692773	0.326	0.0402	5.08E-16	PSPH	7:57093614
SPINK9	5:147733045	0.136	0.0191	1.08E-12	PTX3	3:156184172
SPPL3	12:121198299	-0.0941	0.00897	9.55E-26	PUM2	2:215147863
SPRY1	4:124325753	-0.159	0.0215	1.41E-13	PURB	7:45392654
SPRYD4	12:56863770	0.155	0.0147	5.40E-26	PURB	7:45392654
SPSB2	12:6987335	0.466	0.0208	3.61E-111	QTRTD1	3:113652214
SSR1	6:7292674	-0.0962	0.0121	1.86E-15	RAB28	4:138803729
ST20	15:80206511	-0.179	0.0218	2.19E-16	RAB2B	14:21981216
ST6GALNAC2	17:74574103	0.095	0.0145	5.69E-11	RAB2B	14:21072431
STEAP1B	7:22542138	0.18	0.0269	2.21E-11	RAB33B	4:139948061
STMN4	8:27103024	-0.182	0.0269	1.33E-11	RAB33B	4:139948061
STON1	2:48812387	0.102	0.0121	3.46E-17	RAC1	7:61821560
STRA13	17:79980756	0.184	0.0135	2.67E-42	RAC1	7:61821560
STRA6	15:74493886	0.102	0.0106	6.42E-22	RAC1	7:61821560
STRC	15:43995789	0.209	0.0285	2.24E-13	RAC1	7:61821560
STX16-NPEPL1	20:57263778	0.238	0.032	1.03E-13	RAC1	7:61821560

STX18	4:4435741	-0.105	0.0163	1.18E-10	RALY	20:31877204
STX6	1:180950126	0.0762	0.0102	7.98E-14	RANBP6	9:68400922
STXBP6	14:25414148	-0.124	0.0193	1.32E-10	RANBP6	9:68400922
STYXL1	7:75677216	0.262	0.0337	7.58E-15	RANBP6	9:68400922
SULT1A1	16:28626741	-0.208	0.0259	9.68E-16	RANBP6	9:68400922
SULT1C2	2:108913012	0.265	0.0101	9.90E-152	RANBP6	9:68400922
SUMF1	3:4403537	-0.186	0.0126	2.58E-49	RANBP9	6:145394013
SUPT3H	6:44774597	0.144	0.0137	7.69E-26	RAX	18:573269
SUPT4H1	17:56447952	-0.114	0.0177	1.19E-10	RAX2	19:27851743
SURF1	9:136268084	0.426	0.0235	1.93E-73	RAX2	19:27851743
SURF6	9:136196161	-0.215	0.0264	3.83E-16	RAX2	19:27851743
SUSD1	9:114927849	0.142	0.0189	5.77E-14	RAX2	19:27851743
SUSD2	22:24435954	-0.283	0.0283	1.52E-23	RAX2	19:27851743
SYCE1L	16:77233338	0.323	0.0316	1.59E-24	RBBP8NL	20:60996368
SYCE3	22:51001271	0.859	0.0534	3.19E-58	RBBP9	20:1917479
TACR3	4:104847521	0.0209	0.00302	4.50E-12	REG4	1:121128139
TAF1A	1:222731523	-0.103	0.0144	8.50E-13	RELB	19:45670248
TAF1C	16:84213965	-0.234	0.0168	4.25E-44	RESP18	2:220184857
TAP2	6:32796480	0.27	0.0269	1.05E-23	RESP18	2:220193396
TAS2R4	7:141455077	0.159	0.0142	4.21E-29	RESP18	2:220184857
TAS2R43	12:11218634	-0.017	0.00226	5.39E-14	RIBC2	22:45830097
TAS2R60	7:142172531	-0.0691	0.00969	9.96E-13	RIMS2	8:105215989
TBC1D24	16:33941694	-1.64	0.226	3.97E-13	RMI2	16:11439303
TBC1D26	17:15618623	0.244	0.0235	2.96E-25	RMI2	16:11439303
TBC1D3	17:36412149	-0.424	0.043	6.18E-23	RMND1	6:151694262
TBC1D3C	17:34732888	0.905	0.0643	5.44E-45	RNASE12	14:21981216
TBC1D3F	17:36350337	1.65	0.188	1.68E-18	RNASE12	14:21981216
TBC1D3H	17:34732888	0.52	0.0429	8.15E-34	RNF125	18:29789212
TBC1D9B	5:179334857	0.182	0.0182	1.52E-23	RNF186	1:206514654
TBX5	12:114699538	0.0516	0.00775	2.77E-11	RNF186	1:206514654
TBX6	16:30078492	0.139	0.0198	2.22E-12	RNF186	1:206514654
TC2N	14:92286857	-0.146	0.01	2.81E-48	RNF39	6:29864630
TCEA3	1:23751140	0.294	0.0232	8.41E-37	RNF4	4:3597137
TCEB2	16:2821988	-0.342	0.0229	1.97E-50	RNF4	4:3597137
TCF19	6:31223601	0.215	0.025	7.97E-18	RNF4	4:3597137
TCFL5	20:61500478	-0.139	0.0202	5.94E-12	RNF4	4:3597137
TDGF1	3:46619238	0.368	0.0226	1.30E-59	RNF4	4:3597137
TDO2	4:156752651	-0.208	0.0287	4.25E-13	RNF6	13:26551751
TDRD5	1:179560718	0.182	0.0125	5.05E-48	RP11-1026M7.2	5:177221743

TDRD6	6:46624656	-0.145	0.0121	4.34E-33	RP11-1026M7.2	5:177221743
TECPR1	7:97849648	0.138	0.0181	2.45E-14	RP11-1099M24.7	17:78603090
TECTB	10:113984760	0.0682	0.00868	3.93E-15	RP11-1099M24.7	17:78603090
TEN1	17:74015354	0.172	0.0232	1.23E-13	RP11-169F17.1	18:69918736
TESK2	1:45826272	0.16	0.0197	4.59E-16	RP11-196G11.1	16:31670496
TEX29	13:111980150	0.526	0.0356	2.11E-49	RP11-196G11.1	16:31670496
TEX40	11:64048912	0.403	0.0352	2.38E-30	RP11-196G11.1	16:31203669
TFB1M	6:155635637	-0.111	0.0134	1.20E-16	RP11-297N6.4	8:12452575
TGM1	14:24734645	0.204	0.025	3.35E-16	RP11-318A15.7	17:74926652
TGM3	20:2217291	0.0403	0.00606	2.93E-11	RP11-318A15.7	17:74586504
THEM4	1:151920862	-0.125	0.0166	5.07E-14	RP11-385D13.1	17:15588
THG1L	5:157156103	0.11	0.0123	3.78E-19	RP11-529K1.3	16:70001611
THNSL2	2:88469863	0.959	0.0436	3.19E-107	RP11-536G4.1	12:97166022
THOC3	5:175389016	-0.493	0.0353	2.51E-44	RP11-644F5.10	12:56115585
TIMM10	11:57283988	-0.314	0.0187	2.82E-63	RP11-664I21.6	11:124737216
TIMM21	18:71718499	-0.21	0.0205	1.26E-24	RP11-664I21.6	11:12400992
TIPIN	15:66744369	0.319	0.0311	1.10E-24	RP11-738G5.2	8:49889797
TIPRL	1:168166616	-0.152	0.0129	4.78E-32	RP11-826N14.2	5:175446487
TIRAP	11:126151761	-0.14	0.0179	5.23E-15	RP11-977G19.10	12:56952013
TMC2	20:25904151	-1.11	0.113	8.96E-23	RP11-998D10.1	14:22483999
TMEM11	17:21526192	1.63	0.22	1.27E-13	RPAIN	17:45384410
TMEM121	14:105993258	-0.144	0.0218	3.96E-11	RPGRIP1L	16:54239414
TMEM126A	11:85347415	-0.125	0.0189	3.75E-11	RPGRIP1L	16:54604728
TMEM132E	17:32887254	-0.167	0.0222	5.37E-14	RPL11	1:248601069
TMEM156	4:39025844	0.126	0.018	2.56E-12	RPL11	1:235844
TMEM17	2:62731754	-0.148	0.0203	3.08E-13	RPL22	1:5735297
TMEM171	5:72412602	0.185	0.0134	2.35E-43	RPL26	17:75535140
TMEM200B	1:29414108	-0.148	0.0176	4.13E-17	RPL26	17:80989217
TMEM213	7:138029183	-0.0379	0.0056	1.31E-11	RPL27	17:41382047
TMEM215	9:32904965	0.125	0.00937	1.35E-40	RPL27A	11:94860472
TMEM236	10:17812604	0.012	0.00177	1.20E-11	RPL27A	11:77155274
TMEM244	6:130157007	-0.0829	0.00871	1.77E-21	RPL32	3:125567571
TMEM245	9:111772918	-0.111	0.0119	1.08E-20	RPL32	3:129058118
TMEM255B	13:114502416	0.199	0.023	5.05E-18	RPL9	4:39446549
TMOD3	15:52189201	-0.218	0.015	7.45E-48	RPL9	4:39446549
TMPRSS5	11:113582504	0.628	0.0356	1.20E-69	RPL9	4:39455378
TMPRSS7	3:111731195	-0.0227	0.00321	1.53E-12	RPRD2	1:149582977
TMPRSS9	19:23936403	-1.4	0.0919	2.10E-52	RPS18	6:32485527
TNFRSF10C	8:22941873	0.286	0.0228	4.30E-36	RPS27L	15:63441057

TNFRSF13C	22:42338152	0.114	0.0162	1.96E-12	RSPH10B2	7:61821630
TNN	1:175062557	0.0286	0.00288	3.07E-23	RSPH10B2	7:61821630
TNNI3	19:55681833	0.391	0.0335	1.78E-31	RSPH10B2	7:61821630
TNPO3	7:128595149	-0.091	0.0131	3.74E-12	RSPH10B2	7:61821630
TOM1L1	17:53009971	-0.138	0.0171	7.02E-16	RSPH10B2	7:61821630
TOMM40	19:45398264	-0.12	0.0169	1.24E-12	RTFDC1	20:55514750
TOMM7	7:22861038	-0.167	0.0164	2.36E-24	RUNDC1	17:41382433
TOP3B	22:22290670	-0.226	0.0265	1.49E-17	RWDD2B	21:30400110
TP53TG3	16:33395983	-0.118	0.0181	7.06E-11	RWDD2B	21:30378191
TP53TG3D	16:32411542	-0.179	0.0248	5.29E-13	SAA1	11:18141982
TPTE2	13:19947076	0.0812	0.00915	7.04E-19	SAA1	11:18141982
TRAM2	6:52245945	-0.0707	0.0105	1.66E-11	SAA2	11:1902983
TRAPPC4	11:118893878	0.163	0.0148	3.29E-28	SAA2	11:1902983
TREML4	6:41196104	-0.0206	0.0022	7.70E-21	SAA2	11:19218770
TREX1	3:48508585	-0.313	0.0263	1.17E-32	SAA4	11:1902983
TRIM35	8:27167942	-0.0818	0.0124	4.20E-11	SAA4	11:1902983
TRIM43	2:96603973	-0.0589	0.00775	2.96E-14	SAFB	19:52486363
TRIM49B	11:48363026	-0.139	0.0114	3.39E-34	SAFB	19:55683246
TRIM58	1:248011866	0.138	0.0171	7.02E-16	SCGB1C1	11:1017968
TRIM63	1:26580790	0.124	0.0129	7.09E-22	SCGB3A1	5:179997625
TRIM69	15:45021766	-0.213	0.0286	9.51E-14	SCNN1A	12:69986
TRIML1	4:189058364	0.0477	0.00381	5.83E-36	SCNN1A	12:73752709
TRMT5	14:61579863	-0.0593	0.00905	5.66E-11	SENP5	3:197315059
TRPM1	15:31394868	-0.127	0.00879	2.57E-47	SENP5	3:197387516
TRPV2	17:16318932	-0.446	0.0283	5.89E-56	SEPT12	16:4847524
TRPV3	17:3447317	0.125	0.018	3.80E-12	SEPT12	16:4846533
TSPAN11	12:31049763	0.518	0.0328	3.49E-56	SETD6	16:58525490
TSTD1	1:161008535	0.255	0.0367	3.70E-12	SETD6	16:58515425
TTC21B	2:166714095	-0.154	0.0168	4.88E-20	SF3B14	2:241659876
TTC32	2:20090677	0.433	0.0383	1.23E-29	SF3B14	2:241659876
TTC5	14:20767618	-0.228	0.0163	1.85E-44	SF3B4	1:149582977
TTI1	20:36628578	-0.0755	0.0114	3.52E-11	SF3B4	1:149582977
TTLL6	17:47045862	0.123	0.0164	6.38E-14	SF3B5	6:145416470
TUBA3D	2:132234986	-0.255	0.0345	1.45E-13	SF3B5	6:145416470
TVP23C	17:15406661	-0.239	0.0259	2.76E-20	SGCA	17:48243736
TYW5	2:200850402	0.0691	0.00986	2.42E-12	SGCA	17:48243736
UBALD2	17:74273165	-0.24	0.0228	6.53E-26	SHC3	9:91628082
UBE2R2	9:33914412	-0.123	0.0149	1.52E-16	SHPK	17:3514598
UBOX5	20:25904151	-1.02	0.145	2.00E-12	SHPK	17:3509158

UBQLN3	11:48346090	-0.104	0.015	4.11E-12	SI	3:16435640
UFSP2	4:186347055	0.136	0.0141	5.14E-22	SI	3:163950899
UGT2B17	4:69534622	0.0299	0.00338	9.06E-19	SI	3:16435640
UGT2B4	4:70249082	-0.096	0.0135	1.15E-12	SIGLEC1	20:29439122
UHRF1BP1	6:34745703	0.0867	0.00843	8.26E-25	SIGLEC1	20:29612969
ULBP2	6:150183737	0.114	0.0152	6.38E-14	SIGLEC1	20:29611972
ULK4	3:41971728	0.239	0.0223	8.43E-27	SIGLEC1	20:29637426
UPK1A	19:36177001	0.126	0.012	8.64E-26	SKI	1:145247557
UPK3B	7:76124685	0.289	0.0161	4.77E-72	SLC15A5	12:16903899
UPK3BL	7:102319015	0.767	0.0731	9.35E-26	SLC1A1	9:44898183
URB1	21:33681770	0.127	0.0165	1.39E-14	SLC1A1	9:44898183
USP22	17:21526192	-1.44	0.181	1.78E-15	SLC1A1	9:44898183
USP6	17:4980987	0.179	0.0134	1.06E-40	SLC1A1	9:44898183
UST	6:149239806	0.162	0.0197	1.98E-16	SLC1A7	1:53600889
UTP15	5:72862984	-0.0987	0.0152	8.39E-11	SLC22A10	11:62226112
UTP18	17:49340101	0.0788	0.0114	4.77E-12	SLC23A2	20:51909434
VCPKMT	14:50540085	-0.13	0.02	8.03E-11	SLC27A6	5:129134418
VILL	3:38036807	-0.458	0.0304	2.72E-51	SLC28A2	15:46058456
VN1R1	19:57993074	-0.274	0.0167	1.70E-60	SLC28A2	15:46058456
VPREB3	22:24102408	0.446	0.0439	3.01E-24	SLC30A6	2:31540296
VWA5B2	3:183976694	-0.22	0.032	6.20E-12	SLC38A8	16:84074978
VWDE	7:12406186	0.157	0.0118	2.16E-40	SLX4	16:33941694
WARS2	1:119577837	-0.0902	0.0107	3.46E-17	SLX4	16:33991563
WBP2NL	22:42402399	-0.136	0.0199	8.25E-12	SLX4	16:33941694
WBSCR27	7:73246461	-0.537	0.0245	1.74E-106	SLX4	16:33941694
WDR27	6:170073543	-0.191	0.0165	5.47E-31	SLX4	16:33941694
WDR48	3:39125293	-0.0778	0.012	8.97E-11	SMARCD3	7:15082341
WDR52	3:113262057	-0.118	0.0168	2.16E-12	SMIM20	4:2524559
WDR60	7:158676289	-0.337	0.0253	1.77E-40	SMU1	9:33383540
WDR88	19:33635043	0.0898	0.0124	4.42E-13	SNRPD3	22:25056098
WDR96	10:105933138	0.116	0.0165	2.06E-12	SNRPD3	22:25056128
WDSUB1	2:160144140	-0.131	0.0203	1.10E-10	SNRPD3	22:25056128
WDYHV1	8:124446796	-0.148	0.0162	6.49E-20	SNRPE	1:204092502
WFDC3	20:44402115	0.436	0.0209	1.20E-96	SNX24	5:122109100
WI2-3308P17.:	1:120576209	-0.185	0.0114	3.19E-59	SNX8	7:142116439
WIPI1	17:66441651	-0.219	0.025	1.95E-18	SNX8	7:142116439
WNT3	17:44908263	-1.49	0.0306	<1.0E-314	SNX8	7:142116439
WT1	11:32502239	0.0985	0.00832	2.46E-32	SNX8	7:142116439
WWOX	16:78124987	-0.133	0.0166	1.13E-15	SNX8	7:142116439

XKR3	22:17235076	-0.0803	0.0113	1.19E-12	SOHLH2	13:36792650
XKR9	8:71677839	0.13	0.0128	3.11E-24	SPAG11B	8:7913884
XRCC2	7:152104360	2.12	0.13	8.70E-60	SPAG11B	8:7370143
XRCC6BP1	12:58337132	-0.225	0.0166	7.48E-42	SPAG11B	8:8018749
XRRA1	11:74618865	0.687	0.0215	4.83E-224	SPAG11B	8:8018749
YBEY	21:47726332	-0.461	0.0294	2.06E-55	SPATA2L	16:90158184
YBX2	17:7193255	-0.13	0.0199	6.46E-11	SPATA31A1	9:39377268
YEATS4	12:69658161	0.341	0.0353	4.46E-22	SPATA31A2	9:39944249
YWHAB	20:43534903	-0.254	0.022	7.78E-31	SPATA31A5	9:41915956
ZADH2	18:72912469	-0.107	0.0115	1.35E-20	SPCS3	4:178212874
ZAP70	2:98277804	-0.14	0.0153	5.68E-20	SPCS3	4:17740708
ZBED6CL	7:150033882	-0.0908	0.01	1.09E-19	SPEF2	5:35647998
ZDHHC17	12:77230119	-0.126	0.0119	3.38E-26	SPHAR	1:228842933
ZDHHC4	7:61821560	1.38	0.208	3.25E-11	SPIRE2	16:90158184
ZFP57	6:29643877	0.103	0.00478	5.51E-103	SPIRE2	16:90158184
ZFP69	1:41026035	-0.135	0.0132	1.50E-24	SPIRE2	16:90158184
ZFP82	19:36853523	-0.129	0.0111	3.20E-31	SPRR2F	1:15375046
ZMAT3	3:178758174	-0.293	0.0244	3.22E-33	SPSB2	12:6987335
ZMYND12	1:42932991	-0.186	0.0269	4.70E-12	SPSB2	12:6987981
ZNF12	7:61821560	-1.91	0.147	1.34E-38	SRCAP	16:29961344
ZNF148	3:125076251	-0.134	0.0188	1.02E-12	SRFBP1	5:120768765
ZNF155	19:44488352	-0.199	0.0245	4.57E-16	SRP14	15:40446718
ZNF180	19:44995037	-0.0965	0.0126	1.88E-14	STARD3NL	7:37881065
ZNF200	16:33941694	-2	0.17	5.93E-32	STX2	12:130689914
ZNF202	11:123610548	-0.0801	0.0117	7.59E-12	SULT1C2	2:108904340
ZNF232	17:5154016	0.331	0.0259	2.12E-37	SULT1C2	2:108904340
ZNF253	19:19995472	-0.169	0.0231	2.55E-13	SUMO2	17:7264899
ZNF263	16:33941694	-2.14	0.203	5.54E-26	SURF1	9:136212369
ZNF268	12:133729259	0.199	0.0176	1.21E-29	SURF1	9:136218590
ZNF276	16:89802965	0.119	0.0157	3.47E-14	SYNRG	17:36306936
ZNF286A	17:15594107	0.118	0.0152	8.28E-15	TADA2A	17:36306936
ZNF354C	5:178504338	-0.0714	0.0103	4.15E-12	TAF1C	16:84215314
ZNF391	6:27353197	0.124	0.0159	6.25E-15	TAF8	6:421206
ZNF404	19:44371210	-0.273	0.0206	4.37E-40	TAOK2	16:29086987
ZNF439	19:11977572	-0.124	0.0153	5.29E-16	TAS2R41	7:142460203
ZNF467	7:149472112	-0.323	0.0297	1.51E-27	TAS2R41	7:142460216
ZNF471	19:57042178	-0.117	0.017	5.89E-12	TAS2R41	7:142460203
ZNF500	16:46402985	1.2	0.173	4.02E-12	TAS2R60	7:142143328
ZNF501	3:44883971	0.177	0.0196	1.71E-19	TAS2R60	7:142172531

ZNF502	3:44762830	0.2	0.0159	2.77E-36
ZNF514	2:95901265	0.183	0.0199	3.72E-20
ZNF534	19:52928725	0.437	0.0335	6.81E-39
ZNF577	19:52358593	-0.366	0.0222	4.59E-61
ZNF580	19:56211995	0.248	0.0255	2.35E-22
ZNF593	1:26497340	-0.289	0.0213	6.19E-42
ZNF660	3:44651120	-0.121	0.0158	1.89E-14
ZNF670	1:247192182	-0.0949	0.0104	7.17E-20
ZNF717	3:75270860	1.46	0.186	4.18E-15
ZNF786	7:148799125	0.223	0.0155	6.24E-47
ZNF787	19:56606584	-0.188	0.0234	9.42E-16
ZNF839	14:102902709	-0.15	0.0171	1.76E-18
ZNHIT6	1:86253805	-0.0901	0.0115	4.70E-15
ZNRD1	6:29872510	0.289	0.0356	4.74E-16
ZP3	7:76303260	1.59	0.066	3.11E-128
ZSCAN31	6:28193786	0.374	0.0288	1.47E-38
ZSWIM7	17:15879910	-0.442	0.0147	1.27E-198
ZXDC	3:126179492	0.146	0.0158	2.45E-20

TAS2R60	7:142172531
TAS2R60	7:142172531
TAS2R60	7:142172531
TBC1D15	12:73304072
TBC1D20	20:142909
TBC1D20	20:10945743
TBC1D24	16:33941694
TBC1D24	16:33941694
TBC1D24	16:33941694
TBC1D24	16:33991563
TBC1D24	16:33991563
TBC1D3C	17:34732888
TBC1D9B	5:179352982
TCEB2	16:2821988
TCL1A	14:95313195
TCL1A	14:96355146
TCN1	11:60114141
TFF1	21:44199048
THNSL2	2:88469863
THNSL2	2:88469863
THOC3	5:175386586
THRAP3	1:3577254
TIMM10	11:57283988
TIMM10B	11:64831013
TM4SF4	3:15004731
TMC2	20:26109209
TMC2	20:26109209
TMC2	20:26109209
TMC2	20:26109209
TMC2	20:26109209
TMED4	7:45392654
TMED4	7:45392654
TMEM105	17:78340119
TMEM11	17:21526192
TMEM11	17:21526192
TMEM11	17:21526192
TMEM11	17:21526192
TMEM11	17:21526192
TMEM126B	11:85577403

TMEM184C	4:149032159
TMEM184C	4:149003618
TMEM213	7:138029144
TMEM213	7:138029183
TMEM213	7:138029261
TMEM213	7:138029261
TMEM230	20:52788792
TMEM230	20:54638409
TMEM82	1:17007206
TMOD3	15:52252799
TMOD3	15:52252799
TMPRSS15	21:18951008
TMPRSS15	21:18951008
TMPRSS4	11:117368158
TMPRSS5	11:113582504
TMPRSS9	19:23936403
TMPRSS9	19:23936403
TMPRSS9	19:23936403
TMPRSS9	19:23936403
TMPRSS9	19:23936403
TNF	6:32440603
TNFAIP2	14:102639116
TNFAIP6	2:152640075
TNFRSF9	1:89457587
TNIP1	5:149501688
TNIP3	4:12264899
TNN	1:175062557
TNRC18	7:57613015
TNRC18	7:57613015
TOMM6	6:421206
TOMM6	6:412236
TOP2B	3:24995765
TOP2B	3:24995765
TPST2	22:27773148
TREML4	6:40921531
TRIM43	2:96613853
TRIM49B	11:48363026
TRIM49B	11:48363026
TRIM49B	11:48363026

TRIM49B	11:48379997
TRIM49B	11:49817445
TRIM72	16:31203669
TRIM72	16:30943214
TRIML1	4:189065574
TRIML1	4:189058364
TRMT10B	9:38568
TRPM1	15:31394868
TRPV2	17:16318932
TSPAN11	12:31049763
TSPAN3	15:77310726
TTC26	7:138604993
TUFM	16:29086987
TXNDC15	5:135053175
TXNDC15	5:134643060
UBD	6:2870553
UBD	6:28894987
UBQLN3	11:48346090
UBQLN3	11:48346090
UBQLN3	11:48346090
UBQLN3	11:48346090
UBQLN3	11:48346090
UBXN2A	2:235239010
UBXN2A	2:237950969
UGT2B11	4:70246843
UGT2B11	4:70246843
UGT2B4	4:70249082
UGT2B4	4:70249082
UGT2B4	4:70249082
UGT2B4	4:70249082
UGT2B4	4:70249082
UPK3B	7:76127071
UPK3B	7:76129758
USH2A	1:216910011
USP22	17:21526192
USP22	17:21526192
USP22	17:21526192
USP22	17:21526192
USP22	17:21526192

USP41	22:21483262
UTP11L	1:39284122
VILL	3:38038329
VILL	3:38044143
VMAC	19:55325816
VMAC	19:55683246
VN1R1	19:57999713
VN1R1	19:58060538
VPS35	16:46402985
VWDE	7:12386207
WDR45B	17:80989217
WDR45B	17:80989217
WDR60	7:158674521
WFDC12	20:44056352
WFDC12	20:44729409
WFDC3	20:44438974
WFDC3	20:44413410
WHAMM	15:82996209
WI2-3308P17.2	1:120576209
WI2-3308P17.2	1:120576209
WI2-3308P17.2	1:120576209
WI2-3308P17.2	1:120576209
WI2-3308P17.2	1:120576209
WNT1	12:49470128
WNT3	17:44908263
WNT3	17:44908263
WNT3	17:44908263
XCL2	1:16914503
XKR3	22:16854249
XKR3	22:17235076
XKR3	22:17235076
XKR3	22:17235076
XKR3	22:17235076
XKR9	8:7066735
XRCC2	7:152104360
XRCC2	7:152104360
XRCC2	7:152104360
XRCC2	7:152104360
XRCC2	7:152104360

XRCC5	2:217092097
XRRA1	11:74618865
XRRA1	11:74618865
XRRA1	11:74618865
YBEY	21:47622981
ZC3HAV1L	7:139713109
ZC3HAV1L	7:138899843
ZDHHC4	7:61821560
ZDHHC4	7:61821630
ZDHHC4	7:61821630
ZDHHC4	7:61821630
ZFAND6	15:81387204
ZFP30	19:3861116
ZFP57	6:29642978
ZFYVE20	3:141720388
ZMYM4	1:3577254
ZNF101	19:19096834
ZNF101	19:19714991
ZNF12	7:61821560
ZNF12	7:61821560
ZNF12	7:61821560
ZNF12	7:61821560
ZNF12	7:61821560
ZNF200	16:33941694
ZNF200	16:33991563
ZNF200	16:33941694
ZNF200	16:33941694
ZNF200	16:33941694
ZNF212	7:148086823
ZNF253	19:19714991
ZNF263	16:33991563
ZNF263	16:33941694
ZNF263	16:33941694
ZNF263	16:33941694
ZNF263	16:33941694
ZNF282	7:148086823
ZNF282	7:148645341
ZNF346	5:177157240
ZNF346	5:177239304

ZNF383	19:3861116
ZNF492	19:23818728
ZNF492	19:23649063
ZNF500	16:46402985
ZNF500	16:46402985
ZNF500	16:46402985
ZNF527	19:3861116
ZNF529	19:36635239
ZNF529	19:36170821
ZNF534	19:52925539
ZNF554	19:24533045
ZNF554	19:27866520
ZNF554	19:27866520
ZNF555	19:27866520
ZNF555	19:24531262
ZNF566	19:36635239
ZNF577	19:52352006
ZNF681	19:24533491
ZNF70	22:25056098
ZNF70	22:25056098
ZNF70	22:25056098
ZNF709	19:119541
ZNF717	3:75270860
ZNF717	3:75270860
ZNF717	3:75270860
ZNF717	3:75270860
ZNF717	3:75270860
ZNF721	4:103860868
ZNF74	22:21522286
ZNF74	22:21522286
ZNF747	16:29961344
ZNF747	16:30943214
ZNF783	7:148086823
ZNF783	7:148592640
ZNF785	16:29961344
ZNF785	16:31203669
ZNF785	16:31203669
ZNF786	7:148869277
ZNF829	19:36635239

ZNF829	19:36635239
ZNF843	16:32364540
ZNF843	16:31203669
ZNF850	19:36635239
ZNF850	19:36635239
ZP3	7:76060232
ZP3	7:76060232
ZP3	7:76060232
ZSWIM7	17:15879910
ZSWIM7	17:15879344
ZSWIM7	17:15879910

AP (brain) ct-eQTLs

Cell-type	Beta	Std Error	P-value
Neurons	-0.181	0.0263	5.90E-12
Microglia	-0.473	0.0694	9.39E-12
Endothelial cells	2.18	0.287	3.06E-14
Endothelial cells	1.1	0.155	1.28E-12
Endothelial cells	-1.1	0.163	1.49E-11
Microglia	-0.18	0.0258	3.02E-12
Microglia	0.299	0.0353	2.45E-17
Microglia	0.0372	0.00393	2.92E-21
Endothelial cells	0.0573	0.00645	6.47E-19
Neurons	0.0314	0.00374	4.63E-17
Oligodendroglia	0.102	0.0124	1.94E-16
Endothelial cells	-1.88	0.271	4.00E-12
Microglia	-0.0862	0.01	6.70E-18
Oligodendroglia	0.294	0.0366	9.53E-16
Oligodendroglia	-0.214	0.00941	1.73E-114
Astrocytes	-0.214	0.00942	3.00E-114
Microglia	-0.213	0.00941	1.94E-113
Endothelial cells	-0.214	0.00948	7.83E-113
Neurons	-0.213	0.00945	1.70E-112
Neurons	0.36	0.0284	8.03E-37
Microglia	0.32	0.0279	1.88E-30
Microglia	-0.0339	0.00434	5.67E-15
Astrocytes	0.0916	0.0122	5.99E-14
Neurons	-0.0307	0.0046	2.49E-11
Microglia	-0.168	0.0239	2.08E-12
Endothelial cells	-0.153	0.0229	2.37E-11
Microglia	-0.401	0.0123	3.89E-233
Astrocytes	-1.91	0.0629	1.56E-202
Endothelial cells	-0.81	0.0409	2.73E-87
Oligodendroglia	-1.64	0.0854	3.44E-82
Neurons	-0.344	0.0242	7.41E-46
Oligodendroglia	-7.69	0.733	9.49E-26
Microglia	-0.265	0.0317	6.29E-17
Endothelial cells	-1.89	0.265	9.89E-13
Neurons	-0.187	0.0267	2.49E-12

Astrocytes	0.542	0.0483	3.20E-29
Oligodendroglia	-0.227	0.0309	2.04E-13
Neurons	0.0595	0.00868	7.14E-12
Neurons	-0.851	0.103	1.43E-16
Endothelial cells	-0.756	0.101	7.15E-14
Neurons	-2.14	0.304	1.93E-12
Endothelial cells	-3.82	0.565	1.37E-11
Astrocytes	-0.749	0.0601	1.19E-35
Microglia	-0.202	0.0238	2.11E-17
Neurons	-0.195	0.0237	1.91E-16
Endothelial cells	0.425	0.0423	9.44E-24
Neurons	0.694	0.0978	1.28E-12
Neurons	-0.595	0.086	4.56E-12
Microglia	-0.841	0.0733	1.79E-30
Neurons	-0.741	0.0711	1.97E-25
Endothelial cells	-0.802	0.12	2.34E-11
Microglia	1.05	0.123	1.38E-17
Neurons	0.864	0.127	1.02E-11
Endothelial cells	2.17	0.324	2.12E-11
Endothelial cells	0.673	0.098	6.54E-12
Neurons	-0.721	0.101	9.43E-13
Endothelial cells	1.18	0.137	7.11E-18
Neurons	-0.662	0.094	1.89E-12
Microglia	-2.51	0.366	6.99E-12
Endothelial cells	0.0772	0.00856	1.90E-19
Neurons	-0.101	0.0137	1.68E-13
Endothelial cells	0.23	0.0329	2.73E-12
Neurons	0.164	0.0235	2.98E-12
Endothelial cells	1.09	0.14	6.93E-15
Neurons	0.627	0.0865	4.21E-13
Neurons	-0.206	0.0285	4.90E-13
Microglia	-0.204	0.0284	6.81E-13
Astrocytes	-0.204	0.0285	8.19E-13
Oligodendroglia	-0.204	0.0285	8.19E-13
Endothelial cells	-0.203	0.0285	1.06E-12
Neurons	-2.34	0.249	5.58E-21
Endothelial cells	-2.82	0.337	5.86E-17
Neurons	-1.06	0.0323	3.33E-236
Microglia	-0.89	0.0397	2.62E-111

Oligodendroglia	0.548	0.0612	3.42E-19
Microglia	-0.433	0.0589	1.96E-13
Astrocytes	-2.48	0.341	3.52E-13
Oligodendroglia	1.81	0.262	4.90E-12
Endothelial cells	0.524	0.0549	1.37E-21
Neurons	0.258	0.0311	1.08E-16
Endothelial cells	0.167	0.0125	1.04E-40
Neurons	-0.0671	0.00872	1.42E-14
Neurons	0.337	0.0404	7.33E-17
Endothelial cells	0.48	0.0646	1.08E-13
Neurons	0.475	0.0454	1.28E-25
Microglia	0.534	0.0571	8.60E-21
Oligodendroglia	1.89	0.256	1.55E-13
Oligodendroglia	0.134	0.0195	6.34E-12
Microglia	-0.831	0.105	2.49E-15
Astrocytes	-0.827	0.107	1.08E-14
Oligodendroglia	-0.823	0.107	1.45E-14
Neurons	-1	0.13	1.45E-14
Endothelial cells	-0.826	0.108	2.04E-14
Microglia	0.628	0.0788	1.59E-15
Microglia	0.833	0.0441	1.41E-79
Neurons	0.741	0.0441	2.33E-63
Endothelial cells	0.682	0.0756	1.86E-19
Endothelial cells	0.26	0.0308	3.13E-17
Oligodendroglia	0.134	0.0201	2.62E-11
Microglia	0.349	0.0441	2.50E-15
Endothelial cells	0.63	0.0869	4.18E-13
Neurons	0.651	0.0524	1.94E-35
Microglia	0.854	0.0846	5.84E-24
Neurons	-1.5	0.211	1.17E-12
Oligodendroglia	-2.17	0.313	4.12E-12
Endothelial cells	-2.12	0.309	6.85E-12
Astrocytes	-2.14	0.315	1.09E-11
Microglia	-2.03	0.3	1.32E-11
Endothelial cells	2.09	0.279	6.83E-14
Microglia	-2.25	0.313	6.55E-13
Neurons	-2.05	0.289	1.31E-12
Endothelial cells	1.02	0.151	1.43E-11
Microglia	-0.04	0.00449	5.16E-19

Neurons	0.0493	0.00561	1.52E-18
Neurons	0.115	0.0135	1.62E-17
Oligodendroglia	-0.326	0.0466	2.64E-12
Endothelial cells	-0.585	0.0746	4.44E-15
Microglia	0.277	0.0362	1.98E-14
Neurons	0.289	0.0427	1.30E-11
Neurons	-0.679	0.093	2.85E-13
Endothelial cells	-1.62	0.175	2.10E-20
Neurons	-0.715	0.107	2.35E-11
Astrocytes	-0.207	0.0306	1.34E-11
Microglia	-0.208	0.0309	1.68E-11
Neurons	-0.208	0.0309	1.68E-11
Oligodendroglia	-0.205	0.0308	2.82E-11
Neurons	-0.46	0.0654	2.01E-12
Endothelial cells	1.94	0.199	1.87E-22
Neurons	0.691	0.0897	1.32E-14
Endothelial cells	0.819	0.118	3.90E-12
Endothelial cells	-1.05	0.141	9.56E-14
Neurons	-0.306	0.0451	1.16E-11
Neurons	-1.09	0.154	1.46E-12
Neurons	-2.96	0.284	1.96E-25
Endothelial cells	-2.88	0.284	3.64E-24
Oligodendroglia	-2.9	0.287	5.27E-24
Microglia	-2.85	0.284	1.07E-23
Astrocytes	-2.83	0.286	4.37E-23
Endothelial cells	1.83	0.231	2.34E-15
Neurons	-0.378	0.0492	1.55E-14
Microglia	-0.36	0.0524	6.41E-12
Microglia	-1.6	0.196	3.26E-16
Astrocytes	-1.57	0.199	3.04E-15
Oligodendroglia	-1.6	0.203	3.23E-15
Neurons	-1.62	0.211	1.62E-14
Endothelial cells	-1.42	0.203	2.65E-12
Endothelial cells	0.0811	0.00995	3.62E-16
Neurons	-0.652	0.0798	3.07E-16
Endothelial cells	-0.832	0.12	4.11E-12
Neurons	0.488	0.0714	8.22E-12
Microglia	-0.134	0.0144	1.33E-20
Neurons	0.117	0.0176	2.98E-11

Endothelial cells	1.55	0.223	3.64E-12
Neurons	0.121	0.0104	2.75E-31
Microglia	0.0936	0.0119	3.67E-15
Endothelial cells	0.828	0.107	1.01E-14
Oligodendroglia	-0.265	0.0397	2.47E-11
Endothelial cells	-0.973	0.13	7.18E-14
Neurons	0.411	0.0532	1.11E-14
Endothelial cells	0.625	0.0917	9.38E-12
Microglia	-2.84	0.318	4.23E-19
Oligodendroglia	-2.82	0.352	1.13E-15
Astrocytes	-2.79	0.349	1.30E-15
Neurons	3.69	0.467	2.76E-15
Endothelial cells	8.96	1.17	1.89E-14
Endothelial cells	4.55	0.398	2.89E-30
Neurons	1.44	0.183	3.58E-15
Astrocytes	61.2	8.18	7.34E-14
Microglia	-0.137	0.0161	1.75E-17
Oligodendroglia	-0.394	0.0586	1.77E-11
Endothelial cells	0.456	0.0641	1.13E-12
Astrocytes	-1.43	0.189	3.84E-14
Endothelial cells	-0.511	0.0748	8.40E-12
Astrocytes	0.146	0.0203	6.38E-13
Endothelial cells	0.934	0.114	2.55E-16
Oligodendroglia	-13	0.991	2.59E-39
Neurons	1.35	0.129	1.25E-25
Microglia	-2.91	0.311	8.21E-21
Endothelial cells	4.02	0.534	5.15E-14
Astrocytes	-0.24	0.0338	1.24E-12
Endothelial cells	2.37	0.319	1.09E-13
Neurons	0.423	0.0544	7.50E-15
Microglia	0.452	0.0641	1.77E-12
Astrocytes	7.56	0.808	8.25E-21
Microglia	-6.64	0.83	1.24E-15
Endothelial cells	-4.09	0.525	6.68E-15
Neurons	381	50.9	7.14E-14
Oligodendroglia	-8.14	1.16	2.26E-12
Microglia	0.49	0.0448	7.63E-28
Neurons	0.427	0.0431	3.87E-23
Neurons	0.732	0.109	1.87E-11

Astrocytes	0.159	0.02	1.87E-15
Neurons	0.0312	0.004	6.19E-15
Endothelial cells	-1.46	0.158	2.45E-20
Astrocytes	-1.71	0.189	1.46E-19
Oligodendroglia	-4.7	0.542	4.26E-18
Microglia	2.72	0.334	3.83E-16
Neurons	0.737	0.101	2.94E-13
Microglia	-0.0331	0.00487	1.07E-11
Astrocytes	-0.4	0.0569	2.07E-12
Endothelial cells	-0.399	0.0569	2.34E-12
Neurons	-0.397	0.0568	2.76E-12
Microglia	-0.396	0.0568	3.13E-12
Oligodendroglia	-0.388	0.0574	1.38E-11
Endothelial cells	0.786	0.111	1.43E-12
Microglia	-0.274	0.039	2.13E-12
Neurons	-0.366	0.0276	3.90E-40
Microglia	0.278	0.0302	3.41E-20
Endothelial cells	0.267	0.0375	1.08E-12
Astrocytes	0.476	0.0564	3.18E-17
Endothelial cells	-2.71	0.349	8.16E-15
Neurons	-1.73	0.235	1.82E-13
Microglia	1.91	0.287	2.83E-11
Endothelial cells	-0.763	0.104	2.19E-13
Endothelial cells	-1.32	0.193	7.95E-12
Neurons	0.472	0.0593	1.73E-15
Microglia	0.516	0.0652	2.49E-15
Endothelial cells	0.323	0.0448	5.60E-13
Astrocytes	0.322	0.0449	7.42E-13
Oligodendroglia	0.322	0.045	8.33E-13
Neurons	0.321	0.045	9.80E-13
Microglia	0.313	0.0449	3.15E-12
Microglia	0.668	0.0877	2.60E-14
Microglia	-0.341	0.0474	6.29E-13
Neurons	-0.537	0.0607	9.01E-19
Microglia	-0.543	0.0616	1.20E-18
Endothelial cells	-0.802	0.101	2.01E-15
Oligodendroglia	-1.23	0.183	1.80E-11
Neurons	-0.606	0.0758	1.30E-15
Microglia	-0.58	0.0759	2.14E-14

Neurons	0.497	0.0564	1.23E-18
Microglia	0.393	0.0569	4.96E-12
Microglia	-0.44	0.0652	1.49E-11
Neurons	0.346	0.0438	2.80E-15
Endothelial cells	0.401	0.0543	1.53E-13
Oligodendroglia	2.43	0.21	5.75E-31
Endothelial cells	1.59	0.239	2.88E-11
Microglia	-0.153	0.0175	2.27E-18
Oligodendroglia	-7.28	1.04	2.56E-12
Oligodendroglia	3.61	0.497	3.77E-13
Microglia	0.811	0.111	2.75E-13
Neurons	0.693	0.102	1.09E-11
Oligodendroglia	3.71	0.536	4.46E-12
Oligodendroglia	1.35	0.143	3.71E-21
Astrocytes	-0.151	0.0211	8.28E-13
Neurons	0.374	0.0368	2.90E-24
Microglia	0.384	0.0419	4.97E-20
Oligodendroglia	1.71	0.175	1.49E-22
Endothelial cells	-3.54E-15	3.98E-16	5.87E-19
Endothelial cells	-7.1	0.659	4.57E-27
Oligodendroglia	-5.09	0.507	1.02E-23
Microglia	1.23	0.143	7.88E-18
Astrocytes	26.3	3.72	1.55E-12
Microglia	-0.336	0.0374	2.61E-19
Neurons	-0.272	0.036	4.17E-14
Endothelial cells	-0.43	0.0609	1.66E-12
Microglia	-4.43E-17	5.20E-18	1.61E-17
Oligodendroglia	1.97	0.255	1.11E-14
Neurons	0.604	0.0893	1.34E-11
Endothelial cells	1.2	0.171	2.26E-12
Astrocytes	1.24	0.18	5.62E-12
Microglia	1.26	0.188	2.05E-11
Neurons	3	0.257	1.75E-31
Endothelial cells	4.32	0.398	1.90E-27
Astrocytes	12.8	1.71	7.13E-14
Oligodendroglia	0.65	0.0813	1.29E-15
Oligodendroglia	0.353	0.0368	8.61E-22
Microglia	-0.0943	0.0102	2.35E-20
Microglia	0.343	0.0413	9.97E-17

Neurons	0.32	0.0429	8.70E-14
Endothelial cells	0.699	0.105	2.79E-11
Microglia	-0.303	0.0404	6.38E-14
Oligodendroglia	0.193	0.028	5.47E-12
Endothelial cells	0.193	0.0281	6.50E-12
Neurons	0.192	0.028	7.03E-12
Astrocytes	0.191	0.0281	1.07E-11
Microglia	-0.385	0.0458	4.24E-17
Endothelial cells	-0.234	0.0317	1.56E-13
Neurons	-0.233	0.0317	1.98E-13
Astrocytes	-0.232	0.0316	2.11E-13
Oligodendroglia	-0.232	0.0316	2.11E-13
Endothelial cells	0.62	0.0887	2.75E-12
Microglia	0.222	0.0287	1.03E-14
Neurons	0.208	0.0278	7.32E-14
Endothelial cells	1.2	0.153	4.39E-15
Neurons	0.461	0.0669	5.54E-12
Endothelial cells	1.22	0.171	9.71E-13
Neurons	0.468	0.0569	1.95E-16
Endothelial cells	0.61	0.0876	3.32E-12
Microglia	0.227	0.0273	9.17E-17
Endothelial cells	1.29	0.188	6.80E-12
Neurons	0.304	0.0349	3.02E-18
Endothelial cells	1.66	0.219	3.46E-14
Astrocytes	1.55	0.218	1.16E-12
Oligodendroglia	1.52	0.214	1.22E-12
Microglia	1.54	0.221	3.21E-12
Neurons	1.5	0.223	1.74E-11
Endothelial cells	0.691	0.0875	2.85E-15
Neurons	0.416	0.0534	6.69E-15
Oligodendroglia	-6.49	0.889	2.87E-13
Endothelial cells	1.01	0.104	2.69E-22
Neurons	-0.529	0.0591	3.52E-19
Neurons	1.26	0.152	1.14E-16
Endothelial cells	2.23	0.288	9.71E-15
Endothelial cells	-0.529	0.0291	7.61E-74
Neurons	-0.183	0.0159	1.18E-30
Microglia	0.212	0.0281	4.54E-14
Oligodendroglia	1.07	0.151	1.38E-12

Oligodendroglia	0.129	0.0113	3.48E-30
Microglia	-0.0361	0.00367	7.84E-23
Endothelial cells	-0.663	0.0972	9.04E-12
Endothelial cells	-0.859	0.0933	3.36E-20
Neurons	1.09	0.13	5.09E-17
Neurons	0.361	0.0488	1.39E-13
Neurons	3.98	0.482	1.49E-16
Astrocytes	-3.16	0.397	1.72E-15
Microglia	-3.15	0.401	3.99E-15
Oligodendroglia	-3.13	0.4	5.08E-15
Endothelial cells	-3.08	0.405	2.85E-14
Endothelial cells	-1.51	0.188	9.60E-16
Neurons	-0.668	0.0983	1.08E-11
Neurons	-0.581	0.0781	1.01E-13
Oligodendroglia	1.64	0.194	2.82E-17
Neurons	-0.362	0.0519	3.06E-12
Endothelial cells	1.41	0.163	5.14E-18
Neurons	0.652	0.0974	2.17E-11
Endothelial cells	1.09	0.14	6.93E-15
Oligodendroglia	0.0786	0.00815	5.20E-22
Microglia	-0.0242	0.00278	3.17E-18
Astrocytes	-0.126	0.0175	6.02E-13
Endothelial cells	0.77	0.109	1.62E-12
Neurons	-0.0404	0.00386	1.23E-25
Endothelial cells	0.17	0.0245	3.96E-12
Neurons	0.173	0.0197	1.61E-18
Endothelial cells	-0.251	0.0304	1.50E-16
Oligodendroglia	0.367	0.0405	1.28E-19
Endothelial cells	0.174	0.0193	1.96E-19
Microglia	0.0937	0.0139	1.57E-11
Endothelial cells	-0.866	0.109	1.94E-15
Neurons	-0.562	0.0786	8.67E-13
Neurons	-0.247	0.0293	3.46E-17
Oligodendroglia	-0.247	0.0295	5.62E-17
Astrocytes	-0.246	0.0294	5.89E-17
Endothelial cells	-0.245	0.0293	6.18E-17
Microglia	-0.245	0.0294	7.86E-17
Oligodendroglia	5.39	0.618	2.74E-18
Microglia	-2.14	0.28	2.12E-14

Oligodendroglia	5.28	0.539	1.17E-22
Oligodendroglia	-1.92	0.189	3.03E-24
Microglia	-0.344	0.0464	1.23E-13
Oligodendroglia	0.972	0.0866	3.11E-29
Microglia	-0.185	0.0259	9.14E-13
Oligodendroglia	1.94	0.183	2.95E-26
Microglia	-0.462	0.0579	1.47E-15
Oligodendroglia	0.12	0.0156	1.45E-14
Microglia	-0.552	0.0476	4.29E-31
Neurons	-0.451	0.05	1.88E-19
Microglia	-0.591	0.062	1.54E-21
Neurons	-0.482	0.0613	3.75E-15
Endothelial cells	0.75	0.094	1.48E-15
Oligodendroglia	-1.77	0.212	6.88E-17
Microglia	-1.77	0.213	9.58E-17
Endothelial cells	-1.76	0.212	1.02E-16
Astrocytes	-1.77	0.214	1.33E-16
Neurons	-1.76	0.213	1.42E-16
Endothelial cells	2.25	0.33	9.22E-12
Neurons	-0.372	0.0272	1.40E-42
Microglia	-0.35	0.0295	1.81E-32
Endothelial cells	-0.513	0.0487	6.03E-26
Neurons	-0.598	0.0451	3.98E-40
Microglia	-0.588	0.0471	9.12E-36
Endothelial cells	-0.605	0.0737	2.23E-16
Endothelial cells	0.599	0.071	3.27E-17
Endothelial cells	3.56	0.478	9.50E-14
Oligodendroglia	-0.429	0.0509	3.51E-17
Endothelial cells	2.04	0.275	1.19E-13
Endothelial cells	0.581	0.0464	5.69E-36
Oligodendroglia	0.948	0.094	6.43E-24
Microglia	0.258	0.0351	1.98E-13
Endothelial cells	0.098	0.0113	4.22E-18
Oligodendroglia	-0.152	0.018	3.05E-17
Neurons	0.0324	0.00403	9.01E-16
Microglia	0.0229	0.0032	8.29E-13
Neurons	-0.105	0.00896	1.02E-31
Microglia	0.0319	0.00467	8.44E-12
Microglia	0.0486	0.00409	1.46E-32

Microglia	0.0486	0.00409	1.46E-32
Astrocytes	89.1	2.60E-10	<1.0E-314
Endothelial cells	-2.93	2.70E-13	<1.0E-314
Microglia	-0.377	3.51E-15	<1.0E-314
Neurons	-0.739	1.51E-14	<1.0E-314
Oligodendroglia	-5.08	8.36E-13	<1.0E-314
Neurons	-0.833	0.0467	3.63E-71
Endothelial cells	-1.98	0.117	3.04E-64
Oligodendroglia	-4.07	0.248	1.59E-60
Microglia	-7.42	0.46	1.56E-58
Astrocytes	-23.5	1.48	8.95E-57
Neurons	0.0347	0.0051	1.02E-11
Astrocytes	-0.176	0.0166	2.90E-26
Microglia	-0.0297	0.00332	3.69E-19
Oligodendroglia	-1.27	0.0809	1.55E-55
Endothelial cells	-0.907	0.0627	2.00E-47
Astrocytes	4.24	0.301	4.61E-45
Neurons	-0.621	0.073	1.79E-17
Endothelial cells	1	0.101	4.12E-23
Neurons	0.51	0.0623	2.70E-16
Endothelial cells	1.17	0.127	3.18E-20
Endothelial cells	1.27	0.101	2.93E-36
Neurons	0.437	0.0544	9.50E-16
Microglia	0.26	0.0386	1.63E-11
Neurons	0.387	0.0551	2.16E-12
Endothelial cells	-2.5	0.376	2.95E-11
Endothelial cells	1.05	0.154	9.22E-12
Endothelial cells	-4.11	0.434	2.80E-21
Endothelial cells	2.64	0.314	4.18E-17
Microglia	-0.287	0.0429	2.23E-11
Endothelial cells	-3.05	0.248	9.24E-35
Neurons	-2.18	0.186	1.00E-31
Microglia	1.97	0.239	1.68E-16
Endothelial cells	-0.0491	0.00717	7.49E-12
Oligodendroglia	1.95	0.209	1.06E-20
Neurons	-1.96	0.257	2.41E-14
Astrocytes	-1.61	0.217	1.18E-13
Microglia	-1.6	0.22	3.52E-13
Endothelial cells	-1.59	0.225	1.59E-12

Microglia	-0.326	0.0372	1.89E-18
Neurons	-0.258	0.0372	4.05E-12
Endothelial cells	0.726	0.0745	1.94E-22
Neurons	0.291	0.0407	8.69E-13
Astrocytes	2.46	0.205	3.55E-33
Microglia	2.5	0.221	1.14E-29
Oligodendroglia	2.5	0.225	1.11E-28
Neurons	2.52	0.236	1.29E-26
Endothelial cells	2.28	0.225	3.93E-24
Endothelial cells	-5.75	0.523	4.07E-28
Oligodendroglia	-19.3	1.8	8.01E-27
Microglia	-0.447	0.0454	7.15E-23
Astrocytes	1.61	0.172	7.94E-21
Neurons	-0.781	0.0892	2.03E-18
Oligodendroglia	0.276	0.039	1.47E-12
Neurons	0.328	0.045	3.13E-13
Endothelial cells	-1.94	0.286	1.18E-11
Microglia	-0.341	0.0501	1.00E-11
Microglia	0.436	0.0544	1.10E-15
Microglia	-0.0267	0.00247	3.10E-27
Oligodendroglia	0.0961	0.0107	2.68E-19
Microglia	1.06	0.0818	2.10E-38
Endothelial cells	1.23	0.177	3.67E-12
Neurons	0.538	0.0793	1.17E-11
Neurons	0.51	0.0425	3.55E-33
Microglia	0.386	0.0399	3.88E-22
Neurons	0.793	0.0927	1.18E-17
Endothelial cells	1.25	0.154	4.78E-16
Endothelial cells	0.546	0.0544	1.05E-23
Neurons	0.218	0.031	2.03E-12
Oligodendroglia	0.399	0.0583	7.71E-12
Neurons	0.206	0.0308	2.26E-11
Astrocytes	11.2	1.54	3.52E-13
Oligodendroglia	8.67	1.23	1.80E-12
Endothelial cells	-0.764	0.11	3.77E-12
Microglia	-4.39	0.276	5.78E-57
Astrocytes	-4.39	0.277	1.44E-56
Oligodendroglia	-4.37	0.282	3.67E-54
Endothelial cells	-4.4	0.285	9.00E-54

Neurons	-4.28	0.282	5.00E-52
Endothelial cells	1.18	0.174	1.19E-11
Neurons	0.73	0.0966	4.13E-14
Microglia	-0.445	0.0651	8.16E-12
Microglia	-1.59	0.146	1.28E-27
Neurons	-1.65	0.169	1.62E-22
Endothelial cells	1.54	0.221	3.21E-12
Endothelial cells	-2.43	0.279	3.05E-18
Neurons	-1.16	0.166	2.79E-12
Oligodendroglia	0.0844	0.00683	4.45E-35
Microglia	-0.0245	0.00215	4.41E-30
Astrocytes	1.12	0.148	3.80E-14
Oligodendroglia	1.13	0.151	7.24E-14
Microglia	1.13	0.152	1.05E-13
Neurons	1.13	0.153	1.52E-13
Endothelial cells	1.07	0.152	1.93E-12
Endothelial cells	1.24	0.127	1.61E-22
Neurons	0.52	0.0691	5.26E-14
Microglia	-2.21	0.118	2.88E-78
Neurons	-1.99	0.116	5.75E-66
Endothelial cells	-2.4	0.199	1.71E-33
Neurons	0.262	0.0348	5.12E-14
Microglia	-0.195	0.0231	3.13E-17
Neurons	-0.167	0.0213	4.49E-15
Oligodendroglia	-1.53	0.129	1.90E-32
Microglia	-0.801	0.0738	1.92E-27
Neurons	-0.403	0.0405	2.51E-23
Astrocytes	-2.16	0.239	1.60E-19
Endothelial cells	-0.653	0.0803	4.22E-16
Neurons	0.132	0.0122	2.78E-27
Endothelial cells	-0.33	0.0315	1.11E-25
Endothelial cells	-1.91	0.22	3.89E-18
Neurons	0.675	0.0791	1.42E-17
Endothelial cells	-4.4	0.555	2.23E-15
Astrocytes	-4.96	0.367	1.28E-41
Oligodendroglia	-4.96	0.371	9.14E-41
Neurons	-4.99	0.374	1.31E-40
Microglia	-4.95	0.372	2.12E-40
Endothelial cells	-4.79	0.371	3.90E-38

Endothelial cells	-0.132	0.0131	7.03E-24
Neurons	-2.71	0.314	6.10E-18
Microglia	-2.65	0.308	7.71E-18
Oligodendroglia	-2.65	0.31	1.25E-17
Astrocytes	-2.63	0.311	2.75E-17
Endothelial cells	-2.44	0.307	1.90E-15
Endothelial cells	-0.985	0.123	1.16E-15
Neurons	-0.463	0.0693	2.37E-11
Astrocytes	-1.76	0.223	2.96E-15
Oligodendroglia	-1.75	0.227	1.27E-14
Microglia	-1.75	0.228	1.65E-14
Neurons	-2.19	0.289	3.51E-14
Endothelial cells	-3.98	0.542	2.09E-13
Oligodendroglia	-2.11	0.101	6.45E-97
Astrocytes	2.31	0.21	3.82E-28
Microglia	0.335	0.0348	6.18E-22
Neurons	-0.188	0.0275	8.12E-12
Neurons	-0.267	0.0305	2.06E-18
Endothelial cells	-0.436	0.0589	1.34E-13
Microglia	-0.216	0.032	1.48E-11
Oligodendroglia	0.927	0.115	7.58E-16
Neurons	-0.498	0.0683	3.07E-13
Microglia	-0.448	0.0653	6.86E-12
Endothelial cells	0.932	0.129	5.02E-13
Neurons	0.481	0.0721	2.54E-11
Endothelial cells	-2.02	0.287	1.95E-12
Endothelial cells	-0.832	0.118	1.78E-12
Endothelial cells	-0.55	0.0819	1.87E-11
Microglia	0.409	0.0478	1.16E-17
Neurons	0.363	0.0403	2.11E-19
Endothelial cells	0.822	0.106	8.85E-15
Neurons	0.826	0.0881	6.87E-21
Microglia	-0.322	0.0428	5.34E-14
Neurons	-0.273	0.0401	9.90E-12
Microglia	-0.134	0.0147	7.82E-20
Neurons	0.129	0.0148	2.88E-18
Microglia	-2.5	0.325	1.45E-14
Neurons	-2.27	0.308	1.70E-13
Endothelial cells	-1.05	0.0675	1.46E-54

Astrocytes	-2.53	0.231	6.47E-28
Microglia	0.458	0.0604	3.38E-14
Neurons	0.479	0.0643	9.37E-14
Astrocytes	0.224	0.029	1.13E-14
Endothelial cells	0.0626	0.00674	1.57E-20
Oligodendroglia	1.25	0.141	7.63E-19
Astrocytes	-6.21E-16	9.11E-17	9.32E-12
Endothelial cells	1.15	0.145	2.17E-15
Neurons	0.507	0.0685	1.35E-13
Microglia	0.15	0.0205	2.53E-13
Neurons	-4.54	0.231	5.38E-86
Endothelial cells	-6.39	0.343	1.84E-77
Microglia	-33.2	2.06	1.95E-58
Oligodendroglia	-3090	202	8.00E-53
Astrocytes	201	13.4	7.34E-51
Astrocytes	-0.259	0.0299	4.63E-18
Endothelial cells	-0.241	0.0301	1.18E-15
Oligodendroglia	-0.297	0.0432	6.20E-12
Endothelial cells	1.58	0.191	1.31E-16
Neurons	-0.578	0.043	3.44E-41
Microglia	-0.51	0.0465	5.46E-28
Endothelial cells	-0.693	0.0734	3.68E-21
Neurons	0.106	0.0115	3.04E-20
Microglia	-0.068	0.00979	3.76E-12
Microglia	0.111	0.0146	2.90E-14
Endothelial cells	1	0.137	2.89E-13
Microglia	-0.441	0.0563	4.76E-15
Microglia	-0.593	0.0454	5.45E-39
Neurons	-0.492	0.0442	8.84E-29
Neurons	-0.407	0.0593	6.72E-12
Neurons	0.541	0.0671	7.47E-16
Astrocytes	-0.205	0.0272	4.82E-14
Neurons	0.109	0.0139	4.44E-15
Endothelial cells	0.157	0.0219	7.56E-13
Endothelial cells	-1.19	0.168	1.41E-12
Microglia	-0.346	0.0506	8.03E-12
Oligodendroglia	0.243	0.0284	1.17E-17
Endothelial cells	-0.399	0.0554	5.93E-13
Neurons	-0.246	0.0346	1.16E-12

Endothelial cells	-2.82	0.393	7.20E-13
Microglia	0.053	0.00725	2.66E-13
Microglia	0.552	0.0811	1.00E-11
Neurons	-2.19	0.217	5.98E-24
Microglia	-2.2	0.233	3.66E-21
Microglia	-0.81	0.0966	5.07E-17
Endothelial cells	-1.28	0.164	5.96E-15
Endothelial cells	-1.2	0.167	6.69E-13
Neurons	0.399	0.0429	1.40E-20
Endothelial cells	2.88	0.194	7.46E-50
Oligodendroglia	2.72	0.185	6.19E-49
Astrocytes	2.75	0.191	5.33E-47
Microglia	2.74	0.197	5.61E-44
Neurons	2.76	0.199	9.72E-44
Endothelial cells	-2.1	0.158	2.61E-40
Neurons	0.248	0.0315	3.46E-15
Neurons	-0.345	0.0429	8.84E-16
Microglia	-0.308	0.044	2.56E-12
Neurons	-0.471	0.0661	1.04E-12
Astrocytes	1.08	0.0777	6.37E-44
Microglia	0.149	0.0145	9.05E-25
Astrocytes	-0.172	0.021	2.60E-16
Microglia	-0.0431	0.00537	1.01E-15
Microglia	1.19	0.0835	4.39E-46
Neurons	0.954	0.0961	3.17E-23
Microglia	-2.13	0.125	4.15E-65
Neurons	-2.21	0.153	2.72E-47
Endothelial cells	-2.25	0.243	2.06E-20
Neurons	-2.52	0.137	1.46E-75
Microglia	-1.9	0.137	9.82E-44
Endothelial cells	-2.41	0.22	6.32E-28
Neurons	0.185	0.00808	5.09E-116
Endothelial cells	0.186	0.0082	6.60E-114
Astrocytes	0.186	0.00822	2.31E-113
Microglia	0.186	0.00823	4.31E-113
Oligodendroglia	0.186	0.00823	4.31E-113
Astrocytes	0.458	0.0486	4.35E-21
Neurons	1.49	0.111	4.41E-41
Microglia	1.58	0.119	3.13E-40

Neurons	1.39	0.142	1.26E-22
Microglia	1.34	0.153	1.98E-18
Endothelial cells	0.963	0.135	9.80E-13
Neurons	0.136	0.0108	2.32E-36
Endothelial cells	0.282	0.0259	1.31E-27
Oligodendroglia	0.382	0.0409	9.65E-21
Astrocytes	-0.696	0.0995	2.65E-12
Endothelial cells	0.135	0.0192	2.05E-12
Endothelial cells	-6.46	0.548	4.48E-32
Neurons	-4.46	0.417	1.07E-26
Microglia	3.55	0.525	1.36E-11
Oligodendroglia	0.212	0.0268	2.56E-15
Microglia	2.42	0.349	4.09E-12
Astrocytes	-0.177	0.0213	9.58E-17
Endothelial cells	0.89	0.119	7.49E-14
Astrocytes	0.937	0.0963	2.25E-22
Oligodendroglia	0.938	0.0966	2.73E-22
Microglia	0.934	0.0963	3.05E-22
Endothelial cells	0.933	0.0965	4.11E-22
Neurons	0.935	0.0968	4.50E-22
Endothelial cells	-1.33	0.194	7.10E-12
Endothelial cells	0.845	0.127	2.86E-11
Microglia	-0.124	0.0153	5.29E-16
Astrocytes	-0.577	0.0721	1.22E-15
Neurons	-0.383	0.0503	2.65E-14
Oligodendroglia	0.671	0.0763	1.44E-18
Neurons	-1.32	0.184	7.29E-13
Oligodendroglia	2.16	0.228	2.70E-21
Oligodendroglia	-0.413	0.0396	1.82E-25
Astrocytes	2.61	0.268	2.06E-22
Microglia	-0.115	0.0153	5.63E-14
Astrocytes	5.11	0.494	4.45E-25
Oligodendroglia	1.73	0.184	5.34E-21
Endothelial cells	0.0725	0.00899	7.35E-16
Neurons	0.0405	0.00529	1.92E-14
Neurons	0.472	0.0709	2.79E-11
Oligodendroglia	0.567	0.0829	7.94E-12
Endothelial cells	1.32	0.155	1.65E-17
Endothelial cells	-0.159	0.0224	1.26E-12

Endothelial cells	-0.0541	0.00777	3.34E-12
Endothelial cells	0.568	0.0845	1.79E-11
Oligodendroglia	0.107	0.0132	5.23E-16
Oligodendroglia	2.73	0.195	1.56E-44
Endothelial cells	5.14	0.37	7.09E-44
Neurons	1.19	0.0924	5.93E-38
Endothelial cells	3.42	0.368	1.49E-20
Oligodendroglia	1.81	0.197	4.01E-20
Neurons	0.819	0.0925	8.44E-19
Neurons	12.6	1.75	6.02E-13
Endothelial cells	-4.4	0.658	2.28E-11
Oligodendroglia	-4.39	0.658	2.53E-11
Microglia	-0.894	0.0921	2.82E-22
Neurons	-1.07	0.156	6.94E-12
Microglia	-0.449	0.0614	2.62E-13
Neurons	0.436	0.0624	2.80E-12
Oligodendroglia	0.465	0.067	3.91E-12
Neurons	0.165	0.0191	5.68E-18
Endothelial cells	0.243	0.0292	8.66E-17
Oligodendroglia	1.11	0.138	8.73E-16
Endothelial cells	1.15	0.156	1.68E-13
Endothelial cells	0.198	0.0284	3.13E-12
Endothelial cells	1.24	0.181	7.34E-12
Endothelial cells	1.5	0.193	7.72E-15
Endothelial cells	-0.613	0.0888	5.09E-12
Endothelial cells	-0.937	0.103	9.28E-20
Neurons	-0.384	0.0506	3.23E-14
Endothelial cells	1.47	0.166	8.34E-19
Neurons	0.669	0.0884	3.79E-14
Microglia	1.34	0.197	1.03E-11
Astrocytes	1.26	0.189	2.62E-11
Microglia	0.821	0.0758	2.45E-27
Neurons	0.762	0.0846	2.12E-19
Astrocytes	-1.87	0.166	1.95E-29
Oligodendroglia	-1.86	0.166	3.86E-29
Microglia	-1.87	0.167	4.19E-29
Endothelial cells	-1.87	0.169	1.85E-28
Neurons	-1.81	0.167	2.27E-27
Neurons	-3.03	0.246	7.33E-35

Endothelial cells	-4.06	0.345	5.70E-32
Microglia	1.07	0.152	1.93E-12
Endothelial cells	-0.0801	0.00969	1.38E-16
Oligodendroglia	0.161	0.0217	1.18E-13
Astrocytes	0.311	0.0435	8.71E-13
Endothelial cells	1.04	0.152	7.80E-12
Neurons	-0.401	0.0594	1.47E-11
Endothelial cells	-3.33	0.448	1.06E-13
Endothelial cells	2.43	0.266	6.52E-20
Neurons	3.48	0.425	2.65E-16
Oligodendroglia	6.11	0.76	9.02E-16
Astrocytes	-23.6	3.04	8.28E-15
Microglia	0.504	0.0714	1.68E-12
Microglia	0.0713	0.00956	8.77E-14
Oligodendroglia	0.419	0.0567	1.47E-13
Microglia	-0.0901	0.0124	3.70E-13
Microglia	-0.214	0.032	2.27E-11
Oligodendroglia	-0.41	0.0616	2.82E-11
Endothelial cells	0.0872	0.0131	2.80E-11
Neurons	-0.0247	0.00315	4.46E-15
Astrocytes	-0.203	0.0294	5.03E-12
Oligodendroglia	0.126	0.0184	7.50E-12
Endothelial cells	0.075	0.00648	5.58E-31
Oligodendroglia	-0.127	0.0164	9.64E-15
Endothelial cells	-0.845	0.12	1.90E-12
Endothelial cells	0.33	0.0452	2.86E-13
Microglia	0.398	0.0494	7.84E-16
Endothelial cells	0.106	0.0159	2.62E-11
Endothelial cells	-1.35	0.177	2.40E-14
Neurons	-0.766	0.108	1.32E-12
Oligodendroglia	0.757	0.0676	4.16E-29
Microglia	-0.118	0.0116	2.63E-24
Neurons	0.0433	0.00529	2.72E-16
Microglia	0.0294	0.00263	5.18E-29
Astrocytes	-0.104	0.0123	2.78E-17
Neurons	-0.065	0.00912	1.02E-12
Oligodendroglia	0.149	0.0212	2.09E-12
Astrocytes	-0.171	0.0203	3.65E-17
Microglia	0.484	0.0474	1.77E-24

Neurons	0.464	0.0483	7.50E-22
Endothelial cells	0.612	0.0808	3.61E-14
Endothelial cells	0.263	0.0385	8.42E-12
Microglia	0.428	0.0295	1.07E-47
Endothelial cells	0.517	0.0464	7.81E-29
Neurons	0.279	0.0263	2.72E-26
Endothelial cells	0.658	0.0891	1.52E-13
Microglia	0.0847	0.0109	7.81E-15
Astrocytes	0.664	0.0968	6.91E-12
Neurons	0.612	0.0787	7.46E-15
Endothelial cells	0.922	0.134	5.96E-12
Oligodendroglia	1.17	0.168	3.30E-12
Endothelial cells	-2.7	0.318	2.06E-17
Oligodendroglia	-2.31	0.279	1.24E-16
Astrocytes	-2.39	0.317	4.72E-14
Microglia	-2.36	0.329	7.32E-13
Neurons	-2.4	0.339	1.45E-12
Oligodendroglia	-10.5	1.54	9.22E-12
Oligodendroglia	1.19	0.174	7.97E-12
Neurons	1.61	0.114	2.75E-45
Microglia	1.41	0.103	1.18E-42
Endothelial cells	1.87	0.183	1.64E-24
Endothelial cells	-0.867	0.111	5.68E-15
Endothelial cells	-2.87	0.272	5.00E-26
Neurons	-2.86	0.279	1.17E-24
Astrocytes	-2.8	0.277	5.08E-24
Microglia	-2.73	0.28	1.84E-22
Oligodendroglia	-2.48	0.255	2.35E-22
Endothelial cells	0.554	0.0813	9.47E-12
Neurons	0.374	0.033	8.97E-30
Microglia	0.308	0.0348	8.71E-19
Endothelial cells	0.821	0.122	1.70E-11
Endothelial cells	-1.15	0.17	1.34E-11
Endothelial cells	1.18	0.167	1.60E-12
Neurons	2.08	0.156	1.48E-40
Astrocytes	-1.61	0.121	2.14E-40
Oligodendroglia	-1.61	0.121	2.14E-40
Microglia	-1.6	0.121	6.45E-40
Endothelial cells	-1.58	0.122	2.32E-38

Endothelial cells	1.16	0.174	2.62E-11
Microglia	0.489	0.0428	3.13E-30
Neurons	0.49	0.0429	3.25E-30
Neurons	0.108	0.0162	2.62E-11
Endothelial cells	0.272	0.0408	2.62E-11
Endothelial cells	6.95	0.929	7.37E-14
Astrocytes	-3.77	0.381	4.38E-23
Microglia	-3.75	0.403	1.34E-20
Oligodendroglia	-3.75	0.403	1.34E-20
Endothelial cells	-3.63	0.405	3.16E-19
Neurons	-3.56	0.401	6.82E-19
Endothelial cells	-0.625	0.0904	4.72E-12
Neurons	-0.407	0.0607	2.01E-11
Endothelial cells	-0.177	0.00738	4.11E-127
Astrocytes	-0.176	0.00742	2.26E-124
Microglia	-0.176	0.00743	4.82E-124
Neurons	-0.176	0.00743	4.82E-124
Oligodendroglia	-0.176	0.00744	1.03E-123
Neurons	0.411	0.0444	2.11E-20
Microglia	0.37	0.0428	5.39E-18
Astrocytes	-0.375	0.0316	1.75E-32
Microglia	-0.0551	0.00627	1.52E-18
Endothelial cells	-0.12	0.0157	2.12E-14
Endothelial cells	1.64	0.19	6.05E-18
Neurons	-0.434	0.0416	1.76E-25
Microglia	-0.392	0.0474	1.34E-16
Endothelial cells	-0.552	0.0753	2.29E-13
Microglia	-0.238	0.0342	3.43E-12
Microglia	-0.186	0.0232	1.08E-15
Oligodendroglia	0.578	0.0481	2.91E-33
Microglia	-0.139	0.0163	1.49E-17
Microglia	-0.213	0.0315	1.36E-11
Endothelial cells	-0.865	0.125	4.52E-12
Microglia	-0.414	0.0587	1.75E-12
Endothelial cells	0.513	0.0687	8.19E-14
Endothelial cells	-0.964	0.142	1.13E-11
Endothelial cells	0.147	0.0207	1.23E-12
Astrocytes	0.204	0.0173	4.30E-32
Oligodendroglia	-0.0973	0.0129	4.61E-14

Endothelial cells	1.52	0.222	7.55E-12
Microglia	-4.45	0.317	9.14E-45
Oligodendroglia	-4.44	0.321	1.64E-43
Astrocytes	-4.43	0.323	8.24E-43
Neurons	-4.41	0.326	1.07E-41
Endothelial cells	-4.22	0.317	1.96E-40
Endothelial cells	0.621	0.0862	5.84E-13
Astrocytes	1.75	0.157	7.45E-29
Microglia	1.74	0.16	1.52E-27
Oligodendroglia	1.74	0.162	6.55E-27
Endothelial cells	1.69	0.162	1.77E-25
Neurons	1.7	0.163	1.82E-25
Microglia	-0.18	0.0242	1.02E-13
Astrocytes	-0.179	0.0241	1.11E-13
Oligodendroglia	-0.179	0.0242	1.40E-13
Neurons	-0.179	0.0244	2.20E-13
Endothelial cells	-0.178	0.0244	2.98E-13
Neurons	-0.498	0.0301	1.74E-61
Astrocytes	4.49	0.365	8.91E-35
Microglia	0.237	0.0301	3.44E-15
Endothelial cells	-0.526	0.0788	2.47E-11
Endothelial cells	-0.0437	0.00564	9.32E-15
Neurons	1.7	0.251	1.26E-11
Oligodendroglia	0.572	0.0791	4.78E-13
Oligodendroglia	3.55	0.528	1.77E-11
Endothelial cells	-0.818	0.106	1.19E-14
Neurons	-0.461	0.0618	8.68E-14
Oligodendroglia	-4.87	0.663	2.05E-13
Oligodendroglia	1.66	0.207	1.06E-15
Endothelial cells	0.497	0.0648	1.72E-14
Neurons	-2.39	0.288	1.05E-16
Endothelial cells	-2.07	0.256	6.17E-16
Astrocytes	-8.58	1.12	1.85E-14
Oligodendroglia	-59.5	8.08	1.79E-13
Microglia	1.07	0.091	6.41E-32
Neurons	0.704	0.0907	8.37E-15
Endothelial cells	1.14	0.167	8.71E-12
Oligodendroglia	2.25	0.327	5.95E-12
Endothelial cells	-0.866	0.124	2.87E-12

Neurons	-0.236	0.0341	4.49E-12
Neurons	0.877	0.115	2.42E-14
Endothelial cells	1.08	0.161	1.97E-11
Endothelial cells	0.771	0.114	1.35E-11
Neurons	0.412	0.0513	9.65E-16
Endothelial cells	1.39	0.141	6.32E-23
Oligodendroglia	0.45	0.0645	3.02E-12
Neurons	-0.362	0.0421	8.07E-18
Microglia	-0.354	0.0418	2.48E-17
Endothelial cells	1.03	0.128	8.49E-16
Neurons	0.444	0.0629	1.68E-12
Endothelial cells	-3.92	0.33	1.53E-32
Neurons	-1.16	0.146	1.94E-15
Astrocytes	-2.4	0.188	2.54E-37
Microglia	-2.4	0.189	6.03E-37
Oligodendroglia	-2.4	0.189	6.03E-37
Neurons	-2.37	0.19	1.04E-35
Endothelial cells	-2.35	0.19	3.87E-35
Microglia	0.782	0.107	2.70E-13
Neurons	0.799	0.11	3.77E-13
Neurons	1.25	0.182	6.50E-12
Neurons	-0.483	0.0689	2.38E-12
Neurons	-0.272	0.0286	1.90E-21
Neurons	0.308	0.0454	1.17E-11
Microglia	0.532	0.0624	1.52E-17
Microglia	0.546	0.08	8.79E-12
Microglia	-0.566	0.0445	4.63E-37
Neurons	-0.545	0.047	4.33E-31
Endothelial cells	-0.62	0.0785	2.83E-15
Microglia	-35	4.64	4.59E-14
Astrocytes	-5.14	0.717	7.57E-13
Oligodendroglia	3.77	0.541	3.20E-12
Endothelial cells	-0.838	0.126	2.92E-11
Endothelial cells	0.729	0.104	2.39E-12
Astrocytes	-2.66	0.313	1.92E-17
Endothelial cells	1.14	0.144	2.44E-15
Neurons	0.545	0.0805	1.29E-11
Endothelial cells	0.188	0.0146	6.09E-38
Neurons	0.0722	0.00846	1.41E-17

Microglia	0.187	0.0221	2.64E-17
Neurons	0.0322	0.00301	1.04E-26
Oligodendroglia	0.0862	0.0127	1.14E-11
Endothelial cells	-0.812	0.12	1.32E-11
Neurons	0.863	0.129	2.23E-11
Endothelial cells	3.6	0.524	6.41E-12
Astrocytes	-16.9	2.47	7.80E-12
Oligodendroglia	-14	2.08	1.69E-11
Endothelial cells	-0.787	0.118	2.57E-11
Neurons	-0.269	0.0268	1.04E-23
Microglia	-0.379	0.0322	5.56E-32
Oligodendroglia	-1.94	0.193	9.02E-24
Neurons	-0.102	0.0137	9.68E-14
Endothelial cells	-0.342	0.0484	1.59E-12
Neurons	-0.676	0.0955	1.46E-12
Microglia	-0.135	0.0157	8.06E-18
Microglia	-0.094	0.0108	3.21E-18
Astrocytes	-0.0936	0.0108	4.45E-18
Neurons	-0.0935	0.0108	4.83E-18
Oligodendroglia	-0.0935	0.0108	4.83E-18
Endothelial cells	-0.0922	0.0108	1.38E-17
Astrocytes	-0.343	0.0486	1.69E-12
Microglia	0.0231	0.00326	1.38E-12
Neurons	0.0262	0.00376	3.21E-12
Oligodendroglia	0.0748	0.0109	6.77E-12
Astrocytes	0.419	0.0604	4.00E-12
Astrocytes	0.166	0.0233	1.04E-12
Neurons	-0.0721	0.00943	2.08E-14
Neurons	-0.0717	0.00949	4.18E-14
Neurons	-0.28	0.0348	8.56E-16
Endothelial cells	-0.37	0.0553	2.22E-11
Microglia	-0.107	0.0139	1.38E-14
Neurons	-0.107	0.0139	1.38E-14
Astrocytes	-0.107	0.014	2.12E-14
Endothelial cells	-0.107	0.014	2.12E-14
Oligodendroglia	-0.106	0.0139	2.42E-14
Oligodendroglia	-0.146	0.0184	2.11E-15
Neurons	-0.106	0.0151	2.22E-12
Neurons	-0.198	0.0238	8.85E-17

Astrocytes	-0.523	0.0779	1.90E-11
Oligodendroglia	0.301	0.0384	4.56E-15
Microglia	-0.0705	0.0105	1.89E-11
Astrocytes	-0.0701	0.0105	2.45E-11
Neurons	-0.0699	0.0105	2.79E-11
Neurons	-0.185	0.0213	3.77E-18
Astrocytes	-0.184	0.0213	5.70E-18
Endothelial cells	-0.184	0.0213	5.70E-18
Microglia	-0.184	0.0213	5.70E-18
Oligodendroglia	-0.184	0.0213	5.70E-18
Endothelial cells	-0.133	0.0183	3.65E-13
Oligodendroglia	-0.133	0.0183	3.65E-13
Astrocytes	-0.133	0.0184	4.89E-13
Microglia	-0.133	0.0184	4.89E-13
Neurons	-0.132	0.0183	5.47E-13
Neurons	0.208	0.0182	3.01E-30
Astrocytes	0.209	0.0183	3.29E-30
Endothelial cells	0.209	0.0183	3.29E-30
Microglia	0.209	0.0183	3.29E-30
Oligodendroglia	0.209	0.0183	3.29E-30
Oligodendroglia	0.714	0.0895	1.49E-15
Neurons	-0.371	0.0529	2.33E-12
Astrocytes	-0.311	0.0446	3.10E-12
Neurons	0.104	0.0106	1.01E-22
Endothelial cells	-0.442	0.048	3.31E-20
Oligodendroglia	-1.16	0.137	2.51E-17
Astrocytes	-11.5	1.39	1.30E-16
Microglia	-0.0907	0.0112	5.58E-16
Neurons	-0.109	0.0119	5.21E-20
Oligodendroglia	-0.64	0.0739	4.70E-18
Astrocytes	0.127	0.0158	9.13E-16
Microglia	0.0282	0.00417	1.36E-11
Astrocytes	-0.0834	0.00977	1.39E-17
Neurons	-0.0833	0.00979	1.76E-17
Microglia	-0.083	0.00982	2.86E-17
Endothelial cells	-0.0825	0.00985	5.49E-17
Oligodendroglia	-0.0819	0.0099	1.31E-16
Microglia	-0.147	0.0188	5.32E-15
Neurons	-0.137	0.0177	9.93E-15

Endothelial cells	-0.0939	0.0141	2.75E-11
Endothelial cells	0.197	0.0227	4.01E-18
Astrocytes	-0.123	0.0163	4.49E-14
Microglia	-0.122	0.0163	7.17E-14
Oligodendroglia	-0.122	0.0164	1.01E-13
Neurons	-0.12	0.0162	1.29E-13
Endothelial cells	-0.0842	0.011	1.94E-14
Oligodendroglia	-0.084	0.011	2.23E-14
Astrocytes	-0.0835	0.011	3.18E-14
Microglia	-0.0835	0.011	3.18E-14
Neurons	-0.0834	0.011	3.41E-14
Neurons	0.0378	0.00408	1.96E-20
Oligodendroglia	-0.445	0.0275	6.78E-59
Microglia	-0.0943	0.0084	3.03E-29
Neurons	0.0224	0.00257	2.88E-18
Neurons	0.0852	0.00537	1.09E-56
Neurons	0.0195	0.00257	3.26E-14
Endothelial cells	-0.124	0.00908	1.85E-42
Neurons	-0.124	0.00912	4.20E-42
Oligodendroglia	-0.124	0.00912	4.20E-42
Microglia	-0.124	0.00913	5.15E-42
Astrocytes	-0.124	0.00915	7.72E-42
Neurons	0.0226	0.0032	1.64E-12
Endothelial cells	0.0824	0.00941	2.01E-18
Neurons	0.0442	0.00606	3.01E-13
Endothelial cells	5.38	0.47	2.44E-30
Neurons	-1.46	0.163	3.33E-19
Astrocytes	64.9	9.56	1.13E-11
Neurons	-0.332	0.038	2.40E-18
Endothelial cells	1.91	0.203	5.02E-21
Neurons	0.356	0.0525	1.19E-11
Endothelial cells	0.829	0.0785	4.54E-26
Microglia	0.529	0.0508	2.15E-25
Astrocytes	1.74	0.232	6.38E-14
Oligodendroglia	1.02	0.143	9.83E-13
Endothelial cells	-3.3	0.446	1.37E-13
Endothelial cells	-0.67	0.0996	1.73E-11
Oligodendroglia	2.23	0.242	3.12E-20
Microglia	-0.604	0.0849	1.13E-12

Neurons	2.71	0.394	6.06E-12
Endothelial cells	2.15	0.32	1.83E-11
Endothelial cells	0.882	0.127	3.79E-12
Endothelial cells	1.07	0.153	2.68E-12
Endothelial cells	-0.67	0.0967	4.25E-12
Oligodendroglia	2.28	0.231	5.61E-23
Microglia	-0.573	0.0819	2.63E-12
Endothelial cells	-0.818	0.116	1.77E-12
Neurons	0.586	0.0704	8.51E-17
Endothelial cells	0.763	0.114	2.19E-11
Endothelial cells	-0.964	0.142	1.13E-11
Oligodendroglia	-2.43	0.254	1.10E-21
Astrocytes	15.6	1.7	4.45E-20
Microglia	-0.659	0.0955	5.18E-12
Astrocytes	-1.2	0.175	7.03E-12
Oligodendroglia	-1.24	0.182	9.55E-12
Oligodendroglia	0.229	0.0328	2.92E-12
Astrocytes	0.915	0.0637	8.68E-47
Endothelial cells	0.877	0.115	2.42E-14
Neurons	0.416	0.061	9.12E-12
Neurons	0.602	0.0594	3.88E-24
Microglia	0.466	0.0617	4.26E-14
Neurons	1.06	0.146	3.86E-13
Endothelial cells	-0.272	0.0374	3.52E-13
Microglia	-0.172	0.0244	1.80E-12
Endothelial cells	-0.0995	0.0134	1.12E-13
Oligodendroglia	-0.0991	0.0134	1.41E-13
Neurons	-0.0983	0.0133	1.46E-13
Microglia	-0.099	0.0134	1.49E-13
Astrocytes	-0.0989	0.0134	1.58E-13
Endothelial cells	-0.566	0.0723	4.94E-15
Microglia	0.338	0.0445	3.07E-14
Neurons	-0.319	0.0454	2.12E-12
Oligodendroglia	0.896	0.132	1.14E-11
Endothelial cells	-1.48	0.214	4.65E-12
Neurons	0.402	0.0604	2.82E-11
Neurons	-0.0948	0.00701	1.14E-41
Neurons	-0.112	0.00795	4.50E-45
Microglia	-0.107	0.0113	2.82E-21

Endothelial cells	-0.164	0.0232	1.56E-12
Oligodendroglia	-0.22	0.0312	1.77E-12
Microglia	0.203	0.0267	2.89E-14
Microglia	0.322	0.0458	2.06E-12
Endothelial cells	0.0706	0.0101	2.75E-12
Neurons	-0.0298	0.00286	2.02E-25
Endothelial cells	1.59	0.227	2.48E-12
Endothelial cells	1.89	0.277	8.91E-12
Astrocytes	2.22	0.16	8.98E-44
Oligodendroglia	-3.51	0.272	4.25E-38
Neurons	-4.02	0.313	9.36E-38
Endothelial cells	0.569	0.0472	1.82E-33
Microglia	2.45	0.215	4.41E-30
Endothelial cells	-0.538	0.0511	6.39E-26
Oligodendroglia	-0.526	0.0503	1.36E-25
Microglia	-0.529	0.0507	1.74E-25
Astrocytes	-0.528	0.0507	2.14E-25
Neurons	-0.516	0.051	4.61E-24
Astrocytes	-2	0.254	3.43E-15
Endothelial cells	-0.565	0.0793	1.04E-12
Endothelial cells	1.66	0.178	1.10E-20
Neurons	-0.598	0.0898	2.75E-11
Neurons	0.902	0.114	2.53E-15
Endothelial cells	0.98	0.138	1.23E-12
Oligodendroglia	3.7	0.359	6.59E-25
Endothelial cells	-0.839	0.104	7.19E-16
Endothelial cells	1.23	0.125	7.57E-23
Neurons	0.685	0.0735	1.17E-20
Endothelial cells	0.779	0.105	1.18E-13
Endothelial cells	0.901	0.109	1.38E-16
Neurons	0.746	0.0778	8.92E-22
Endothelial cells	1.04	0.111	7.30E-21
Endothelial cells	-0.647	0.0892	4.07E-13
Neurons	-0.444	0.0658	1.50E-11
Neurons	2.76	0.291	2.43E-21
Endothelial cells	2.73	0.293	1.19E-20
Oligodendroglia	2.6	0.291	4.08E-19
Astrocytes	2.61	0.293	5.20E-19
Microglia	2.6	0.292	5.38E-19

Endothelial cells	0.848	0.127	2.44E-11
Astrocytes	1.53	0.159	6.42E-22
Endothelial cells	1.6	0.177	1.57E-19
Oligodendroglia	1.5	0.173	4.30E-18
Microglia	1.51	0.177	1.45E-17
Neurons	1.47	0.179	2.17E-16
Endothelial cells	-0.855	0.119	6.73E-13
Oligodendroglia	-0.0389	0.00569	8.11E-12
Astrocytes	1.41	0.161	1.99E-18
Neurons	1.41	0.161	1.99E-18
Microglia	1.39	0.16	3.71E-18
Endothelial cells	1.37	0.158	4.29E-18
Oligodendroglia	1.34	0.161	8.58E-17
Endothelial cells	0.432	0.0642	1.71E-11
Endothelial cells	1.12	0.138	4.82E-16
Endothelial cells	0.165	0.0166	2.80E-23
Oligodendroglia	3.04	0.397	1.90E-14
Microglia	-0.577	0.0668	5.73E-18
Neurons	-0.485	0.0579	5.45E-17
Endothelial cells	-0.734	0.109	1.65E-11
Microglia	-0.225	0.031	3.93E-13
Microglia	0.288	0.0429	1.90E-11
Microglia	-0.519	0.0479	2.35E-27
Endothelial cells	-0.524	0.0776	1.45E-11
Endothelial cells	1	0.145	5.33E-12
Neurons	0.078	0.00522	1.74E-50
Endothelial cells	0.112	0.00774	1.87E-47
Endothelial cells	0.887	0.127	2.86E-12
Neurons	-2.36	0.178	4.03E-40
Oligodendroglia	4.35	0.526	1.34E-16
Endothelial cells	1.34	0.187	7.73E-13
Endothelial cells	0.204	0.0261	5.45E-15
Astrocytes	-1.66	0.164	4.42E-24
Microglia	-1.65	0.165	1.52E-23
Oligodendroglia	-1.65	0.165	1.52E-23
Neurons	-1.64	0.167	9.20E-23
Endothelial cells	-1.62	0.167	3.00E-22
Endothelial cells	-0.934	0.135	4.56E-12
Microglia	0.681	0.072	3.13E-21

Endothelial cells	0.856	0.123	3.42E-12
Astrocytes	0.214	0.0176	5.13E-34
Microglia	0.0358	0.00375	1.34E-21
Neurons	-0.365	0.0418	2.50E-18
Neurons	0.819	0.112	2.62E-13
Endothelial cells	1.26	0.173	3.26E-13
Oligodendroglia	-4.38	0.654	2.12E-11
Neurons	-2.28	0.263	4.35E-18
Oligodendroglia	0.243	0.0241	6.57E-24
Endothelial cells	0.0643	0.00789	3.65E-16
Astrocytes	0.354	0.0478	1.30E-13
Astrocytes	-0.15	0.0209	7.12E-13
Astrocytes	-0.211	0.0284	1.09E-13
Microglia	-0.286	0.0413	4.36E-12
Astrocytes	0.166	0.0206	7.74E-16
Endothelial cells	-0.0506	0.00756	2.18E-11
Neurons	0.0529	0.00514	7.67E-25
Microglia	-0.533	0.0687	8.60E-15
Astrocytes	-8.58	1.22	2.02E-12
Astrocytes	-0.285	0.036	2.44E-15
Endothelial cells	0.804	0.105	1.90E-14
Endothelial cells	0.74	0.108	7.29E-12
Neurons	0.372	0.0544	8.02E-12
Endothelial cells	3.7	0.384	5.67E-22
Neurons	1.49	0.198	5.26E-14
Endothelial cells	2.56	0.321	1.52E-15
Neurons	-0.798	0.0909	1.65E-18
Endothelial cells	-1.21	0.167	4.31E-13
Endothelial cells	-3.99	0.501	1.66E-15
Endothelial cells	-1.48	0.165	2.97E-19
Neurons	-0.726	0.0939	1.06E-14
Neurons	2.68	0.265	4.83E-24
Endothelial cells	-1.2	0.136	1.11E-18
Neurons	-2.3	0.114	1.61E-90
Microglia	-2.37	0.137	4.76E-67
Endothelial cells	-1.45	0.201	5.44E-13
Endothelial cells	2.06	0.297	4.03E-12
Neurons	-0.719	0.108	2.79E-11
Microglia	-0.598	0.0824	3.95E-13

Oligodendroglia	-1.91	0.198	5.09E-22
Neurons	-1.93	0.201	7.84E-22
Astrocytes	-1.91	0.199	8.15E-22
Microglia	-1.91	0.199	8.15E-22
Endothelial cells	-1.87	0.201	1.36E-20
Endothelial cells	-0.685	0.0889	1.31E-14
Endothelial cells	1.51	0.206	2.30E-13
Microglia	-0.408	0.0479	1.63E-17
Neurons	-0.436	0.0537	4.69E-16
Oligodendroglia	5.66	0.461	1.19E-34
Microglia	-1.26	0.164	1.55E-14
Oligodendroglia	4.3	0.335	1.03E-37
Microglia	-1.18	0.11	7.58E-27
Astrocytes	-4.14	0.598	4.42E-12
Oligodendroglia	2.48	0.214	4.70E-31
Microglia	-0.689	0.0725	2.03E-21
Endothelial cells	0.978	0.124	3.09E-15
Neurons	0.618	0.0877	1.83E-12
Oligodendroglia	1.32	0.177	8.81E-14
Microglia	0.573	0.0802	9.02E-13
Endothelial cells	-2.33	0.3	8.06E-15
Neurons	0.493	0.0721	8.05E-12
Endothelial cells	0.978	0.122	1.09E-15
Neurons	-0.655	0.0966	1.20E-11
Neurons	0.544	0.0562	3.68E-22
Microglia	0.519	0.0607	1.23E-17
Endothelial cells	-1.23	0.151	3.77E-16
Neurons	-0.639	0.0861	1.16E-13
Neurons	-0.568	0.0761	8.40E-14
Endothelial cells	-0.916	0.136	1.64E-11
Endothelial cells	2.39	0.343	3.22E-12
Neurons	1.72	0.249	4.93E-12
Endothelial cells	-1.1	0.135	3.70E-16
Neurons	-0.58	0.0808	7.06E-13
Microglia	-0.459	0.0433	2.97E-26
Neurons	-0.422	0.0429	7.81E-23
Neurons	0.392	0.0538	3.19E-13
Neurons	0.473	0.0493	8.45E-22
Microglia	0.377	0.0566	2.72E-11

Endothelial cells	-0.0898	0.00731	1.10E-34
Oligodendroglia	0.127	0.0154	1.63E-16
Microglia	-0.0329	0.00473	3.51E-12
Oligodendroglia	13.6	1.27	9.27E-27
Microglia	2.77	0.269	7.24E-25
Astrocytes	13.5	1.45	1.27E-20
Endothelial cells	4.16	0.579	6.73E-13
Neurons	-2.33	0.334	3.04E-12
Oligodendroglia	-0.514	0.0506	3.05E-24
Oligodendroglia	-30	4.31	3.39E-12
Astrocytes	-32.7	4.71	3.85E-12
Endothelial cells	4.98	0.738	1.50E-11
Neurons	4.61	0.688	2.08E-11
Neurons	1.05	0.145	4.44E-13
Endothelial cells	0.735	0.108	1.01E-11
Endothelial cells	-0.816	0.109	7.09E-14
Oligodendroglia	-0.872	0.101	5.94E-18
Endothelial cells	0.382	0.0519	1.83E-13
Neurons	0.206	0.0295	2.89E-12
Neurons	0.418	0.0617	1.25E-11
Microglia	-0.631	0.0789	1.27E-15
Microglia	-2.32	0.212	7.15E-28
Neurons	3.06	0.315	2.62E-22
Astrocytes	-2.28	0.235	2.95E-22
Oligodendroglia	-2.29	0.239	9.56E-22
Endothelial cells	-2.19	0.239	5.04E-20
Endothelial cells	1.03	0.142	4.06E-13
Endothelial cells	-1.17	0.156	6.38E-14
Neurons	-0.841	0.116	4.17E-13
Endothelial cells	-4.02	0.425	3.11E-21
Astrocytes	23.5	2.7	3.21E-18
Oligodendroglia	5.62	0.65	5.33E-18
Neurons	-0.657	0.0917	7.80E-13
Endothelial cells	-0.895	0.129	3.98E-12
Neurons	-2.23	0.253	1.21E-18
Oligodendroglia	-2.18	0.25	2.78E-18
Endothelial cells	-2.21	0.254	3.30E-18
Microglia	-2.16	0.252	1.02E-17
Astrocytes	-2.14	0.252	2.03E-17

Microglia	0.251	0.0271	2.01E-20
Astrocytes	-0.232	0.0176	1.12E-39
Endothelial cells	0.151	0.014	4.02E-27
Oligodendroglia	-0.174	0.019	5.29E-20
Neurons	0.0832	0.00909	5.54E-20
Neurons	-4.42	0.617	7.85E-13
Neurons	0.0302	0.00401	5.03E-14
Neurons	0.0032	0.000429	8.70E-14
Endothelial cells	-0.506	0.0561	1.89E-19
Endothelial cells	0.921	0.1	3.26E-20
Neurons	0.484	0.0675	7.48E-13
Microglia	0.257	0.0381	1.53E-11
Endothelial cells	1.62	0.234	4.42E-12
Neurons	-3.54	0.389	9.01E-20
Endothelial cells	-5.96	0.694	8.86E-18
Microglia	5.62	0.786	8.67E-13
Endothelial cells	0.0907	0.0126	6.09E-13
Neurons	0.513	0.0541	2.48E-21
Microglia	0.425	0.0504	3.38E-17
Endothelial cells	0.931	0.132	1.75E-12
Neurons	0.376	0.0549	7.45E-12
Neurons	-0.722	0.101	8.77E-13
Endothelial cells	0.616	0.0888	4.01E-12
Neurons	-0.474	0.0687	5.22E-12
Microglia	0.274	0.0275	2.20E-23
Neurons	0.248	0.0271	5.63E-20
Endothelial cells	-0.729	0.105	3.84E-12
Neurons	-0.499	0.0553	1.82E-19
Microglia	-0.488	0.0602	5.22E-16
Endothelial cells	-1.29	0.178	4.26E-13
Endothelial cells	-2.08	0.275	3.92E-14
Microglia	-0.32	0.0466	6.56E-12
Endothelial cells	0.981	0.138	1.17E-12
Endothelial cells	0.863	0.124	3.41E-12
Endothelial cells	0.23	0.0289	1.74E-15
Astrocytes	0.23	0.032	6.60E-13
Oligodendroglia	0.453	0.0666	1.03E-11
Oligodendroglia	-0.2	0.0239	5.85E-17
Endothelial cells	-0.069	0.00969	1.07E-12

Neurons	-0.069	0.0097	1.13E-12
Astrocytes	-0.0688	0.00969	1.25E-12
Microglia	-0.0687	0.0097	1.42E-12
Endothelial cells	0.864	0.108	1.24E-15
Endothelial cells	0.734	0.0922	1.71E-15
Neurons	0.337	0.0503	2.09E-11
Oligodendroglia	-1.68	0.198	2.16E-17
Microglia	-1.65	0.222	1.07E-13
Astrocytes	-1.65	0.226	2.86E-13
Neurons	2.08	0.293	1.26E-12
Endothelial cells	4.49	0.67	2.06E-11
Neurons	1.34	0.161	8.58E-17
Neurons	0.386	0.0579	2.62E-11
Neurons	-0.458	0.0601	2.52E-14
Endothelial cells	0.252	0.0314	1.01E-15
Neurons	0.125	0.0179	2.88E-12
Astrocytes	0.325	0.0479	1.16E-11
Neurons	0.0277	0.00349	2.07E-15
Microglia	1.02	0.119	1.02E-17
Neurons	0.921	0.117	3.50E-15
Neurons	-0.63	0.0783	8.56E-16
Neurons	-0.347	0.0505	6.36E-12
Neurons	-0.441	0.0476	1.96E-20
Endothelial cells	0.727	0.096	3.65E-14
Neurons	0.0383	0.00402	1.61E-21
Neurons	-2.11	0.185	3.93E-30
Endothelial cells	-3.06	0.278	3.53E-28
Microglia	-14.9	1.66	2.81E-19
Oligodendroglia	-1390	163	1.49E-17
Astrocytes	89.9	10.7	4.39E-17
Endothelial cells	1.18	0.141	5.82E-17
Neurons	0.602	0.0803	6.54E-14
Oligodendroglia	0.512	0.0715	8.02E-13
Neurons	1.73	0.219	2.80E-15
Endothelial cells	1.68	0.221	2.92E-14
Astrocytes	1.63	0.218	7.60E-14
Oligodendroglia	1.62	0.217	8.30E-14
Microglia	1.63	0.22	1.27E-13
Endothelial cells	-0.882	0.13	1.16E-11

Endothelial cells	0.874	0.0932	6.74E-21
Neurons	0.412	0.0565	3.05E-13
Neurons	-0.15	0.012	7.47E-36
Astrocytes	-0.747	0.0707	4.29E-26
Microglia	-0.15	0.0173	4.30E-18
Endothelial cells	0.127	0.0168	4.05E-14
Neurons	-0.72	0.0828	3.45E-18
Endothelial cells	-1.1	0.143	1.45E-14
Endothelial cells	0.142	0.0186	2.27E-14
Microglia	-0.285	0.0384	1.15E-13
Endothelial cells	-0.44	0.0638	5.33E-12
Oligodendroglia	-0.101	0.00823	1.28E-34
Microglia	0.0271	0.00274	4.58E-23
Endothelial cells	0.162	0.0219	1.39E-13
Neurons	0.71	0.0895	2.14E-15
Oligodendroglia	-1.4	0.0916	9.80E-53
Microglia	-1.41	0.0925	1.83E-52
Astrocytes	-1.4	0.092	2.71E-52
Endothelial cells	-1.4	0.092	2.71E-52
Neurons	-1.4	0.0923	5.77E-52
Oligodendroglia	-1.33	0.131	3.22E-24
Oligodendroglia	4.34	0.572	3.26E-14
Oligodendroglia	3.68	0.346	2.03E-26
Oligodendroglia	0.174	0.0249	2.79E-12
Oligodendroglia	1.48	0.205	5.22E-13
Oligodendroglia	1.01	0.125	6.48E-16
Microglia	0.0482	0.00705	8.09E-12
Neurons	-2.76	0.388	1.13E-12
Microglia	-2.45	0.358	7.72E-12
Endothelial cells	-1.45	0.176	1.74E-16
Neurons	-0.66	0.0953	4.34E-12
Neurons	-0.468	0.0653	7.67E-13
Endothelial cells	-0.673	0.0944	1.01E-12
Endothelial cells	1.08	0.16	1.48E-11
Microglia	-0.0685	0.00993	5.26E-12
Oligodendroglia	0.237	0.0323	2.18E-13
Astrocytes	5.84	0.214	5.62E-164
Microglia	0.205	0.0132	2.16E-54
Endothelial cells	0.299	0.0232	5.26E-38

Oligodendroglia	-0.622	0.0718	4.60E-18
Neurons	0.0298	0.00377	2.69E-15
Endothelial cells	0.707	0.0954	1.25E-13
Neurons	0.372	0.0528	1.85E-12
Astrocytes	0.371	0.0497	8.34E-14
Microglia	0.0833	0.0113	1.68E-13
Endothelial cells	1.03	0.155	3.03E-11
Microglia	-0.175	0.0229	2.14E-14
Neurons	-0.494	0.0727	1.08E-11
Microglia	0.624	0.0838	9.60E-14
Neurons	-0.409	0.0597	7.34E-12
Endothelial cells	0.629	0.0857	2.14E-13
Endothelial cells	0.93	0.138	1.59E-11
Endothelial cells	1.6	0.222	5.71E-13
Neurons	0.696	0.0998	3.08E-12
Oligodendroglia	4.48	0.377	1.45E-32
Microglia	-0.986	0.136	4.17E-13
Endothelial cells	-0.106	0.015	1.59E-12
Microglia	-0.105	0.015	2.56E-12
Oligodendroglia	-0.105	0.015	2.56E-12
Astrocytes	-0.104	0.015	4.11E-12
Neurons	-0.104	0.015	4.11E-12
Endothelial cells	1.61	0.187	7.33E-18
Neurons	0.547	0.0784	3.01E-12
Oligodendroglia	0.454	0.0456	2.37E-23
Neurons	-0.152	0.0161	3.69E-21
Astrocytes	-0.0958	0.0134	8.73E-13
Endothelial cells	-0.0965	0.0135	8.80E-13
Oligodendroglia	-0.0961	0.0135	1.09E-12
Microglia	-0.0955	0.0135	1.50E-12
Neurons	-0.0947	0.0135	2.30E-12
Microglia	0.366	0.0414	9.52E-19
Neurons	0.302	0.0411	2.01E-13
Neurons	0.0333	0.0047	1.39E-12
Microglia	-1.45	0.165	1.52E-18
Oligodendroglia	-1.45	0.172	3.45E-17
Astrocytes	-1.43	0.173	1.39E-16
Neurons	-1.42	0.182	6.08E-15
Endothelial cells	-1.19	0.16	1.03E-13

Oligodendroglia	-1.4	0.137	1.63E-24
Endothelial cells	-1.15	0.135	1.62E-17
Microglia	-0.589	0.0779	4.00E-14
Neurons	-0.56	0.079	1.35E-12
Endothelial cells	1.38	0.182	3.39E-14
Neurons	0.751	0.106	1.39E-12
Neurons	-0.419	0.0495	2.57E-17
Microglia	0.361	0.0428	3.32E-17
Endothelial cells	-3.02	0.44	6.71E-12
Microglia	-0.222	0.0322	5.41E-12
Neurons	-0.696	0.0701	3.12E-23
Endothelial cells	-0.99	0.108	4.88E-20
Neurons	-0.519	0.066	3.73E-15
Oligodendroglia	0.0848	0.00765	1.48E-28
Microglia	-0.0317	0.00367	5.74E-18
Microglia	0.513	0.0587	2.34E-18
Neurons	0.449	0.0555	5.96E-16
Endothelial cells	0.997	0.119	5.38E-17
Astrocytes	-0.185	0.0114	3.19E-59
Neurons	-0.185	0.0114	3.19E-59
Oligodendroglia	-0.185	0.0114	3.19E-59
Endothelial cells	-0.184	0.0114	1.33E-58
Microglia	-0.184	0.0114	1.33E-58
Microglia	-0.413	0.059	2.56E-12
Neurons	-1.82	0.0814	9.93E-111
Microglia	-1.57	0.0839	3.90E-78
Endothelial cells	-1.36	0.136	1.52E-23
Neurons	-0.596	0.0764	6.14E-15
Astrocytes	0.465	0.0644	5.18E-13
Microglia	-0.081	0.0113	7.60E-13
Endothelial cells	-0.0801	0.0112	8.57E-13
Neurons	-0.0803	0.0113	1.19E-12
Oligodendroglia	-0.0779	0.0113	5.43E-12
Endothelial cells	0.685	0.0989	4.32E-12
Microglia	2.12	0.128	1.30E-61
Oligodendroglia	2.11	0.129	3.90E-60
Astrocytes	2.12	0.13	8.70E-60
Endothelial cells	2.1	0.131	7.82E-58
Neurons	2.1	0.131	7.82E-58

Endothelial cells	-0.69	0.0887	7.31E-15
Microglia	0.746	0.0531	7.82E-45
Neurons	0.783	0.0572	1.18E-42
Endothelial cells	0.656	0.0909	5.33E-13
Microglia	-0.683	0.099	5.24E-12
Endothelial cells	0.726	0.107	1.16E-11
Neurons	0.509	0.0761	2.25E-11
Neurons	1.44	0.209	5.58E-12
Astrocytes	1.39	0.207	1.88E-11
Microglia	1.37	0.205	2.34E-11
Oligodendroglia	1.39	0.208	2.35E-11
Endothelial cells	-0.892	0.113	2.93E-15
Endothelial cells	1.26	0.178	1.46E-12
Neurons	0.117	0.012	1.84E-22
Endothelial cells	1.16	0.143	4.98E-16
Endothelial cells	-0.662	0.0926	8.74E-13
Endothelial cells	1.31	0.147	5.03E-19
Neurons	0.645	0.0823	4.61E-15
Neurons	-1.96	0.148	4.94E-40
Astrocytes	-1.91	0.147	1.34E-38
Microglia	-1.91	0.147	1.34E-38
Oligodendroglia	-1.91	0.147	1.34E-38
Endothelial cells	-1.9	0.149	3.05E-37
Microglia	-2	0.168	1.12E-32
Neurons	2.63	0.221	1.18E-32
Astrocytes	-2	0.169	2.59E-32
Oligodendroglia	-1.99	0.169	5.24E-32
Endothelial cells	-1.99	0.171	2.66E-31
Endothelial cells	0.72	0.107	1.71E-11
Endothelial cells	0.96	0.13	1.53E-13
Neurons	2.8	0.252	1.11E-28
Astrocytes	-2.14	0.203	5.54E-26
Microglia	-2.14	0.203	5.54E-26
Oligodendroglia	-2.14	0.203	5.54E-26
Endothelial cells	-2.13	0.205	2.75E-25
Endothelial cells	0.872	0.105	1.00E-16
Neurons	0.527	0.0766	5.99E-12
Endothelial cells	0.713	0.0958	9.87E-14
Neurons	0.444	0.0616	5.69E-13

Endothelial cells	0.916	0.132	3.94E-12
Endothelial cells	0.368	0.042	1.92E-18
Neurons	-0.358	0.0505	1.35E-12
Endothelial cells	5.9	0.586	7.63E-24
Neurons	2.14	0.249	8.37E-18
Astrocytes	79	11.5	6.44E-12
Endothelial cells	0.898	0.121	1.16E-13
Endothelial cells	1.14	0.13	1.80E-18
Neurons	0.525	0.0676	8.08E-15
Neurons	0.648	0.0964	1.79E-11
Endothelial cells	3.25	0.419	8.73E-15
Neurons	-2.23	0.32	3.20E-12
Microglia	2.28	0.341	2.29E-11
Neurons	-1.33	0.152	2.13E-18
Endothelial cells	1.88	0.216	3.21E-18
Endothelial cells	1.58	0.224	1.74E-12
Microglia	-0.47	0.0613	1.76E-14
Endothelial cells	1.44	0.207	3.49E-12
Endothelial cells	-5.38	0.526	1.48E-24
Astrocytes	17.6	2.43	4.40E-13
Neurons	-1.35	0.193	2.66E-12
Endothelial cells	1.13	0.142	1.75E-15
Astrocytes	1.46	0.186	4.18E-15
Oligodendroglia	1.46	0.186	4.18E-15
Microglia	1.45	0.185	4.58E-15
Neurons	1.45	0.188	1.23E-14
Endothelial cells	1.44	0.188	1.87E-14
Endothelial cells	-2.14	0.254	3.60E-17
Endothelial cells	0.988	0.137	5.53E-13
Neurons	0.645	0.092	2.37E-12
Endothelial cells	1	0.118	2.36E-17
Neurons	0.419	0.0601	3.13E-12
Endothelial cells	0.828	0.122	1.15E-11
Neurons	0.572	0.086	2.91E-11
Endothelial cells	1.74	0.192	1.27E-19
Neurons	0.926	0.108	9.99E-18
Oligodendroglia	-5.17	0.752	6.20E-12
Microglia	0.261	0.039	2.20E-11
Neurons	0.545	0.061	4.09E-19

Endothelial cells	0.904	0.104	3.55E-18
Endothelial cells	-2.02	0.217	1.29E-20
Neurons	0.403	0.058	3.70E-12
Endothelial cells	0.558	0.0656	1.80E-17
Neurons	0.266	0.0384	4.30E-12
Microglia	1.66	0.176	4.03E-21
Neurons	1.52	0.163	1.11E-20
Endothelial cells	2.1	0.313	1.96E-11
Microglia	-0.53	0.0382	9.06E-44
Neurons	-0.444	0.0422	6.89E-26
Endothelial cells	-0.564	0.0699	7.11E-16
